# Experimental and computational insights into the mechanism of the copper(i)-catalysed sulfonylative Suzuki–Miyaura reaction†

**DOI:** 10.1039/d3sc01337e

**Published:** 2023-05-31

**Authors:** Callum G. J. Hall, Helen F. Sneddon, Peter Pogány, David M. Lindsay, William J. Kerr

**Affiliations:** a Medicines Design, GlaxoSmithKline Gunnels Wood Road, Stevenage SG1 2NY England UK; b Department of Pure and Applied Chemistry, University of Strathclyde 295 Cathedral Street, Glasgow G1 1XL Scotland UK w.kerr@strath.ac.uk

## Abstract

A mechanistic study into the copper(i)-catalysed sulfonylative Suzuki–Miyaura reaction, incorporating sulfur dioxide, is described. Utilising spectroscopic and computational techniques, an exploration into the individual components of the competing catalytic cycles is delineated, including identification of the resting state catalyst, transmetalation of arylboronic acid onto copper(i), the sulfur dioxide insertion process, and the oxidative addition of aryl halide to Cu^I^. Studies also investigated prominent side-reactions which were uncovered, including a competing copper(ii)-catalysed mechanism. This led to an additional proposed and connected Cu^I^/Cu^II^/Cu^III^ catalytic cycle to account for by-product formation.

## Abstract 2

A detailed study into the copper(i)-catalysed sulfonylative Suzuki–Miyaura reaction, using spectroscopic and computational techniques. The study explores key mechanistic steps and side-reactions, including a competing copper(ii)-catalysed mechanism.

## Graphical abstract

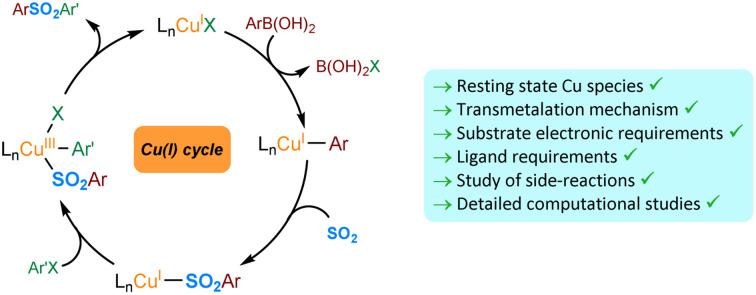

## Introduction

**Fig. 1:**
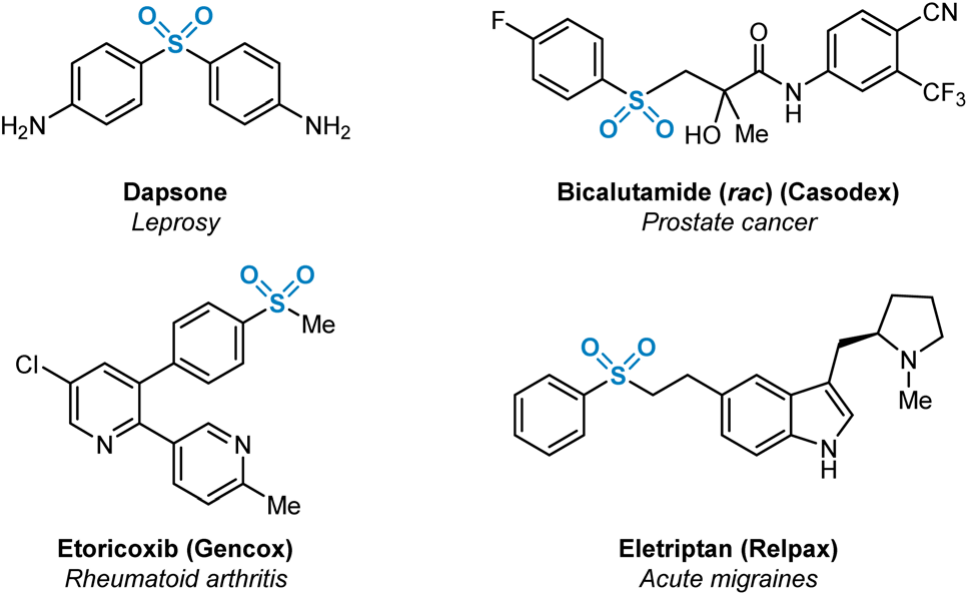
Selected marketed pharmaceuticals containing sulfur(vi) functionality in the form of sulfone (R-SO_2_-R′) functional groups.

Sulfur(vi)-containing functional groups are prevalent within pharmaceutical compounds as has been evident for some time within both the scientific literature and patents.^1^ The beneficial physicochemical properties of molecules containing such functional groups, most notably sulfones and sulfonamides, have been extensively studied. These favourable properties are related primarily to increased target specificity *via* hydrogen bonding with protein active site residues, or the utilisation of sulfur(vi) moieties as metabolically resistant linker groups between aromatic and heteroaromatic fragments.^2^ Examples of medicinally-relevant sulfone-containing pharmaceuticals are shown in Fig. 1.

**Scheme 1:**
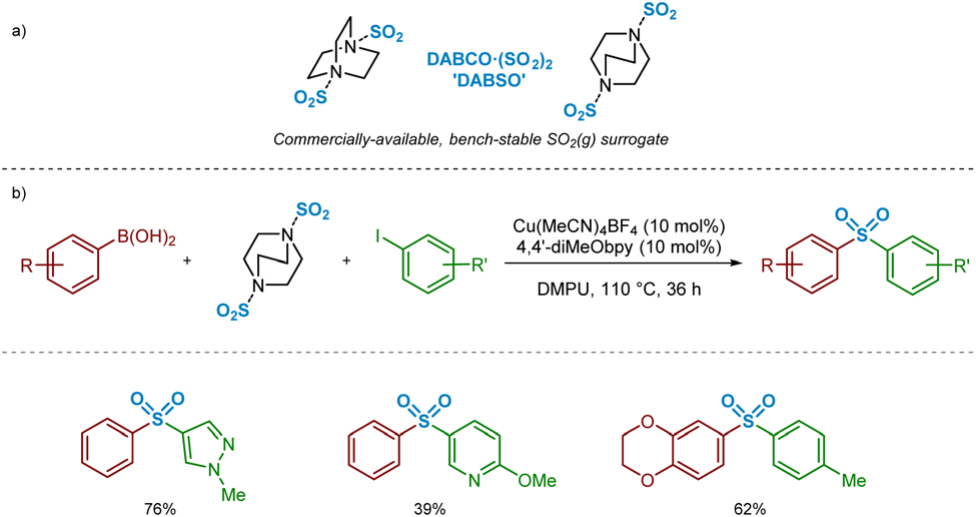
Copper(i)-catalysed sulfonylative Suzuki–Miyaura reaction for the preparation of biaryl sulfones (Chen and Willis, *Chem. Sci.*, 2017).^4^

In the past decade, there have been a number of significant developments in the methods available for introduction of sulfur(vi) functionality into molecules, with the aim of facilitating more efficient preparation of sulfones for medicinal chemistry applications.^3^ As part of these advances, a landmark publication by Chen and Willis in 2017 describes a novel copper(i)-catalysed sulfonylative Suzuki–Miyaura transformation in which arylboronic acids and aryl iodides are able to react, with the insertion of sulfur dioxide, to yield a range of medicinally-relevant sulfones, Ar–SO_2_–Ar′.^4^ Furthermore, this was one of several recently reported transformations using a bench-stable surrogate of sulfur dioxide,^3^ 1,4-diazabicyclooctane·(SO_2_)_2_ (‘DABSO’).^5^ As detailed in Scheme 1, the use of an inexpensive copper(i) catalyst, Cu(MeCN)_4_BF_4_, in combination with the commercially available electron-rich 4,4′-dimethoxy-2,2′-bipyridine (4,4′-diMeObpy) ligand at relatively low catalyst loadings results in a catalytic transformation highly favourable for accessing biaryl sulfones. A selection of pharmaceutically-relevant sulfone products synthesised by Chen and Willis, with their associated yields, is shown in Scheme 1.

Also described was diversification chemistry involving reaction of arylboronic acids in the presence of NaBF_4_ within the Cu^I^-catalysed process with DABSO, which is presumed to proceed *via* trapping of the intermediate reactive species in the form of a sulfinate.^4^ This alternative preparative approach can be used to synthesise other medicinally-relevant sulfur(vi) compounds, including alkylsulfones, sulfonamides, and sulfonyl fluorides.

At present, limitations of the described Cu^I^-catalysed processes are perceived to include the somewhat reduced performance of certain electron-deficient and heteroaryl iodides, and issues associated with the use of pyridyl boronic acids due to their inherent instability within such reaction manifolds.^6^

Throughout investigations within our laboratories searching for and optimising novel and more environmentally-acceptable methods for incorporation of sulfur dioxide into medicinally-relevant small molecules, a number of key observations were made when utilising Willis' sulfonylative Suzuki–Miyaura chemistry, including notable by-product formation. This led to a detailed investigation of the reaction mechanism, both experimentally and computationally, with the aim of revealing vital information to aid the optimisation of such catalytic processes.

Herein is described a rigorous investigation of individual reaction steps and side reactions of the copper(i)-catalysed sulfonylative Suzuki–Miyaura coupling process using sulfur dioxide. Given the complexity of the perceived reaction pathways, involving multiple reaction steps favoured by differing sets of conditions, this communication aims to both complement and enhance previous studies in the field of copper(i)-catalysed sulfonylative cross-coupling transformations.^4,7–9^ Using a combination of experimental observations and theoretical justification, we believe that this work provides a key contribution towards a predictive approach relating to ligand and substrate selection as associated with individual requirements in transformations involving copper(i)-catalysed transmetalation, migratory insertion, oxidative addition, and reductive elimination as part of sulfonylative processes.

## Results & discussion

Based on the perceived preparative value of the copper-based sulfonylative Suzuki–Miyaura reaction and the potential to further expand the synthetic utility of this transformation, coupled with the requirement for enhanced mechanistic understanding of this emerging process, a detailed investigation was initiated, utilising both experimental and computational evidence. Each individual reaction step was investigated in turn, and as discussed in the sections below. During the preparation of this manuscript, complementary studies into a related copper(i)-catalysed sulfonylative Hiyama cross-coupling revealed outcomes that support our observations as related to specific aspects of the reaction mechanism which are shared between both processes.^10^ These comparisons will be discussed, as appropriate, within the relevant sections.

### Building a mechanistic hypothesis

For the mechanistic elucidation of the copper(i)-catalysed sulfonylative Suzuki–Miyaura reaction, a number of key points require consideration. Based on the original paper by Willis,^4^ and a publication describing related examples,^11^ the overall conversion of boronic acid to sulfone is likely to involve a sulfinate intermediate. Additionally, from the substrate scope as published it would appear that electron-deficient boronic acids may perform somewhat less efficiently in the transformation, representing a known challenge that has been addressed in related palladium- and nickel-catalysed cross-coupling processes.^12–14^

**Fig. 2:**
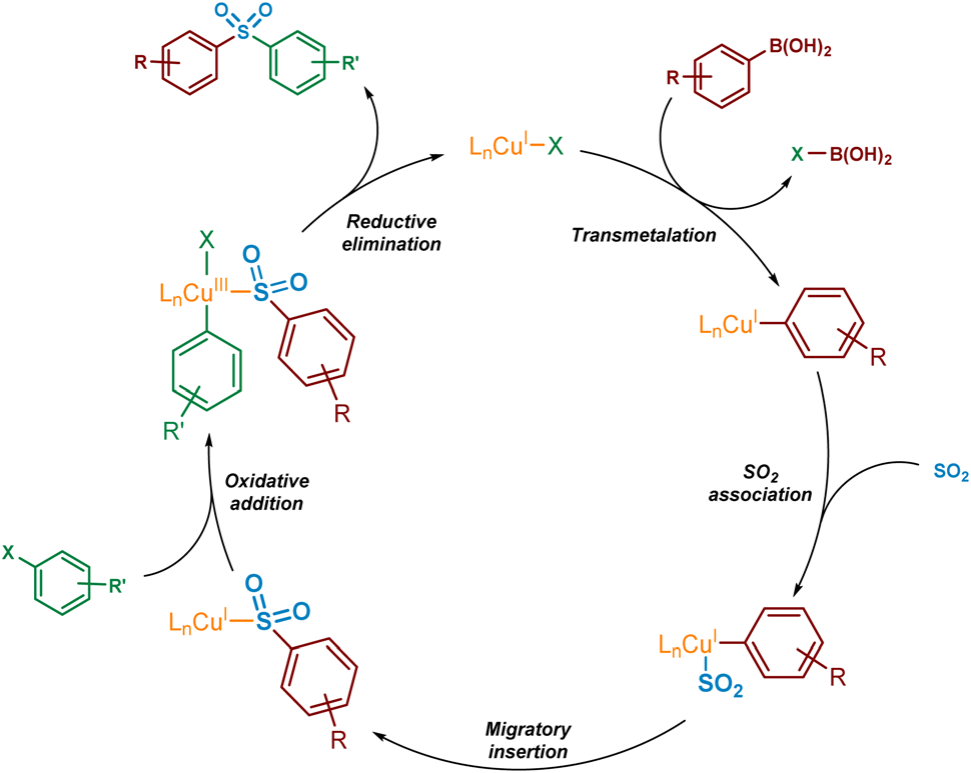
Simplified potential mechanism, based on existing literature precedent, for the Cu(i)-catalysed sulfonylative Suzuki–Miyaura reaction.

For copper(i) catalysis, there is known to be a reversal in order of transmetalation and oxidative addition steps of the catalytic cycle when compared with traditional palladium-catalysed cross-couplings.^15^ The higher activation barriers associated with the oxidative addition of copper(i) into carbon-halogen bonds will result in the transmetalation of the boronic acid onto copper(i) occurring first.^16^ This transmetalation step could occur either before or after the binding of SO_2_ to the copper species. Also, due to the instability of the copper(iii) species formed upon oxidative addition into a carbon–halogen bond, this species is also likely to be extremely short-lived. As a result, it is expected that a rapid reductive elimination would occur to yield the sulfone product. A simplified version of a potential catalytic cycle is shown in Fig. 2.

### Identification of a sulfinate intermediate

**Fig. 3:**
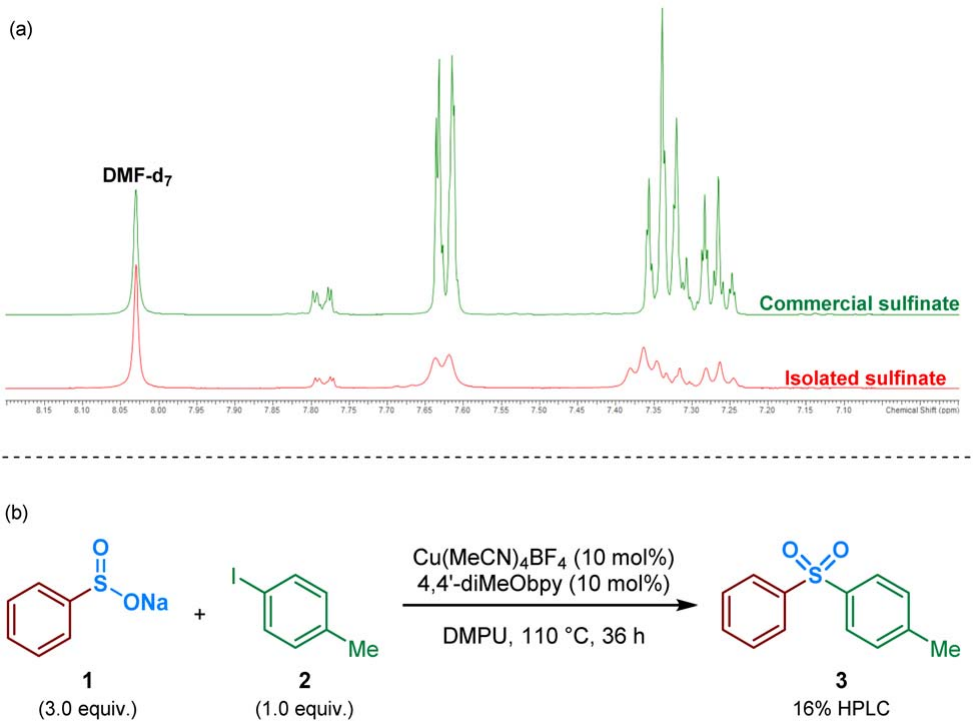
Identification and isolation of a benzenesulfinate intermediate in the Cu(i)-catalysed sulfonylative Suzuki–Miyaura reaction. (a) Isolation of a benzenesulfinate intermediate: ^1^H NMR comparison with a commercial sodium benzenesulfinate source; due to the quadrupolar ^63^Cu and ^65^Cu nuclei present within the aqueous layer from which the sulfinate was isolated, the ^1^H resonances observed are broad and unresolved. (b) Test reaction of sodium benzenesulfinate with 4-iodotoluene.

As an initial approach to probing the mechanism, and due to the prevalence of a sulfinate intermediate being proposed within the majority of published literature on DABSO-based transition metal catalysis, we attempted to isolate an intermediate sulfinate species from the reaction mixture. This was achieved by trapping the benzenesulfinate, arising from phenylboronic acid, with an NaBF_4_ additive, followed by acidification and neutralisation of the organic phase during work-up. The isolated solid, obtained by concentration of the aqueous phase, yielded a comparable ^1^H NMR spectrum to the commercial sodium benzenesulfinate (Fig. 3a).

Following the isolation of a benzenesulfinate species, the coupling process was attempted using an intermediate sodium sulfinate salt. Exposing sodium benzenesulfinate 1 and 4-iodotoluene 2 to the reaction conditions divulged by Willis,^4^ provided the desired sulfone product 3 in 16% HPLC yield (Fig. 3b).

In addition, the presence of the sulfinate intermediate was also observed by LCMS and HRMS analysis of the crude reaction mixture after stirring phenylboronic acid 4, Cu(MeCN)_4_BF_4_, 4,4′-diMeObpy, and DABSO in DMPU for 2 h. LCMS and HPLC data from these investigations can be found in the Experimental ESI Section 4.1.3., ES13–15.†

### Identification and analysis of copper(i) intermediates

With the aim of deducing the mechanism of the catalytic process, we initially opted to identify intermediate copper species using spectroscopic methods. Aliquots from a mixture of Cu(MeCN)_4_BF_4_ and 4,4′-diMeObpy with DABSO in DMPU were analysed by LCMS after 2 h at 110 °C. From the resulting mass spectra, a number of the potential resting-state catalytic species were identified within the mixture. Specifically, a mass peak corresponding to a Cu(4,4′-diMeObpy)^+^ species 5, and a solvated variant, Cu(4,4′-diMeObpy)(DMPU)^+^6, supported an assumption that the resting state Cu^I^ catalyst species could be this latter 16 valence electron species. An additional complex containing the organic base, Cu(4,4′-diMeObpy)(DABCO)^+^7, was also observed in low abundance. Full experimental details, and LCMS and HRMS analysis of these species are documented in Experimental ESI Section 4.2.1., ES15–17.†

**Fig. 4:**
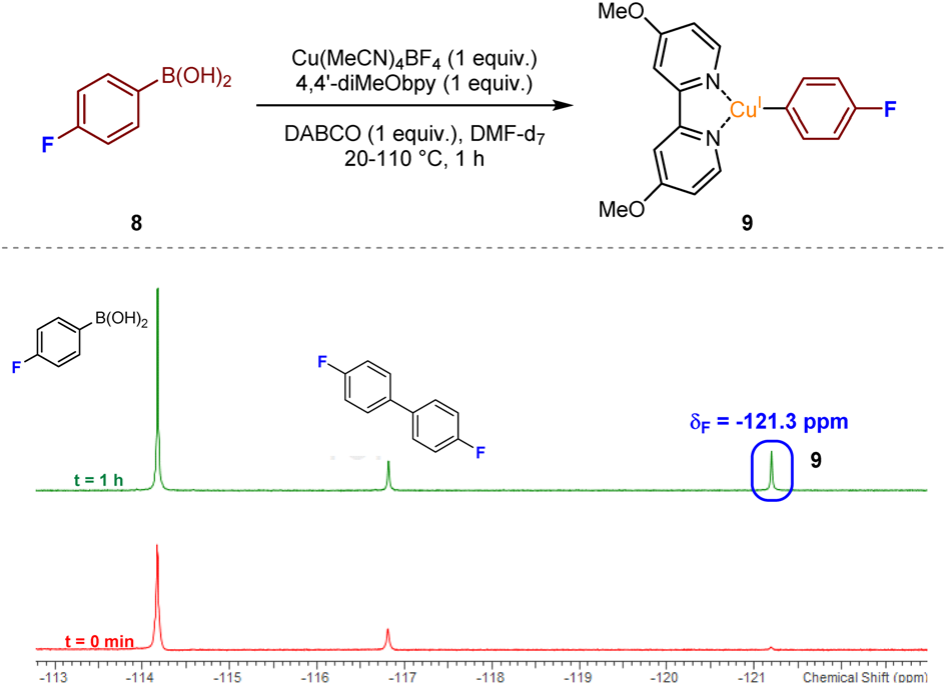
Detection of a transient post-transmetalation Cu(i)-aryl species *via*^19^F NMR; conditions: 4-fluorophenylboronic acid (0.05 mmol), Cu(MeCN)_4_BF_4_ (0.05 mmol), 4,4′-diMeObpy (0.05 mmol), DABCO (0.05 mmol), DMF-d_7_ (0.5 mL), N_2_(g), 20–110 °C, 1 h, followed by ^19^F NMR analysis.

Detection of additional copper-containing intermediates within the reaction mixture was found to be difficult due to the inherent instability or short-lived nature of such Cu^I^ or Cu^III^ species. There have been a few reports of LCu(Ar) species, where L has a similar backbone to a bipyridyl unit (generally 1,10-phenanthroline), often requiring the aryl ring to be polyfluorinated to enhance stability.^17^ Thus, we opted to use ^19^F NMR for the attempted detection of an LCu(Ar) species, with reaction of 4-fluorophenylboronic acid 8 our preferred choice, given that the use of, for example, a more heavily fluorinated phenylboronic acid would likely lead to low transmetalation reactivity.^12^ Upon mixing of equimolar ratios of 4-fluorophenylboronic acid 8, Cu(MeCN)_4_BF_4_, 4,4′-diMeObpy, and DABCO, in DMF-d_7_ with stirring at over the temperature range 20–110 °C for approximately 1 h, emergence of a resonance at *δ*_F_ −121.3 ppm would appear to confirm the presence of the copper(i) aryl species 9, in accordance with literature precedent (Fig. 4).^18^ A similar NMR experiment using DABSO as a replacement for DABCO, introducing SO_2_ into the reaction mixture, also revealed the presence of intermediate fluorinated arylsulfinate species, LCu(SO_2_Ar), at *δ*_F_ −113.9 and −115.5 ppm (see Experimental ESI, Section 4.4.2., ES27–28†). The absence of a resonance at *δ*_F_ −121.3 ppm for the copper(i) aryl species in this NMR experiment suggests that upon introducing SO_2_ into the reaction mixture, the copper(i) aryl species is rapidly converted to the intermediate sulfinate species *via* migratory insertion.

### Arylboronic acid transmetalation with copper(i)

Following our observation, as shown in Fig. 3b, that the conversion of the arylsulfinate to the biaryl sulfone, does not require an amine base, it can be proposed that the conversion of the arylboronic acid to arylsulfinate requires such a base species to be present in stoichiometric quantities. This is also supported by a publication from Baskin and Wang disclosing an efficient method for the Cu^I^-catalysed transformation of aryl sulfinates and aryl iodides into biaryl sulfones, where the diamine (DMEDA, 10 mol%) is only thought to behave as a ligand.^7^

Little is known surrounding transmetalation processes involving the copper(i) oxidation state, in contrast to copper(ii) (Chan–Evans–Lam)^19^ and Pd(0/ii) (Suzuki–Miyaura & related) reactions.^20^ Our initial thoughts surrounding the nature of this process involved Lewis-base activation of the arylboronic acid with the amine base, DABCO, in a similar fashion to that observed within Pd(0/ii) catalysis.^21^ However, a test reaction involving the mixing of arylboronic acid and DABCO under reaction-like conditions revealed rapid formation of a boroxine (boronic acid anhydride) which was coordinated to DABCO – literature evidence for the formation of such species exists, where they behave as fluctional systems under NMR conditions.^22^ We also observed that 2,4,6-triphenylboroxine performed relatively poorly in the Cu(i)-catalysed coupling process when compared to phenylboronic acid (see Experimental ESI, Section 4.3.1., ES17–18†). Given these observations, action of the amine base is unlikely to play a prominent role as part of arylboronic acid activation within this reaction manifold.

**Fig. 5:**
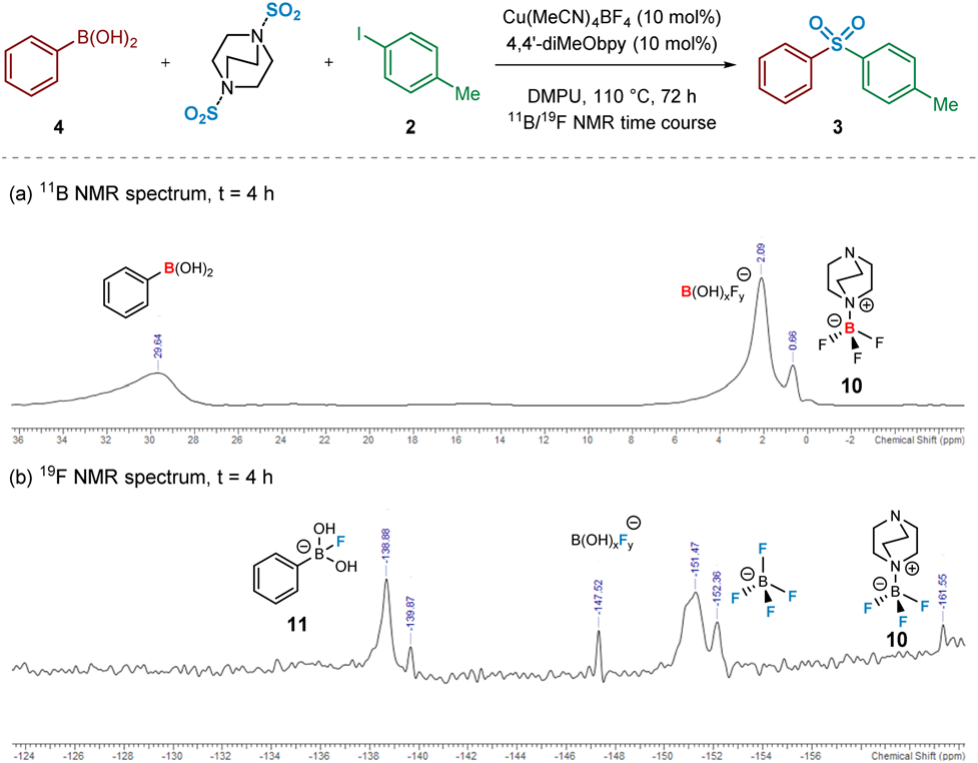
Heteronuclear NMR time course of a model sulfonylative Suzuki–Miyaura reaction for identification of reaction intermediates. (a) ^11^B NMR spectrum of the reaction mixture after 4 h. (b) ^19^F NMR spectrum of the reaction mixture after 4 h. Conditions for (a) and (b): phenylboronic acid (0.6 mmol), 4-iodotoluene (0.2 mmol), DABSO (0.3 mmol), Cu(MeCN)_4_BF_4_ (0.02 mmol, 10 mol%), 4,4′-diMeObpy (0.02 mmol, 10 mol%), DMPU (1 mL), 110 °C, 4 h, followed by ^11^B and ^19^F NMR analysis.

Intrigued, we next turned to the use of ^11^B and ^19^F NMR to track the reaction pathway, given that fluoride (F^−^) has been known to act as a Lewis-base activator within metal-catalysed processes,^23^ and that tetrafluoroborate (BF_4_^−^) is inherently prone to hydrolysis.^24^ An NMR time course studying these nuclei at intervals over 72 h was carried out in order to attempt identification of reaction intermediates, and hence deduce the reaction mechanism for the initial transmetalation step. In particular, the ^11^B and ^19^F NMR spectra recorded at *t* = 4 h revealed important information surrounding the nature of activation of the arylboronic acid species (Fig. 5). Based on literature ref. 25–27 and independent preparation of the fluoride “ate complex” of the boronic acid, it appears that the mechanism is proceeding by liberation of fluoride from BF_4_^−^, alongside the DABCO present, forming the BF_3_-DABCO species 10. This is followed by the activation of the arylboronic acid with liberated fluoride to yield arylfluoroboronate 11. The B(OH)_2_F species subsequently formed by the transmetalation step is thought to exist in equilibrium between neutral B(OH)_3_ and anionic BF_4_^−^, both species being observed at the final ^11^B and ^19^F NMR timepoints; see Experimental ESI Sections 4.3.3. and 4.3.4., ES20–22.†^25^

To further test the hypothesis in which BF_4_^−^ is liberating the activating F^−^ for transmetalation, a screen was carried out in which the nature of the copper(i) counterion was varied, using a number of commercially available tetrakis(acetonitrile) copper(i) salts (Table 1). Interestingly, exchange of the counteranion of the copper species from BF_4_^−^ to PF_6_^−^ resulted in a reduction in HPLC yield to 2%, suggesting BF_4_^−^ is necessary for reaction. This was supported by the reported hydrolysis rates for both ions, which suggest that liberation of fluoride is much more likely with tetrafluoroborate, whilst hexafluorophosphate anions are essentially inert.^24^ However, when the transformation was carried out with the copper(i) tetrafluoroborate counter-ion species in combination with stoichiometric quantities of inorganic fluorides (KF, AgF; see Experimental ESI Section 4.3.6., ES22–23†), inhibition of the reaction was observed. This inhibition could potentially arise from the highly favourable formation of copper fluoride salts,^28^ in turn deactivating the copper(i) catalyst. As a comparison, the use of Cu(MeCN)_4_OTf, containing a counterion unable to liberate fluoride for activation, also performs less effectively that of BF_4_^−^, although some boronic acid activation does appear to be occurring with this species (Table 1, entry 2).

**Table 1:** Effect on yield of sulfone 3 by variation of the tetrakis(acetonitrile) copper(i) counterion. Conditions: phenylboronic acid (0.3 mmol), 4-iodotoluene (0.1 mmol), DABSO (0.15 mmol), Cu(MeCN)_4_BF_4_ (0.01 mmol, 10 mol%), 4,4′-diMeObpy (0.01 mmol, 10 mol%), DMPU (0.5 mL), 110 °C, 36 h. Yields were determined by HPLC (*λ* = 235 nm) using dimethyl terephthalate as an internal standard

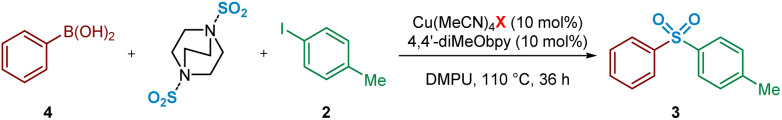		
Entry	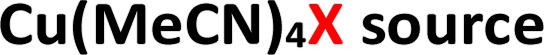	HPLC yield 3 (%)
1	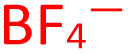	41
2	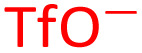	25
3	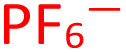	2

Modelling the transmetalation process computationally was challenging due to the multicomponent nature of the reaction system; the copper(i) species, boronic acid, tetrafluoroborate, and DABCO all play a role in this key step. After multiple attempts including using the amine as a Lewis-base activator, and direct activation by tetrafluoroborate, we were able to model a reaction scheme consistent with the experimental and spectroscopic investigations detailed above, in which a pre-formed phenylboronic acid fluoride ‘ate’ complex, PhB(OH)_2_F^−^11, forms a stable cation–π adduct with the copper(i) species prior to transmetalation (see Computational ESI Section 2.1, CS2–3†). This modelling study then led to the liberation of fluoroboric acid, B(OH)_2_F, and a phenylcopper(i) species similar to 9.

Following the transmetalation step, the energetically favourable association of sulfur dioxide to the copper–aryl species occurs. This ordering of transmetalation followed by SO_2_ binding to the copper(i) centre has also been proposed as a result of the observations by Cantat and colleagues.^10^ Through a combination of computational calculations^29^ and literature precedent,^30,31^ it is strongly believed that this species has a coordination mode of planar η^1^-SO_2_ binding through the sulfur atom. This is commonly seen with first row transition metals.^32^

### Migratory insertion of SO_2_ into the copper–carbon bond

The substrate scope reported by Willis and Chen may suggest that electron-deficient boronic acid species perform less effectively in the sulfonylative Suzuki–Miyaura coupling reaction.^4^ In addition to the previously discussed transmetalation step, these lower yields might also be rationalised by considering the key Cu–C/SO_2_ migratory insertion stage. A publication from Vitzthum and Lindner, highlighting the analysis and characterisation of metal–sulfinate complexes, discusses the lowered tendency for SO_2_ to insert into a metal–carbon bond where the carbon unit possesses electron-withdrawing properties.^33,34^ More specifically, this analysis proposes that the less effective insertion of SO_2_ into a metal–carbon bond in the presence of strongly electron-withdrawing groups is due to resonance effects between the metal atom and the electron-withdrawing unit, strengthening the metal–carbon bond.

**Scheme 2:**
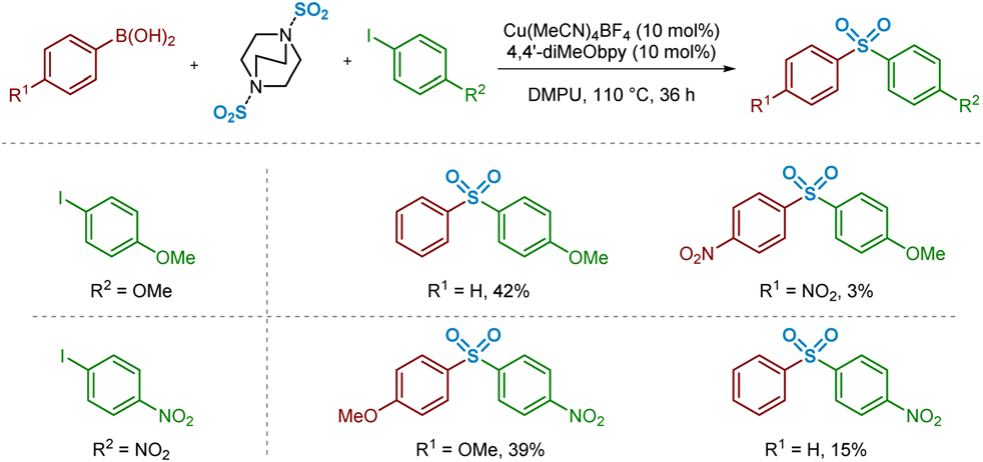
Experimental observations informing the migratory insertion of sulfur dioxide into the copper–aryl bond. Isolated yields from sulfonylative Suzuki–Miyaura reactions using a selection of electron-rich, -neutral, and -poor arylboronic acids. Conditions: arylboronic acid (1.2 mmol), aryl iodide (0.4 mmol), DABSO (0.6 mmol), Cu(MeCN)_4_BF_4_ (0.04 mmol, 10 mol%), 4,4′-diMeObpy (0.04 mmol, 10 mol%), DMPU (2 mL), 110 °C, 36 h. Isolated yields.

In order to compare this reactivity experimentally, a small number of combinations of electron-rich and -poor boronic acids were compared with aryl iodide coupling partners (Scheme 2). It appears that, based on the isolated yields obtained, the electron-deficient boronic acid performs poorly as a cross-coupling partner, whereas use of electron-neutral (Ar = phenyl) or -rich (Ar = 4-methoxyphenyl) boronic acids are better tolerated. These differences in isolated yield of the desired sulfone may arise from a combination of this migratory insertion effect alongside the more facile transmetalation of electron-rich boronic acids onto copper, a well-known electronic requirement of transmetalation.^13^

**Fig. 6:**
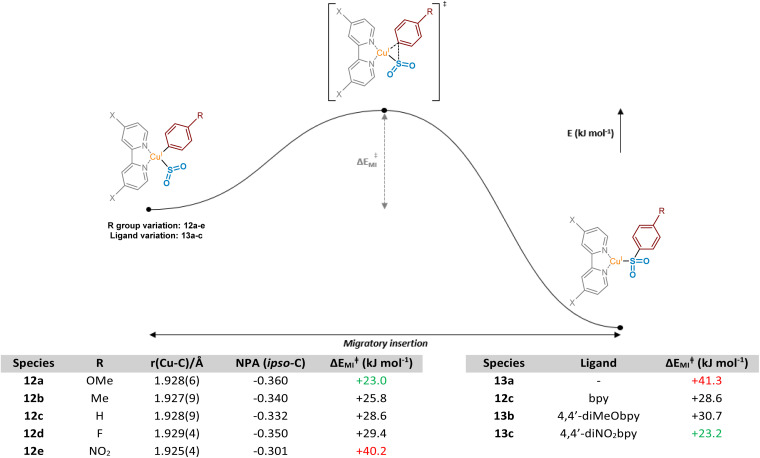
Computational observations relating to the migratory insertion of sulfur dioxide into the copper–aryl bond. Computed values of copper–carbon bond length (Å), NPA atomic charge for the *ipso* (migrating) carbon atom, and dependence of migratory insertion activation energy on R group and complex ligand; for 12c and 13a–c, R = H.

These observed experimental results were additionally explored computationally, first through visualisation of migrating carbon Natural Population Analysis (NPA) atomic charges and the corresponding Cu–C bond length in a series of (bpy)Cu(Ar)(SO_2_) complexes 12a–12e (where Ar is a series of aryl rings, modified at the *para*-position) (Fig. 6, Computational ESI Section 2.2, CS3†). The optimised structures generated were then used in subsequent calculations to determine aryl substituent dependence on the activation barrier to the migratory insertion reaction (see Computational ESI Section 2.3, CS5†). The benchmark for comparison was chosen to be that with Ar = phenyl, (bpy)Cu(Ph)(SO_2_) 12c, with a copper–carbon bond length of 1.928(9) Å, an NPA atomic charge of −0.332, and a migratory insertion activation energy of +28.6 kJ mol^−1^.

Electronic effects appear to have a small impact on the length, and presumably the strength, of the Cu–C bond. When the *para-*substituent of the aryl ring is electron-donating in comparison to H (R = OMe 12a, R = Me 12b), there is a slight shortening of the bond, suggesting the electron-donating groups are also able to contribute through resonance or σ-donor effects to the copper species, possibly with vacant valence orbitals on the metal acting as acceptors. Perhaps more importantly, these groups also show a build-up of NPA atomic charge on the migrating carbon atom, increasing the nucleophilicity and likely tendency to migrate (NPA atomic charges of −0.360 and −0.340, respectively, for R = OMe 12a and Me 12b). Introduction of these electron-donating groups has also been shown to lower the activation barrier for migratory insertion with respect to electron-neutral groups, with the 4-methoxyphenyl and 4-methylphenyl units having +23.0 and +25.8 kJ mol^−1^ activation energies, respectively. These observations are in line with those reported for insertion of SO_2_ preferentially into metal-tolyl bonds over metal-phenyl bonds in equivalent Ar^1^–Hg–Ar^2^ complexes.^35^

With π electron-withdrawing groups, such as in 12e, R = NO_2_, resonance contributions appear to lead to donation of electron density from copper into the aryl ring, resulting in shortening and strengthening of the Cu–Ar bond. This effect then leads to an increase in the subsequent migratory insertion activation energy to +40.2 kJ mol^−1^. This also links very well with the apparent depletion of NPA atomic charge on the *ipso*-carbon of 12e (−0.301).

Interestingly, a switch to a σ-electron-withdrawing group (Ar = 4-fluorophenyl 12d) results in a lengthening of the Cu–C bond. We propose that the presence of a 4-fluoro group on the phenyl ring results in σ-withdrawal of electrons from the copper–carbon bond, leading to subsequent lengthening of this bond. Moreover, the effect of this σ-electron-withdrawal results in a less nucleophilic carbon for migration and, as a result, species 12d has a slightly higher Δ*E*_MI_ value than 12c (Ar = Ph), although less significant compared with when π-backbonding resonance effects are involved (12e, Ar = 4-nitrophenyl).

Further computational probing of this key migratory insertion step involved observing the electronic demands of the ligand on the copper-sulfinate species, (L)Cu(Ph)(SO_2_) 12c, 13a, 13b, and 13c. Using a small number of substituted electron-rich, -neutral, and -deficient bipyridyl ligands, in addition to the process in the absence of a ligand, activation energies were calculated (see Computational ESI Section 2.4, CS5–6†). Initial results showed the strong requirement for a coordinating ligand such as bipyridyl, with a large drop in activation energy for the insertion reaction from +41.3 to +28.6 kJ mol^−1^ upon the introduction of bpy as a ligand, as seen between 13a and 12c. Interestingly, introduction of electron-donating groups onto the bpy at the 4- and 4′-positions in 13b, as found by Willis to be effective in the overall sulfonylative Suzuki–Miyaura transformation,^4^ would appear to actually inhibit this stage of the catalytic cycle when compared to bpy, 12c. Switching to the 4,4′-dinitro-2,2′-bipyridine (4,4′-diNO_2_bpy) ligand lowers the activation barrier, suggesting that electron-deficient bipyridyls (13c) are preferred for the migratory insertion. Withdrawal of electron density from the breaking copper–carbon bond in this step likely results in a weakening of the bond, facilitating migration. This is observed in the geometry-optimised structures of (4,4′-diNO_2_bpy)Cu(Ph)(SO_2_) 13c and (4,4′-diMeObpy)Cu(Ph)(SO_2_) 13b, with respective Cu–C bond lengths of 1.929(4) and 1.928(6) Å (see Computational ESI Section 2.2.2, CS4–5†).

### Oxidative addition of copper(i) into the carbon–iodine bond

**Fig. 7:**
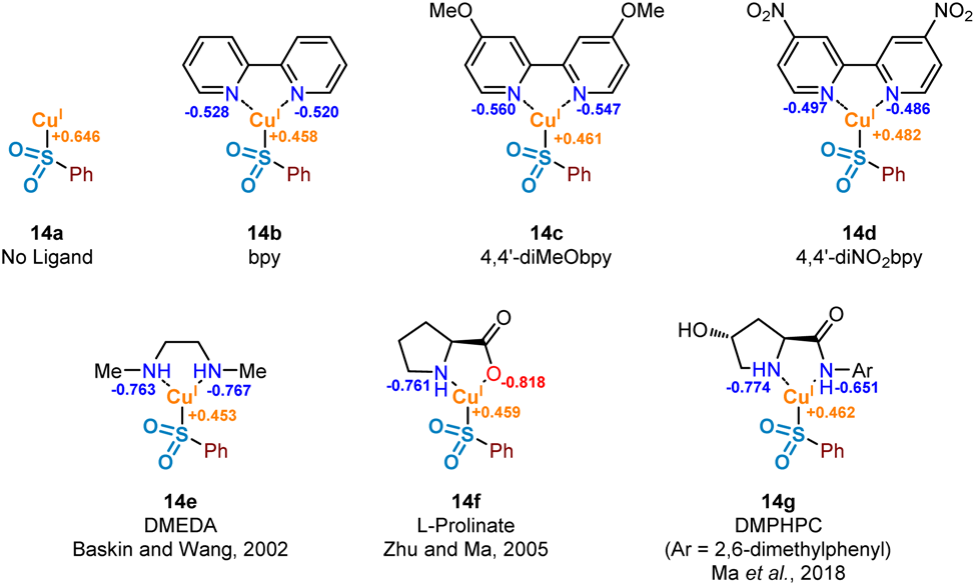
Computational investigations of the oxidative addition of Cu(i) into the carbon–iodine bond of aryl iodides. Computed natural population analysis (NPA) charges for three electronically-varied bipyridyl ligands, in addition to three literature-reported efficient ligands for copper(i)-catalysed sulfinate couplings.

It has been proposed that the rate determining step associated with copper(i) catalysis for the functionalisation of carbon–halogen bonds is the oxidative addition process involving the C–X bond, with trends in reactivity favouring C–I bonds over C–Br and C–Cl bonds.^36^ Indeed, as part of this study we have observed the following trend in relation to the aryl halides employed in the formation of product 3: ArI > ArBr > ArCl (see Experimental ESI Section 4.5.1., ES29–30†). Early examples involving stoichiometric Cu^I^ (Ullmann^37^ and Goldberg reactions,^38^ for C–C, C–O and C–N bond formation) highlight the magnitude of this activation barrier, with some reactions requiring temperatures in excess of 200 °C. This energy barrier is likely to be a result of the inherent instability of the Cu^III^ species formed, with very few reported characterisations of compounds containing this high oxidation state.^39^ Identification of an accelerating effect in copper(i)-catalysed reactions when organic ligands are incorporated suggests a lowering of the activation barrier to oxidative addition.^40^ These ligands, typically containing electron-donating oxygen and nitrogen units as a means of stabilising the higher oxidation states, include bipyridyl, phenanthroline, and ethylenediamine derivatives; a selection of these ligands, 14b–14g, can be seen in Fig. 7.

A number of publications in the last 20 years have focused on the utility of sulfinate salts in copper-catalysed cross-coupling reactions with aryl halides. The first of these, in 2002, used a combination of copper(i) triflate benzene complex and *N*,*N*′-dimethylethylenediamine (DMEDA) (see complex 14e) for the efficient synthesis of arylsulfones from aryl iodides.^7^ This alkyl diamine ligand is likely to have been chosen as a means to increase electron density on the copper(i) centre, thus facilitating the key oxidative addition step. Following on from this, a series of more recent investigations have been published by Ma and co-workers, optimising the coupling process with sulfinate salts further.^8,9^ A switch of ligand system to l-proline (as in 14f) and l-hydroxyproline derivatives, such as DMPHPC (as in 14g), has allowed for tolerance of aryl bromides, in addition to a lowering of reaction temperature from 110 °C to as low as room temperature. Use of these l-hydroxyproline derivatives has also been demonstrated to have utility with more challenging aryl chloride substrates, although requiring elevated temperatures of 120 °C as a result of a more demanding oxidative addition into the C–Cl bond.^41^ From these reports, a number of conclusions can be drawn surrounding the catalytic requirements of the ligand used. Most specifically, it appears that bidentate ligands of a highly electron-donating nature are most favourable.

In order to investigate the ligand effect on the step involving conversion of aryl sulfinate to sulfone, initial NPA (natural population analysis) charge calculations were carried out on eight geometry-optimised copper(i)-sulfinate species 14a–14g, with the aim of visualising the electron density present on the copper atom (Fig. 7, Computational ESI Section 2.6, CS7†). Comparing with an NPA charge of +0.646 for the ligandless sulfinate species CuSO_2_Ph 14a, complexation of all ligands delivered an increase in electron density, supporting the suggestion that an electron-rich copper favours oxidative addition into a carbon–halogen bond. Although the observed variation in electron density across the selection of ligands bound to the copper(i) sulfinate species appears minimal, the extraction of some general trends is possible. As expected, introduction of electron-donating (X = OMe 14c, Cu NPA = +0.461) or electron-withdrawing (X = NO_2_14d, Cu NPA = +0.482) groups to the bipyridyl ring system result in a more or less electron-rich Cu species. The very small difference between the calculated copper NPA values in the bpy and 4,4′-diMeObpy complexes 14b and 14c may explain why these ligands were observed as broadly comparable in Willis' optimisation notes (38% *versus* 49% HPLC yield of the model substrate used in this study),^4^ although participation in the overall mechanism will include an influence at not only the oxidative addition step. For the latter three ligands 14e–14g in Fig. 7, chosen from literature-precedented sulfinate couplings,^7–9^ these all result in a comparably or slightly more electron-rich copper centre, which can be expected to facilitate the oxidative addition into the required carbon–halogen bond.

**Fig. 8:**
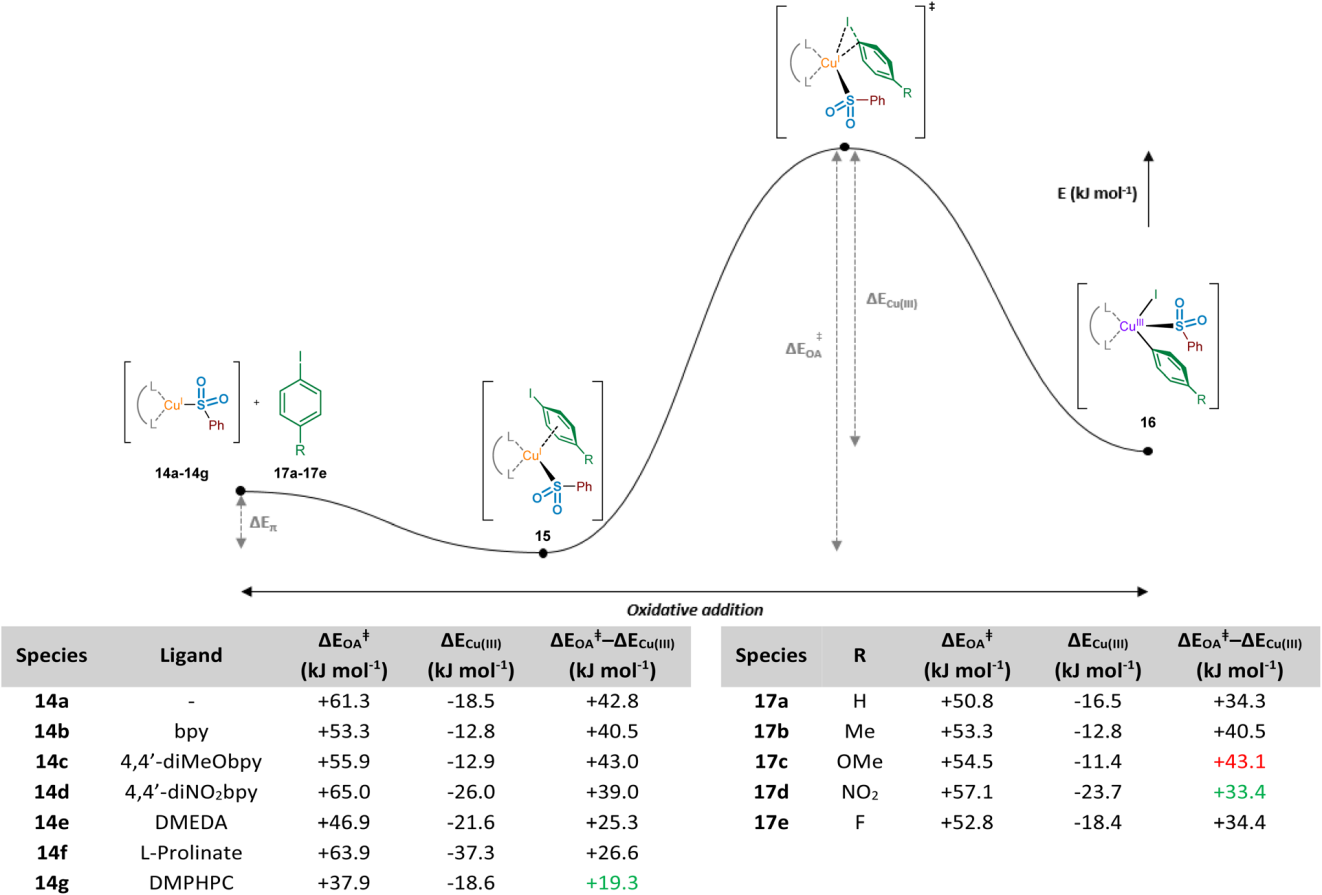
Computational investigations of the oxidative addition of Cu(i) into the carbon–iodine bond of aryl iodides. Computed energy profile for the oxidative addition process, exploring effects of ligand and aryl iodide R group on the activation energy and stability of the resultant Cu(iii) species; left-hand table, ArI = 4-iodotoluene 17b (R = Me) employed; and right-hand table, bpy employed.

To further enhance our understanding of the electronic requirements of the resting state catalyst species for oxidative addition, we modelled the oxidative addition of each copper(i) complex into the C–I bond of 4-iodotoluene, 17b (see Computational ESI Section 2.7, CS7–8†). Using previous literature precedent in the field of copper(i)-catalysed carbon–heteroatom bond forming reactions,^42^ in addition to our experimental NMR observations of the behaviour of sodium sulfinates in the presence of Cu(MeCN)_4_BF_4_, where a shift in the ^19^F resonance of 4-fluorophenylsulfinate has been observed compared with that of the sodium sulfinate (Experimental ESI Section 4.4.3., ES28–29†), there is a high level of confidence that the phenylsulfinate is copper(i)-bound prior to this oxidative addition. Additionally, having conducted initial computational studies, with and without the sulfinate bound to the copper, the sulfinate-bound species delivered oxidative addition transition state energies which aligned more realistically with the precedented values cited previously within the scientific literature.^43^ Importantly, throughout these investigations we discovered the presence of a potential energy well prior to oxidative addition, in which the respective copper(i) sulfinate species forms an appreciably stabilised cation–π complex 15 with the π system of the aryl iodide; such a stabilising interaction has been proposed for copper catalysis in the chemical literature previously.^44^ The computed activation energies for oxidative addition (Δ*E*_OA_) and the relative stabilities of the Cu^III^ species compared to the transition state (Δ*E*_Cu(III)_) upon variation of the chelating ligand are shown in Fig. 8. It is also important to consider the overall reaction energy starting specifically from copper(i) cation–π complex 15, described by (Δ*E*_OA_^‡^ − Δ*E*_Cu(III)_). Throughout our investigations, this value has been shown to give the closest correlation between computational and experimental results, as it takes into account the two determining factors for oxidative addition ability of complexes 14a–14g, as well as for aryl iodides 17a–17e. When considering the copper(i) systems coordinated with bipyridyl ligands, including correlations with experimental observations using a sulfinate substrate (see Experimental ESI Section 4.5.4., ES32–33†), it was revealed that the electron density on the copper is linked directly to Δ*E*_OA_, which is one of the driving forces for oxidative addition. Upon plotting NPA charge *versus* computed Δ*E*_OA_ value for these three bipyridyl ligands, an *R*^2^ value of 0.99 shows an excellent linear correlation between copper electron density and oxidative addition activation energy. Using the non-ligated copper species 14a as a benchmark for comparison, in the majority of cases ligation with electron-donating ligands results in a reduction of the oxidative addition activation energy, Δ*E*_OA_. As seen for ligands bpy 14b and 4,4′-diMeObpy 14c, coordination of electron-rich bipyridyls results in a lowering of Δ*E*_OA_ in the region of 5–8 kJ mol^−1^. It does appear, however, that introduction of electron-withdrawing groups such as in 14d (4,4′-diNO_2_bpy) leads to an increase in activation energy, correlating well with the previously calculated NPA charges of the copper(i) sulfinate species. These computational findings correlate with experimental results, where variation of the ligand within model reactions of sodium benzenesulfinate 1 and 4-iodotoluene 2 showed enhanced yields for 4,4′-diMeObpy over 4,4′-diNO_2_bpy (see Experimental ESI Section 4.5.4., ES32–33†).

For the non-bipyridyl based ligands 14e–g, reported in the literature to be highly effective for copper(i)-catalysed sulfinate cross couplings,^7–9^ the two parameters, Δ*E*_OA_ and Δ*E*_Cu(III)_, appear to play a significant role. For DMEDA 14e, the driving force for the lowering of the activation barrier for oxidative addition looks to be mainly driven by the increased electron density of the copper(i) centre, given the NPA charge of +0.453 (Fig. 7), the most electron-rich of any complex investigated here. As a result of this increased electron density, the calculated value of Δ*E*_OA_ is lowered to +46.9 kJ mol^−1^, and the relative stability of the copper(iii) species results in a much lower value for Δ*E*_OA_ − Δ*E*_Cu(III)_. The combination of these two factors leads to a more facile oxidative addition of the aryl iodide to copper when DMEDA is used in place of bpy, as reported by Baskin and Wang.^7^

The primary ligand precursor species reported by Zhu and Ma is the l-proline sodium salt,^8^ providing the ligand depicted in 14f. Given that the resulting complex with copper is formally ionic, this likely leads to the increase in Δ*E*_OA_ observed, with two species with high electron density interacting in the transition state. However, the highly donating nature of the anionic ligand to the copper leads to a significant stabilisation of the resulting copper(iii) species once the activation barrier is overcome, as indicated by the Δ*E*_Cu(III)_ value of −37.3 kJ mol^−1^. It is this stabilisation of the copper(iii) species which we propose leads to the utility of l-prolinate 14f as an effective ligand in copper(i)-catalysed sulfinate coupling processes,^8^ with a comparable Δ*E*_OA_ − Δ*E*_Cu(III)_ value to DMEDA.

The most recent generation of ligands for these coupling processes, based on l-hydroxyproline derivatives,^9^ includes DMPHPC (see 14g). Based on our computed results, such ligands have the potential to overcome the challenging oxidative addition process by both lowering the oxidative addition activation energy (Δ*E*_OA_) and providing concomitant stabilisation for the copper(iii) species (Δ*E*_Cu(III)_). Under the published reaction conditions, using K_3_PO_4_ as a base, it is unlikely that the amide N–H is deprotonated, despite this being proposed in the original publication.^9^ In contrast to the l-prolinate ligand, this would result in an oxidative addition to a copper(i) species which is formally neutral. Through our computational studies, geometry optimised structures for the transition state suggest a hydrogen-bonding stabilisation occurring between the bound sulfinate and the ligand's amide N–H, likely further lowering the Δ*E*_OA_, in addition to providing an electron-rich copper species. Furthermore, there also appears to be a significant stabilisation of the copper(iii) species resulting from the oxidative addition, with π–π interactions occurring between the copper-bound aryl ring and the aryl unit of the ligand in (DMPHPC)Cu(*p*-Tol)(I)(SO_2_Ph). A combination of the low oxidative addition activation energy (Δ*E*_OA_ = +37.9 kJ mol^−1^) and more effective stabilisation of the copper(iii) species (Δ*E*_Cu(III)_ = −18.6 kJ mol^−1^) than provided by bipyridyl ligands 14b and 14c results in a Δ*E*_OA_ − Δ*E*_Cu(III)_ of +19.3 kJ mol^−1^, more favourable than any bpy system, DMEDA, or l-prolinate. This hypothesis supports the observation that when using the DMPHPC ligand in cross-coupling reactions of sulfinates and aryl iodides, the transformation is possible at room temperature, with low catalyst loadings.^9^

One final area of the oxidative addition step of the process studied relates to the electronic requirements of the aryl iodide species for facile oxidative addition. Similarly to the previously conducted investigations with the migratory insertion step, the oxidative addition transition state searches were modified such that R groups were introduced onto the aryl iodide ring at the position *para* to the iodine (17a–17e). Using the bpy ligand, the overall energetic profile was constructed for each aryl iodide species, and respective Δ*E*_OA_ and Δ*E*_Cu(III)_ values were extracted for comparison, in addition to the difference, Δ*E*_OA_ − ΔE_Cu(III)_ (Fig. 8, right-hand table and Computational ESI Section 2.8, CS9†). Based on the Δ*E*_OA_ − Δ*E*_Cu(III)_ values for each species, it is suggested that electron-deficient aryl iodides (R = NO_2_17d, Δ*E*_OA_ − Δ*E*_Cu(III)_ = +33.4 kJ mol^−1^; and R = F 17e, Δ*E*_OA_ − Δ*E*_Cu(III)_ = +34.4 kJ mol^−1^) are more effectively tolerated than electron-rich aryl iodides (R = OMe 17c, Δ*E*_OA_ − Δ*E*_Cu(III)_ = +43.1 kJ mol^−1^), with the Cu^III^ species from the electron-deficient substrates also more thermodynamically stable than the electron-rich counterparts. The trends observed computationally for Δ*E*_OA_ − Δ*E*_Cu(III)_ are in agreement with reported electronic requirements of aryl halides for oxidative addition, with a more electron-deficient C–I bond desirable for more facile oxidative addition.^45^ These observations are further supported by the experimental results of Baskin and Wang, where a comparative reaction of 4-iodoanisole (R = OMe) 17c with sodium *p-*toluenesulfinate required 48 h to achieve a 75% yield of coupled product, whereas the reaction with iodobenzene (R = H) 17a only required 20 h to deliver a similar 70% yield.^7^

### Reductive elimination from copper(iii) to yield sulfone

**Fig. 9:**
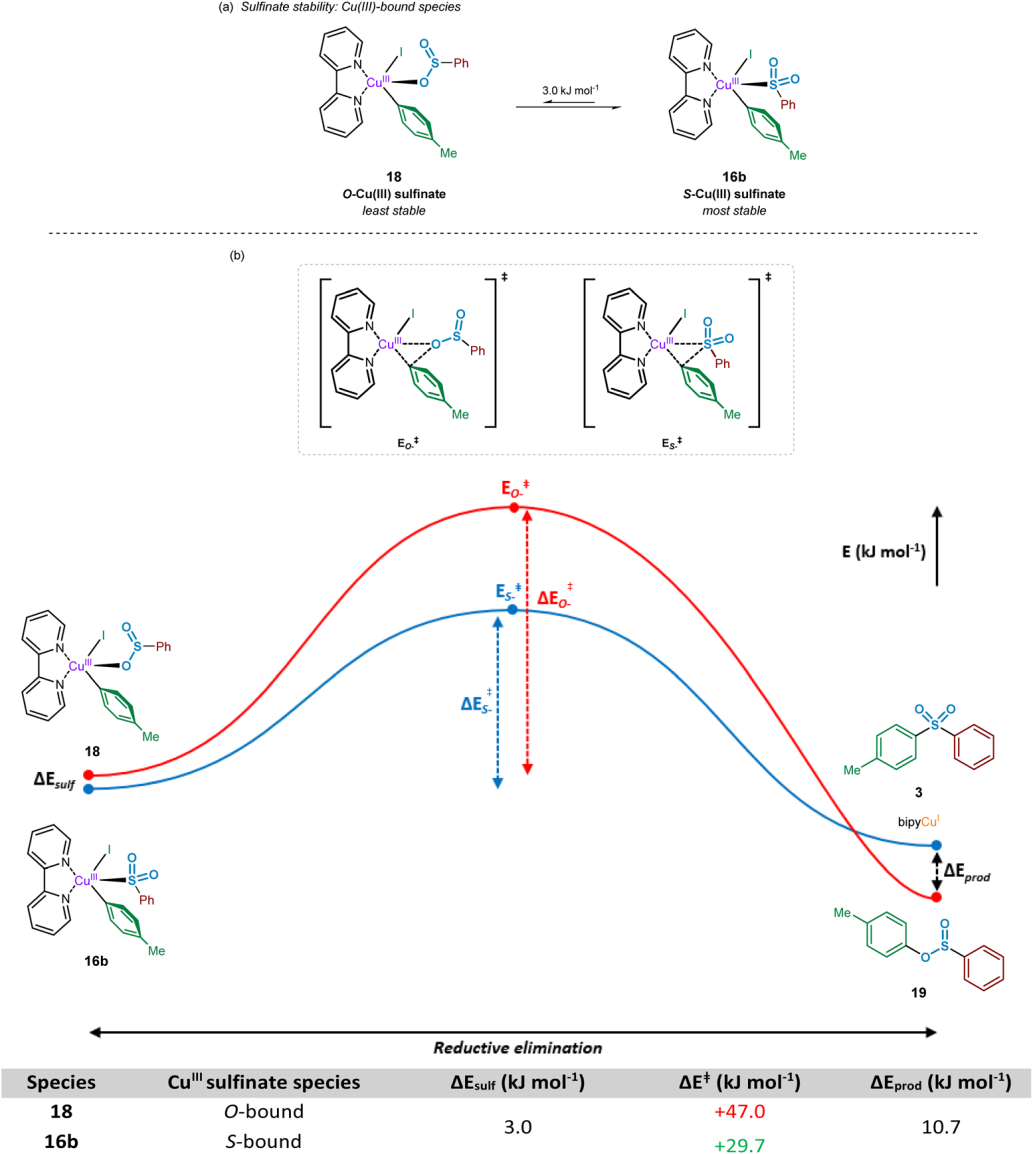
Computational investigations into the reductive elimination from a copper(iii) sulfinate species. (a) Relative stabilities of the oxygen and sulfur bound Cu(iii) sulfinate species thought to participate within the sulfonylative Suzuki–Miyaura reaction. (b) Comparative energy profile for reductive elimination of sulfone and sulfinic acid ester products from the corresponding *S*- and *O*-bound Cu(iii) sulfinate species.

Following the oxidative addition of the aryl iodide species to the copper(i)-bound sulfinate, a transient copper(iii) sulfinate species is formed. This species could exist as one of two regioisomers, arising from the known binding modes of sulfinates to metal species:^33^ through the oxygen atom, the *O*-sulfinate 18, or through the sulfur atom, the *S*-sulfinate 16b (Fig. 9).

Computational calculations suggest that for the mechanistically penultimate copper(iii) sulfinate species, the *S*-bound sulfinate (bpy)Cu(*p*-Tol)(I)(SO_2_Ph) 16b should be thermodynamically more stable than the *O*-bound sulfinate (bpy)Cu(*p*-Tol)(I)(OSOPh) 18, by approximately 3 kJ mol^−1^. The differences in energies of these species are shown in Fig. 9a. Given the small magnitude of this energy difference, a thermodynamic driving force is required for formation of the sulfone product 3 over the corresponding sulfinic acid ester 19. This is further supported by the observation that no sulfinic acid ester product has ever been reported from these Cu-catalysed sulfonylative cross-coupling reactions, suggesting reductive elimination from the *S-*bound copper(iii) sulfinate species, 16b, is favoured. There may, however, be a small possibility of a thermal rearrangement from the sulfinic acid ester to the sulfone, following reductive elimination from an *O*-bound Cu^III^ sulfinate species 18.

Using the geometry-optimised structures of the *O*-bound sulfinate, (bpy)Cu(*p*-Tol)(I)(OSOPh) 18, and the *S-*bound sulfinate, (bpy)Cu(*p*-Tol)(I)(SO_2_Ph) 16b, the reductive elimination was modelled computationally by gradual movement of the copper-bound heteroatom and the *ipso*-carbon of the phenyl group. This gave two reductive elimination energy profiles (Fig. 9b, Computational ESI Section 2.9, CS10†). The results obtained suggest why no sulfinic acid ester species 19 has been observed, with the activation barrier for such reductive elimination 1.5 times the magnitude (+47.0 kJ mol^−1^) of the similar process to yield the sulfone product (+29.7 kJ mol^−1^). Most importantly, the numerical difference between these two activation energies is appreciably greater than the difference in ground-state energies of the two Cu^III^ species 18 and 16b, leading to a more thermodynamically favourable process: rearrangement of an *O*-bound 18 to an *S-*bound sulfinate 16b, followed by reductive elimination (summed process +26.7 kJ mol^−1^, compared with the direct reductive elimination from the *O-*bound Cu^III^ sulfinate 18 of +47.0 kJ mol^−1^). It is also thought that the lower thermodynamic stability of species such as 19 under the reaction conditions contributes to the observation of only the sulfone product.^46^

**Fig. 10:**
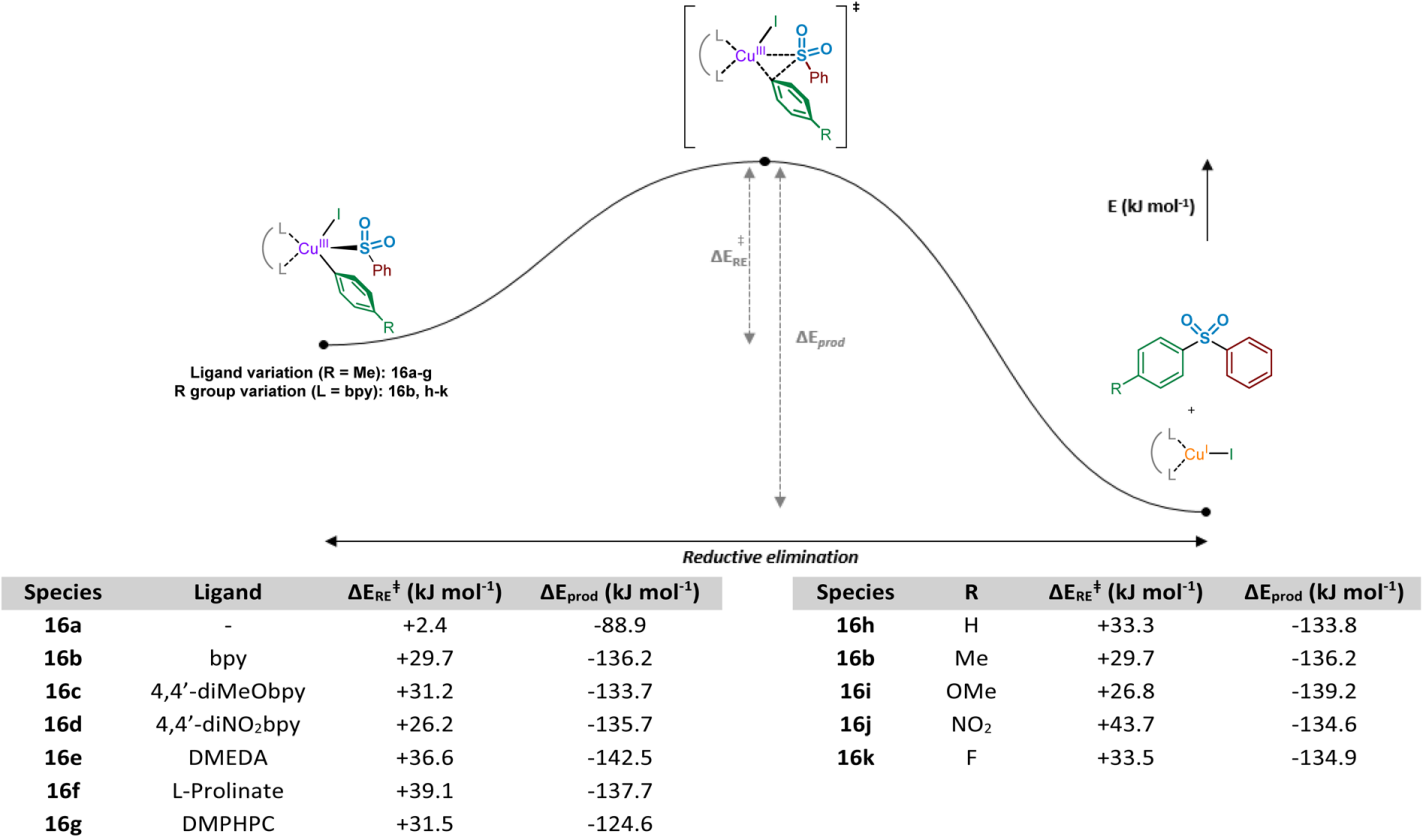
Computational investigations of the reductive elimination of biaryl sulfone product from a short-lived copper(iii) LCu(SO_2_Ph)(Ar)(I) complex. Computed energy profile for the reductive elimination process, exploring effects of ligand and aryl iodide R group on the activation energy and stability of the resultant sulfone product and Cu(i) species; left-hand table, ArI = 4-iodotoluene 2 employed; and right-hand table, L = bpy employed.

Following the computational evaluation of the pathway for reductive elimination through the *S-*bound copper(iii) sulfinate, the electronic requirements for facilitation of the reductive elimination were explored. Given the inherent instability of the copper(iii) species formed upon oxidative addition, the reductive elimination to yield the sulfone product 3 is expected to occur rapidly. The effect of electronic requirements for both ligand and aryl halide R group on the reductive elimination activation energy, Δ*E*_RE_, were calculated to produce the energy profile and values shown in Fig. 10 (see Computational ESI Sections 2.10 and 2.11, CS11–12†). The calculated energy between the reductive elimination transition state and the products, Δ*E*_prod_, is highly favourable (≥approx. −90 kJ mol^−1^) in all cases, allowing a conclusion that the reductive elimination step is almost certainly irreversible. Upon comparison of the ligandless complex 16a and bipyridyl-based ligands 16b–d, calculated Δ*E*_RE_ values for the reductive elimination to form the sulfone suggest that less electron-donating ligands are more favourable, in agreement with the expected reversal for the oxidative addition process (Fig. 8). The favouring of an electron-poor copper is further supported by the observation of a minimal +2.4 kJ mol^−1^ reductive elimination activation energy for the ligandless complex. For the literature-sourced ligands 16e–g,^7–9^ which are highly electron-rich, more elevated Δ*E*_RE_ values are likely linked to the significant increase in electron density on copper. However, given the generally reduced magnitude of the Δ*E*_RE_ values compared to activation energies for oxidative addition, Δ*E*_OA_ (Fig. 8), as aligned with well-documented difficult oxidative additions to copper(i) centres,^36^ these increased reductive elimination activation energies are not considered to be problematic.

As a direct comparison with the computed values for reductive elimination by Cantat *et al.*,^10^ from the high-energy copper(iii) species (DMEDA)Cu(SO_2_Ph)(Ph)(I), our calculated values are comparable. The reported value for the activation energy for reductive elimination from the *S-*bound copper(iii) sulfinate species is +36.0 kJ mol^−1^ compared to our +36.6 kJ mol^−1^. Also reported within the communication by Cantat *et al.*, although not identical to our studied system for the *O*- *versus S-*bound reductive elimination, is the significantly higher activation barrier for *O*-bound reductive elimination (Fig. 9).^10^ For reductive elimination from (DMEDA)Cu(OSOPh)(Ph)(I), a significantly higher reductive elimination barrier of +112.5 kJ mol^−1^ for the *O*-bound process is suggested than the *S*-bound value of +36.0 kJ mol^−1^.

Similar to the ligand trend reversal when compared with the oxidative addition activation energy, Δ*E*_OA_, calculated values for the reductive elimination reveal a preference for electron-rich aryl halide coupling partners. In a similar fashion to that reported for reductive elimination from palladium complexes,^47^ the presence of electron-deficient metal-bound groups is unfavourable (exemplified when R = NO_2_, 16j, Δ*E*_RE_ = +43.7 kJ mol^−1^), whereas the process is significantly more facile with electron-rich eliminating groups (see R = OMe, 16i, Δ*E*_RE_ = +26.8 kJ mol^−1^). Another contributing factor when R = NO_2_ may be resonance contributions (*cf.* the migratory insertion of SO_2_ section), which result in strengthening of the Cu–Ar bond and disfavouring of the bond-breaking process.

In order to provide further overarching insight, a comparison of activation energies for the key reaction steps (transmetalation, migratory insertion, oxidative addition, and reductive elimination) for the overall sulfonylative cross-coupling of phenylboronic acid, sulfur dioxide, and 4-iodotoluene, with bpy ligand, is provided within the Computational ESI Section 2.12, CS13.† These values suggest that either the transmetalation step (+81.0 kJ mol^−1^), or oxidative addition (+53.3 kJ mol^−1^), may be the rate-determining step in this process, although this is challenging to predict accurately due to the complexity of the overall catalytic cascade. In addition, a complete potential energy surface for this catalytic process is also included in the Computational ESI (Fig. S1, Section 2.13†), including the relative energies of all key reaction intermediates.

### Investigation of side-reactions

**Fig. 11:**
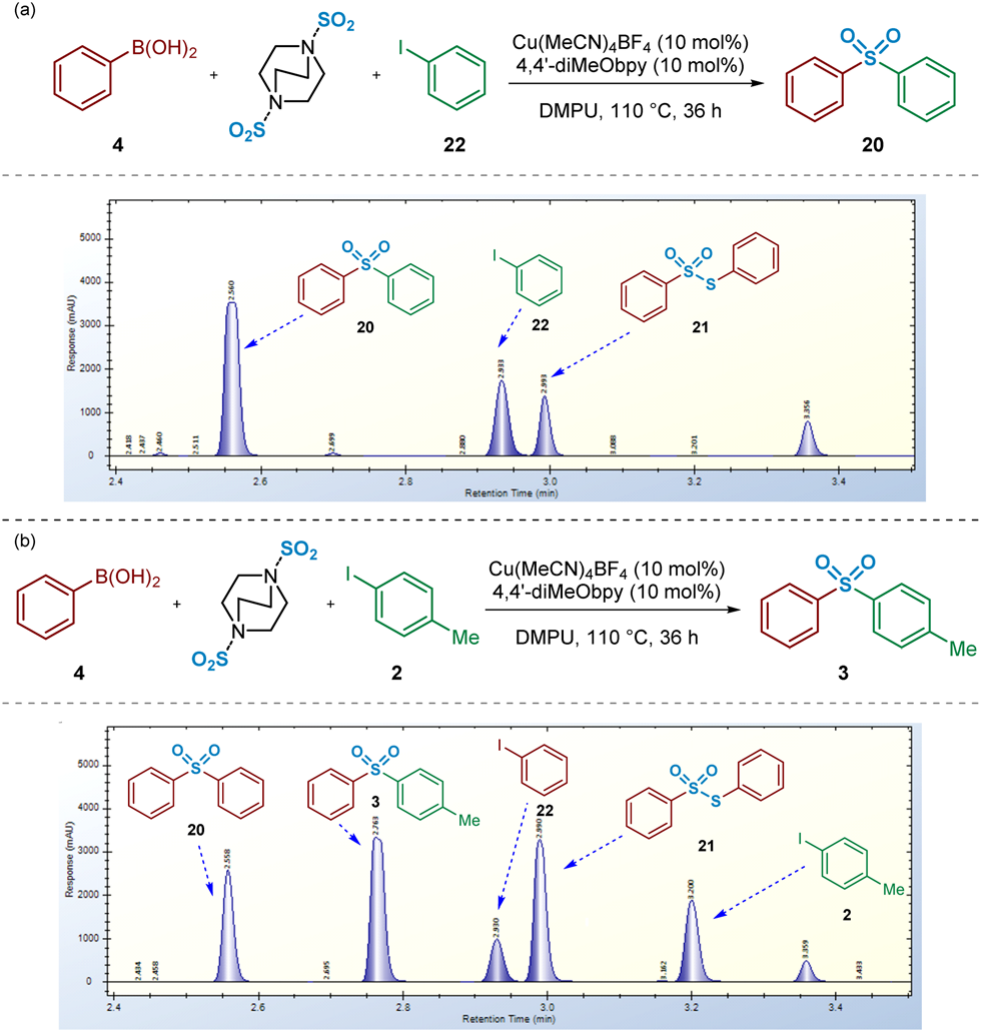
Crossover experiment investigations into the fates of reaction components by switching to an unsymmetrical targeted product. (a) HPLC trace obtained for the reaction of phenyboronic acid, iodobenzene, and DABSO under Cu(i) catalysis. (b) HPLC trace obtained for the reaction of phenylboronic acid, 4-iodotoluene, and DABSO under Cu(i) catalysis. Conditions for (a) and (b): phenylboronic acid (0.6 mmol), aryl iodide (0.2 mmol), DABSO (0.3 mmol), Cu(MeCN)_4_BF_4_ (0.02 mmol, 10 mol%), 4,4′-diMeObpy (0.02 mmol, 10 mol%), DMPU (1 mL), 110 °C, 36 h.

When initially investigating the Cu^I^-catalysed sulfonylative Suzuki–Miyaura reaction of boronic acids and aryl iodides in the presence of DABSO within this study, the symmetrical sulfonyldibenzene 20 was chosen as the test targeted product for simplicity. As detailed in Fig. 11, monitoring the reaction of phenylboronic acid, DABSO, and iodobenzene under the literature conditions^4^ resulted in formation of the desired product sulfonyldibenzene (*t*_ret_ = 2.56 min) in addition to another, initially, unknown by-product signified by the peak at *t*_ret_ = 2.99 min. This side-product was, in turn, identified as *S*-phenyl benzenethiosulfonate 21 through comparison with a commercial reference.

As previously discussed, a benzenesulfinate species (*e.g.*1) is formed as an intermediate in these coupling processes; as this species accumulates, an alternative disproportionation side-reaction becomes viable. More specifically, arylthiosulfonates, such as 21, are known to form alongside arylsulfonic acids as a result of the disproportionation of 3 equivalents of the arylsulfinic acid,^48^*i.e.* the protonated form of reaction intermediate 1. This disproportionation process is known to occur readily in the presence of trace amounts of acid, which are likely to arise from the dissolution of SO_2_(g) in residual water found in the hygroscopic solvent. Reaction of commercial sodium benzenesulfinate 1 under the reaction conditions (in the absence of DABSO) resulted in reduced amounts of the thiosulfonate. Based on comparison of HPLC yields, when SO_2_ is present in the sulfonylative Suzuki–Miyaura process, a ratio of sulfone:thiosulfonate of approx. 6 : 1 is observed, whilst use of the sulfinate intermediate (with no SO_2_ additive) provides a ratio of 123 : 1. These observations suggest that DABSO is at least contributing to this side-reaction (see Experimental ESI Sections 4.6.1.2 and 4.6.1.3., ES36–38†).

Karl-Fischer titrations revealed commercially obtained DMPU contained approximately 0.06% H_2_O by weight, which equates to a relatively large excess compared with the amount of forming benzenesulfinate. Despite the hygroscopic nature of DMPU, the use of drier solvent, as anticipated, resulted in diminished yields of the thiosulfonate by-product 21, increasing the product:by-product ratio from 5.6 : 1 to 8 : 1 (see Experimental ESI Section 4.6.1.2., ES36–38†).

Upon switching to an unsymmetrical system, using the coupling of phenylboronic acid 4 and 4-iodotoluene 2, HPLC spectra revealed a larger number of unexpected species forming in the reaction mixture, in addition to the desired (*p*-tolyl)sulfonylbenzene 3 (Fig. 11b). Furthermore, the unsymmetrical nature of this coupling system supports the identification of the origin of each reaction component. Related to this, it appeared that, in addition to the side-reaction involving disproportionation of the intermediate benzenesulfinate to *S-*phenyl benzenethiosulfonate 21, the boronic acid or intermediate sulfinate component was also leading to formation of both symmetrical sulfone 20 and iodobenzene 22. Indeed, these additional side products may explain the diminished yields, compared to those published,^4^ that have been obtained in our laboratories when attempting to synthesise unsymmetrical sulfone products (*e.g.*Fig. 11b).

**Scheme 3:**
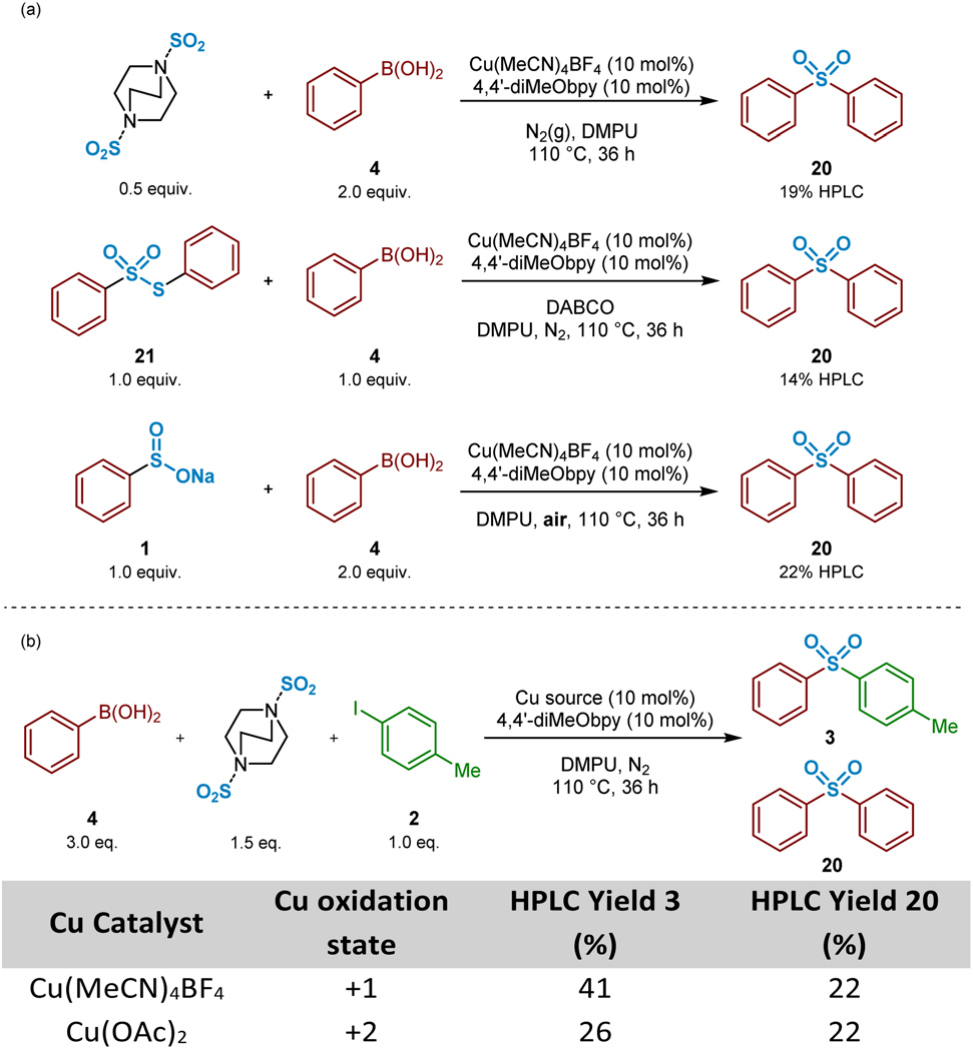
Control experiments investigating symmetrical sulfone formation when targeting unsymmetrical sulfone synthesis. (a) Preliminary control experiments identifying the benzenesulfinate intermediate and phenylboronic acid starting material as the source of symmetrical sulfone formation. (b) HPLC yields of unsymmetrical and symmetrical sulfone products when varying the copper oxidation state. Yields were determined by HPLC (*λ* = 235 nm) using dimethyl terephthalate as an internal standard.

Based on that observed and as detailed in Fig. 11b, a number of control experiments to investigate the formation of symmetrical sulfone 20 were then considered. As described above (Fig. 3), the test reaction of the intermediate sodium benzenesulfinate 1 with 4-iodotoluene 2 allowed us to identify that the symmetrical sulfone product formation did not arise directly from a homocoupling of the sulfinate intermediate, *via* a desulfinylative-style coupling process; in the reaction shown in Fig. 3b, no symmetrical sulfonyldibenzene 20 was observed by HPLC, with only the desired unsymmetrical product 3 identified. This observation supported the view that the phenylboronic acid component 4 was, at least, somewhat involved in the pathway leading to this side-reaction product formation. To test this hypothesis, as described in Scheme 3, a 4 : 1 mixture of phenylboronic acid 4 and DABSO (1.0 eq. SO_2_(g)) were mixed under the reaction conditions, and resulted in formation of 19% of the symmetrical sulfone 20 by HPLC, suggesting that the observed reactivity results from either a sulfonylative boronic acid homocoupling, or a copper-catalysed reaction of the intermediate sulfinate species 1 with a remaining equivalent of arylboronic acid (Scheme 3a, entry 1).

In the presence of organic base, thiosulfonates are known to revert back to their corresponding sulfinates.^49,50^ Based on this, it could, therefore, be that the build-up of *S-*phenyl benzenethiosulfonate 21, arising from the primary disproportionation reaction, is contributing to formation of the undesired symmetrical sulfone product 20. Exposure of a 1 : 1 mixture of commercial *S-*phenyl benzenethiosulfonate 21 and phenylboronic acid 4 under catalysis conditions in the presence of DABCO, the carrier base of the sulfur dioxide in DABSO, resulted in formation of the symmetrical sulfone 20 in 14% HPLC yield (Scheme 3a, entry 2). These combined observations have led to a conclusion that this side-reactivity is arising from a copper-catalysed cross-coupling of the benzenesulfinate intermediate with an equivalent of phenylboronic acid, partly promoted by the large excess (3 : 1 ratio) of arylboronic acid to aryl iodide present within the standard reaction mixture, as generally employed by Willis.^4^

A test reaction using 2 : 1 phenylboronic acid and sodium benzenesulfinate with Cu(MeCN)_4_BF_4_, under aerobic conditions, provided the symmetrical sulfone product 20 in a 22% HPLC yield, suggesting oxidation of Cu^I^ to Cu^II^*in situ* (Scheme 3a, entry 3). Based on this, and upon researching the potential of this suggested pathway, a publication from Huang and Batey supported our emerging propositions: arylboronic acids can efficiently react with aryl sulfinates under oxidative Cu^II^ catalysis within Chan–Evans–Lam-type sulfinate couplings.^51^ It was surprising, however, to observe how effectively this side-reaction was occurring under the Willis conditions, especially given the reactions were being carried out under N_2_(g): the Cu^I^-catalysed reaction of phenylboronic acid 4, 4-iodotoluene 2, and DABSO under inert conditions yielded an identical 22% HPLC yield of the unexpected symmetrical sulfone product (Scheme 3b, Table entry 1). Similarly, colleagues elsewhere in our laboratories investigating the utility of this copper(i)-catalysed transformation for medicinal applications also observed formation of a symmetrical sulfone product when carried out in a glovebox.^52^ These observations led to a suggestion that a reaction component is facilitating the conversion of Cu^I^ to Cu^II^, prior to a coupling similar to that reported by Huang and Batey.^51^ A parallel reaction, in which Cu(MeCN)_4_BF_4_ was substituted with Cu(OAc)_2_ additionally resulted in 26% of the unsymmetrical sulfone 3, and again 22% HPLC of the symmetrical sulfone 20 (Scheme 3b, Table entry 2), suggesting a Cu^I^ species, able to catalyse the cross-coupling of arylsulfinates and aryl halides to yield unsymmetrical sulfones, is a by-product of the Cu^II^ cycle.

Test reactions carried out with extensive degassing of solvent (freeze–pump–thaw method) resulted in little decrease in the occurrence of the side products, highlighting that the oxidation process was not occurring from residual oxygen trapped within the solvent (Experimental ESI, Section 4.6.3.8., ES47†). An additional mechanistic control experiment with the Willis reaction performed under air resulted in significantly reduced yields of both sulfone products, ruling out an aerobic oxidation process, and further supporting the proposal of *in situ* oxidation of Cu^I^ to Cu^II^ facilitated by a reaction component (Experimental ESI, Section 4.6.3.7., ES46†). These observations, in combination with literature precedence of high rates of Cu^I^ disproportionation to Cu^0^ and Cu^II^ in dipolar aprotic solvents,^53^ supports a disproportionation proposition to facilitate formation of copper(ii) within the reaction manifold. A further experimental observation supporting the Cu^I^ to Cu^0^ and Cu^II^ disproportionation was the common deposition of metallic copper on stirrer bars used throughout these studies.

**Fig. 12:**
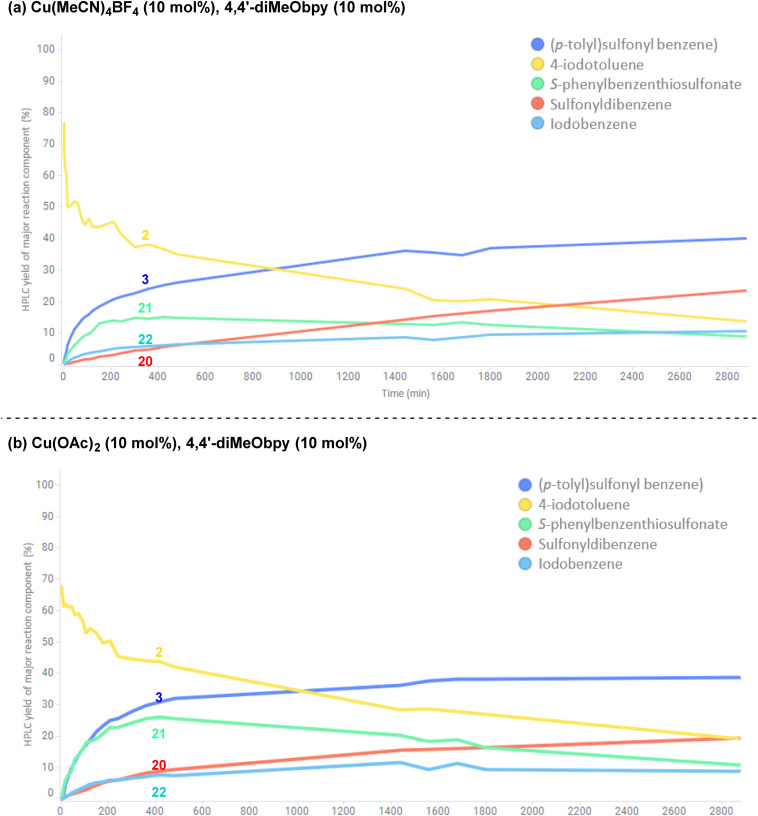
Comparative reaction time courses of two separate sulfonylative Suzuki–Miyaura reactions for the formation of (*p*-tolyl)sulfonylbenzene. (a) Time course of reaction catalysed by the Cu(i) salt, Cu(MeCN)_4_BF_4_. (b) Time course of reaction catalysed by the Cu(ii) salt, Cu(OAc)_2_. Both reactions were performed using the substrates and reagents: phenylboronic acid (0.6 mmol), 4-iodotoluene (0.2 mmol), DABSO (0.3 mmol), copper source (0.02 mmol, 10 mol%), 4,4′-diMeObpy (0.02 mmol, 10 mol%), dimethyl terephthalate (internal standard, 0.04 mmol), DMPU (1 mL), N_2_, 110 °C, 48 h. Yields quantified for each major reaction component by HPLC and by comparison of % area with the internal standard.

To investigate the potential copper(ii)-catalysed transformation further, we opted to track both a Cu^I^ and Cu^II^-catalysed reaction in a 48 h time course, for comparison of relative rates of reaction and formation of by-products (copper(i): Fig. 12a; copper(ii): Fig. 12b; full data tables are provided in the Experimental ESI Sections 4.6.3.10. and 4.6.3.11, ES48–51†). Surprisingly, both graphical representations of the time courses (Fig. 12) show a very close resemblance, suggesting both that the reaction profile when employing either a Cu^I^ or Cu^II^ salt is similar, and that there is a continuous exchange between Cu^I^ and Cu^II^ species throughout the reaction time period. Closer analysis of the data points within the reaction profile resulting from use of the Cu^I^-species reveals that unsymmetrical sulfone 3 product formation is occurring from the first timepoint after *t* = 0 h, whereas there is a 5 minute lag period in the reaction profile for the same sulfone product 3 where the Cu^II^-salt was employed, indicating the potential time requirement for generation of the Cu^I^ species from Cu^II^. The opposite is seen for the Cu^II^-generated product 20 in the process employing the Cu^I^-species, again with an observed 5 minute delay period.

In both reaction profiles, the HPLC yield of the disproportionation product, *S-*phenyl benzenethiosulfonate 21 is observed to reach a peak after approximately 6 h, followed by a slow decrease across the remainder of the reaction profile; this can be attributed to the previously discussed equilibrium with the benzenesulfinate species in the presence of DABCO, presumably contributing to formation of small quantities of the symmetrical sulfone product 20. The formation of small quantities of iodobenzene 22 in the reaction mixture is thought to arise from an oxidative protodeboronation-halogenation process,^54^ including reaction with small amounts of iodide liberated over time in the reaction mixture by solvation of (4,4′-diMeObipy)Cu–I, a product of the final reductive elimination.

### Proposed overall copper(i)/(ii)/(iii) catalytic cycles

**Fig. 13:**
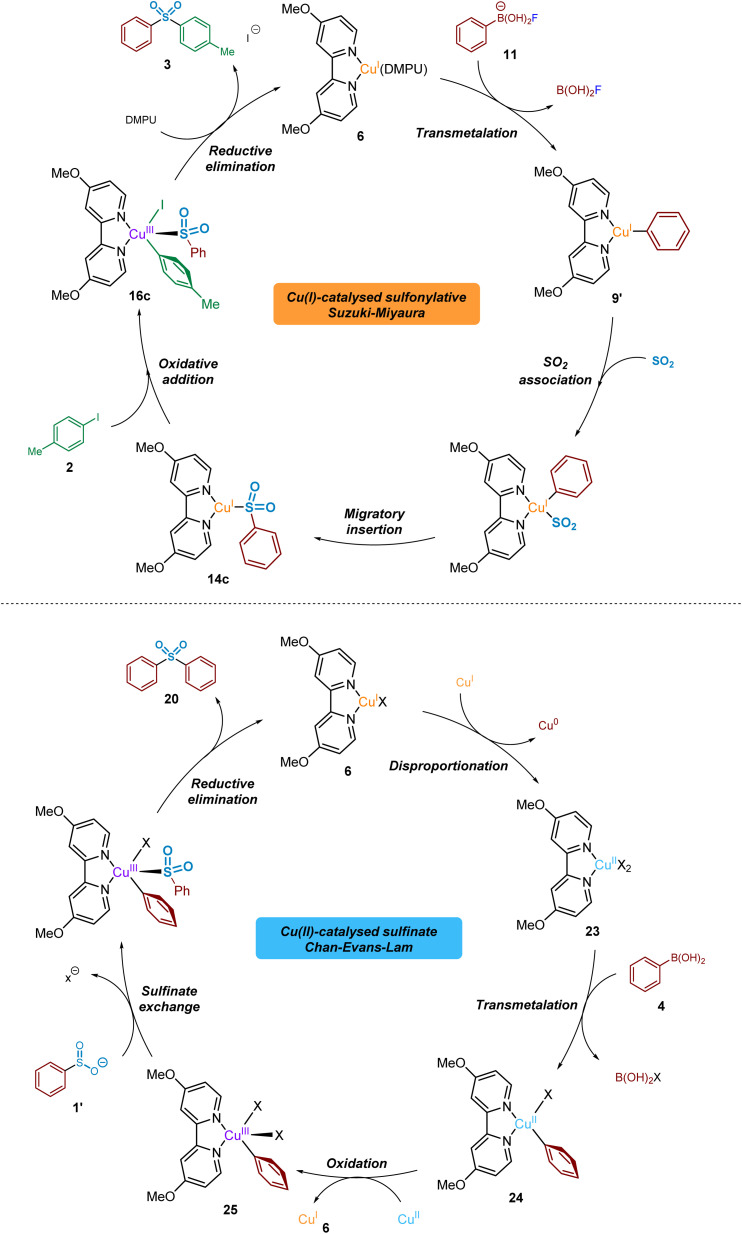
Proposed catalytic cycles based on experimental and computational observations. (a) Catalytic cycle for the Cu(i)-catalysed sulfonylative Suzuki–Miyaura reaction of arylboronic acids, sulfur dioxide and aryl iodides. (b) Cu(ii)-based Chan–Evans–Lam side reaction of the intermediate arylsulfinate with arylboronic acid starting material, yielding symmetrical sulfone products.

From the data generated and described herein, we can propose a series of interchanging Cu^I^/Cu^II^/Cu^III^-based catalytic processes, where intermediates and by-products from each are capable of participating in competing cycles (Fig. 13).

From the LCMS-based investigations of the Cu^I^-catalysed process, a resting state 16-valence electron catalytic species such as (4,4′-diMeObpy)Cu^I^(DMPU) 6 is proposed. This species is thought to participate as the catalytic component in a cycle that delivers both the sulfonylative conversion of arylboronic acid to arylsulfinate, and the subsequent production of biarylsulfone in the presence of an aryl iodide.

The BF_4_^−^ (tetrafluoroborate) counterion of the copper(i) catalyst, Cu(MeCN)_4_BF_4_, is proposed to undergo a hydrolysis-like mechanism with the carrier base of DABSO, DABCO, leading to the liberation of a catalytic amount of fluoride. The presence of a DABCO-BF_3_ adduct 10 has been detected by both ^11^B and ^19^F NMR (see Fig. 5), supporting our proposal of this pathway. The liberated fluoride then participates in the subsequent Lewis-base activation of the arylboronic acid, *via* reactive arylfluoroboronate species 11, facilitating transmetalation of the aryl group onto copper(i) to yield (4,4′-diMeObpy)Cu^I^(Ar) 9′ (and B(OH)_2_F), a fluorinated variant 9 of the copper(i) aryl species having been detected by ^19^F NMR. The metastable B(OH)_2_F species is thought to exist in equilibrium between B(OH)_3_, B(OH)_*x*_F_*y*_ species, and BF_4_^−^ throughout the reaction process. Computational studies have supported the order of transmetalation before the binding of SO_2_ to the copper(i) centre, further confirmed by the observation of the Cu^I^-aryl species by ^19^F NMR in the absence of sulfur dioxide.

Following transmetalation, binding of sulfur dioxide to the copper(i) centre is thought to occur, with computational calculations suggesting this is through the sulfur atom (Computational ESI Section 2.2., CS3–5†).^29^ Fluorine-19 NMR experiments using 4-fluorophenylboronic acid 8 in the presence of DABSO, in comparison to without SO_2_(g), showed no formation of the non-sulfinylated intermediate copper(i)-aryl species 9; this indicates the binding of SO_2_(g) to copper(i), and the subsequent migratory insertion to form the sulfinate species is extremely rapid.

This migratory insertion step is thought to be favoured by the presence of electron-withdrawing ligands (*e.g.* 4,4′-diNO_2_bpy), and by use of electron-rich boronic acid starting substrates (*e.g.* 4-iodoanisole). The generated sulfinate intermediate 1′ has been identified by LCMS, HRMS, and NMR studies, in addition to isolation as a sodium salt.

The previously discussed metal-bound nature of the sulfinate species through the subsequent oxidative addition step is supported by a number of publications covering copper(i)-catalysed carbon-heteroatom bond-forming transformations.^42,55^ In order to generate the desired sulfone product, oxidative addition of the copper(i) sulfinate into the carbon–iodine bond occurs to form a transient Cu^III^ species 16c. Calculations also predict that this oxidative addition step is promoted by the use of ligands which are both electron-donating, and also stabilise significantly the resulting copper(iii) species *via* intramolecular interactions, such as π–π stacking between the copper-aryl group and the ligand (*e.g.* DMPHPC).

Computational methods have revealed that the most stable binding mode of the sulfinate to the resulting copper(iii) species is through the sulfur atom, in an *S-*bound fashion. This preference, in turn, leads to a rapid reductive elimination of the bound aryl and sulfinate groups to furnish the sulfone product 3 in preference to the sulfinic acid ester 19. Calculations also predict that this reductive elimination process, to yield the biarylsulfone, is favoured by the use of less electron-donating ligands (*e.g.* 4,4′-diNO_2_bpy) and copper-aryl species containing electron-rich aryl rings (*e.g.* 4-iodoanisole).

Throughout our mechanistic investigations, discovery of a second competing catalytic cycle, involving Cu^I^, Cu^II^, and Cu^III^ species in a Chan–Evans–Lam coupling, results in formation of undesired symmetrical sulfone products such as 20, when only formation of unsymmetrical products should be otherwise occurring. Starting from the resting state (4,4′-diMeObpy)Cu^I^ species 6 an *in situ* disproportionation process to (4,4′-diMeO bpy)Cu^II^23 and Cu^0^, thought to be facilitated the dipolar aprotic solvent DMPU,^53^ begins the catalytic process. Similar to the copper(i) process, transmetalation of the arylboronic acid species occurs, producing the copper(ii)-aryl species (4,4′-diMeObpy)Cu^II^(Ph)X 24. Another equivalent of the Cu^II^ species 23 is able to act as an oxidant,^56^ resulting in the formation of a (4,4′-diMeObpy)Cu^III^(Ph)X_2_ complex 25, as widely proposed in the mechanism of such Chan–Evans–Lam cross-coupling processes.^19,57^ The copper(i) species 6 generated can then enter the desired sulfonylative Suzuki–Miyaura cycle. Experimental results from a time course study using Cu(OAc)_2_ suggest a delay before the Cu^I^ cycle begins; generation of this Cu^I^ species by reduction in the Cu^II^ cycle explains this observation. Lastly, in the Chan–Evans–Lam cycle the intermediate sulfinate species, generated from the copper(i) catalytic cycle, intercepts the (4,4′-diMeObpy)Cu^III^(Ph)X_2_ intermediate 25, and a subsequent reductive elimination forms a symmetrical sulfone product 20 and the resting state catalytic species 6.

Based on our experimental observations, these two catalytic processes are directly involved with each other, with catalytic species and other components from the Cu^I^ process able to engage in the Chan–Evans–Lam-cycle, and Cu^I^ intermediates generated in the latter Cu^II^ cycle able to participate in the sulfonylative Suzuki–Miyaura process. As part of this, it is believed that the use of excess phenylboronic acid (3.0 eq), leading to accumulation of an arylsulfinate intermediate, contributes to the occurrence of the Cu^II^-catalysed Chan–Evans–Lam process and the undesired by-products observed when conducting the methodology targeted at delivering sulfonylative Suzuki–Miyaura coupling.

## Conclusions

The utility of novel methods for synthesis of sulfones and additional S^VI^ moieties is of paramount importance in many sectors, including the pharmaceutical industry. Our search for new and applicable catalytic sulfonylation methods led us to conduct a detailed mechanistic study of the sulfonylative Suzuki–Miyaura reaction, using the SO_2_(g) surrogate DABSO.

Throughout our investigations we have identified resting-state and reactive Cu^I^ species, in addition to an arylsulfinate intermediate generated from the arylboronic acid starting material. In a combined experimental and computational study, the factors influencing the reaction, including effects of ligand and coupling partners, were explored. Individual key reaction steps, including transmetalation of arylboronic acid onto copper(i), insertion of sulfur dioxide to generate a sulfinate species, oxidative addition of copper(i) into the C–I bond of the aryl halide coupling partner, and the reductive elimination process have been studied. Importantly, these investigations further gave rise to the discovery of the formation of undesired (symmetrical) sulfone (and other) by-products upon attempted sulfonylative Suzuki–Miyaura processes to deliver (unsymmetrical) sulfones. This, in turn, has led to a proposed Cu^I^/Cu^II^/Cu^III^ catalytic sulfonylative Chan–Evans–Lam process, that is directly connected with the Suzuki–Miyaura catalytic cycle, and *via* which the symmetrical sulfone by-products are believed to result.

In summary, a detailed mechanistic investigation, supported by computational methods and identification of key reactive intermediates, has delivered a more thorough understanding of a complex catalytic cascade as applied to the preparation of medicinally relevant sulfone products. Utility of density functional theory methods to provide theoretical insights into previously unexplored reaction steps of importance, including migratory insertion of copper(i)–sulfur dioxide complexes, oxidative addition of aryl halides to copper(i)-bound sulfinates, and the subsequent reductive elimination, have the potential to provide the foundations for the design and utility of an *in-silico* predictive tool for challenging copper(i)-catalysed sulfonylative cross-couplings. The implications of this study, especially as relating to establishing copper-catalysed sulfonylative processes of enhanced selectivity and utility, coupled with the prevention of unproductive by-product formation, and as aligned with our efforts towards developing applicable processes for use within pharmaceutically-aligned medicinal chemistry programmes, are currently the subject of on-going investigations within our laboratories.

## Data availability

All experimental procedures, characterisation, and computational data for this study can be found in the ESI files.†

## Author contributions

CGJH: data curation; formal analysis; investigation; methodology; software; writing – original draft. HFS: conceptualisation; project administration; resources; supervision; writing – review & editing. PP: formal analysis; methodology; software. DML: conceptualisation; supervision. WJK: conceptualisation; funding acquisition; project administration; supervision; writing – review & editing.

## Conflicts of interest

The authors have no conflicts to declare.

## Supplementary Material

SC-014-D3SC01337E-s001

SC-014-D3SC01337E-s002
