# Supplementary material for: Experimental and computational insights into the mechanism of the copper(i)-catalysed sulfonylative Suzuki–Miyaura reaction
Source: Chem Sci. 2023 May 31;14(24):6738–55. doi: 10.1039/d3sc01337e (PMC10284122; doi:10.1039/d3sc01337e)
Supplement: SC-014-D3SC01337E-s001 [file SC-014-D3SC01337E-s001.pdf]

Electronic Supplementary Material (ESI) for Chemical Science.
This journal is © The Royal Society of Chemistry 2023

Experimental and computational insights into the mechanism of the copper(I)-catalysed sulfonylative Suzuki-Miyaura reaction

Callum G. J. Hall,[a,b] Helen F. Sneddon,*[a,†] Peter Pogány,[a] David M. Lindsay,[b] and William J. Kerr*[b]

[a] Medicines Design, GlaxoSmithKline, Gunnels Wood Road, Stevenage, SG1 2NY, England, UK

[b] Department of Pure and Applied Chemistry, University of Strathclyde, 295 Cathedral Street, Glasgow, G1 1XL, Scotland, UK

[†] Present address: Green Chemistry Centre of Excellence, Department of Chemistry, University of York, York, YO10 5DD, England, UK

Computational Supporting Information

Contents

1. General computational methods 2

2. Key computational findings for the copper(I)-catalysed sulfonylative Suzuki-Miyaura reaction 2

2.1. Energy profile construction: transmetalation 2
2.2. Migratory insertion: Natural population analysis (NPA) & bond length analysis of ligated arylcopper(I) sulfur dioxide complexes 3
2.2.1. Ligand: 2,2'-Bipyridine, variation of aryl R group 4
2.2.2. Aryl: phenyl, variation of 4,4'-disubstituted-2,2'-bipyridine ligands 4
2.3. Migratory insertion energy profile construction: Variation of $\Delta E_{MI}^{‡}$ with aryl R group 5
2.4. Migratory insertion energy profile construction: variation of $\Delta E_{MI}^{‡}$ with complex ligand 5
2.5. Calculation of ^{19}F NMR shielding constants for copper-bound fluorinated sulfinate species 6
2.6. Oxidative addition: natural population analysis (NPA) of resting state copper(I) complexes 7
2.7. Oxidative addition energy profile construction: Variation of $\Delta E_{OA}^{‡}$ with ligand 7
2.8. Oxidative addition energy profile construction: Variation of $\Delta E_{OA}^{‡}$ with aryl iodide R group 9
2.9. Reductive elimination energy profile construction: *O*- versus *S*-bound sulfinate reductive elimination 10
2.10. Reductive elimination energy profile construction: Variation of $\Delta E_{RE}^{‡}$ with ligand 11
2.11. Reductive elimination energy profile construction: Variation of $\Delta E_{RE}^{‡}$ with aryl R group 11
2.12. Tabulated activation energies for key mechanistic steps in the copper(I)-catalysed sulfonylative Suzuki-Miyaura reaction of phenylboronic acid, sulfur dioxide, and 4-iodotoluene, using L=bpy 13

3. Computed atomic coordinates, energies, and vibrational frequencies for optimised ground state and transition state species 15

3.1. Transmetalation of phenylboronic acid onto copper(I) 15
3.1.1. Ground state geometry optimisations 15
3.1.2. Transition state optimisations 18
3.2. Insertion of SO_2 into the copper–carbon bond 19
3.2.1. SO_2 insertion into the copper–carbon bond: variation of $\Delta E_{MI}^{‡}$ with aryl R group 19
3.2.2. SO_2 insertion into the copper–carbon bond: variation of $\Delta E_{MI}^{‡}$ with complex ligand 26
3.3. Oxidative addition of copper(I) into the copper–carbon bond 33
3.3.1. Oxidative addition of 4-iodotoluene to (L)Cu(SO_2Ph): Variation of $\Delta E_{OA}^{‡}$ with complex ligand 33
3.4. Oxidative addition to (bpy)Cu(SO_2Ph): Variation of $\Delta E_{OA}^{‡}$ aryl iodide 4-position substituent 59
3.4.1. Geometry optimisations of common species to all pathways 59
3.5. Reductive elimination from copper(III) 73
3.5.1. Reductive elimination pathways *via* an *O*-bound or *S*-bound copper(III) sulfinate 73
3.5.2. Reductive elimination of (*p*-tolyl)sulfonylbenzene from (L)Cu(Tol)(SO_2Ph)(I): Variation of $\Delta E_{RE}^{‡}$ with complex ligand 78
3.5.3. Reductive elimination of biaryl sulfones from (bpy)Cu(Ar)(SO_2Ph)(I): variation of $\Delta E_{RE}^{‡}$ with aryl R group 89

4. References 97

1. General computational methods

All computational Density Functional Theory (DFT) calculations were performed using a combination of Schrödinger (Maestro)[1] and the Gaussian 16[2] series of programs, built into a linux-based supercomputing system. GaussView 5.0.9 was used for visualisation of structures, vibrational frequencies, predicted NMR spectra, NPA atomic charges, and energies.

Within Maestro, molecular structures were imported through the two-dimensional sketch tool, and from this a conformational search in three-dimensional space was carried out using the 'conformational search' macromodel keyword[3] (default settings). The computed three-dimensional conformers were then exported as .xyz files containing atomic coordinates for input into Gaussian .gjf files.

Within Gaussian, computations were carried out using the B3LYP density functional,[4,5] and the 6-31G+(d,p)[6,7] basis set level of theory for lighter atoms. Alternatively, the SDD (ECP46MWB pseudopotential)[8] basis set was used to model heavier atoms in order to account for the relativistic effects of electrons (e.g. iodine); when this was used, the 'B3LYP/gen pseudo=read' keyword was used instead of the usual 'B3LYP/6-31+G(d,p)' keyword. The 'EmpiricalDispersion=GD3' keyword was used to account for dispersion and non-bonding interactions between neighbouring atoms.[9] The 'Integral(Grid=Ultrafine)' keyword was used for improved computational accuracy when modelling diffuse functions. Additionally, all species were modelled using the 'SCRF=(solvent=*N*,*N*-Dimethylformamide)' keyword,[10] applying similar solvation conditions to those expected when using *N*,*N'*-dimethylpropylene urea (DMPU) as the solvent under experimental conditions; currently a SCRF model for DMPU is not available. Natural population analyses (NPA atomic charges and molecular orbital populations) were performed using the 'pop=nbo' keyword.[11] Energies in atomic units (a.u.) were converted into kJ mol^{-1} by multiplication by the conversion factor of 2625.499.

For geometry optimisations and ground state energy calculations, the keywords 'opt' and 'freq' were used for geometry optimisations and frequency calculations, respectively. The lowest energy conformers containing only positive vibrational frequencies were used as the ground state structure.

For calculation of NMR shielding constants, the keyword 'nmr=gaio' was used, with a higher basis set of aug-cc-PVTZ used in place of 6-31+G(d,p) to ensure a higher accuracy of calculations. These calculations were carried out as a single-point-energy calculation.

For transition state searches, each energy maxima was found on the potential energy surface through use of the scan function within the 'opt=modredundant' keyword. The selected atoms were moved towards or away from each other sequentially in a number of steps by a set distance. Within each scan step, a geometry optimisation was carried out. A frequency calculation of the energy-maxima structures generated from the transition state search was subsequently carried out using the 'opt(TS,calcfc,noeigentest)' keyword, and the presence of a single intense negative vibrational frequency confirmed the structure was a valid transition state.

2. Key computational findings for the copper(I)-catalysed sulfonylative Suzuki-Miyaura reaction

2.1. Energy profile construction: transmetalation

Using the above calculated energies for geometry-optimised starting materials and the optimised transition state found from the transition state search, it is possible to construct an energy profile for the transmetalation step between phenylboronic acid and (2,2'-bipyridyl)copper(I), *via* the intermediate phenylfluoroboronate. An energy profile for the transmetalation step, including tabulated energy values, are shown in Table S1.

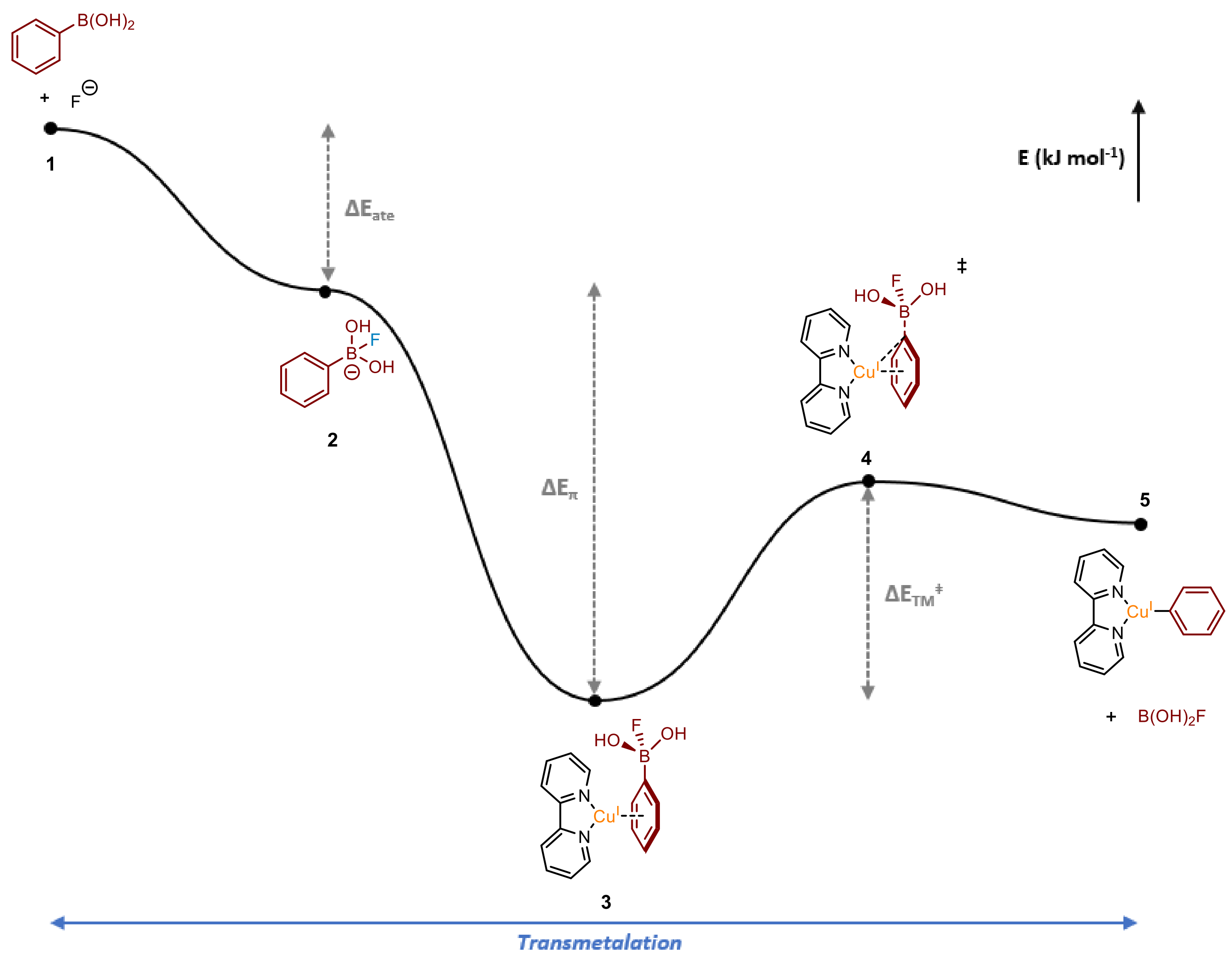

Species	1	Δate	2	Δπ	3
**E (a.u.)**	-508.31782422	-0.02288199	-508.34070621	-0.05695451	-2644.07375476
**Δ (kJ mol⁻¹)**		**-60.0**		**-149.5**	

Species	$\Delta_{TM}^{‡}$	4	Δ_{prod}	5
**E (a.u.)**	0.03083615	-2644.04291861	-0.00509778	-2644.04801639
**Δ (kJ mol⁻¹)**	**+81.0**		**-13.4**	

Table S1. Energy values, in atomic units (a.u., Hartrees) and kJ mol^{-1} for individual species and energy barriers involved in the transmetalation of phenylboronic acid onto a copper(I) bipyridyl species.

2.2. Migratory insertion: Natural population analysis (NPA) & bond length analysis of ligated arylcopper(I) sulfur dioxide complexes

Following previous reports suggesting the preference for SO_2-insertion into electron-rich metal–carbon bonds,[12] and the poor performance of electron-deficient groups, the R group on the *para*-position of the aryl ring was modified. The geometry-optimised species were then used in the calculation of atomic charges based on NPA, in particular the *ipso*-carbon (migrating carbon). Calculated bond lengths and computed NPA charges are shown for variation of 4,4'-disubstituted-2,2'-bipyridine ligands (Table S3) and variation of aryl R group (Table S2).

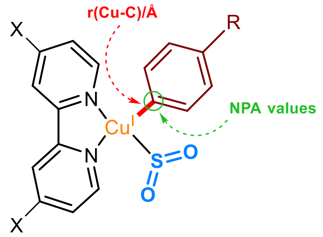

2.2.1. Ligand: 2,2'-Bipyridine, variation of aryl R group

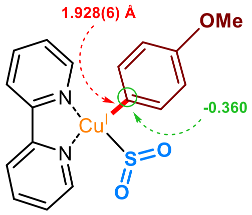

12a (R = OMe)

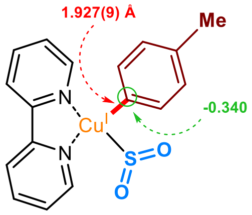

12b (R = Me)

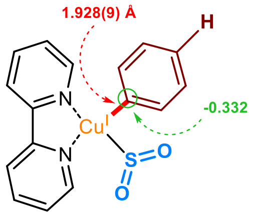

12c (R = H)

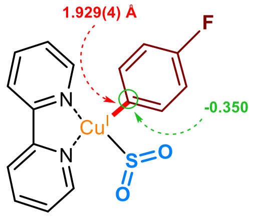

12d (R = F)

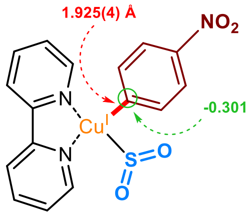

12e (R = NO_2)

Species	R group	r(Cu–C)/Å	NPA (*ipso*- C)
**12a**	OMe	1.928(6)	-0.360
**12b**	Me	1.927(9)	-0.340
**12c**	H	1.928(9)	-0.332
**12d**	F	1.929(4)	-0.350
**12e**	NO_2	1.925(4)	-0.301

Table S2. Calculated values for the length of the Cu–C bond length, r(Cu–C)/Å, and the electron density on the *ipso*- carbon to copper, NPA(*ipso*- C) upon variation of the R group at the 4-position on the aryl ring of (bipy)Cu^{I}(Ar)(SO_2).

2.2.2. Aryl: phenyl, variation of 4,4'-disubstituted-2,2'-bipyridine ligands

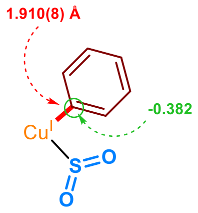

13a (No L)

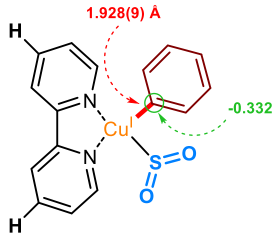

12c (L = bpy)

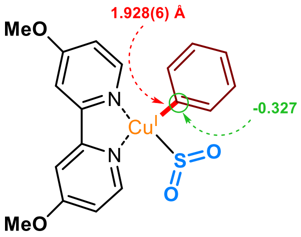

13b (L = diMeObpy)

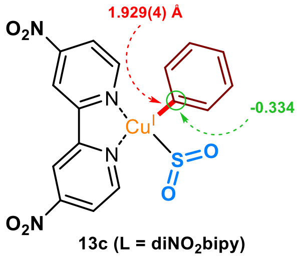

13c (L = diNO_2bipy)

Species	Ligand	r(Cu–C)/Å	NPA (*ipso*- C)
**13a**	-	1.910(8)	-0.382

**12c**	Bpy	1.928(9)	-0.332
**13b**	4,4'-diMeObpy	1.928(6)	-0.327
**13c**	4,4'-diNO$_2$bpy	1.929(4)	-0.334

Table S3. Calculated values for the length of the Cu–C bond length, r(Cu–C)/Å, and the electron density on the ipso- carbon to copper, NPA(ipso- C) upon variation of the ligand in (L)Cu^{I}(Ph)(SO$_2$).

2.3. Migratory insertion energy profile construction: Variation of $\Delta E_{MI}^{‡}$ with aryl R group

From the computed energy values for the corresponding arylcopper(I) sulfur dioxide complexes ($E_{bipyCu(Ar)(SO2)}$) and the related transition state energy ($E_{MI}^{‡}$), the activation barrier for the migratory insertion step ($\Delta E_{MI}^{‡}$) can be calculated. A graphical representation of these energies, in addition to energy values for individual species (a.u.) and energy changes (a.u. and kJ mol^{-1}), are tabulated in Table S4.

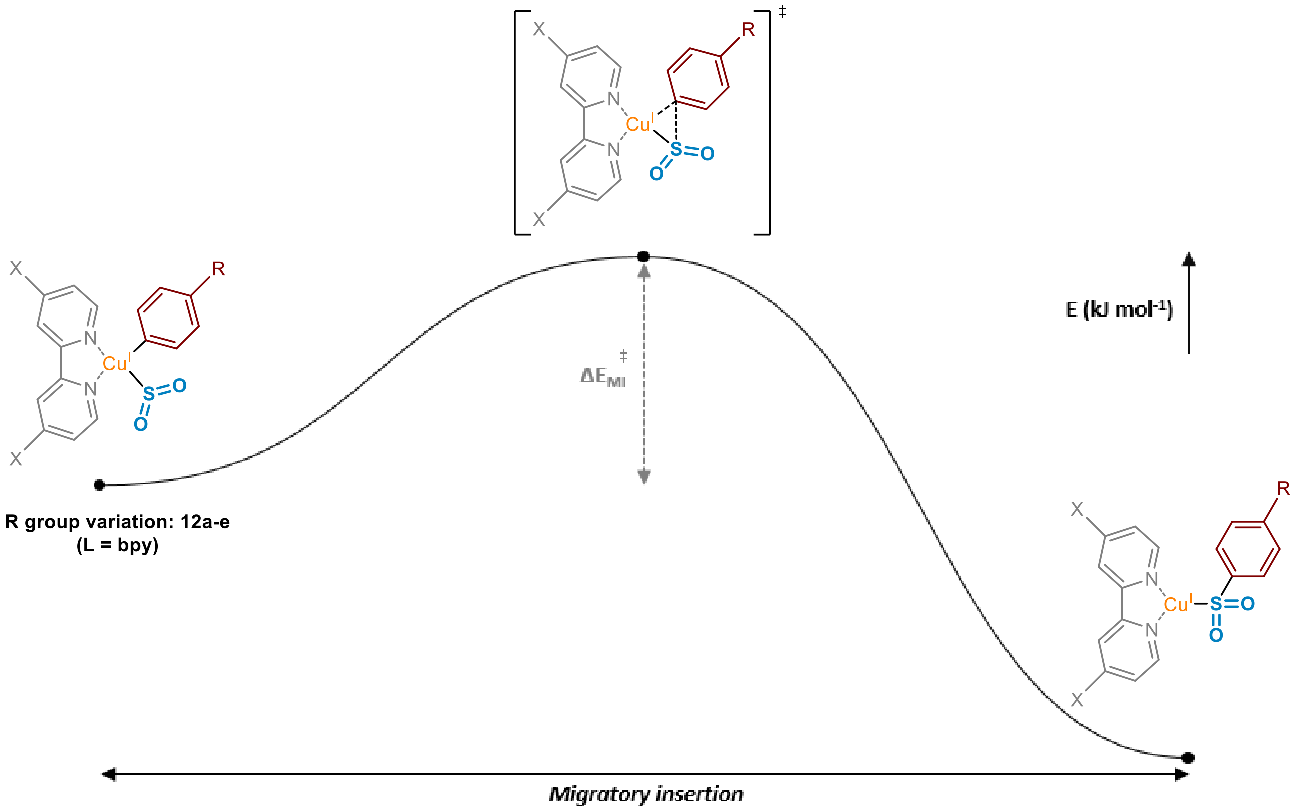

R group	$E_{bpyCu(Ar)(SO2)}$	$\Delta E_{MI}^{‡}$ (a.u.)	$\Delta E_{MI}^{‡}$ (kJ mol^{-1})	$E_{MI}^{‡}$
OMe (**12a**)	-3030.67374712	+0.00874845	**+23.0**	-3030.66499867
Me (**12b**)	-2955.46237133	+0.00981564	**+25.8**	-2955.45255569
H (**12c**)	-2916.13931626	+0.01090862	**+28.6**	-2916.12840764
F (**12d**)	-3015.38193959	+0.01119828	**+29.4**	-3015.37074131
NO$_2$ (**12e**)	-3120.66229754	+0.01529518	**+40.2**	-3120.64700236

Table S4. Calculated energy profile for the migratory insertion various the copper(I)-bound aryl groups into sulfur dioxide, in the complex (bpy)Cu(SO$_2$)(Ar). Energy change values represent the following: $\Delta E_{MI}^{‡}$, the migratory insertion activation energy.

2.4. Migratory insertion energy profile construction: variation of $\Delta E_{MI}^{‡}$ with complex ligand

From the computed energy values for the corresponding phenylcopper(I) sulfur dioxide complexes and the related transition state energy, the activation barrier for the migratory insertion step can be calculated. values for the migratory insertion activation energy, $\Delta E_{MI}^{‡}$, are tabulated in Table S5.

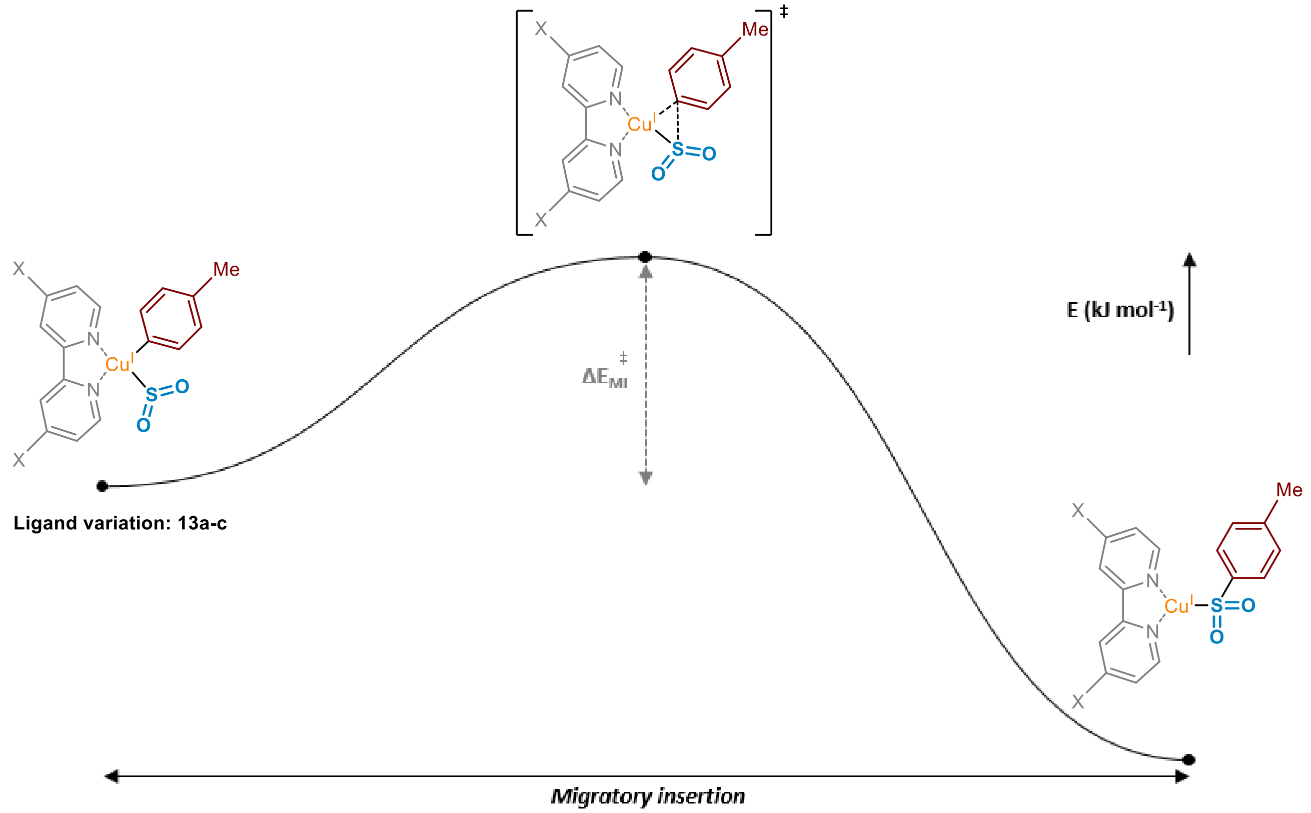

Ligand	$E_{LCu(Ph)(SO2)}$	$\Delta E_{MI}^{‡}$ (a.u.)	$\Delta E_{MI}^{‡}$ (kJ mol^{-1})	$E_{MI}^{‡}$
None (**13a**)	-2420.63226902	+0.01573791	**+41.3**	-2420.61653111
bpy (**12c**)	-2916.13931626	+0.01090862	**+28.6**	-2916.12840764
4,4'-diMeObpy (**13b**)	-3145.21971560	+0.01170143	**+30.7**	-3145.20801417
4,4'-diNO_2bpy (**13c**)	-3325.15005759	+0.00881989	**+23.2**	-3325.14123770

Table S5. Calculated energy profile for the migratory insertion of the copper(I)-bound phenyl group into sulfur dioxide, varying dependent on ligand present in the complex. Energy change values (ΔE) represent the following: $\Delta E_{MI}^{‡}$, the migratory insertion activation energy.

2.5. Calculation of ^{19}F NMR shielding constants for copper-bound fluorinated sulfinate species

Calculation of ^{19}F NMR shielding constants for *O*- and *S*-bound (4,4'-diMeObpy)Cu^{I} sulfinate species was carried out for prediction of relative chemical shifts to identify species observed experimentally. Relative energies of the two species were also calculated, as different quantitative ratios of each species were observed experimentally by NMR.

**Species**	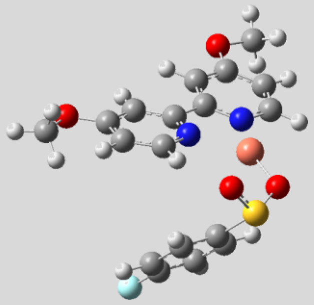 **(4,4'-diMeObpy)Cu^{I}(OSO-*p*-C_6H_5F)**	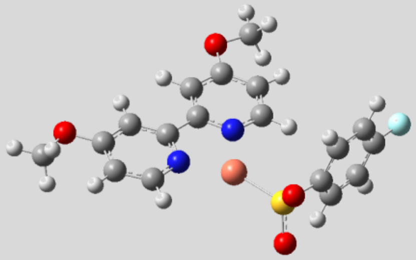 **(4,4'-diMeObpy)Cu^{I}(OSO-*p*-C_6H_5F)**
E(B3LYP) (a.u.)	-3245.14902535	-3245.15217001
ΔE (kJ mol^{-1})	8.3	
Magnetic shielding (ppm)	296.469	295.641

Table S6. Calculation of relative energies and ^{19}F NMR shielding constants for copper(I) sulfinate species present within the reaction mixture when using L=4,4'-diMeObpy and 4-fluorobenzenesulfinate.

Calculations predict that for the fluorinated sulfinate species, the *S*-bound sulfinate is more stable by 8.3 kJ mol^{-1}. The *S*-bound sulfinate is also predicted to have a smaller shielding value, which would result in a less negative ^{19}F NMR shift. These predictions are in agreement with experimental results, with the less shielded resonance (δ -113.9 ppm) in the ^{19}F spectrum shown in experimental supporting information section 4.4.3. is the most abundant in the mixture. Likewise, the more shielded resonance (δ -115.5 ppm) is less abundant, and can be assigned as *O*-bound sulfinate species.

2.6. Oxidative addition: natural population analysis (NPA) of resting state copper(I) complexes

It is widely known within the literature that, due to the high activation barriers for oxidative addition of copper(I) into a metal-halide bond, an electron-rich metal centre is required.[13] This often involves employing electron-donating ligands. The resting state copper(I) species which is thought to undergo oxidative addition, LCu, was analysed using NPA (Natural Population Analysis) to determine i) the donor abilities of the nitrogen-containing ligands (relative values on the nitrogen atoms, N^1 = left, N^2 = right) and ii) the relative electron density present on the copper atom in the corresponding complexes. The resulting values for the copper(I) ion and complexes thereof with the ligands bpy, 4,4'-diMeObpy, 4,4'-diNO$_2$bpy, DMEDA, L-prolinate, and DMPHPC, are shown below and tabulated for comparison in Table S7. Less positive (more negative) values imply increased electron density.

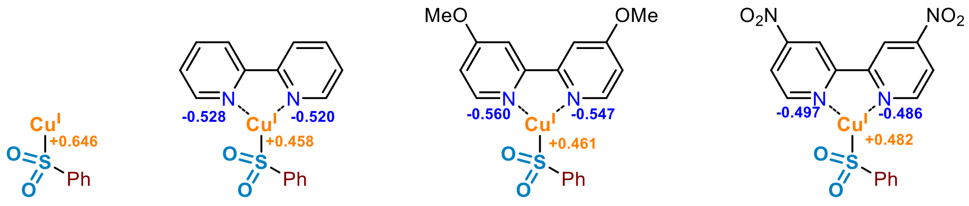

14a No Ligand | **14b** bpy | **14c** 4,4'-diMeObpy | **14d** 4,4'-diNO$_2$bpy

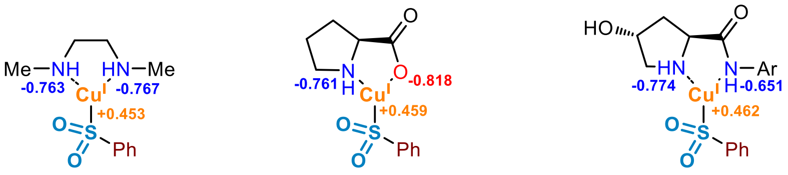

14e DMEDA Baskin *et al.*, 2002 | **14f** L-Prolinate Ma *et al.*, 2005 | **14g** DMPHPC Ma *et al.*, 2018

Species	Ligand	NPA (Cu^I)	NPA (N^1)	NPA (N^2/O^2)
**14a**	-	+0.646	-	-
**14b**	bpy	+0.458	-0.528	-0.520
**14c**	4,4'-diMeObpy	+0.461	-0.560	-0.547
**14d**	4,4'-diNO$_2$bpy	+0.482	-0.497	-0.486
**14e**	DMEDA	+0.453	-0.763	-0.767
**14f**	L-Prolinate	+0.459	-0.761	-0.818
**14g**	DMPHPC	+0.462	-0.774	-0.651

Table S7. Calculated NPA charges for the copper and ligating atoms in complexes of the form LCu^I(SO$_2$Ph).

2.7. Oxidative addition energy profile construction: Variation of $\Delta E_{OA}^{‡}$ with ligand

Using the above calculated (geometry-optimised) energy values of each (ligand)copper(I) benzenesulfinate complex, (ligand)copper(I) benzenesulfinate–4-iodotoluene cation–π complex, oxidative addition transition states, and (ligand)tolyl(*S*-sulfinylphenyl)copper(III) iodide species, energy values for the π-complexation energy (ΔE_π), oxidative addition activation energy ($\Delta E_{OA}^{‡}$) and transition state-copper(III) energy difference

($\Delta E_{Cu(III)}$) can be calculated. A graphical representation of these energies, in addition to energy values for individual species (a.u.) and energy changes (a.u. and kJ mol^{-1}), are tabulated in Table S8.

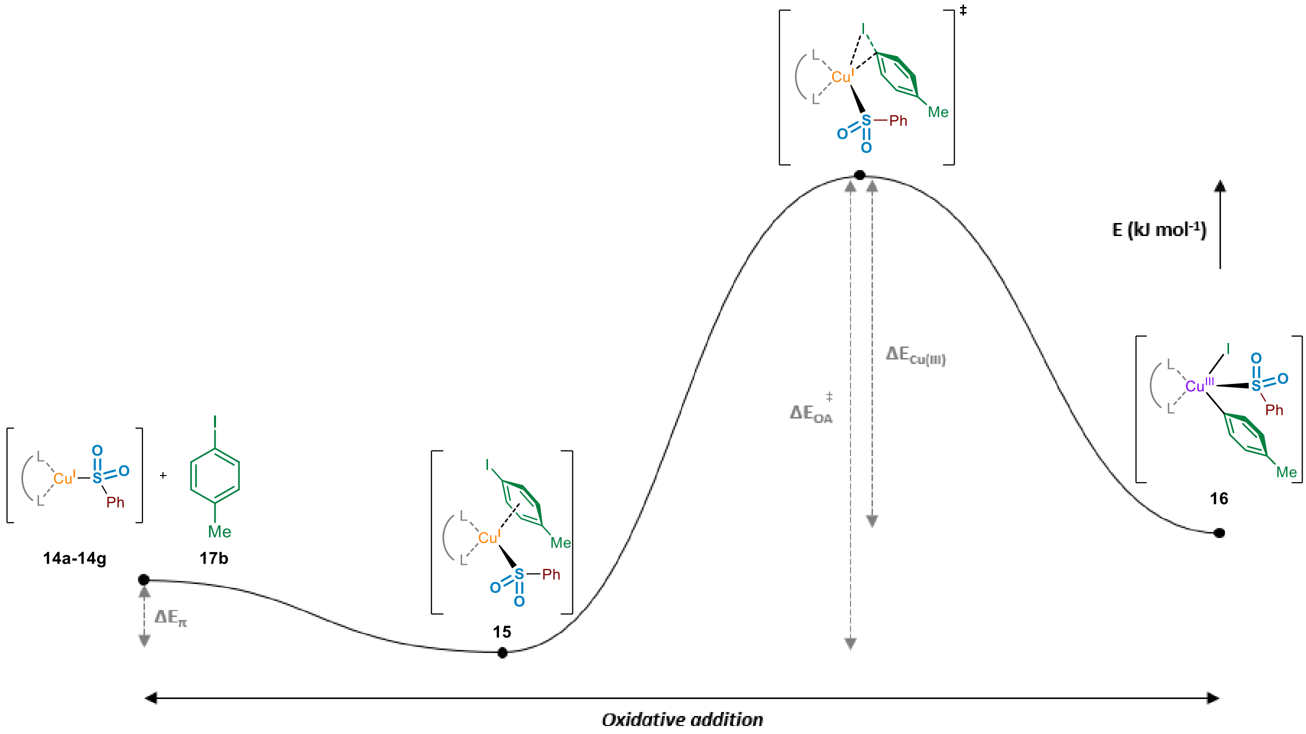

Ligand	E_{SM}	ΔE_{π} (a.u.)	ΔE_{π} (kJ mol^{-1})	E_{π}	ΔE_{OA} (a.u.)
**- (14a)**	-2703.05034876	-0.03151166	**-82.7**	-2703.08186042	+0.02333097
**bpy (14b)**	-3198.56638645	-0.00289440	**-7.6**	-3198.56928085	+0.02029517
**4,4'-diMeObpy (14c)**	-3427.64632431	-0.00156610	**-4.1**	-3427.64789041	+0.02128872
**4,4'-diNO₂bpy (14d)**	-3607.57844607	+0.00086153	**+2.3**	-3607.57758454	+0.02474837
**DMEDA (14e)**	-2972.31582493	-0.00205047	**-5.4**	-2972.31787540	+0.01786207
**L-prolinate (14f)**	-3103.88408594	-0.00146570	**-3.8**	-3103.88555164	+0.02433911
**DMPHPC (14g)**	-3469.42110553	+0.00453259	**+11.9**	-3469.41657294	+0.01442690

Ligand	ΔE_{OA} (kJ mol^{-1})	E_{OA}‡	$\Delta E_{Cu(III)}$ (a.u.)	$\Delta E_{Cu(III)}$ (kJ mol^{-1})	$E_{Cu(III)}$
**- (14a)**	**+61.3**	-2703.05852945	-0.00706275	**-18.5**	-2703.06559220
**bpy (14b)**	**+53.3**	-3198.54898568	-0.00487607	**-12.8**	-3198.55386175
**4,4'-diMeObpy (14c)**	**+55.9**	-3427.62660169	-0.00491506	**-12.9**	-3427.63151675
**4,4'-diNO₂bpy (14d)**	**+65.0**	-3607.55283617	-0.00727439	**-19.1**	-3607.56011056
**DMEDA (14e)**	**+46.9**	-2972.300013	-0.00824538	**-21.6**	-2972.30825871
**L-prolinate (14f)**	**+63.9**	-3103.86121253	-0.01422571	**-37.3**	-3103.87543824
**DMPHPC (14g)**	**+37.9**	-3469.40214604	-0.00709247	**-18.6**	-3469.40923851

Ligand	$\Delta E_{OA} - \Delta E_{Cu(III)}$ (kJ mol^{-1})
**- (14a)**	**+42.8**
**bpy (14b)**	**+40.5**
**4,4'-diMeObpy (14c)**	**+43.0**
**4,4'-diNO₂bpy (14d)**	**+39.0**
**DMEDA (14e)**	**+25.3**
**Lprolinate (14f)**	**+26.6**
**DMPHPC (14g)**	**+19.3**

Table S8. Calculated energy values for species of the form $LCu^{I}(SO_2Ph)$, $LCu^{I}(SO_2Ph)$-4-iodotoluene cation-π complex, the oxidative addition transition state, and the resulting species, $LCu^{III}(Ph)(SO_2Ph)(I)$. Energy change values (ΔE) represent the following: ΔE_{π}, the π complexation energy; ΔE_{OA}‡, the activation energy for oxidative addition; $\Delta E_{Cu(III)}$, the energy difference between transition state & copper(III) oxidative addition product.

2.8. Oxidative addition energy profile construction: Variation of $\Delta E_{OA}^{\ddagger}$ with aryl iodide R group

Using the above calculated (geometry-optimised) energy values of the (bpy)copper(I) complex, (bpy)copper(I)–aryl iodide cation–π complex, (bpy)copper(I)–aryl iodide oxidative addition transition state, and (bpy)arylcopper(III) iodide species, energy values for the π-complexation energy (ΔE_{π}), oxidative addition activation energy ($\Delta E_{OA}^{\ddagger}$) and transition state-copper(III) energy difference ($\Delta E_{Cu(III)}$) can be calculated. A graphical representation of these energies, in addition to energy values for individual species (a.u.) and energy changes (a.u. and kJ mol^{-1}), are tabulated in Table S9.

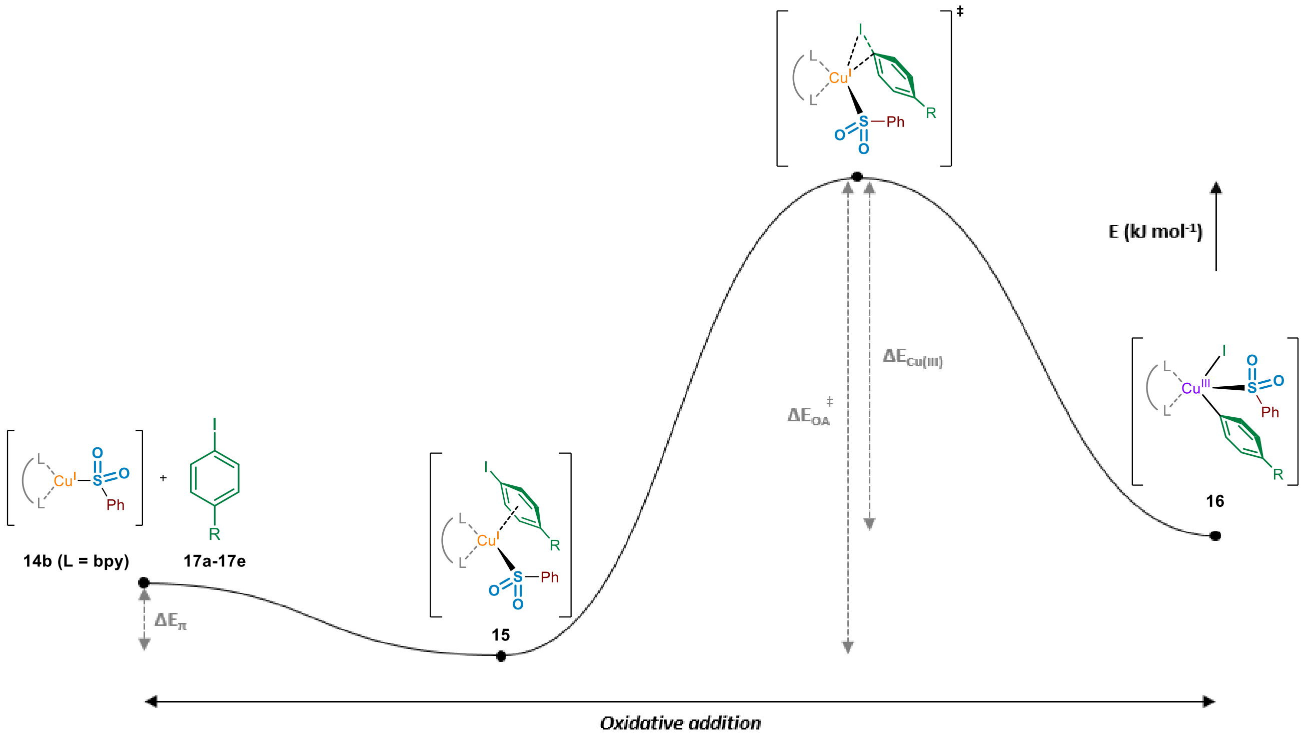

R group	E_{SM}	ΔE_{π} (a.u.)	ΔE_{π} (kJ mol^{-1})	E_{π}	ΔE_{OA} (a.u.)
**H (17a)**	-3159.24318038	-0.00118180	**-3.1**	-3159.24436218	+0.01935236
**Me (17b)**	-3198.56638645	-0.00289502	**-7.6**	-3198.56928085	+0.02029517
**OMe (17c)**	-3273.77663452	-0.00376969	**-9.9**	-3273.78040421	+0.02077655
**NO_2 (17d)**	-3363.75608597	-0.00397340	**-10.4**	-3363.76005937	+0.02174597
**F (17e)**	-3258.48286384	-0.00167485	**-4.4**	-3258.48453869	+0.02009320

R group	ΔE_{OA} (kJ mol^{-1})	$E_{OA}^{\ddagger}$	$\Delta E_{Cu(III)}$ (a.u.)	$\Delta E_{Cu(III)}$ (kJ mol^{-1})	$E_{Cu(III)}$
**H (17a)**	**+50.8**	-3159.22500982	-0.00627058	**-16.5**	-3159.23128040
**Me (17b)**	**+53.3**	-3198.54898568	-0.00487607	**-12.8**	-3198.55386175
**OMe (17c)**	**+54.5**	-3273.75962766	-0.00434715	**-11.4**	-3273.76397481
**NO_2 (17d)**	**+57.1**	-3363.73831340	-0.00903361	**-23.7**	-3363.74734701
**F (17e)**	**+52.8**	-3258.46444549	-0.00700827	**-18.4**	-3258.47145376

R group	$\Delta E_{OA} - \Delta E_{Cu(III)}$ (kJ mol^{-1})
**H (17a)**	**+34.3**
**Me (17b)**	**+40.5**
**OMe (17c)**	**+43.1**
**NO_2 (17d)**	**+33.4**
**F (17e)**	**+34.4**

Table S9. Calculated energy barriers between species of the form LCu^{I}, LCu^{I}-aryl iodide cation-π complex, the oxidative addition transition state, and the resulting species, LCu^{I}(Ar)(I). Energy change values (ΔE) represent the following: ΔE_{π}, the π complexation energy; $\Delta E_{OA}^{\ddagger}$, the activation energy for oxidative addition; $\Delta E_{Cu(III)}$, the energy difference between transition state & copper(III) oxidative addition product.

2.9. Reductive elimination energy profile construction: *O*- versus *S*-bound sulfinate reductive elimination

Using the above calculated (geometry-optimised) energy values of each copper(III) sulfinate species, copper(III) sulfinate reductive elimination transition state, and resulting products, energy values for the activation energy ($\Delta E_{RE}^{\ddagger}$), the energy difference between transition state and products (ΔE_{prod}), can be calculated. A graphical representation of these energies, in addition to energy values for individual species (a.u.) and energy changes (a.u. and kJ mol^{-1}), are tabulated in Table S10.

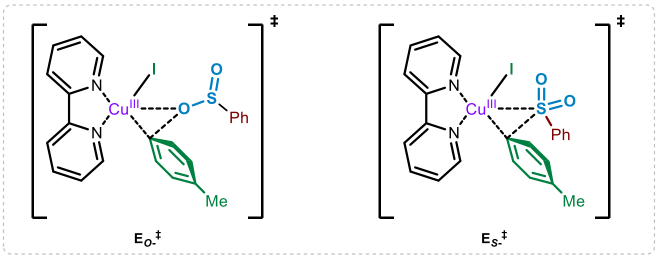

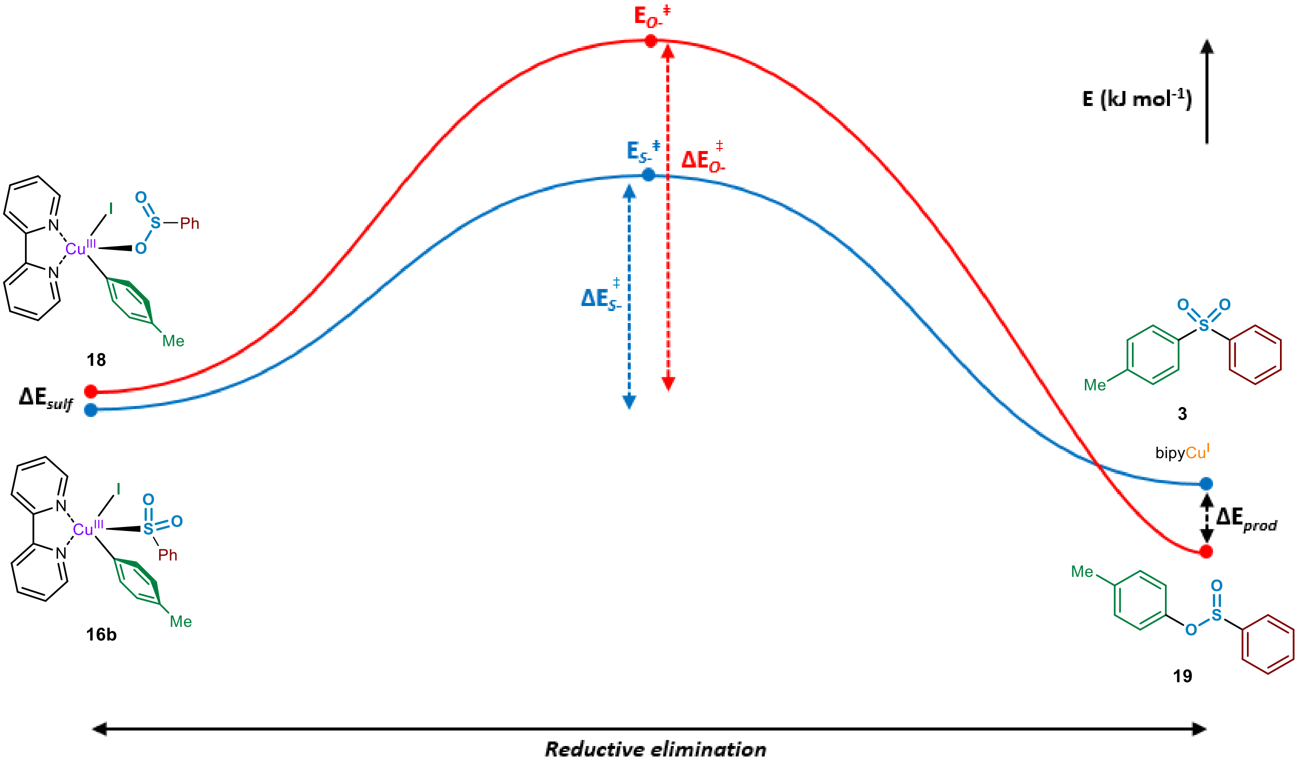

***O*-bound (18)**	**E_{O^-} (a.u.)**	**$\Delta E_{O^-}^{\ddagger}$ (a.u.)**	**$\Delta E_{O^-}^{\ddagger}$ (kJ mol^{-1})**	**$E_{O^-}^{\ddagger}$**
	-3198.55271298	+0.01791382	**+47.0**	-3198.53479916
	**ΔE_{prod} (a.u.)**	**ΔE_{prod} (kJ mol^{-1})**	**ΔE_{prod} (a.u.)**	
	-0.06370485	-167.3	-3198.59850401	
***S*-bound (16b)**	**E_{S^-} (a.u.)**	**$\Delta E_{S^-}^{\ddagger}$ (a.u.)**	**$\Delta E_{S^-}^{\ddagger}$ (kJ mol^{-1})**	**$E_{S^-}^{\ddagger}$**
	-3198.55386175	+0.01132921	+29.7	-3198.54253254
	**ΔE_{prod} (a.u.)**	**ΔE_{prod} (kJ mol^{-1})**	**ΔE_{prod} (a.u.)**	
	-0.05186838	-136.2	-3198.59440092	

Table S10. Calculated energy profile for the competing reductive elimination of sulfinic acid ester and sulfone from *O*-bound and *S*-bound copper(III) sulfinate species, respectively. Energy change values (ΔE) represent the following: $\Delta E_{O^-}^{\ddagger}$, the activation energy for reductive elimination from the *O*-bound copper(III) sulfinate to yield a sulfinic acid ester; $\Delta E_{S^-}^{\ddagger}$, the activation energy for reductive elimination from the *S*-bound copper(III)sulfinate to yield a sulfone; ΔE_{prod},the energy difference between transition state & resulting (bpy)CuI and respective reductive elimination product.

2.10. Reductive elimination energy profile construction: Variation of $\Delta E_{RE}^{‡}$ with ligand

Using the above calculated (geometry-optimised) energy values of each (ligand)copper(III) *p*-tolyl(S-sulfinylphenyl) iodide complex, (ligand)copper(III) *p*-tolyl(S-sulfinylphenyl) iodide complex reductive elimination transition state and resulting (ligand)copper(I) iodide and (*p*-tolyl)sulfonylbenzene product, energy values for the reductive elimination activation energy ($\Delta E_{RE}^{‡}$) and the difference between reductive elimination products and transition state can be calculated. A graphical representation of these energies, in addition to energy values for individual species (a.u.) and energy changes (a.u. and kJ mol^{-1}), are tabulated in Table S11.

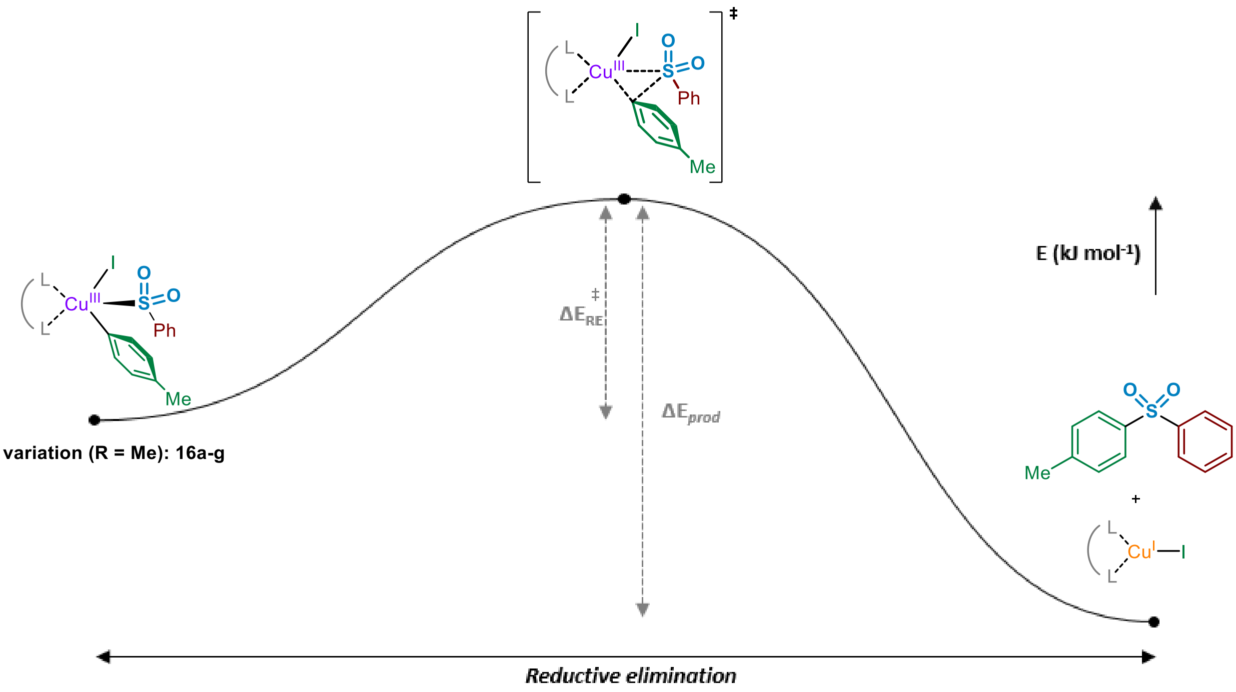

Ligand	$E_{Cu(III)}$	$\Delta E_{RE}^{‡}$ (a.u.)	$\Delta E_{RE}^{‡}$ (kJ mol^{-1})	$E_{RE}^{‡}$	ΔE_{prod} (a.u.)
**- (16a)**	-2703.06559220	+0.00092039	**+2.4**	-2703.06467181	-0.03386166
**bpy (16b)**	-3198.55386175	+0.01132921	**+29.7**	-3198.54253254	-0.05186838
**4,4'-diMeObpy (16c)**	-3427.63151675	+0.01187551	**+31.2**	-3427.61964124	-0.05091603
**4,4'-diNO$_2$bpy (16d)**	-3607.56011056	+0.00998644	**+26.2**	-3607.55012412	-0.05167654
**DMEDA (16e)**	-2972.30825871	+0.01393278	**+36.6**	-2972.29432593	-0.05428328
**Lprolinate (16f)**	-3103.87543824	+0.01489275	**+39.1**	-3103.86054549	-0.05243489
**DMPHPC (16g)**	-3469.40923851	+0.01198533	**+31.5**	-3469.39725318	-0.04746887

Ligand	ΔE_{prod} (kJ mol^{-1})	E_{prod}
**- (16a)**	**-88.9**	-2703.09853347
**bpy (16b)**	**-136.2**	-3198.59440092
**4,4'-diMeObpy (16c)**	**-133.7**	-3427.67055727
**4,4'-diNO$_2$bpy (16d)**	**-135.7**	-3607.60180066
**DMEDA (16e)**	**-142.5**	-2972.34860921
**Lprolinate (16f)**	**-137.7**	-3103.91298038
**DMPHPC (16g)**	**-124.6**	-3469.44472205

Table S11. Calculated energy barriers between species of the form (L)Cu^{III}(Tol)(SO_2Ph)(I), reductive elimination transition state, and the resulting species, (L)CuI and (*p*-tolyl)sulfonylbenzene, Tol(SO_2Ph). Energy change values (ΔE) represent the following: $\Delta E_{RE}^{‡}$, the activation energy for reductive elimination; ΔE_{prod},the energy difference between transition state & resulting (L)CuI and (*p*-tolyl)sulfonylbenzene, Tol(SO_2Ph).

2.11. Reductive elimination energy profile construction: Variation of $\Delta E_{RE}^{‡}$ with aryl R group

Using the above calculated (geometry-optimised) energy values of each (bpy)copper(III) aryl(*S*-sulfinylphenyl) iodide complex, (bpy)copper(III) aryl(*S*-sulfinylphenyl) iodide complex reductive elimination transition state and resulting (bpy)copper(I) iodide and biaryl sulfone product Ar(SO_2Ph), energy values for the reductive

elimination activation energy ($\Delta E_{RE}^{\ddagger}$) and the difference between reductive elimination products and transition state can be calculated. A graphical representation of these energies, in addition to energy values for individual species (a.u.) and energy changes (a.u. and kJ mol^{-1}), are tabulated in Table S12.

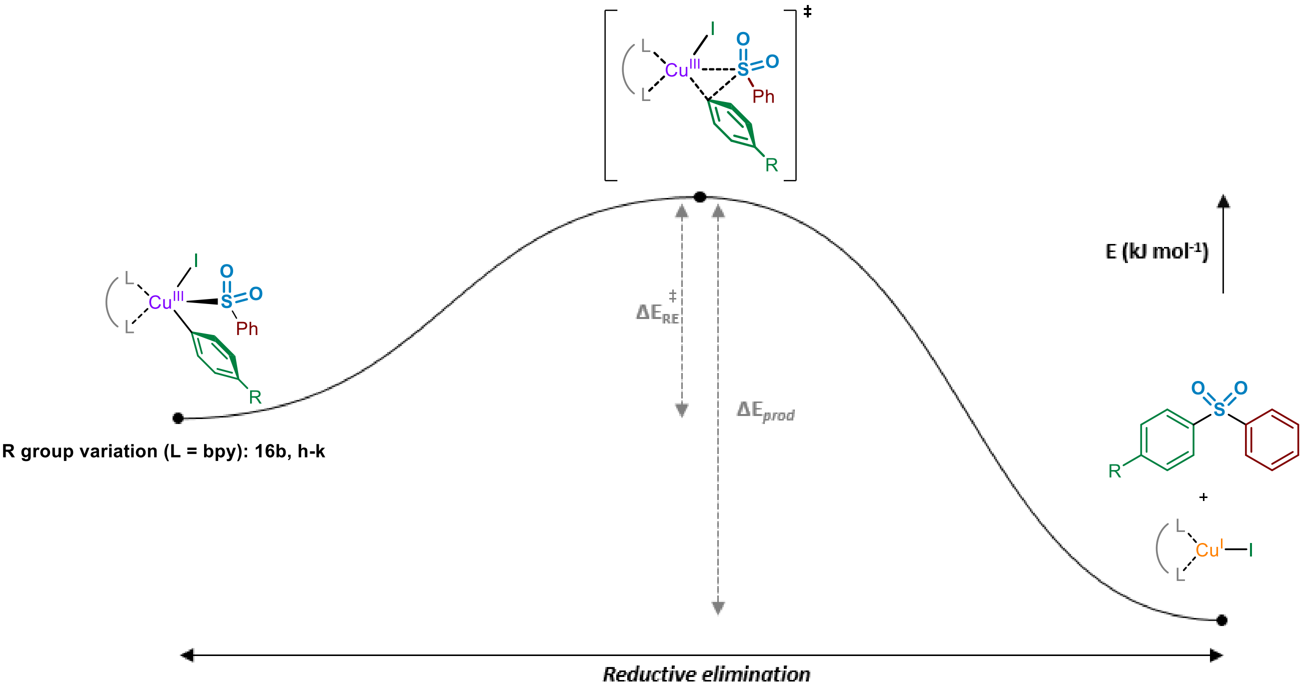

R group	$E_{Cu(III)}$	$\Delta E_{RE}^{\ddagger}$ (a.u.)	$\Delta E_{RE}^{\ddagger}$ (kJ mol^{-1})	$E_{RE}^{\ddagger}$	ΔE_{prod} (a.u.)
**H (16h)**	-3159.23128040	+0.01270118	**+33.3**	-3159.21857922	-0.05095089
**Me (16b)**	-3198.55386175	+0.01132921	**+29.7**	-3198.54253254	-0.05186838
**OMe (16i)**	-3273.76397481	+0.01020460	**+26.8**	-3273.75377021	-0.05303696
**NO_2 (16j)**	-3363.74734701	+0.01662555	**+43.7**	-3363.73072146	-0.05127688
**F (16k)**	-3258.47145376	+0.01275636	**+33.5**	-3258.45869740	-0.05137510

Ligand	ΔE_{prod} (kJ mol^{-1})	E_{prod}
**H (16h)**	**-133.8**	-3159.26953011
**Me (16b)**	**-136.2**	-3198.59440092
**OMe (16i)**	**-139.2**	-3273.80680717
**NO_2 (16j)**	**-134.6**	-3363.78199834
**F (16k)**	**-134.9**	-3258.51007250

Table S12. Calculated energy barriers between species of the form (bpy)Cu^{III}(Ar)(SO_2Ph)(I), reductive elimination transition state, and the resulting species, (bpy)CuI and biaryl sulfone Ar(SO_2Ph). Energy change values (ΔE) represent the following: $\Delta E_{RE}^{\ddagger}$, the activation energy for reductive elimination; ΔE_{prod},the energy difference between transition state & resulting (bpy)CuI and biaryl sulfone Ar(SO_2Ph).

2.12. Tabulated activation energies for key mechanistic steps in the copper(I)-catalysed sulfonylative Suzuki-Miyaura reaction of phenylboronic acid, sulfur dioxide, and 4-iodotoluene, using L=bpy

Mechanistic step	Visualisation of transition state	Activation energy ($\Delta E^{\ddagger}$, kJ mol^{-1})	Predicted rate determining step(s)
Transmetalation	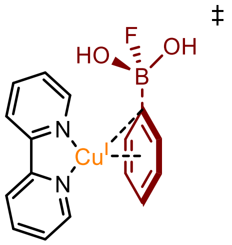	+81.0	✓
Migratory insertion	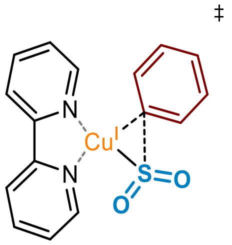	+28.6	✗
Oxidative addition	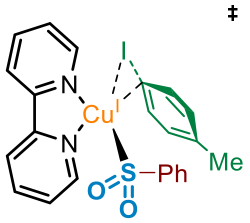	+53.3	✓
Reductive elimination	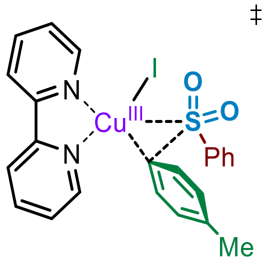	+29.7	✗

Table S13 – Summarised calculated activation energies for the key mechanistic steps of transmetalation, migratory insertion, oxidative addition, and reductive elimination, for the copper(I) catalysed sulfonylative Suzuki-Miyaura reaction of phenylboronic acid, sulfur dioxide, and 4-iodotoluene, using 2,2-bipyridine as the ligand.

Conclusion: Through comparison of computed activation energies for the key mechanistic steps of the catalytic cycle, the transmetalation and oxidative addition steps are predicted to most likely be the rate-determining steps. Although activation energies are generally an indication of the rate determining step of the process, due to the complexity of the catalytic cycle and number of species involved, it is hard in this instance to accurately pinpoint the rate-determining step. Furthermore, the dependence of the oxidative addition step on both the activation energy, and relative stability of the Cu^{III} species further complicates this comparison.

2.13. Overall energy profile for the copper(I)-catalysed sulfonylative Suzuki-Miyaura reaction of phenylboronic acid, sulfur dioxide, and 4-iodotoluene, using L=bpy

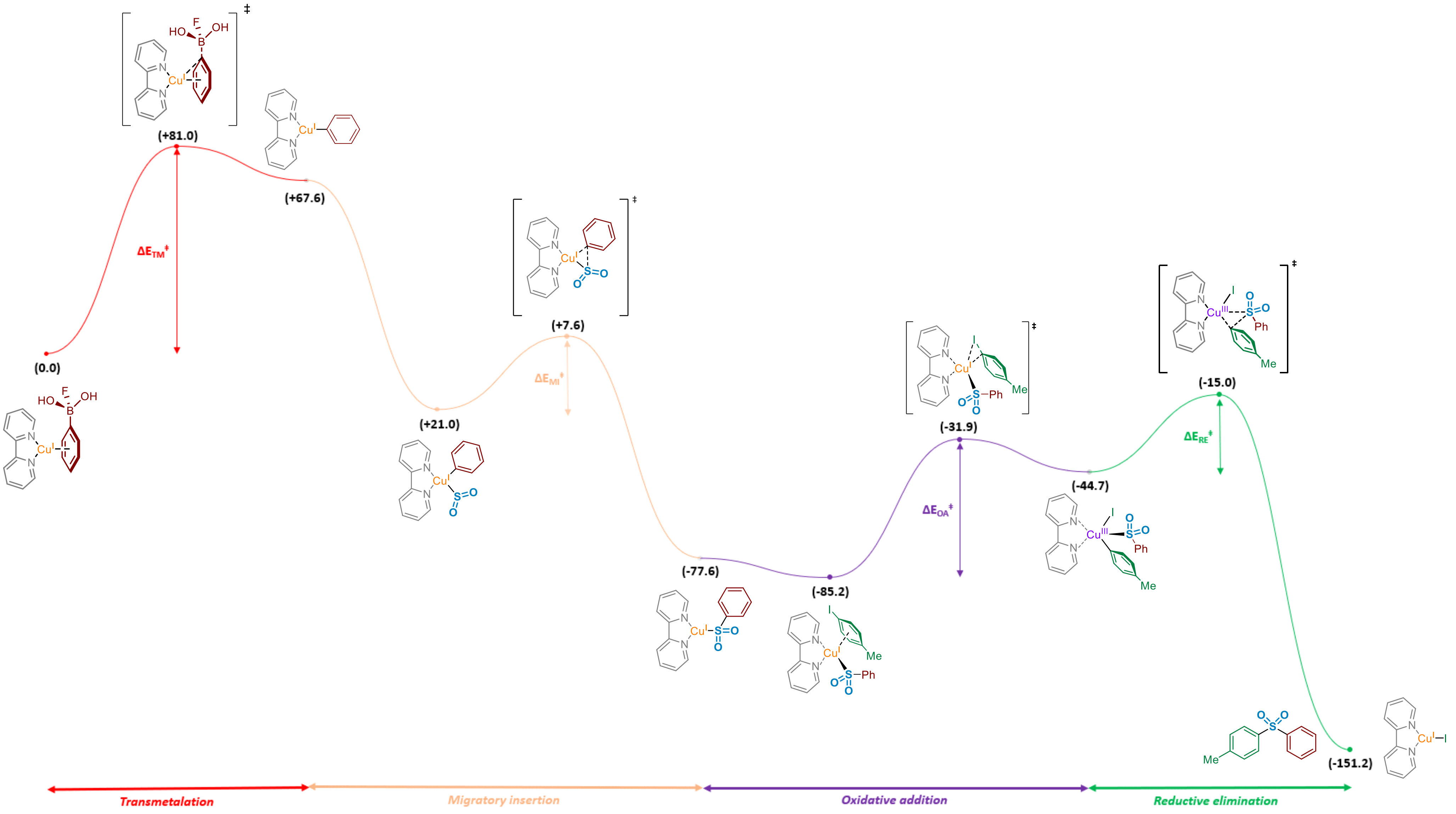

Figure S1 – Visualised overall energy profile for the copper(I)-catalysed sulfonylative Suzuki-Miyaura reaction of phenylboronic acid, sulfur dioxide, and 4-iodotoluene, using L=bpy, highlighting relative energies of all key intermediates in the catalytic cycle.

3. Computed atomic coordinates, energies, and vibrational frequencies for optimised ground state and transition state species

3.1. Transmetalation of phenylboronic acid onto copper(I)

3.1.1. Ground state geometry optimisations

Phenylboronic acid (**4**)

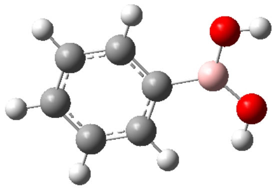

Geometry-optimised cartesian coordinates

Charge = 0 Multiplicity = 1

ATOM	X	Y	Z
C	-1.93338400	-1.22118600	0.00017100
C	-0.53666300	-1.20044300	0.00023800
C	0.17852600	0.01427500	0.00010000
C	-0.56506800	1.21017100	-0.00015100
C	-1.96248400	1.19951800	-0.00022000
C	-2.64978800	-0.01892400	-0.00005800
B	1.74825800	0.00031400	0.00025600
O	2.53902400	1.12613100	0.00024800
O	2.38766300	-1.21473600	-0.00046800
H	-2.46285900	-2.16978900	0.00029600
H	0.01291000	-2.13717500	0.00041900
H	-0.05979100	2.17363200	-0.00030300
H	-2.51277900	2.13597900	-0.00040800
H	-3.73604000	-0.03103600	-0.00011300
H	2.06562800	1.96658200	0.00065600
H	3.35131300	-1.13139100	-0.00054800

E(RB3LYP) -408.32479785 a.u.

(2,2'-Bipyridyl)copper(I)

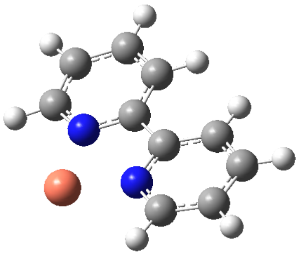

Geometry-optimised cartesian coordinates

Charge = 1 Multiplicity = 1

ATOM	X	Y	Z
C	0.74966200	0.73245800	-0.00056900
C	2.70467900	-0.54502100	0.04030300
C	3.51342400	0.58901600	0.00567500
C	2.89511800	1.83750100	-0.03909700
C	1.50229400	1.91100600	-0.04357100
C	-0.74856000	0.73282600	0.00197800
C	-2.70445300	-0.54347400	-0.03975000
C	-3.51246000	0.59109500	-0.00671000
C	-2.89341000	1.83921800	0.03806700
C	-1.50057200	1.91182400	0.04386000
H	3.12966400	-1.54182700	0.07222500
H	4.59245600	0.48738700	0.01127200
H	3.48529700	2.74712400	-0.07230000
H	1.02108200	2.87908200	-0.08603500
H	-3.12998400	-1.54004700	-0.07109100
H	-4.59155400	0.49018600	-0.01350000

H	-3.48305700	2.74922400	0.07018900
H	-1.01878100	2.87963000	0.08616200
N	-1.36417100	-0.47584400	-0.03512500
N	1.36449800	-0.47652000	0.03699300
Cu	-0.00143900	-1.95941100	-0.00038300

E(RB3LYP) -2135.67609404 a.u.

Fluoride anion

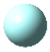

Geometry-optimised cartesian coordinates

Charge = -1 Multiplicity = 1

ATOM	X	Y	Z
F	0.00000000	0.00000000	0.00000000

E(RB3LYP) -99.99302637 a.u.

Phenylfluoroboronate (**11**)

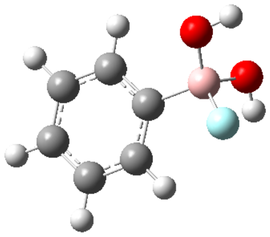

Geometry-optimised cartesian coordinates

Charge = -1 Multiplicity = 1

ATOM	X	Y	Z
B	1.55620100	0.02885600	0.01066600
C	-0.07393600	0.01822600	0.04777900
C	-0.81839500	1.21325600	0.00101600
C	-0.81046700	-1.18222400	0.06002600
C	-2.21837300	1.21711200	-0.03336700
H	-0.28434300	2.16095000	-0.00335900
C	-2.21091900	-1.19721500	0.02257300
H	-0.27226500	-2.12660100	0.10627900
C	-2.92295900	0.00689800	-0.02462300
H	-2.76061000	2.15971700	-0.06555100
H	-2.74635700	-2.14416200	0.03394200
H	-4.00970300	0.00273200	-0.05113500
O	2.08064500	1.18946600	0.72168000
O	2.10368200	0.03450900	-1.36502700
H	1.80884600	-0.75126600	-1.84047000
H	3.01927200	1.28365800	0.51477500
F	2.03553700	-1.21971400	0.66206000

E(RB3LYP) -508.34070621 a.u.

(2,2'-Bipyridyl)copper(I)-phenylfluoroboronate π adduct

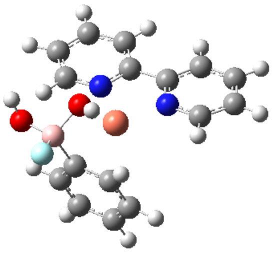

Geometry-optimised cartesian coordinates

Charge = 0 Multiplicity = 1

ATOM	X	Y	Z
C	-2.54349700	-0.23492700	-0.13022800
C	-1.89981500	-2.39319200	-0.72557900

C	-3.21178800	-2.85962400	-0.67423100
C	-4.21960200	-1.95837500	-0.32970000
C	-3.88293500	-0.63373500	-0.05399600
C	-2.08968200	1.15890600	0.14630400
C	-0.24576800	2.57402800	0.32513400
C	-1.05369800	3.67479100	0.61012600
C	-2.43570500	3.48687600	0.66077100
C	-2.96314300	2.21709900	0.42386400
H	-1.07501700	-3.05017200	-0.98050000
H	-3.42886100	-3.89839800	-0.89567000
H	-5.25451700	-2.27922400	-0.27278100
H	-4.65731900	0.07103200	0.22127200
H	0.83625200	2.64485100	0.25344000
H	-0.60756100	4.64771500	0.78375800
H	-3.09946900	4.31751800	0.87752900
H	-4.03500300	2.06649700	0.45095500
N	-0.75331900	1.35336100	0.10593000
N	-1.57115800	-1.11907800	-0.46356700
Cu	0.31691600	-0.35128000	-0.38700300
B	2.75732200	0.50737800	-1.09684000
C	2.38801600	-0.35188600	0.24727700
C	2.43941300	0.19261000	1.55821900
C	2.01097700	-1.72121900	0.14189200
C	2.15396100	-0.57222700	2.68595000
H	2.71131200	1.23818800	1.66901400
C	1.71914800	-2.49769800	1.28719300
H	2.06773400	-2.22473500	-0.82256000
C	1.78901400	-1.92506700	2.55281800
H	2.20874700	-0.12375900	3.67462800
H	1.44786900	-3.54351700	1.17201400
H	1.56475300	-2.51843800	3.43481300
O	2.90254600	1.90743800	-0.76288200
O	1.64747900	0.33474800	-2.10338800
H	1.85047400	-0.39067300	-2.70717700
H	2.93876000	2.43024700	-1.57358600
F	3.96627000	-0.02805900	-1.72212600

E(RB3LYP) -2644.07375476 a.u.

<u>(2,2'-Bipyridyl) phenylcopper(I)</u>

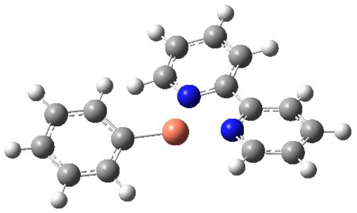

Geometry-optimised cartesian coordinates

Charge = 0 Multiplicity = 1

**ATOM**	**X**	**Y**	**Z**
C	2.38060200	-0.41980900	0.00695300
C	1.57489100	-2.60206800	0.11623100
C	2.85990700	-3.13842300	0.11417600
C	3.94244600	-2.26099500	0.05137400
C	3.69910800	-0.88997400	-0.00427500
C	2.04765900	1.03091300	-0.05379600
C	0.39272100	2.60030600	-0.44981800
C	1.26131100	3.66551800	-0.20221100
C	2.58067600	3.37120800	0.14478000
C	2.98299700	2.03765600	0.22369600
N	0.77270800	1.31997500	-0.37932900
N	1.33173100	-1.27919200	0.06276800
Cu	-0.54826100	-0.62054700	-0.02336500
C	-2.45273700	-0.39470100	0.03983100
C	-3.39999500	-1.37430900	-0.35060800
C	-3.00838200	0.83844200	0.46623600
C	-4.78376600	-1.14818500	-0.32583100
C	-4.38857600	1.08223300	0.50273600
C	-5.28696800	0.08561900	0.10358700

H	0.69938300	-3.23958700	0.16611700
H	2.99880300	-4.21252600	0.15874900
H	4.96086300	-2.63509700	0.03943500
H	4.52830500	-0.19716000	-0.07449100
H	-0.64689400	2.77265500	-0.71515900
H	0.90885600	4.68860300	-0.27496200
H	3.28790100	4.16607900	0.35866900
H	3.99854900	1.79626200	0.51352300
H	-3.04763500	-2.34806100	-0.68726800
H	-2.34005900	1.63848700	0.78219500
H	-5.46926000	-1.93367500	-0.63887000
H	-4.76292600	2.04707700	0.84007300
H	-6.35875500	0.26673600	0.12712100

E(RB3LYP) -2135.67609404 a.u.

Fluoroboric acid

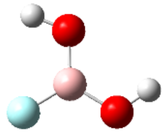

Geometry-optimised cartesian coordinates
Charge = 0 Multiplicity = 1

**ATOM**	**X**	**Y**	**Z**
B	0.01415000	-0.00417600	-0.00002700
O	1.36685800	0.11397500	0.00006800
O	-0.81746400	1.07315800	-0.00000900
F	-0.48724700	-1.26114000	-0.00001900
H	1.67600500	1.02948800	-0.00034500
H	-1.75668700	0.84458400	0.00017800

E(RB3LYP) -276.54894956 a.u.

3.1.2. Transition state optimisations

(2,2'-Bipyridyl)copper(I)-phenylfluoroboronate transmetalation transition state‡

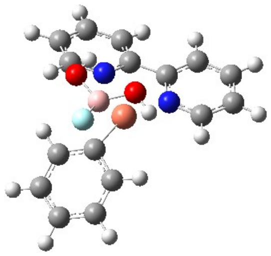

Geometry-optimised cartesian coordinates
Charge = 0 Multiplicity = 1

**ATOM**	**X**	**Y**	**Z**
C	2.65327200	-0.59987000	-0.22755900
C	1.57836000	-2.66487000	-0.24426700
C	2.78687100	-3.35734200	-0.22201800
C	3.97098000	-2.62004700	-0.19612900
C	3.90379100	-1.22756100	-0.19769600
C	2.48904200	0.88072600	-0.23201400
C	1.00486300	2.64791200	0.01211800
C	2.00126300	3.59393600	-0.22913400
C	3.29398000	3.13726500	-0.48820000
C	3.54343900	1.76482800	-0.49275900
H	0.62811500	-3.18714800	-0.26266600
H	2.79141000	-4.44135800	-0.22236700
H	4.93438100	-3.11843600	-0.16997800
H	4.81488900	-0.64364200	-0.15974500
H	-0.01862600	2.94593100	0.21800200
H	1.76430300	4.65191400	-0.21646300
H	4.09848500	3.83667200	-0.69070000
H	4.53860200	1.39828400	-0.71238100

N	1.23907900	1.32872600	0.01393500
N	1.50767000	-1.32310500	-0.24726000
Cu	-0.23650500	-0.32794500	-0.08634400
B	-1.88255400	0.08694100	1.92892300
C	-2.18308400	-0.14708100	-0.27017200
C	-2.68140100	1.06501200	-0.80433500
C	-2.89091300	-1.31810600	-0.63873400
C	-3.79644000	1.11522200	-1.64819900
H	-2.18767900	1.99601300	-0.53399800
C	-4.00850400	-1.28974800	-1.48043800
H	-2.56040100	-2.28005400	-0.24905700
C	-4.46536700	-0.06712000	-1.98841600
H	-4.14767200	2.06842600	-2.03724500
H	-4.52317000	-2.21204500	-1.74070200
H	-5.33539800	-0.03593200	-2.63934600
O	-1.33992500	1.35725300	2.14324800
O	-1.13207200	-1.01663300	2.38487200
H	-1.55186800	-1.85568200	2.15948000
H	-0.37483300	1.33364300	2.11855900
F	-3.25014400	0.04176500	2.16599900

**E(RB3LYP)**	-2644.04291861	a.u.
**ν**	-246.84	cm^{-1}

3.2. Insertion of SO_2 into the copper–carbon bond

3.2.1. SO_2 insertion into the copper–carbon bond: variation of $\Delta E_{MI}^{‡}$ with aryl R group

For all geometry optimisations in the following section, the ligand is 2,2'-bipyridine, with variation of the migrating 4-substituted aryl group bound to copper.

3.2.1.1. Aryl group: Phenyl (R = H)

See section 3.2.2.2 for the previously reported geometry-optimised structures of (2,2'-bipyridyl) phenylcopper(I) sulfur dioxide complex and (2,2'-bipyridyl) phenylcopper(I) sulfur dioxide complex migratory insertion transition state‡.

3.2.1.2. Aryl group: *p*-Tolyl (R = Me)

(2,2'-Bipyridyl) *p*-tolylcopper(I) sulfur dioxide complex (**12b**)

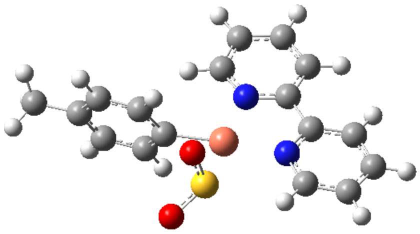

Geometry-optimised cartesian coordinates

Charge = 0 Multiplicity = 1

**ATOM**	**X**	**Y**	**Z**
C	2.90937500	0.39202700	-0.08623800
C	2.76037800	-1.92194800	-0.27945900
C	4.14095700	-2.05630200	-0.40746300
C	4.92340000	-0.90371900	-0.36999900
C	4.30060500	0.33315700	-0.20710900
C	2.16161500	1.66527200	0.08171000
C	0.04574000	2.62488400	0.22939600
C	0.57949100	3.90432900	0.37932600
C	1.96729700	4.04571300	0.38103300
C	2.77134200	2.91528300	0.23083500
N	0.81773800	1.54116400	0.08555300
N	2.15525500	-0.73602900	-0.12427300
Cu	0.12705400	-0.44292000	-0.02753800
S	-0.48124600	-2.72696200	0.23896500
O	-0.96091000	-2.78266500	1.66929900
O	-1.48682600	-3.23665100	-0.75942600

C	-1.77695900	-0.15142100	-0.10792500
C	-2.30663900	0.38016300	-1.29524000
C	-2.61411700	-0.22505700	1.00938800
C	-3.63390300	0.82445700	-1.35598700
C	-3.93867400	0.22923300	0.95295900
C	-4.47244400	0.75726600	-0.23228300
H	2.10814700	-2.78854000	-0.29634400
H	4.57925900	-3.03959900	-0.53224800
H	6.00237200	-0.96096800	-0.46722600
H	4.89617300	1.23657900	-0.18237600
H	-1.02369800	2.44331900	0.21999400
H	-0.07813600	4.75851200	0.49311400
H	2.42343800	5.02296400	0.49953800
H	3.84923300	3.01568900	0.23755400
H	-1.68487000	0.46357900	-2.18426900
H	-2.23957500	-0.66558900	1.93130400
H	-4.02013200	1.23265000	-2.28838600
H	-4.56564400	0.16822300	1.84087000
C	-5.91358100	1.20977900	-0.30823900
H	-6.03302500	2.05025200	-0.99971500
H	-6.56265400	0.40021500	-0.66572100
H	-6.28787700	1.51867000	0.67290800

E(RB3LYP) -2955.46237133 a.u.

(2,2′-Bipyridyl) *p*-tolylcopper(I) sulfur dioxide complex migratory insertion transition state‡

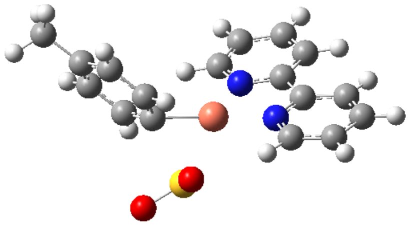

Geometry-optimised cartesian coordinates

Charge = 0 Multiplicity = 1

**ATOM**	**X**	**Y**	**Z**
C	2.86056100	-0.06542400	0.37260100
C	2.08637700	-2.24088800	0.70615100
C	3.35248900	-2.69006900	1.07813200
C	4.40349000	-1.77264800	1.08756500
C	4.15816900	-0.44653300	0.73020500
C	2.49018500	1.32433700	-0.02432800
C	0.78028000	2.72034400	-0.76954800
C	1.62052100	3.83250100	-0.78689900
C	2.94923100	3.66061100	-0.39820600
C	3.39050100	2.39512000	-0.01101200
N	1.20324400	1.50310900	-0.40303900
N	1.85011300	-0.96512200	0.36701700
Cu	0.04389400	-0.20046100	-0.22484300
S	-1.09964200	-2.08603900	-1.30468800
O	-1.15275300	-3.10756400	-0.18430200
O	-2.28813100	-2.16158300	-2.24042700
C	-1.93246800	-0.28562900	-0.13550900
C	-2.60362100	0.73586000	-0.83196200
C	-2.39407400	-0.65807200	1.13227300
C	-3.67919800	1.40015000	-0.24330400
C	-3.47009000	0.01488200	1.72390500
C	-4.12792300	1.05118200	1.04531100
H	1.22456600	-2.90069300	0.66737200
H	3.50332100	-3.72901400	1.34830300
H	5.40528200	-2.08197100	1.36691300
H	4.97083900	0.26873000	0.72792800
H	-0.26519900	2.79110500	-1.05246300
H	1.23823900	4.79949100	-1.09331800
H	3.63676500	4.49992400	-0.39226700
H	4.41809300	2.25632600	0.30003400
H	-2.28053500	1.01299800	-1.83261200
H	-1.92119400	-1.49489100	1.64224600

H	-4.18182300	2.19923200	-0.78434900
H	-3.80945900	-0.27575400	2.71570100
C	-5.31400000	1.75819100	1.65771000
H	-5.39274000	1.55460100	2.72942800
H	-5.24464200	2.84226000	1.51751200
H	-6.24917400	1.43142400	1.18650600

E(RB3LYP) -2955.45255569 a.u.
ν -167.82 cm^{-1}

3.2.1.3. Aryl group: 4-Methoxyphenyl (R = OMe)

(2,2'-Bipyridyl) 4-methoxyphenylcopper(I) sulfur dioxide complex (**12a**)

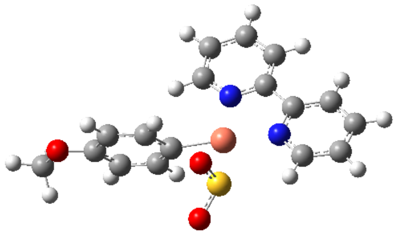

Geometry-optimised cartesian coordinates
Charge = 0 Multiplicity = 1

**ATOM**	**X**	**Y**	**Z**
C	3.12295800	0.57546000	-0.13370800
C	3.14441900	-1.74097700	-0.34929400
C	4.52681200	-1.76888100	-0.51808200
C	5.22035500	-0.56060100	-0.49009200
C	4.51044200	0.62361000	-0.29561200
C	2.28672500	1.78659900	0.07220200
C	0.11081200	2.57878900	0.31012200
C	0.54946600	3.89529000	0.44697000
C	1.92108100	4.14389700	0.39409300
C	2.80351300	3.07973000	0.20494000
N	0.95777400	1.55875200	0.12838600
N	2.45554900	-0.60637600	-0.16289200
Cu	0.41482600	-0.47240300	0.01212400
S	-0.00695100	-2.81005800	0.18635300
O	-0.46386100	-2.99074700	1.61308100
O	-0.97666600	-3.34025100	-0.83626800
C	-1.50873600	-0.33356000	0.02815100
C	-2.13649500	0.19054400	-1.10905300
C	-2.29135200	-0.52459000	1.17537300
C	-3.50159400	0.52313200	-1.11475800
C	-3.64671200	-0.19046500	1.19744800
C	-4.25849000	0.33484300	0.04881400
H	2.55985600	-2.65468000	-0.35848400
H	5.03417200	-2.71503500	-0.66631900
H	6.29727300	-0.53458300	-0.61837600
H	5.03642000	1.56933000	-0.27725700
H	-0.94086800	2.31476900	0.34155200
H	-0.16743900	4.69530400	0.59228800
H	2.30472100	5.15322500	0.49999000
H	3.86969500	3.26404900	0.16826000
H	-1.56763600	0.35806900	-2.02113200
H	-1.84324400	-0.96884600	2.06137300
H	-3.94723700	0.92302300	-2.01836800
H	-4.24725500	-0.33593900	2.09136600
O	-5.59563200	0.62882000	0.16397200
C	-6.27298100	1.16158500	-0.97528300
H	-6.24843600	0.45994400	-1.81786600
H	-7.30614400	1.31684000	-0.66286700
H	-5.83797200	2.11951200	-1.28551700

E(RB3LYP) -3030.67374712 a.u.

(2,2'-Bipyridyl) 4-methoxyphenylcopper(I) sulfur dioxide complex migratory insertion transition state‡

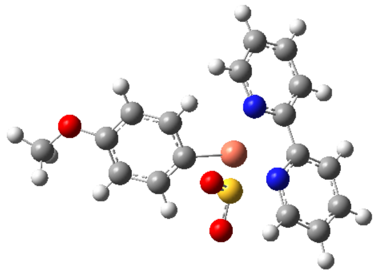

Geometry-optimised cartesian coordinates

Charge = 0 Multiplicity = 1

ATOM	X	Y	Z
C	3.08381500	0.08388200	-0.49571200
C	2.41261300	-2.12163700	-0.85326000
C	3.67813000	-2.48802000	-1.30850400
C	4.67466700	-1.51254600	-1.34886600
C	4.37744300	-0.21298200	-0.93752400
C	2.66174300	1.43848400	-0.03460700
C	0.92062400	2.71302000	0.84213900
C	1.69923400	3.86943500	0.85446500
C	3.01359400	3.78351800	0.39476400
C	3.50196500	2.55697700	-0.05635400
N	1.38893300	1.53351000	0.41384800
N	2.12575800	-0.87119400	-0.46205400
Cu	0.31350800	-0.23640600	0.24142100
S	-0.63537200	-2.25674200	1.34696400
O	-1.74466200	-2.44308000	2.35921600
O	-0.72939000	-3.21425100	0.17518500
C	-1.64479700	-0.41879300	0.34991800
C	-2.24224400	-0.78130700	-0.86181500
C	-2.30284000	0.52184800	1.16950800
C	-3.43202400	-0.18536700	-1.30060000
C	-3.48711100	1.11970600	0.75665700
C	-4.05354200	0.77242600	-0.48609000
H	1.59260700	-2.83071800	-0.78812000
H	3.86992800	-3.50899800	-1.61831800
H	5.67409700	-1.75647600	-1.69376000
H	5.14777600	0.54734500	-0.95974500
H	-0.11088200	2.71637100	1.18035100
H	1.28198200	4.80394700	1.21189200
H	3.65387200	4.65934200	0.38325700
H	4.51866300	2.48477800	-0.42129100
H	-1.78407300	-1.56567900	-1.46074500
H	-1.88125100	0.78774200	2.13573800
H	-3.86291600	-0.48105500	-2.24976200
H	-3.99515500	1.85493800	1.37393000
O	-5.21728100	1.41328100	-0.79933000
C	-5.85961000	1.10325100	-2.04069000
H	-6.14985300	0.04723100	-2.08218300
H	-6.75178600	1.72845800	-2.07605000
H	-5.21092500	1.34121900	-2.89150800

E(RB3LYP) -3030.66499867 a.u.

ν -159.93 cm^{-1}

3.2.1.4. Aryl group: 4-Nitrophenyl (R = NO_2)

(2,2'-Bipyridyl) 4-nitrophenylcopper(I) sulfur dioxide complex (**12e**)

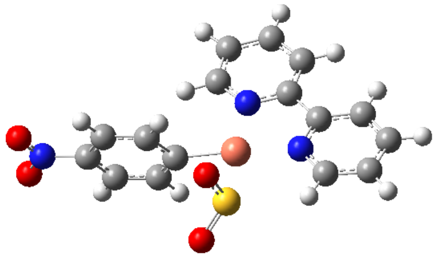

Geometry-optimised cartesian coordinates

Charge = 0 Multiplicity = 1

ATOM	X	Y	Z
C	3.31363100	0.55129000	-0.05594400
C	3.31361700	-1.76924200	-0.23604600
C	4.70326900	-1.81950100	-0.31327400
C	5.41207000	-0.62147700	-0.25345900
C	4.70938100	0.57569900	-0.12304700
C	2.48567000	1.77849100	0.07393400
C	0.31372900	2.60670400	0.20866200
C	0.76506500	3.92170700	0.31092800
C	2.14068900	4.15131100	0.29063000
C	3.01403400	3.06995600	0.17053900
N	1.15205500	1.56943000	0.09393200
N	2.63147500	-0.62198600	-0.11084600
Cu	0.60117900	-0.45106700	-0.02663300
S	0.12756200	-2.82082000	0.11208500
O	-0.37929700	-2.99557900	1.51752900
O	-0.82561300	-3.29091100	-0.94840500
C	-1.31916600	-0.31631300	-0.06025400
C	-1.93804600	0.15322000	-1.23561100
C	-2.10883200	-0.47158800	1.09067500
C	-3.29728600	0.45972800	-1.26813900
C	-3.46793900	-0.16344900	1.08973900
C	-4.04735500	0.30049400	-0.09704100
H	2.71882200	-2.67524800	-0.27236600
H	5.20410900	-2.77508100	-0.41558600
H	6.49548700	-0.61316700	-0.30757500
H	5.24869600	1.51285000	-0.07675700
H	-0.74288100	2.36218400	0.21559200
H	0.05435300	4.73491900	0.40312400
H	2.53445200	5.15926900	0.36767500
H	4.08281500	3.24063800	0.15528300
H	-1.35570600	0.28651400	-2.14303900
H	-1.66033800	-0.86798800	1.99739400
H	-3.77245800	0.81738900	-2.17353400
H	-4.07355000	-0.28004600	1.98036400
N	-5.46735300	0.62590000	-0.11430100
O	-5.96929600	1.03868400	-1.16949300
O	-6.12297000	0.47983500	0.92701800

E(RB3LYP) -3120.66229754 a.u.

(2,2'-Bipyridyl) 4-nitrophenylcopper(I) sulfur dioxide complex migratory insertion transition state‡

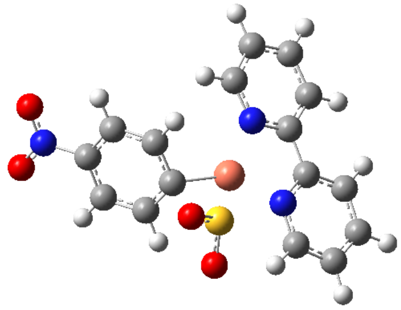

Geometry-optimised cartesian coordinates

Charge = 0 Multiplicity = 1

ATOM	X	Y	Z
C	3.18138700	0.35947900	-0.43847900
C	2.78768900	-1.89891300	-0.88157100
C	4.09432200	-2.09227800	-1.32728000
C	4.96469300	-1.00237700	-1.31740100
C	4.50655700	0.23676700	-0.86824800
C	2.59238700	1.63898000	0.05661200
C	0.69076100	2.68126700	0.91108900
C	1.33624600	3.91396300	0.99481400
C	2.66753200	3.98937700	0.58562700
C	3.30375800	2.84219700	0.11060500
N	1.30106400	1.57814800	0.45821000
N	2.34773600	-0.70677100	-0.45255700
Cu	0.47465500	-0.29752300	0.22704200
S	-0.36057600	-2.58300000	1.26074100
O	-1.53582200	-3.02658300	2.10199600
O	-0.20366800	-3.38999300	-0.01109200
C	-1.43462400	-0.81219800	0.39080200
C	-1.91536200	-1.06801200	-0.90406000
C	-2.16510500	0.03659300	1.24716500
C	-3.06384400	-0.43374200	-1.37765900
C	-3.31727300	0.67300300	0.79833300
C	-3.74388800	0.43054600	-0.51505300
H	2.05775300	-2.70251800	-0.85462100
H	4.41323100	-3.07055600	-1.66836800
H	5.99049800	-1.11064200	-1.65379500
H	5.17830300	1.08550700	-0.85527900
H	-0.34517500	2.55954000	1.21120600
H	0.80580400	4.78229100	1.36846100
H	3.20807600	4.92892700	0.63225700
H	4.33546800	2.89511400	-0.21272100
H	-1.38884000	-1.78521800	-1.52902800
H	-1.82206600	0.20749600	2.26337000
H	-3.43301100	-0.60372900	-2.38148700
H	-3.87776100	1.34523500	1.43603800
N	-4.95182000	1.10221800	-0.99826200
O	-5.32898100	0.87693600	-2.15455200
O	-5.54507000	1.86984400	-0.23080500

E(RB3LYP) -3120.64700236 a.u.

ν -154.17 cm^{-1}

3.2.1.5. Aryl group: 4-Fluorophenyl (R = F)

(2,2'-Bipyridyl) 4-fluorophenylcopper(I) sulfur dioxide complex (**12d**)

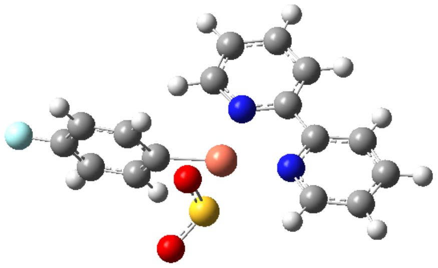

Geometry-optimised cartesian coordinates

Charge = 0 Multiplicity = 1

ATOM	X	Y	Z
C	2.90270000	0.34071600	-0.07400400
C	2.69964400	-1.96965700	-0.26081800
C	4.07782600	-2.13834600	-0.37140100
C	4.88799100	-1.00550200	-0.32657700
C	4.29346100	0.24660800	-0.17552100
C	2.18563900	1.63335600	0.07862700
C	0.09371400	2.64482200	0.21959300
C	0.65774200	3.91297200	0.35294400
C	2.04844000	4.02158400	0.34769000
C	2.82500100	2.87070300	0.20902200
N	0.83913100	1.54130000	0.08681300
N	2.12138800	-0.76890700	-0.11688800
Cu	0.10404500	-0.42676100	-0.02818400
S	-0.55534100	-2.71390200	0.20757100
O	-1.04413900	-2.77754500	1.63252300
O	-1.56493200	-3.18016300	-0.80489300
C	-1.79609200	-0.10047400	-0.10295200
C	-2.32078200	0.44482400	-1.28738300
C	-2.63206400	-0.18322600	1.01700300
C	-3.64615600	0.89721600	-1.35806300
C	-3.95752700	0.27211000	0.97799300
C	-4.43009400	0.80022800	-0.21594600
H	2.02769800	-2.82072800	-0.28375000
H	4.49301400	-3.13258100	-0.48805800
H	5.96643600	-1.08946700	-0.40880800
H	4.91069900	1.13496500	-0.14334400
H	-0.97983600	2.48989000	0.21540000
H	0.02067800	4.78359100	0.45860800
H	2.52787100	4.98929400	0.45162300
H	3.90494200	2.94679600	0.20841600
H	-1.69880400	0.52993200	-2.17501200
H	-2.25939800	-0.63743500	1.93200400
H	-4.06056300	1.31550400	-2.26999200
H	-4.60994400	0.21393400	1.84371200
F	-5.72915000	1.24467700	-0.27070700

E(RB3LYP) -3015.38193959 a.u.

(2,2'-Bipyridyl) 4-fluorophenylcopper(I) sulfur dioxide complex migratory insertion transition state‡

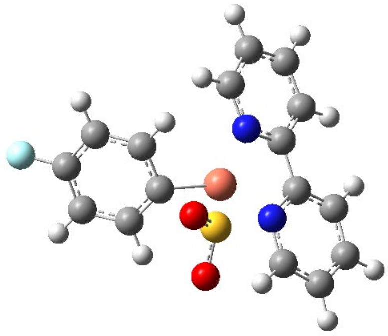

Geometry-optimised cartesian coordinates

Charge = 0 Multiplicity = 1

ATOM	X	Y	Z
C	2.84490000	-0.14576000	-0.35527800
C	1.99019200	-2.29192200	-0.68287300
C	3.24232700	-2.79301600	-1.03564500
C	4.32848600	-1.91762400	-1.03801200
C	4.13091700	-0.58030900	-0.69290100
C	2.52564900	1.26098300	0.02673500
C	0.86593500	2.73170700	0.74124400
C	1.75077400	3.80859600	0.76121200
C	3.07570100	3.57946700	0.38984300
C	3.46901200	2.29408800	0.01672200
N	1.24242600	1.49527300	0.38804400
N	1.79987100	-1.00520300	-0.35616500
Cu	0.02075400	-0.16149600	0.20794100
S	-1.20378600	-2.02874700	1.32931800
O	-2.41247400	-2.07098000	2.23790500
O	-1.25246900	-3.05857400	0.21869200
C	-1.95950600	-0.23259500	0.12440600
C	-2.41245500	-0.61499500	-1.14638000
C	-2.60882300	0.81688600	0.80070100
C	-3.45989500	0.07000800	-1.77297000
C	-3.66036100	1.51025600	0.19838300
C	-4.05295900	1.11765500	-1.07926800
H	1.10321400	-2.91766900	-0.64907100
H	3.35562300	-3.83911200	-1.29635600
H	5.32080800	-2.26807700	-1.30209900
H	4.97094600	0.10240900	-0.68462100
H	-0.17864200	2.84827600	1.01167900
H	1.40482500	4.79257700	1.05645100
H	3.79736100	4.38965900	0.38648300
H	4.49378400	2.11135500	-0.28078100
H	-1.95637200	-1.47365300	-1.63337900
H	-2.29225100	1.09553800	1.80215400
H	-3.82014200	-0.20214800	-2.75966700
H	-4.17003200	2.32989800	0.69421300
F	-5.08514300	1.78885100	-1.67286900

**E(RB3LYP)**	-3015.37074131	a.u.
**ν**	-161.61	cm^{-1}

3.2.2. SO_2 insertion into the copper–carbon bond: variation of $\Delta E_{MI}^{‡}$ with complex ligand

For all geometry optimisations in the following section, the migrating aryl group on copper is phenyl, with variation of the ligand bound to copper.

3.2.2.1. Ligand: None

Phenylcopper(I) sulfur dioxide complex (**13a**)

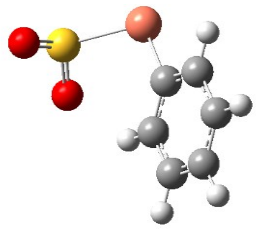

Geometry-optimised cartesian coordinates

Charge = 0 Multiplicity = 1

**ATOM**	**X**	**Y**	**Z**
Cu	-0.72128000	1.19650000	-0.36588100
S	-2.32405400	-0.36784900	0.12470900
O	-2.08892300	-1.55225300	-0.74175000
O	-2.40288100	-0.62910700	1.58383600
C	0.96823400	0.34100100	-0.11147600
C	2.05790400	1.10099000	0.34978300
C	1.15813200	-1.01360700	-0.41107600
C	3.32088000	0.50991900	0.49351800
C	2.42163100	-1.60380100	-0.27058200
C	3.50391200	-0.84183800	0.18275400
H	1.93406500	2.15152600	0.59739000
H	0.32217000	-1.62108600	-0.75360200
H	4.15686900	1.10630000	0.85008400
H	2.55664600	-2.65527700	-0.51073700
H	4.48252300	-1.29947100	0.29785500

E(RB3LYP) -2420.63226902 a.u.

Phenylcopper(I) sulfur dioxide complex migratory insertion transition state‡

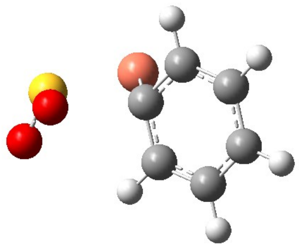

Geometry-optimised cartesian coordinates

Charge = 0 Multiplicity = 1

**ATOM**	**X**	**Y**	**Z**
Cu	0.48145700	1.86829700	-0.04730500
S	1.73287500	-0.94868500	-0.35034900
O	2.29443700	-0.74531600	1.03203700
O	1.21754700	-2.34428000	-0.56905700
C	-0.30008500	0.08817100	-0.09175400
C	-1.16522400	-0.02692300	-1.20875800
C	-0.80726900	-0.23305400	1.18984500
C	-2.49050400	-0.43112700	-1.04408500
C	-2.13379500	-0.63059800	1.35665200
C	-2.97124900	-0.73435400	0.23736100
H	-0.78574900	0.18607900	-2.20479400
H	-0.12996600	-0.20626700	2.04085100
H	-3.14708500	-0.51885600	-1.90502700
H	-2.51393800	-0.87438800	2.34463000
H	-3.99861500	-1.06413400	0.36235400

E(RB3LYP) -2420.61653111 a.u.

ν -142.18 cm^{-1}

3.2.2.2. Ligand: 2,2,'-Bipyridine (bpy)

(2,2'-Bipyridyl) phenylcopper(I) sulfur dioxide complex (**12c**)

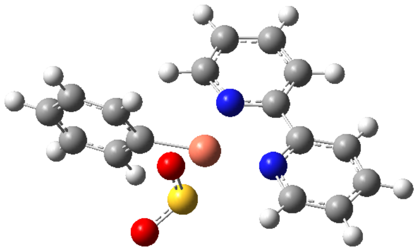

Geometry-optimised cartesian coordinates

Charge = 0 Multiplicity = 1

ATOM	X	Y	Z
C	2.68296000	0.05449100	-0.04605600
C	2.19781700	-2.21551700	-0.20460200
C	3.54649000	-2.55600000	-0.27844300
C	4.49010500	-1.53169200	-0.23135100
C	4.05316900	-0.21281500	-0.11396100
C	2.12903200	1.42822500	0.07366000
C	0.17702400	2.69315100	0.16881100
C	0.89247500	3.88410800	0.28651200
C	2.28584900	3.81984000	0.29731000
C	2.91527300	2.57923200	0.19009800
N	0.78140800	1.50345200	0.06564600
N	1.77079100	-0.94995600	-0.09178000
Cu	-0.19166000	-0.35783800	-0.04154600
S	-1.14236200	-2.53537600	0.15188000
O	-1.65391300	-2.56406200	1.57137300
O	-2.19143800	-2.85358200	-0.88002300
C	-2.03410600	0.20715100	-0.12391700
C	-2.48040800	0.81171500	-1.31136400
C	-2.86771500	0.24457500	0.99962800
C	-3.73165900	1.44089300	-1.37031000
C	-4.11480200	0.88314400	0.95183700
C	-4.55080800	1.48137500	-0.23595500
H	1.42513500	-2.97628900	-0.23342800
H	3.83704900	-3.59611600	-0.36973600
H	5.55122900	-1.75064800	-0.28598900
H	4.77540000	0.59259500	-0.08099200
H	-0.90734000	2.67215500	0.15228700
H	0.36694900	4.82870900	0.36785500
H	2.88051200	4.72261200	0.38947400
H	3.99623600	2.52091500	0.20195200
H	-1.85249100	0.80180700	-2.19972100
H	-2.55433200	-0.25465100	1.91424900
H	-4.06322200	1.89954900	-2.29899500
H	-4.74602500	0.90701200	1.83718100
H	-5.51992300	1.97109500	-0.27862200

E(RB3LYP) -2916.13931626 a.u.

(2,2'-Bipyridyl) phenylcopper(I) sulfur dioxide complex migratory insertion transition state‡

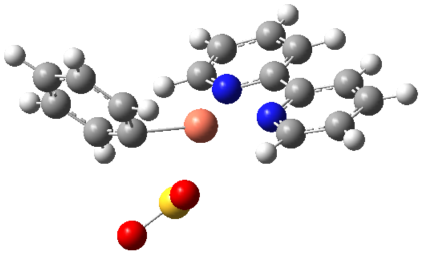

Geometry-optimised cartesian coordinates
Charge = 0 Multiplicity = 1

ATOM	X	Y	Z
C	2.59552000	-0.46354100	0.24547300
C	1.46922900	-2.49341500	0.47903200
C	2.65291700	-3.17865300	0.74878400
C	3.84680800	-2.45699300	0.76160700
C	3.82106900	-1.08560500	0.50621400
C	2.45676800	0.99433600	-0.04085100
C	0.98400200	2.72087300	-0.57009400
C	2.00954800	3.66459900	-0.58884900
C	3.30780900	3.23250500	-0.31719700
C	3.53584300	1.88477100	-0.03883300
N	1.20129000	1.42487700	-0.30750900
N	1.44466700	-1.17453500	0.23841300
Cu	-0.22586500	-0.05610100	-0.17111200
S	-1.74980900	-1.57582500	-1.31544300
O	-1.88673100	-2.71543200	-0.32333300
O	-2.99910500	-1.34352500	-2.13979200
C	-2.18281700	0.14841700	0.12235300
C	-2.72209700	1.34307000	-0.38561600
C	-2.57282000	-0.30466500	1.38916600
C	-3.60308900	2.10030300	0.39061900
C	-3.45198400	0.45475500	2.17154000
C	-3.96485900	1.65656800	1.67108500
H	0.50460600	-2.99124400	0.44440800
H	2.63237800	-4.24540500	0.94051800
H	4.79036900	-2.95236800	0.96549700
H	4.74448600	-0.52061900	0.51005600
H	-0.04702800	2.99634900	-0.76720100
H	1.79014000	4.70324500	-0.80821200
H	4.13589800	3.93352700	-0.31888300
H	4.54004500	1.54309100	0.17739400
H	-2.45264900	1.67715500	-1.38451600
H	-2.20483200	-1.26585100	1.74179500
H	-4.01076500	3.02983000	0.00194800
H	-3.74528800	0.10573800	3.15815400
H	-4.65643000	2.24196700	2.27045300

**E(RB3LYP)**	-2916.12840764	a.u.
**ν**	-179.07	cm^{-1}

3.2.2.3. Ligand: 4,4’-Dimethoxy-2,2,’-bipyridine (4,4’-diMeObpy)

(4,4’-Dimethoxy-2,2’-bipyridyl) phenylcopper(I) sulfur dioxide complex (**13b**)

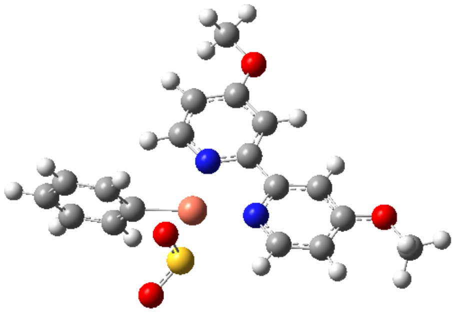

Geometry-optimised cartesian coordinates
Charge = 0 Multiplicity = 1

ATOM	X	Y	Z
C	-2.02730000	-0.03226500	0.02900700
C	-1.72062900	-2.32979200	0.07554500
C	-3.08579600	-2.58162600	0.13528500
C	-3.95493900	-1.48175900	0.14163500
C	-3.40423600	-0.19012300	0.08735700
C	-1.36841000	1.30071700	-0.02615800
C	0.66514800	2.41567500	-0.04497900
C	0.05562400	3.66411800	-0.11809400
C	-1.34631700	3.70767800	-0.14836700
C	-2.06414900	2.49993200	-0.10211300

N	-0.01502500	1.26481300	-0.00012600
N	-1.18542200	-1.10298100	0.02438600
Cu	0.80782000	-0.65692900	0.01968900
S	1.58028500	-2.88229700	-0.29803500
O	2.13071000	-2.86403600	-1.70436100
O	2.57244600	-3.34765700	0.73653700
C	2.68409400	-0.23510900	0.16463100
C	3.14613500	0.26700200	1.39317700
C	3.54706200	-0.20300700	-0.93681300
C	4.44128700	0.78951700	1.51450300
C	4.83926100	0.32932000	-0.82628200
C	5.29039800	0.82549400	0.40225300
H	-1.01600000	-3.15477700	0.06474000
H	-3.43599900	-3.60401600	0.17441200
H	-4.07719500	0.65722000	0.09800600
H	1.74576000	2.32352300	-0.01822500
H	0.66746500	4.55544600	-0.15101400
H	-3.14526300	2.54192600	-0.13158000
H	2.49662600	0.25945100	2.26593700
H	3.21927300	-0.62524800	-1.88445600
H	4.78376500	1.16899700	2.47446700
H	5.49316800	0.35030800	-1.69512800
H	6.29380800	1.23264600	0.49321100
O	-5.29494700	-1.55315800	0.19683700
O	-2.08405100	4.82993100	-0.22095700
C	-1.41352100	6.10274700	-0.27241400
H	-0.78285800	6.17186900	-1.16421700
H	-2.20765900	6.84604500	-0.32297500
H	-0.81374000	6.26073700	0.62933700
C	-5.92257900	-2.84846200	0.25321800
H	-6.99248100	-2.64952900	0.28751900
H	-5.68144200	-3.43322500	-0.63980700
H	-5.61548500	-3.38748900	1.15475500

E(RB3LYP) -3145.21971560 a.u.

<u>(4,4’-Dimethoxy-2,2’-bipyridyl) phenylcopper(I) sulfur dioxide complex migratory insertion transition state‡</u>

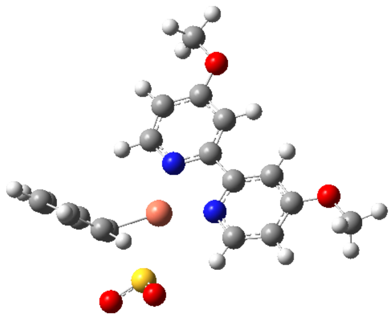

Geometry-optimised cartesian coordinates

Charge = 0 Multiplicity = 1

ATOM	X	Y	Z
C	1.90144600	-0.55036300	0.06356800
C	0.80385000	-2.59674700	0.15281400
C	1.99308200	-3.30357100	0.30115300
C	3.19001200	-2.57273900	0.32726700
C	3.13455600	-1.17389800	0.20396600
C	1.74492800	0.92963500	-0.07151000
C	0.25697500	2.68549700	-0.37002900
C	1.26773000	3.64083800	-0.33059100
C	2.58366300	3.19110200	-0.14841200
C	2.81639000	1.81166000	-0.01592600
N	0.47251200	1.36985000	-0.24928800
N	0.74713600	-1.26316200	0.03973200
Cu	-0.93747400	-0.12881500	-0.19908400
S	-2.50773500	-1.58102000	-1.37702600
O	-2.60342800	-2.77581700	-0.44525000
O	-3.79363000	-1.30626600	-2.13142400
C	-2.88277400	0.05771000	0.16507900
C	-3.44110100	1.27643800	-0.25821200

C	-3.22350100	-0.46168200	1.42070900
C	-4.29043200	1.99072500	0.59046900
C	-4.07050500	0.25490300	2.27579500
C	-4.60162900	1.48059000	1.85957000
H	-0.15387900	-3.10803300	0.11762400
H	1.96595000	-4.38135600	0.38824200
H	4.06376600	-0.61923800	0.21860700
H	-0.77814400	2.98431600	-0.50206000
H	1.01797700	4.68783300	-0.43684300
H	3.83417800	1.47421400	0.13143700
H	-3.21118200	1.66243300	-1.24820200
H	-2.84291700	-1.43963200	1.70770400
H	-4.71255800	2.93870500	0.26718600
H	-4.32501600	-0.14558000	3.25362900
H	-5.26840100	2.03321300	2.51551300
O	4.41900300	-3.10537000	0.46139200
O	3.67014900	3.98264800	-0.08445100
C	3.50187500	5.40678700	-0.20756300
H	2.86975700	5.79267300	0.59834400
H	4.50381200	5.82492800	-0.12370200
H	3.07291800	5.66327500	-1.18133700
C	4.54797700	-4.53295300	0.59063900
H	5.61652900	-4.71959200	0.68650700
H	4.02708100	-4.88949800	1.48485400
H	4.16109200	-5.03866200	-0.29961600

E(RB3LYP) -3145.20801417 a.u.
ν -179.90 cm^{-1}

3.2.2.4. Ligand: 4,4'-Dinitro-2,2,'-bipyridine (4,4'-diNO$_2$bpy)

(4,4'-Dinitro-2,2'-bipyridyl) phenylcopper(I) sulfur dioxide complex (**13c**)

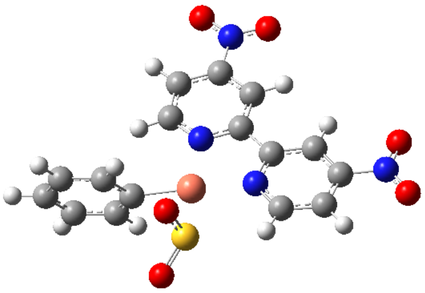

Geometry-optimised cartesian coordinates
Charge = 0 Multiplicity = 1

ATOM	X	Y	Z
C	-1.73169400	-0.41799800	0.03165600
C	-1.06148400	-2.64648500	0.06160700
C	-2.37563700	-3.10738500	0.12159100
C	-3.37179300	-2.14329500	0.13661200
C	-3.07690700	-0.78762100	0.09232700
C	-1.29242800	1.00057700	-0.01778600
C	0.55202200	2.42088300	-0.02255100
C	-0.25028400	3.56010300	-0.09957100
C	-1.62235100	3.35260700	-0.13665600
C	-2.17709600	2.07934300	-0.09746600
N	0.04089400	1.18761000	0.01626100
N	-0.74459900	-1.34687000	0.01923600
Cu	1.18589900	-0.61105300	0.02248200
S	2.31319900	-2.69505600	-0.33059500
O	2.83918300	-2.57056000	-1.73520300
O	3.36202000	-3.01981200	0.69361700
C	2.97159300	0.10257800	0.17901800
C	3.32843900	0.68387300	1.40819600
C	3.83118300	0.26507900	-0.91323400
C	4.51858500	1.41327400	1.53753900
C	5.01683300	1.00328700	-0.79373400
C	5.36421500	1.57717800	0.43446600
H	-0.23217100	-3.34407300	0.04355500
H	-2.60018000	-4.16483000	0.15501400

H	-3.87755600	-0.06220700	0.11131100
H	1.63346900	2.48876700	0.01208100
H	0.17743700	4.55310400	-0.12996500
H	-3.25021300	1.95795800	-0.13533200
H	2.67844800	0.57730300	2.27395500
H	3.58904700	-0.21185200	-1.86073200
H	4.78177400	1.85257000	2.49684100
H	5.66946200	1.12375300	-1.65521000
H	6.28622100	2.14376600	0.53229900
N	-2.52963800	4.52237300	-0.22368100
N	-4.79200300	-2.56470200	0.20125800
O	-3.74046000	4.30740800	-0.22813500
O	-5.64863600	-1.68286900	0.21406900
O	-2.01523500	5.63703100	-0.28569800
O	-5.02434100	-3.77090900	0.23761600

E(RB3LYP) -3325.15005759 a.u.

<u>(4,4′-Dinitro-2,2′-bipyridyl) phenylcopper(I) sulfur dioxide complex migratory insertion transition state‡</u>

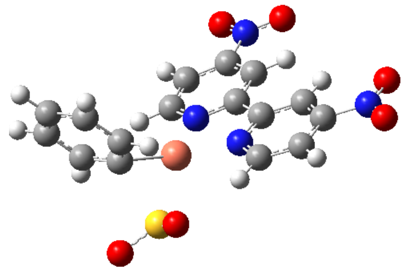

Geometry-optimised cartesian coordinates

Charge = 0 Multiplicity = 1

**ATOM**	**X**	**Y**	**Z**
C	1.49936600	-0.72576000	-0.02527100
C	0.10497500	-2.59541700	-0.08793700
C	1.17008800	-3.47022000	0.12542100
C	2.42820200	-2.89840100	0.26413500
C	2.62511400	-1.52496400	0.19076100
C	1.56341000	0.75863000	-0.13562500
C	0.35596600	2.72056200	-0.50200000
C	1.49516100	3.51580100	-0.39467700
C	2.69153600	2.85781500	-0.14141900
C	2.75467500	1.47727500	-0.00599600
N	0.38841100	1.38646400	-0.37807200
N	0.27010800	-1.26870900	-0.15542400
Cu	-1.23857400	0.15275200	-0.40430000
S	-2.94278600	-0.93563600	-1.61299500
O	-3.09763300	-2.30330500	-0.98239100
O	-4.21051900	-0.39162500	-2.22600700
C	-3.10792200	0.30232500	0.31720200
C	-3.66837800	1.58704600	0.23727700
C	-3.33185600	-0.48537600	1.45309200
C	-4.40585900	2.09709600	1.30989900
C	-4.06872500	0.02317800	2.52919400
C	-4.60261600	1.31510300	2.45643300
H	-0.91308800	-2.94994200	-0.21563900
H	1.02133700	-4.54018700	0.17994600
H	3.62059400	-1.11879800	0.29833300
H	-0.61569300	3.16119400	-0.69064900
H	1.44558300	4.59095500	-0.50104700
H	3.70401700	1.00030900	0.19137100
H	-3.53205700	2.18271300	-0.66163000
H	-2.95033900	-1.50364800	1.47704500
H	-4.83110700	3.09555000	1.25244800
H	-4.23534100	-0.58688500	3.41297000
H	-5.18392100	1.70826200	3.28566700
N	3.93854100	3.64560800	-0.01063100
N	3.60057100	-3.77383400	0.49473100
O	4.98095000	3.03376900	0.21855700
O	4.70569400	-3.24030100	0.58303400
O	3.85654900	4.86583400	-0.13952700

O	3.39883200	-4.98362400	0.58466500

E(RB3LYP) -3325.14123770 a.u.
ν -181.89 cm^{-1}

3.3. Oxidative addition of copper(I) into the copper–carbon bond

3.3.1. Oxidative addition of 4-iodotoluene to (L)Cu(SO_2Ph): Variation of $\Delta E_{OA}^{‡}$ with complex ligand

3.3.1.1. Geometry optimisations of common species to all pathways

Geometry optimisations, followed by a frequency calculation were carried out for each species. The geometry-optimised molecular coordinates (Å) and energies (B3LYP/6-31G+(d,p) for C, H, N, O, S, Cu & SDD/ ECP46MWB for I) are shown below (solvent = *N,N*-dimethylformamide).

4-Iodotoluene (**17b**)

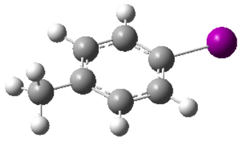

Geometry-optimised cartesian coordinates
Charge = 0 Multiplicity = 1

ATOM	X	Y	Z
C	-2.99041000	0.00014200	-0.01128200
C	-2.26787400	-1.20245100	-0.01104000
C	-0.86885300	-1.21463800	-0.00530000
C	-0.18196800	0.00009000	-0.00152900
C	-0.86876700	1.21477000	-0.00530700
C	-2.26788100	1.20263600	-0.01103200
H	-2.80034700	-2.15022700	-0.01729900
H	-0.33510400	-2.15875500	-0.00697900
H	-0.33503600	2.15889700	-0.00698200
H	-2.80025000	2.15046300	-0.01726300
C	-4.50086000	-0.00014700	0.01700000
H	-4.90933800	-0.88430800	-0.48201200
H	-4.86873300	-0.00721200	1.05075900
H	-4.90957400	0.89040700	-0.47022900
I	1.97430300	-0.00003200	0.00228200

E(RB3LYP) -282.40553667 a.u.

3.3.1.2. Ligand: None

Copper(I) phenylsulfinate (**14a**)

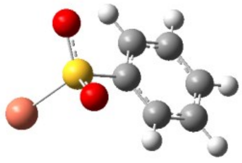

Geometry-optimised cartesian coordinates
Charge = 0 Multiplicity = 1

ATOM	X	Y	Z
Cu	2.06110000	-1.06983200	0.21878000
S	0.89811600	0.76872100	-0.15210700
C	-0.85596800	0.28478700	-0.05743100
C	-1.52161000	0.35872100	1.16852700
C	-2.85574600	-0.05243800	1.24195200
C	-3.50478100	-0.53691200	0.10059200
C	-2.82397400	-0.60971200	-1.12022800
C	-1.49018100	-0.20044500	-1.20398300
O	1.08480800	1.79126800	0.94459400
O	1.08850600	1.28257300	-1.56059200
H	-1.00563200	0.74319700	2.04199200
H	-3.38696400	0.00810600	2.18704400

H	-4.54089600	-0.85599200	0.16186400
H	-3.33047900	-0.98100400	-2.00602300
H	-0.95075200	-0.24344900	-2.14439200

E(RB3LYP) -2420.64481194 a.u.

Copper(I) phenylsulfinate-4-iodotoluene cation-π complex (**15a**)

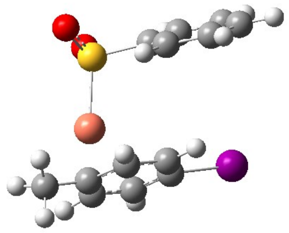

Geometry-optimised cartesian coordinates

Charge = 0 Multiplicity = 1

ATOM	X	Y	Z
Cu	-1.80689400	-0.11060700	-0.69566700
S	-1.65305800	2.09616700	-0.49109000
C	-0.01143100	2.31519200	0.27993900
C	1.02943200	2.90711900	-0.43608300
C	2.28817400	3.02486300	0.16449100
C	2.49692700	2.53800700	1.45882500
C	1.44710400	1.93367600	2.16219600
C	0.18613900	1.82096600	1.57334100
O	-1.57456200	2.85080600	-1.80264000
O	-2.63658200	2.67191900	0.51101600
H	0.84817100	3.26854000	-1.44302200
H	3.10451900	3.48863700	-0.38158000
H	3.47762400	2.62194100	1.91783100
H	1.61113600	1.54984900	3.16478100
H	-0.63316300	1.35129200	2.11003200
C	0.05531600	-1.83417700	1.40103800
C	0.43246800	-1.75692500	0.05955700
C	-0.51652600	-1.95317600	-0.95796100
C	-1.86459900	-2.22512300	-0.60703800
C	-2.26509400	-2.27587400	0.75355300
C	-1.28185800	-2.08022000	1.73589500
H	0.78442100	-1.68044100	2.18828700
H	-0.22201000	-1.96187100	-2.00167800
H	-2.57155500	-2.49691500	-1.38876500
H	-1.56153100	-2.11740000	2.78459300
I	2.45328000	-1.30846400	-0.46256700
C	-3.68949000	-2.59913700	1.12331900
H	-4.37957900	-2.33340700	0.31883200
H	-3.79219900	-3.67427500	1.31305100
H	-3.99102700	-2.07137800	2.03201800

E(RB3LYP) -2703.08186042 a.u.

Copper(I) phenylsulfinate-4-iodotoluene oxidative addition transition state‡

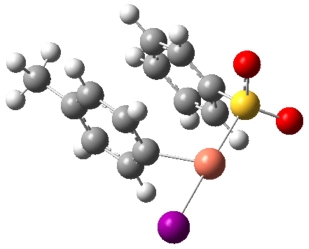

Geometry-optimised cartesian coordinates

Charge = 0 Multiplicity = 1

ATOM	X	Y	Z
Cu	-1.15791000	-0.90202000	0.12584600
S	0.64963700	-2.19435400	0.59712900
C	-0.41700700	0.92771300	0.13968600

I	-3.16915300	0.43924000	-0.34093700
C	2.11833200	-1.35238400	-0.05984100
C	0.22051500	1.32871000	-1.02585300
C	1.24692600	2.26966100	-0.88855700
C	1.57371500	2.82130200	0.36036000
C	0.84268900	2.41817900	1.48812600
C	-0.19379800	1.47941300	1.39166400
C	2.87877100	-0.54339600	0.78628500
C	3.96707300	0.15521000	0.25664300
C	4.27497500	0.04914900	-1.10364500
C	3.49973900	-0.76151400	-1.94155300
C	2.41065800	-1.46554800	-1.42181200
O	0.87973200	-2.30740000	2.08454300
O	0.54435800	-3.49795600	-0.16232500
C	2.70951700	3.80633000	0.48591300
H	-0.02870300	0.90860100	-1.99231000
H	1.79711100	2.57049100	-1.77571600
H	1.07295800	2.83727700	2.46392800
H	-0.76042700	1.17811400	2.26415100
H	2.62025500	-0.46591000	1.83645700
H	4.56783900	0.78594900	0.90481200
H	5.11643400	0.60080000	-1.51231600
H	3.74061100	-0.84329700	-2.99720400
H	1.79954900	-2.09678000	-2.05877900
H	2.77141800	4.45821800	-0.39047400
H	2.60429200	4.43027900	1.37757100
H	3.66365700	3.27064300	0.56371900

E(RB3LYP) -2703.05852945 a.u.
ν -93.00 cm^{-1}

<u>*p*-Tolyl(*S*-sulfinylphenyl)copper(III) iodide (**16a**)</u>

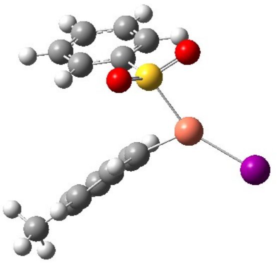

Geometry-optimised cartesian coordinates
Charge = 0 Multiplicity = 1

**ATOM**	**X**	**Y**	**Z**
Cu	1.06977100	0.29385700	0.29697500
S	-0.58471500	1.44215500	1.21806900
C	-0.28920500	-1.06619000	0.23387500
I	3.40207200	-0.05726900	-0.51904000
C	-2.01436300	1.63909000	0.15536000
C	-0.86136100	-1.29415900	-1.01307800
C	-1.62390200	-2.45665800	-1.16982500
C	-1.83758400	-3.34082100	-0.09813900
C	-1.26512700	-3.04099400	1.14663100
C	-0.49716800	-1.88193200	1.33429800
C	-3.17467200	0.92349400	0.45992800
C	-4.26368900	1.03653900	-0.40558800
C	-4.17117400	1.83604400	-1.55149100
C	-2.99274800	2.53398800	-1.84039000
C	-1.89280200	2.43596900	-0.98519900
O	-1.03549000	1.00341900	2.55860100
O	0.27353000	2.66533200	1.09354200
C	-2.69711600	-4.56884900	-0.28608600
H	-0.72124100	-0.61059200	-1.84371400
H	-2.06265900	-2.66833600	-2.14164800
H	-1.42452300	-3.70601300	1.99121200
H	-0.09140000	-1.63575800	2.30963400
H	-3.22067400	0.30051500	1.34595400
H	-5.17975800	0.49730300	-0.18699200
H	-5.02052400	1.91364900	-2.22317000

H	-2.92727300	3.15297300	-2.72934800
H	-0.96976200	2.96586000	-1.19266500
H	-2.41294900	-5.11276400	-1.19292900
H	-2.61338800	-5.25058300	0.56433900
H	-3.75240900	-4.29044200	-0.39182400

E(RB3LYP) -2703.06559220 a.u.

3.3.1.3. Ligand: 2,2'-Bipyridine (bpy)

(2,2'-Bipyridyl)copper(I) phenylsulfinate (**14b**)

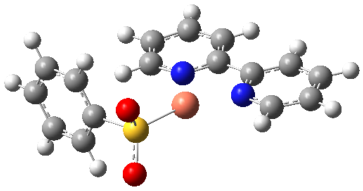

Geometry-optimised cartesian coordinates

Charge = 0 Multiplicity = 1

**ATOM**	**X**	**Y**	**Z**
C	2.90866600	0.38754400	-0.00446800
C	3.15691500	-1.93082000	-0.01690100
C	4.54724600	-1.83705600	-0.02483900
C	5.12310600	-0.56710700	-0.02217000
C	4.29691200	0.55653700	-0.01192100
C	1.94644900	1.52792500	0.00623800
C	-0.29852300	2.14599900	0.01773500
C	0.01942400	3.50367300	0.02606800
C	1.36609300	3.86626500	0.02428900
C	2.34272300	2.86974500	0.01406500
N	0.63795800	1.18740600	0.00833900
N	2.35882100	-0.85159100	-0.00694500
Cu	0.33813100	-0.88380300	0.00524800
S	-1.58580400	-1.94365100	0.02075700
O	-1.89842600	-2.73577000	-1.24175000
O	-1.89580700	-2.68375800	1.31506100
C	-2.79303400	-0.56652300	-0.00606200
C	-3.21376500	0.00157600	1.19939500
C	-4.03330100	1.13457100	1.17539400
C	-4.41959800	1.69545800	-0.04747800
C	-3.99311500	1.11898500	-1.24981400
C	-3.17407000	-0.01418000	-1.23221600
H	2.65428500	-2.89136400	-0.01846700
H	5.15209400	-2.73641100	-0.03283600
H	6.20140900	-0.44782800	-0.02810000
H	4.73633600	1.54563200	-0.01003900
H	-1.32805100	1.80464500	0.01863000
H	-0.76962900	4.24697800	0.03377900
H	1.65802400	4.91124600	0.03081800
H	3.38980200	3.14471600	0.01277200
H	-2.90078700	-0.44151900	2.13923100
H	-4.36624000	1.57973700	2.10839900
H	-5.05022400	2.57935300	-0.06363200
H	-4.29495400	1.55226500	-2.19885200
H	-2.83136400	-0.46878400	-2.15615700

E(RB3LYP) -2916.16084963 a.u.

(2,2'-Bipyridyl)copper(I) phenylsulfinate-4-iodotoluene cation-π complex (**15b**)

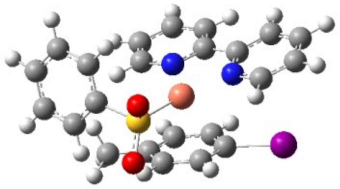

Geometry-optimised cartesian coordinates

Charge = 0 Multiplicity = 1

**ATOM**	**X**	**Y**	**Z**
C	1.58171400	-0.86338500	1.82551500
C	2.05435500	-2.64667100	0.40258800
C	3.24030400	-2.93919800	1.07363900
C	3.59358700	-2.14763100	2.16580000
C	2.75749500	-1.09778800	2.54542300
C	0.61508200	0.21760900	2.17148400
C	-1.48667700	1.11815600	1.74328000
C	-1.32281700	2.10508600	2.71431000
C	-0.12217700	2.13945800	3.42340200
C	0.85972800	1.18601300	3.15146500
N	-0.54460900	0.20460700	1.47798300
N	1.24567100	-1.64081200	0.76771600
Cu	-0.52818200	-1.17167500	-0.08972700
S	-2.24979400	-1.79688000	-1.32099100
O	-2.29099900	-1.29292200	-2.75718600
O	-2.62619000	-3.26703200	-1.17246000
C	-3.61479400	-0.89816500	-0.49308100
C	-4.04048800	-1.33556100	0.76620400
C	-4.98944000	-0.58893300	1.46890200
C	-5.50429700	0.59086000	0.91509300
C	-5.07420400	1.01919200	-0.34524400
C	-4.12318200	0.27499100	-1.05304100
H	1.73214600	-3.22504900	-0.45609300
H	3.86241600	-3.76303300	0.74281500
H	4.50752300	-2.34107800	2.71783500
H	3.01986700	-0.48166900	3.39618800
H	-2.39560800	1.04451600	1.15625600
H	-2.11231700	2.82562800	2.89593500
H	0.05306100	2.89863400	4.17875800
H	1.79779800	1.20957300	3.69169400
H	-3.62626900	-2.24489400	1.19137900
H	-5.32324800	-0.92256000	2.44719100
H	-6.23562200	1.17455700	1.46642500
H	-5.47114900	1.93424300	-0.77524500
H	-3.77332800	0.59547100	-2.02819400
C	0.69427300	0.79636000	-1.86750100
C	1.72269300	1.36402700	-1.11239800
C	1.50616500	2.52234200	-0.36576000
C	0.24850700	3.13194800	-0.40655700
C	-0.79720800	2.60565000	-1.17871600
C	-0.55752500	1.42637500	-1.89744900
H	0.85446300	-0.10887300	-2.44339500
H	2.29866300	2.95519100	0.23467000
H	0.08505800	4.03491500	0.17576100
H	-1.34676000	0.96476600	-2.48231600
C	-2.14972900	3.27554500	-1.20399800
H	-2.05675700	4.36152900	-1.10817600
H	-2.68753700	3.05485600	-2.13024500
H	-2.77484900	2.92445800	-0.37467500
I	3.67525100	0.46854500	-1.12644400

E(RB3LYP) -3198.56928085 a.u.

<u>(2,2′-Bipyridyl)copper(I) phenylsulfinate-4-iodotoluene oxidative addition transition state‡</u>

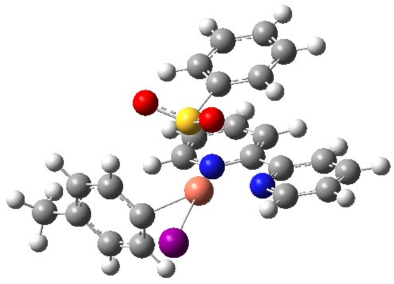

Geometry-optimised cartesian coordinates

Charge = 0 Multiplicity = 1

**ATOM**	**X**	**Y**	**Z**
C	2.08289300	-0.53489800	-1.59638000
C	1.42610800	-2.70024600	-1.03886900
C	2.61364300	-3.24310900	-1.52427200
C	3.56063400	-2.37649500	-2.07325900
C	3.29427600	-1.00843300	-2.11083900
C	1.71344100	0.90375900	-1.55182400
C	0.07739500	2.44025000	-0.92922700
C	0.85883500	3.51911300	-1.33812300
C	2.11977700	3.25563300	-1.87368600
C	2.55506900	1.93507800	-1.98064000
N	0.49187700	1.17275700	-1.03819800
N	1.16833100	-1.38671900	-1.07843200
Cu	-0.50846500	-0.48942400	-0.28462900
S	0.54473600	-0.48413100	2.06316800
C	2.11991100	0.42254300	1.83026300
C	2.11614600	1.82049600	1.87073600
C	3.27962700	-0.27791700	1.49043000
C	3.28835100	2.52161700	1.57697800
C	4.45161300	0.42792000	1.19865900
C	4.45599100	1.82632500	1.23836400
O	0.99343100	-1.87219300	2.52543400
O	-0.17377000	0.31667000	3.15538400
H	0.64979200	-3.31987700	-0.60121700
H	2.78458300	-4.31228100	-1.47116100
H	4.49808700	-2.75803500	-2.46458100
H	4.02507000	-0.32725600	-2.52783500
H	-0.90629600	2.58070400	-0.49604300
H	0.48671000	4.53166600	-1.23072200
H	2.76394300	4.06674900	-2.19696300
H	3.53791700	1.72167300	-2.38096400
H	1.20339700	2.34794200	2.12943100
H	3.25723000	-1.36244400	1.45582400
H	3.28907400	3.60756800	1.60268100
H	5.35527500	-0.11329200	0.93263700
H	5.36373100	2.37376900	1.00126600
I	-2.35725600	-2.26862300	0.00454700
C	-2.39036700	0.21088400	-0.12284000
C	-2.60476200	0.90246700	1.07243700
C	-2.89141300	0.67607200	-1.34432600
C	-3.26940500	2.13176200	1.01528500
H	-2.21754000	0.52524900	2.01228500
C	-3.55080300	1.90757200	-1.36974700
H	-2.75333400	0.10420200	-2.25444200
C	-3.75327000	2.65278100	-0.19621000
H	-3.41260300	2.69048600	1.93679000
H	-3.91932700	2.28584900	-2.31981400
C	-4.44722500	3.99255900	-0.24128400
H	-5.18114600	4.03530700	-1.05153000
H	-4.95802400	4.21008800	0.70130000
H	-3.71980900	4.79587600	-0.41427600

**E(RB3LYP)**	-3198.54898568	a.u.
**ν**	-77.74	cm^{-1}

(2,2'-Bipyridyl) *p*-tolyl(*S*-sulfinylphenyl)copper(III) iodide (**16b**)

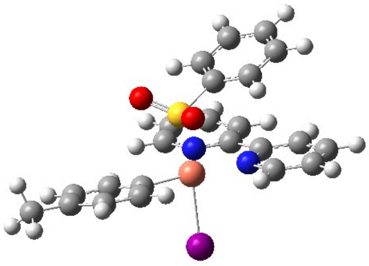

Geometry-optimised cartesian coordinates

Charge = 0 Multiplicity = 1

ATOM	X	Y	Z
C	2.27495800	1.14242700	-0.10055500
C	1.39411300	1.11205300	-2.26448000
C	2.57217700	1.60741800	-2.81309400
C	3.63656100	1.87899000	-1.95218100
C	3.48703300	1.64604000	-0.58678800
C	2.02990100	0.84389700	1.33505800
C	0.54088200	-0.00984700	2.89176900
C	1.44508000	0.16376100	3.93967800
C	2.69329400	0.71402700	3.64719700
C	2.99215600	1.05848100	2.32967800
N	0.81910700	0.32416100	1.62672200
N	1.25498000	0.88432000	-0.94933400
Cu	-0.51320900	0.21502000	-0.23796400
S	-0.01867000	-2.00937600	-0.68852500
C	1.78401600	-2.15654100	-0.48995800
C	2.30412500	-2.41316100	0.78038300
C	2.61223800	-1.96331400	-1.59741700
C	3.69040700	-2.47158000	0.94292800
C	3.99689100	-2.02420400	-1.42314400
C	4.53505200	-2.27157400	-0.15490300
O	-0.29224900	-2.25051100	-2.14587500
O	-0.59622000	-2.98871300	0.28701800
H	0.53159700	0.88314300	-2.88040600
H	2.64602400	1.77094200	-3.88189900
H	4.57479700	2.26465600	-2.33707600
H	4.31005900	1.84964600	0.08556600
H	-0.43864300	-0.44001100	3.06964400
H	1.17317900	-0.12619500	4.94844000
H	3.42839000	0.86941800	4.43010600
H	3.96138300	1.47775800	2.09147600
H	1.63791900	-2.56037200	1.62274500
H	2.17843700	-1.76970400	-2.57173100
H	4.10765500	-2.66619900	1.92609600
H	4.65220400	-1.87277500	-2.27539700
H	5.61215900	-2.30812800	-0.02229600
I	-1.52448700	2.62608900	-0.15740600
C	-2.31842900	-0.45643900	-0.05514800
C	-3.04232100	-0.74675900	-1.20647700
C	-2.86089400	-0.65627700	1.20932600
C	-4.33385700	-1.27670400	-1.08140600
H	-2.61108800	-0.58692100	-2.18927700
C	-4.15245500	-1.18744400	1.31799600
H	-2.30974800	-0.39626600	2.10513100
C	-4.90619200	-1.50859900	0.17838800
H	-4.89786300	-1.51067300	-1.98155100
H	-4.57708900	-1.34318200	2.30720700
C	-6.28693700	-2.11139100	0.30649600
H	-6.90891900	-1.87328000	-0.56184200
H	-6.22998100	-3.20477400	0.37967500
H	-6.79849400	-1.75051900	1.20434400

E(RB3LYP) -3198.55386175 a.u.

3.3.1.4. Ligand: 4,4'-Dimethoxy-2,2'-bipyridine (4,4'-diMeObpy)

(4,4'-Dimethoxy-2,2'-bipyridyl)copper(I) phenylsulfinate (**14c**)

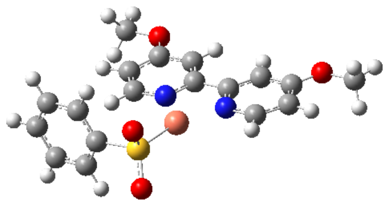

Geometry-optimised cartesian coordinates

Charge = 0 Multiplicity = 1

**ATOM**	**X**	**Y**	**Z**
C	-1.26307900	1.23240600	0.00326900
C	1.00129500	1.73289600	0.03369900
C	0.76865500	3.10487900	0.01384700
C	-0.56358300	3.54100700	-0.01704200
C	-1.59111500	2.58217100	-0.02223500
C	-2.28741500	0.14448500	0.00272300
C	-2.66655000	-2.14698500	-0.01932500
C	-4.04812100	-1.98892400	-0.00203600
C	-4.55587300	-0.68215800	0.02095200
C	-3.65291600	0.39435700	0.02422800
N	-1.79859900	-1.12466300	-0.01726900
N	0.02703800	0.81435400	0.02812300
Cu	0.21155000	-1.26395400	-0.01045400
S	2.07988600	-2.41450000	-0.04139700
C	3.34963900	-1.09450800	0.00486200
O	2.35529600	-3.23800600	1.21019600
O	2.36033200	-3.15066700	-1.34529900
C	3.78068400	-0.51624000	-1.19226400
C	4.64954800	0.57881400	-1.15252100
C	5.07517500	1.09257900	0.07809500
C	4.63837100	0.50631800	1.27189800
C	3.76976400	-0.58913800	1.23837700
O	-0.95644600	4.82825200	-0.04379000
C	0.04588000	5.86097700	-0.04301700
O	-5.86114800	-0.35909100	0.04153000
C	-6.83985000	-1.41495900	0.03946300
H	2.01564700	1.34920800	0.05468300
H	1.60692700	3.78836400	0.02096200
H	-2.61589600	2.92939400	-0.04978700
H	-2.22881400	-3.13876600	-0.03608600
H	-4.68499700	-2.86294100	-0.00576800
H	-4.05357900	1.39925700	0.04668100
H	3.43661900	-0.92185600	-2.13819600
H	4.99010300	1.03161700	-2.07909800
H	5.74422200	1.94744600	0.10681600
H	4.97050800	0.90286800	2.22688600
H	3.41847500	-1.05060500	2.15565800
H	-0.50536500	6.79968700	-0.07033000
H	0.65126500	5.81242700	0.86758800
H	0.68573800	5.78030700	-0.92740100
H	-7.80624400	-0.91355200	0.05782000
H	-6.75301400	-2.02103800	-0.86770900
H	-6.73112000	-2.04475200	0.92789100

E(RB3LYP) -3145.24078749 a.u.

(4,4'-Dimethoxy-2,2'-bipyridyl)copper(I) phenylsulfinate-4-iodotoluene cation-π complex (**15c**)

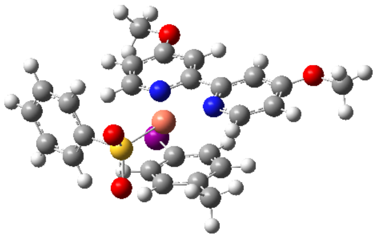

Geometry-optimised cartesian coordinates

Charge = 0 Multiplicity = 1

**ATOM**	**X**	**Y**	**Z**
C	0.98929800	-0.70624300	-1.36911100
C	-1.23850700	-0.07996900	-1.49826600
C	-1.62718600	-1.31258800	-2.01045800
C	-0.63502100	-2.28639700	-2.19291500
C	0.69371500	-1.96903800	-1.86875200
C	2.36919100	-0.28558500	-0.98516400
C	3.69710400	1.41346500	-0.12619400
C	4.85440300	0.64554200	-0.20713400

C	4.73811600	-0.65904200	-0.70853100
C	3.47152500	-1.12103700	-1.10433500
N	2.48483100	0.97726400	-0.49517100
N	0.02645900	0.22845000	-1.18587700
Cu	0.72677700	1.95710500	-0.27999200
S	-0.43992200	3.73937800	0.26293500
C	-2.11172200	3.27662300	-0.32568800
O	-0.62844900	3.97842000	1.75520300
O	-0.09980900	4.99641200	-0.52856300
C	-2.42302500	3.43306200	-1.68018500
C	-3.64254300	2.95132900	-2.16595300
C	-4.53879900	2.31040600	-1.30132700
C	-4.22068400	2.16175900	0.05368800
C	-3.00329500	2.64455000	0.54540900
O	-0.85064000	-3.53347700	-2.65360300
C	-2.19646800	-3.93155300	-2.96838200
O	5.75533400	-1.52842800	-0.84840800
C	7.08003600	-1.11649100	-0.46582300
H	-1.97436800	0.69684100	-1.32308200
H	-2.67055000	-1.49316000	-2.23047900
H	1.45157500	-2.72966600	-2.00498400
H	3.73839900	2.42609600	0.25935400
H	5.79732600	1.06705500	0.11433000
H	3.39016800	-2.12871800	-1.49072600
H	-1.71493700	3.92200500	-2.34192800
H	-3.89020100	3.07066700	-3.21677800
H	-5.48110100	1.92813300	-1.68269700
H	-4.91647900	1.66875200	0.72649100
H	-2.74473600	2.53819000	1.59325300
H	-2.12351700	-4.96695400	-3.29861200
H	-2.83354700	-3.86875300	-2.08050700
H	-2.60562100	-3.31330000	-3.77405300
H	7.72050700	-1.97340000	-0.66997800
H	7.40594200	-0.25873200	-1.06280300
H	7.11655700	-0.87146400	0.60043400
C	-1.23695900	-0.39286300	2.00035200
C	-0.71341600	-1.66491100	1.76838500
C	0.65593100	-1.91028900	1.87954300
C	1.50633100	-0.86356000	2.25013900
C	1.01038900	0.42124400	2.50917500
C	-0.36936300	0.64023000	2.36812800
H	-2.29712000	-0.19502800	1.88649200
H	1.06434800	-2.89431600	1.67723000
H	2.57386500	-1.05414600	2.32299600
H	-0.76149400	1.63979500	2.52907400
I	-2.03577000	-3.28210700	1.24893600
C	1.91996000	1.54855000	2.93622100
H	1.63022900	2.48513600	2.44846800
H	1.85477100	1.71019300	4.01912200
H	2.96342000	1.33374700	2.69197900

E(RB3LYP) -3427.64789041 a.u.

(4,4'-Dimethoxy-2,2'-bipyridyl)copper(I) phenylsulfinate-4-iodotoluene oxidative addition transition state‡

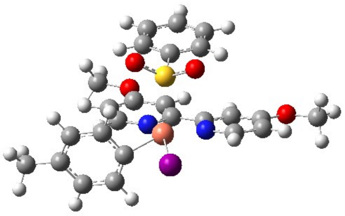

Geometry-optimised cartesian coordinates

Charge = 0 Multiplicity = 1

ATOM	X	Y	Z
C	1.94063400	-0.44266300	-1.06755400
N	1.02271200	-1.37984200	-0.72123500
C	1.39716100	-2.66270700	-0.66480200
C	2.68537500	-3.10129400	-0.95130900
C	3.63576400	-2.13816400	-1.32497800

C	3.24721400	-0.78985200	-1.38441000
Cu	-0.83054600	-0.64442100	-0.20553300
N	0.12817000	1.10946300	-0.78658000
C	1.45041900	0.96276100	-1.05001500
C	2.27714400	2.06012000	-1.24751700
C	1.73495400	3.35301700	-1.16510300
C	0.36511100	3.49855300	-0.90292100
C	-0.38531800	2.34160300	-0.71930600
O	4.91932200	-2.39852100	-1.63526600
O	2.60217000	4.36883900	-1.33572800
C	2.11984100	5.71931500	-1.22379900
C	5.38434700	-3.75936200	-1.58852900
S	-0.21008200	-0.55245200	2.28374200
C	1.30347000	0.48284800	2.31178800
O	-1.16692600	0.18542700	3.22792500
O	0.26686700	-1.89773900	2.83805300
C	2.55490800	-0.11806900	2.15561600
C	3.69830400	0.68198900	2.06037900
C	3.58513600	2.07545900	2.11166600
C	2.32705500	2.67091300	2.26552400
C	1.18269000	1.87517200	2.36308700
H	0.62394600	-3.36687000	-0.37277500
H	2.91873400	-4.15513500	-0.88063300
H	3.98595200	-0.05077900	-1.66641200
H	3.33837900	1.95616500	-1.43170800
H	-0.11532000	4.46441500	-0.82365500
H	-1.44474900	2.40019500	-0.49646600
H	2.99093300	6.35311500	-1.38456500
H	1.36449400	5.92684900	-1.98862800
H	1.70773800	5.90106800	-0.22585600
H	6.43405000	-3.71711000	-1.87593600
H	5.29294500	-4.16546600	-0.57602900
H	4.83030100	-4.38283000	-2.29759800
H	2.62490000	-1.20003500	2.10631800
H	4.67268700	0.21795900	1.93556400
H	4.47201900	2.69649900	2.02427100
H	2.23702100	3.75313500	2.29702700
H	0.20139700	2.32516800	2.47480400
C	-2.79515500	-0.16100700	-0.38024100
C	-3.26334400	0.51477900	0.75177800
C	-3.08793900	0.28645400	-1.67512200
C	-3.97358100	1.70468800	0.56822200
H	-3.03327300	0.15617500	1.74849800
C	-3.79906100	1.47929200	-1.82674200
H	-2.75237100	-0.27068500	-2.54208300
C	-4.25579400	2.20486700	-0.71399500
H	-4.31354600	2.25002200	1.44513500
H	-4.00623500	1.84289400	-2.82994000
C	-5.00089200	3.50551700	-0.89078000
H	-4.29964500	4.34951900	-0.91261900
H	-5.56144100	3.52082200	-1.83011100
H	-5.69911400	3.68124400	-0.06713400
I	-2.56643300	-2.57727600	-0.21659700

E(RB3LYP) -3427.62660169 a.u.
ν -82.36 cm^{-1}

(4,4'-Dimethoxy-2,2'-bipyridyl) *p*-tolyl(*S*-sulfinylphenyl)copper(III) iodide (**16c**)

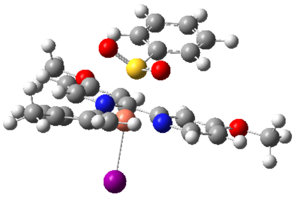

Geometry-optimised cartesian coordinates
Charge = 0 Multiplicity = 1

ATOM	X	Y	Z
C	2.27495800	1.14242700	-0.10055500
C	1.39411300	1.11205300	-2.26448000

C	2.57217700	1.60741800	-2.81309400
C	3.63656100	1.87899000	-1.95218100
C	3.48703300	1.64604000	-0.58678800
C	2.02990100	0.84389700	1.33505800
C	0.54088200	-0.00984700	2.89176900
C	1.44508000	0.16376100	3.93967800
C	2.69329400	0.71402700	3.64719700
C	2.99215600	1.05848100	2.32967800
N	0.81910700	0.32416100	1.62672200
N	1.25498000	0.88432000	-0.94933400
Cu	-0.51320900	0.21502000	-0.23796400
S	-0.01867000	-2.00937600	-0.68852500
C	1.78401600	-2.15654100	-0.48995800
C	2.30412500	-2.41316100	0.78038300
C	2.61223800	-1.96331400	-1.59741700
C	3.69040700	-2.47158000	0.94292800
C	3.99689100	-2.02420400	-1.42314400
C	4.53505200	-2.27157400	-0.15490300
O	-0.29224900	-2.25051100	-2.14587500
O	-0.59622000	-2.98871300	0.28701800
H	0.53159700	0.88314300	-2.88040600
H	2.64602400	1.77094200	-3.88189900
H	4.57479700	2.26465600	-2.33707600
H	4.31005900	1.84964600	0.08556600
H	-0.43864300	-0.44001100	3.06964400
H	1.17317900	-0.12619500	4.94844000
H	3.42839000	0.86941800	4.43010600
H	3.96138300	1.47775800	2.09147600
H	1.63791900	-2.56037200	1.62274500
H	2.17843700	-1.76970400	-2.57173100
H	4.10765500	-2.66619900	1.92609600
H	4.65220400	-1.87277500	-2.27539700
H	5.61215900	-2.30812800	-0.02229600
I	-1.52448700	2.62608900	-0.15740600
C	-2.31842900	-0.45643900	-0.05514800
C	-3.04232100	-0.74675900	-1.20647700
C	-2.86089400	-0.65627700	1.20932600
C	-4.33385700	-1.27670400	-1.08140600
H	-2.61108800	-0.58692100	-2.18927700
C	-4.15245500	-1.18744400	1.31799600
H	-2.30974800	-0.39626600	2.10513100
C	-4.90619200	-1.50859900	0.17838800
H	-4.89786300	-1.51067300	-1.98155100
H	-4.57708900	-1.34318200	2.30720700
C	-6.28693700	-2.11139100	0.30649600
H	-6.90891900	-1.87328000	-0.56184200
H	-6.22998100	-3.20477400	0.37967500
H	-6.79849400	-1.75051900	1.20434400

E(RB3LYP) -3427.63151675 a.u.

3.3.1.5. Ligand: 4,4'-Dinitro-2,2'-bipyridine (4,4'-diNO_2bpy)

(4,4'-Dinitro-2,2'-bipyridyl)copper(I) phenylsulfinate (**14d**)

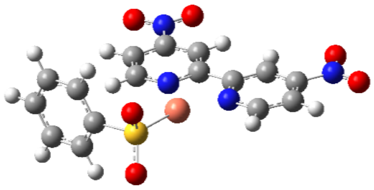

Geometry-optimised cartesian coordinates

Charge = 0 Multiplicity = 1

ATOM	X	Y	Z
C	-1.15293900	0.94172100	-0.00047000
N	0.17217000	0.68566600	0.00438400
C	1.05062900	1.69463600	0.00286200
C	0.65753900	3.03333300	-0.00421200
C	-0.70834800	3.27983100	-0.01005500
C	-1.64176300	2.25072000	-0.00814900

Cu	0.60724600	-1.38200500	-0.00542200
N	-1.41763600	-1.45663100	0.00067200
C	-2.04110900	-0.25515300	0.00253400
C	-3.43476300	-0.16261400	0.00759300
C	-4.15791500	-1.34817600	0.01070500
C	-3.53430500	-2.58770500	0.00933900
C	-2.13997900	-2.58550000	0.00414600
N	-1.19149700	4.68105100	-0.01878300
N	-5.63861500	-1.28119400	0.01564500
O	-6.16022300	-0.16748900	0.01680800
O	-0.34627500	5.57383400	-0.01897000
S	2.60832200	-2.30102900	-0.02981500
C	3.70434400	-0.83533300	0.00533500
O	2.97408700	-3.07455800	1.22785400
O	2.96541700	-3.00695400	-1.32894800
C	4.07601100	-0.22771200	-1.19712900
C	4.79987600	0.96855000	-1.16574600
C	5.13953100	1.55124700	0.06087800
C	4.76319000	0.93417200	1.25987400
C	4.03976000	-0.26226700	1.23543200
O	-6.25538300	-2.34454100	0.01823000
O	-2.40728400	4.86702900	-0.02549600
H	2.09927200	1.42127500	0.00725000
H	1.38319800	3.83541300	-0.00540500
H	-2.69728200	2.48223500	-0.01335100
H	-3.95509200	0.78443900	0.00975800
H	-4.09691900	-3.51138300	0.01197900
H	-1.58114600	-3.51331200	0.00242200
H	3.79898200	-0.68873900	-2.13958500
H	5.09425000	1.44583200	-2.09572400
H	5.69481900	2.48411300	0.08251000
H	5.02913600	1.38489500	2.21141300
H	3.73527500	-0.74947100	2.15613000

E(RB3LYP) -3325.17290925 a.u.

<u>(4,4′-Dinitro-2,2′-bipyridyl)copper(I) phenylsulfinate-4-iodotoluene cation-π complex (**15d**)</u>

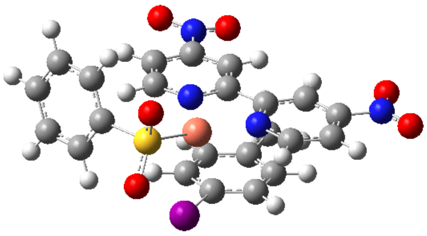

Geometry-optimised cartesian coordinates

Charge = 0 Multiplicity = 1

**ATOM**	**X**	**Y**	**Z**
C	-1.03132900	-0.68220600	1.08395800
N	0.30461900	-0.71506600	0.89198200
C	0.93737900	-1.88906800	0.78953200
C	0.27243500	-3.11260700	0.87526900
C	-1.10140800	-3.06121600	1.06594200
C	-1.78347400	-1.85619000	1.17325500
Cu	1.15739600	1.20675400	0.71399900
N	-0.78412900	1.71488600	1.05436900
C	-1.63938800	0.67299100	1.16652000
C	-3.01342700	0.87502400	1.32241500
C	-3.47240500	2.18433000	1.34549700
C	-2.60850400	3.26614900	1.22657700
C	-1.25420500	2.97083700	1.08318100
N	-1.87267200	-4.32213700	1.13148100
N	-4.92540800	2.42740200	1.48925400
O	-5.66657200	1.44721300	1.55398900
O	-1.24192500	-5.37781100	1.13037100
S	3.32161100	1.67061900	0.87782800
C	4.04348900	-0.00879700	0.79267000
O	3.90127400	2.38670500	-0.33479200
O	3.79974200	2.22003400	2.21479100

C	4.24056700	-0.73500900	1.97066400
C	4.65891100	-2.06777800	1.89378500
C	4.86753400	-2.66714200	0.64570600
C	4.66741700	-1.93136100	-0.52905900
C	4.25182300	-0.59772500	-0.45883900
O	-5.30561200	3.59635100	1.53149200
O	-3.10039000	-4.24029900	1.17345100
H	2.00754500	-1.84682500	0.62667100
H	0.80226300	-4.05142200	0.78524900
H	-2.85449300	-1.85546600	1.31486200
H	-3.71539900	0.05810400	1.40856500
H	-2.96554600	4.28714800	1.24261900
H	-0.51827300	3.75973800	0.98464900
H	4.06495900	-0.25867300	2.92987800
H	4.81579900	-2.63781800	2.80491700
H	5.18313500	-3.70470600	0.58848000
H	4.83091400	-2.39554500	-1.49733200
H	4.08351700	-0.01770100	-1.35999100
C	-0.76962100	-0.04912100	-2.27306100
C	-0.63424200	-1.43775600	-2.34479100
C	-1.77576700	-2.23454100	-2.23847800
C	-3.04963000	-1.67121600	-2.05355500
C	-3.15171000	-0.27461700	-2.00524800
C	-2.02090600	0.54399700	-2.11516100
H	0.33989100	-1.89772600	-2.46884500
H	-1.66871000	-3.31534600	-2.28494600
H	-4.12407800	0.19087200	-1.86853900
H	-2.12518500	1.62233700	-2.06409700
C	-4.26131200	-2.55630200	-1.88461400
H	-5.18860100	-1.97905700	-1.93077300
H	-4.29719300	-3.32956600	-2.65922200
H	-4.23030200	-3.07005300	-0.91612500
I	0.98994600	1.18186300	-2.33404200

E(RB3LYP) -3607.57521541 a.u.

(4,4’-Dinitro-2,2’-bipyridyl)copper(I) phenylsulfinate-4-iodotoluene oxidative addition transition state‡

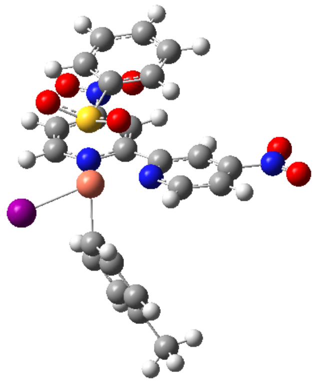

Geometry-optimised cartesian coordinates

Charge = 0 Multiplicity = 1

ATOM	X	Y	Z
C	1.07177200	1.23307000	0.16190500
N	-0.06218500	1.22579700	-0.57057600
C	-0.38353600	2.27726400	-1.33257200
C	0.41155600	3.41846700	-1.40898800
C	1.56893600	3.42105300	-0.64040500
C	1.92363300	2.34188600	0.15641000
Cu	-1.28289600	-0.45710300	-0.43559000
N	0.37676500	-0.91579600	0.98118500
C	1.36046300	0.00382200	0.94725400
C	2.58579400	-0.21336000	1.58451100
C	2.74227400	-1.41232400	2.26782500
C	1.73079600	-2.36168300	2.32929200
C	0.54938000	-2.05735700	1.65171900
N	2.45556800	4.60800100	-0.66964100
N	4.03832800	-1.69060500	2.92685800
O	4.14866900	-2.74886100	3.54386100

O	3.48124300	4.57285900	0.00845000
S	-0.22993400	-1.42141000	-2.35840600
C	1.57616300	-1.35761700	-2.08299300
I	-3.18268300	-2.05227300	0.05643600
C	-2.64676300	0.49267600	0.65944000
C	-3.43770100	1.36587800	-0.08165700
C	-3.82092200	2.57677100	0.51170700
C	-3.46605300	2.88493500	1.83242200
C	-2.71670500	1.94294300	2.56123900
C	-2.31606800	0.73201000	1.99237100
O	-0.51317200	-2.88819400	-2.62461700
O	-0.46755200	-0.49655300	-3.54310900
C	2.19805000	-2.40517500	-1.39607000
C	3.55323300	-2.30255400	-1.07199100
C	4.27678000	-1.15806100	-1.43060200
C	3.64861100	-0.12098000	-2.12861500
C	2.29223300	-0.21783500	-2.45607700
C	-3.87181200	4.19342300	2.46801800
O	4.93038000	-0.85074900	2.81303900
O	2.11156900	5.55700000	-1.37170500
H	-1.30454600	2.19858000	-1.89717900
H	0.13799400	4.25754200	-2.03432400
H	2.82967700	2.37996000	0.74394400
H	3.40150200	0.49409300	1.54456900
H	1.85654100	-3.29551100	2.86072900
H	-0.28392500	-2.75233100	1.63412200
H	-3.73519200	1.13041800	-1.09765000
H	-4.41476500	3.27895900	-0.06778800
H	-2.43965200	2.15666300	3.59078100
H	-1.73351800	0.01699500	2.56078200
H	1.62515300	-3.28450800	-1.12007100
H	4.04150600	-3.10893200	-0.53255000
H	5.32639800	-1.07574000	-1.16467900
H	4.20925600	0.76530100	-2.41086400
H	1.78902700	0.57947000	-2.99324600
H	-3.00500600	4.85703200	2.57437800
H	-4.62107100	4.71583600	1.86714600
H	-4.28338900	4.03587400	3.47044100

**E(RB3LYP)**	-3607.55150263	a.u.
**ν**	-76.26	cm^{-1}

(4,4′-Dinitro-2,2′-bipyridyl) *p*-tolyl(*S*-sulfinylphenyl)copper(III) iodide (**16d**)

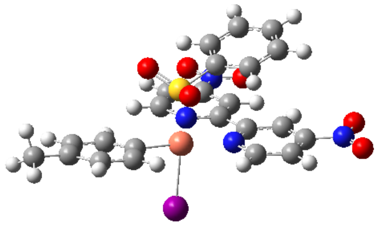

Geometry-optimised cartesian coordinates

Charge = 0 Multiplicity = 1

ATOM	X	Y	Z
C	1.78312300	-0.40862100	-0.55402300
N	0.67776900	-1.17973200	-0.46831600
C	0.77102300	-2.51338400	-0.56588600
C	1.98719400	-3.16931600	-0.73147800
C	3.12016600	-2.36900200	-0.79341400
C	3.04643000	-0.98631800	-0.71246600
Cu	-1.15831700	-0.34182000	-0.16369500
N	0.31912400	1.45989300	-0.19329400
C	1.57951500	1.05892300	-0.44663700
C	2.63038600	1.97257300	-0.58152100
C	2.32579000	3.31832100	-0.42917700
C	1.03480000	3.75120900	-0.15742700
C	0.05620400	2.76148200	-0.05087200
N	4.45051900	-3.00509900	-0.93654500
N	3.41496300	4.31536100	-0.55385000

O	3.11987400	5.50061000	-0.41218300
O	4.48634900	-4.23006900	-1.03372600
S	-1.07063900	-0.57146300	2.15222300
C	0.69905700	-0.50490900	2.57048300
O	-1.69279900	0.56995000	2.89373000
O	-1.52218500	-1.95786500	2.50488700
C	1.29527100	0.73836700	2.79239700
C	2.67079100	0.79432500	3.03210900
C	3.42945900	-0.38149300	3.04453500
C	2.81290400	-1.62158700	2.83812200
C	1.43841400	-1.69001800	2.59913200
O	4.54811500	3.89878300	-0.78970300
O	5.43685600	-2.27049800	-0.94465100
H	-0.15741700	-3.06770400	-0.49956700
H	2.04227900	-4.24752700	-0.79606500
H	3.95063000	-0.39714700	-0.75965000
H	3.64737300	1.67263200	-0.79020100
H	0.79758000	4.79930700	-0.03266500
H	-0.97088800	3.02949200	0.16614500
H	0.69498100	1.64103400	2.77424900
H	3.14749700	1.75492400	3.20069600
H	4.50026000	-0.33249400	3.21677800
H	3.40074300	-2.53414600	2.85682400
H	0.94535300	-2.64179500	2.43648500
C	-3.04350400	-0.05601300	0.17072100
C	-3.85133500	-1.16535700	0.38909000
C	-3.55266800	1.23642100	0.20973300
C	-5.20720100	-0.96531700	0.68328200
H	-3.44213900	-2.16935900	0.35526600
C	-4.90965300	1.41811800	0.50500700
H	-2.92681500	2.09718500	0.00711800
C	-5.75504200	0.32448700	0.74865300
H	-5.84047600	-1.83106400	0.86277000
H	-5.31099400	2.42840700	0.53647700
I	-1.71568700	-0.35089800	-2.70643300
C	-7.21113100	0.53406000	1.09742600
H	-7.82019800	-0.32603100	0.80310300
H	-7.33650200	0.67109300	2.17883200
H	-7.61475300	1.42505100	0.60640700

E(RB3LYP) -3607.56011056 a.u.

3.3.1.6. Ligand: *N,N'*-Dimethylethylenediamine (DMEDA)

(*N*,*N*'-Dimethylethylenediamine)copper(I) phenylsulfinate (**14e**)

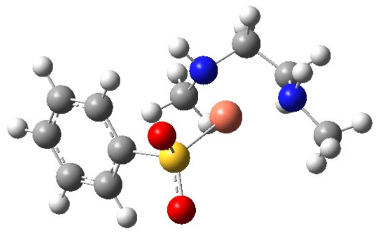

Geometry-optimised cartesian coordinates

Charge = 0 Multiplicity = 1

**ATOM**	**X**	**Y**	**Z**
C	2.35230600	-2.11868800	-0.68420800
N	0.94998700	-1.69590200	-0.46514400
Cu	1.11394300	0.43951000	-0.19876700
N	3.13516400	0.13415600	-0.03653200
C	3.29768400	-1.32194000	0.21577100
C	0.30170800	-2.33170200	0.70052900
C	3.83468400	0.96745800	0.96897200
S	-0.69533200	1.69193200	-0.17882700
O	-0.89236300	2.60058400	1.02782000
O	-0.98243700	2.36800900	-1.51549800
C	-2.02613500	0.44290800	-0.01354900
C	-2.59843300	0.19319800	1.23488900
C	-3.52668000	-0.84547600	1.36939000

C	-3.86681300	-1.63037300	0.26260300
C	-3.28561600	-1.37324600	-0.98572100
C	-2.36113700	-0.33526000	-1.12637800
H	2.59717300	-1.92175600	-1.73295600
H	2.48818100	-3.19352800	-0.50180800
H	3.05891600	-1.49783000	1.26888100
H	4.33465900	-1.64118600	0.05179900
H	0.34613900	-3.42808500	0.65020100
H	0.79209500	-2.00132700	1.61908600
H	-0.74061400	-2.01082400	0.73935000
H	3.73780900	2.01713300	0.68713400
H	4.89782400	0.70613300	1.04687800
H	3.35925100	0.82178300	1.94133000
H	-2.31565100	0.80698500	2.08383600
H	-3.97878600	-1.04325100	2.33697600
H	-4.58144600	-2.44082600	0.37107500
H	-3.55137500	-1.98071900	-1.84592400
H	-1.90064000	-0.12892200	-2.08783200
H	0.40457600	-1.91751900	-1.29434500
H	3.54578600	0.33924700	-0.94883800

E(RB3LYP) -2689.91028811 a.u.

(*N*,*N'*-Dimethylethylenediamine)copper(I) phenylsulfinate-4-iodotoluene cation-π complex (**15e**)

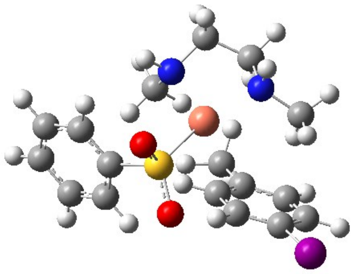

Geometry-optimised cartesian coordinates
Charge = 0 Multiplicity = 1

**ATOM**	**X**	**Y**	**Z**
C	2.15089700	2.75026600	-1.97082000
N	2.54559400	1.64933000	-1.06291600
Cu	0.91179100	0.22572200	-1.32734900
N	-0.09814800	1.75387500	-2.23529400
C	0.64377400	2.99448300	-1.88756900
C	2.76951400	2.07628300	0.33195000
C	-1.54635700	1.87000800	-1.94862200
S	1.42290900	-1.87009300	-0.83792500
O	0.39197800	-2.72113700	-0.10615600
O	2.10509500	-2.58251800	-1.99938600
C	2.75444900	-1.64116700	0.39728100
C	2.43061500	-1.59275500	1.75581300
C	3.42562600	-1.28200000	2.68872900
C	4.73188000	-1.01927500	2.25987600
C	5.04788700	-1.07506200	0.89657900
C	4.05701600	-1.38353800	-0.04030700
H	2.41474500	2.45222100	-2.99072600
H	2.68855100	3.68097000	-1.74259800
H	0.35865300	3.26551900	-0.86679600
H	0.35753500	3.82520900	-2.54508100
H	3.54847900	2.84721800	0.40863800
H	1.84187200	2.47614800	0.74340600
H	3.06075200	1.20773200	0.92568900
H	-2.04452500	0.94413700	-2.24016600
H	-1.99836500	2.71575400	-2.48265400
H	-1.68179300	2.01088400	-0.87635300
H	1.41415200	-1.80238800	2.07323300
H	3.18089600	-1.24427300	3.74634600
H	5.50151100	-0.77223400	2.98519600
H	6.06264200	-0.87620400	0.56401800
H	4.28568000	-1.42848100	-1.10073700
H	3.40394900	1.22673000	-1.40799700
H	0.00786700	1.60035000	-3.23935700

C	-0.95001000	2.08542700	2.18418400
C	-2.31727700	2.34471500	1.99885100
C	-3.15990700	1.41261700	1.38515900
C	-2.62226700	0.20084700	0.94766300
C	-1.27203800	-0.09634100	1.12324900
C	-0.44857400	0.85335500	1.74165800
H	-2.73636200	3.28873300	2.33804100
H	-4.21138900	1.64015000	1.24774900
H	-0.85498800	-1.03945200	0.78477400
H	0.60270000	0.61836500	1.88016800
C	-0.05106900	3.11248700	2.83164900
H	0.18463300	3.92341700	2.13131700
H	-0.53414400	3.56873300	3.70166800
H	0.89342800	2.66871200	3.15842500
I	-3.88544600	-1.21656000	-0.06672500

E(RB3LYP) -2972.31787540 a.u.

(*N,N'*-Dimethylethylenediamine)copper(I) phenylsulfinate-4-iodotoluene oxidative addition transition state‡

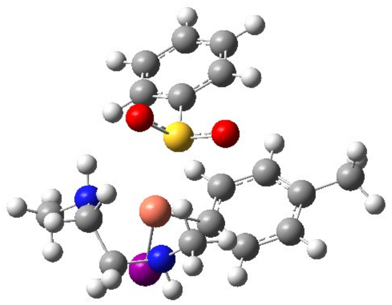

Geometry-optimised cartesian coordinates

Charge = 0 Multiplicity = 1

**ATOM**	**X**	**Y**	**Z**
C	-3.19822600	1.17641400	-0.32404000
N	-1.82955000	1.50545000	-0.79286600
Cu	-0.63991700	-0.11995800	-0.14016200
N	-2.42536000	-1.10688000	0.21562100
C	-3.46183200	-0.31775500	-0.50035900
C	-2.67856800	-1.25055700	1.67008200
C	-1.38784300	2.85026900	-0.36857700
H	-3.25531500	1.45077900	0.73343500
H	-3.95802800	1.75968700	-0.85832400
H	-3.39869900	-0.58008600	-1.56087300
H	-4.46987800	-0.56742900	-0.14666900
H	-3.65902600	-1.70105400	1.86701100
H	-1.89518800	-1.87762800	2.10002800
H	-2.63419100	-0.27022000	2.14837500
H	-2.10146500	3.62800600	-0.66761900
H	-1.28033500	2.86062900	0.71821300
H	-0.41585900	3.06047600	-0.81434500
H	-1.82499300	1.47613000	-1.81329700
H	-2.39223000	-2.03968300	-0.19171400
C	1.28186400	0.52121400	0.06376600
C	1.79782200	1.26160400	-1.01237400
C	1.29620100	1.01478100	1.37783300
C	2.28172500	2.54602500	-0.76307900
H	1.80586000	0.84666900	-2.01334900
C	1.77120200	2.31408200	1.59784800
H	0.94804000	0.40548000	2.20410000
C	2.27050000	3.07278700	0.53678600
H	2.66814000	3.13628600	-1.58855600
H	1.76563800	2.71545600	2.60667300
H	2.65618900	4.07082000	0.71868000
I	1.31974000	-1.82676800	-0.16988100

E(RB3LYP) -2972.30001333 a.u.

ν -107.15 cm^{-1}

(*N*,*N'*-Dimethylethylenediamine) *p*-tolyl(*S*-sulfinylphenyl)copper(III) iodide (**16e**)

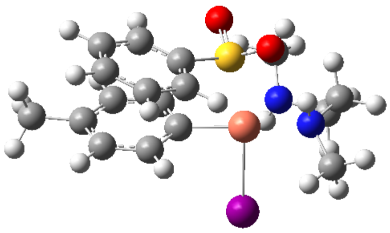

Geometry-optimised cartesian coordinates

Charge = 0 Multiplicity = 1

ATOM	X	Y	Z
C	3.44805100	-0.93238300	1.30162200
N	2.23194500	-0.32236800	1.88002300
Cu	0.89142700	-0.23520300	-0.03927800
N	2.21802400	-1.67858700	-0.71224700
C	3.05418400	-2.12531100	0.43732600
C	1.80879200	-0.97289200	3.13422200
C	3.01652700	-1.38195800	-1.92275000
S	-0.63920700	-1.95788300	0.42821000
C	-2.23893800	-1.67024500	-0.37069700
C	-2.32805900	-1.81174200	-1.75732800
C	-3.54640900	-1.53958800	-2.38374400
C	-4.64906500	-1.12862100	-1.62410900
C	-4.53848200	-0.98951800	-0.23655000
C	-3.32321300	-1.25368300	0.40055300
O	-0.09800000	-3.21629900	-0.21849700
O	-0.90905100	-2.04841400	1.89959900
H	3.94392600	-0.17910300	0.68349400
H	4.15613900	-1.25046500	2.07963600
H	3.94380000	-2.65548600	0.07790000
H	2.45639300	-2.83177800	1.01919500
H	1.49999000	-1.99914900	2.93218400
H	2.61190400	-0.97643800	3.88481700
H	0.94101500	-0.45315200	3.54225200
H	3.54081200	-2.28185600	-2.26480200
H	2.35189000	-1.02137600	-2.70916100
H	3.74451300	-0.60043500	-1.70520300
H	-1.46421500	-2.12758000	-2.33351900
H	-3.63435800	-1.64665400	-3.46063400
H	-5.59302600	-0.91307000	-2.11585500
H	-5.39247200	-0.66398600	0.34965700
H	-3.20924700	-1.12943800	1.47128600
H	1.58163400	-2.45019400	-0.93326600
H	2.41692800	0.66046300	2.06583800
C	-0.60717400	0.94760300	0.34700400
C	-1.43627900	1.37995900	-0.68690900
C	-0.87734700	1.27051100	1.66857300
C	-2.57844700	2.12235400	-0.37617000
H	-1.20991800	1.13745800	-1.71908300
C	-2.02952600	2.01627600	1.96487700
H	-0.23134100	0.93375900	2.46939300
C	-2.89746200	2.44591900	0.95296800
H	-3.23087000	2.44938000	-1.18245400
H	-2.24912000	2.25444100	3.00311100
C	-4.16143800	3.20940900	1.27513200
H	-4.32342000	4.02938700	0.56761400
H	-5.03743600	2.55132700	1.21483500
H	-4.13057800	3.62897200	2.28491800
I	2.09632700	1.92999700	-0.93022300

E(RB3LYP) -2972.30825871 a.u.

3.3.1.7. Ligand: L-Prolinate

(L-Prolinate)copper(I) phenylsulfinate (**14f**)

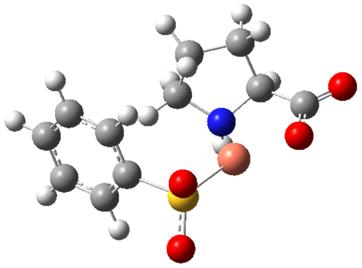

Geometry-optimised cartesian coordinates

Charge = -1 Multiplicity = 1

ATOM	X	Y	Z
Cu	-0.80339300	-0.99203700	0.13778900
O	-2.77040900	-1.23421000	-0.08109100
C	-3.48330000	-0.18445600	0.13449100
C	-2.74139900	1.12792500	0.45848500
N	-1.36725400	0.89263300	0.97933400
O	-4.72752600	-0.13729700	0.05781600
C	-0.54183000	2.04809400	0.54779000
C	-0.99619000	2.22525400	-0.89943200
C	-2.52315500	1.99979800	-0.82268200
S	1.21665900	-1.75580700	-0.20724200
O	1.74940000	-2.66661900	0.89392400
O	1.49840900	-2.26532000	-1.61686700
C	2.25160300	-0.24938400	-0.07568700
C	2.76992100	0.12509900	1.16667700
C	3.45582600	1.33772600	1.28777500
C	3.61091200	2.17057100	0.17349300
C	3.08767600	1.78708300	-1.06689800
C	2.40366600	0.57461500	-1.19430400
H	-3.34644400	1.67782000	1.18688100
H	0.51846100	1.81245800	0.65255600
H	-0.77104600	2.94414100	1.14274700
H	-0.52431100	1.45228700	-1.51708200
H	-0.72860800	3.20147100	-1.31150500
H	-2.91019000	1.50730700	-1.71854800
H	-3.05469500	2.94860500	-0.71410000
H	2.63534500	-0.52934100	2.02187000
H	3.86491800	1.63314800	2.24964500
H	4.13725200	3.11544100	0.27143200
H	3.20906500	2.43202400	-1.93235600
H	1.98925700	0.26698400	-2.14901000
H	-1.37775200	0.80480700	1.99361600

E(RB3LYP) -2821.47854912 a.u.

(L-Prolinate)copper(I) phenylsulfinate-4-iodotoluene cation-π complex (**15f**)

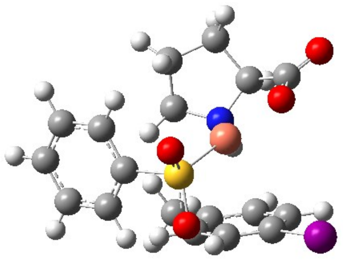

Geometry-optimised cartesian coordinates

Charge = -1 Multiplicity = 1

ATOM	X	Y	Z
Cu	-0.24531200	0.49070900	1.17531500
O	0.87298300	2.02933900	1.84110200
C	0.86422800	3.07393000	1.09343000
C	-0.04686900	3.05807400	-0.15190200
N	-0.42525100	1.67879500	-0.55981600

O	1.50789600	4.12135100	1.31588600
C	-1.79465400	1.74842500	-1.12530600
C	-2.48508700	2.69972000	-0.14975200
C	-1.41019700	3.77759000	0.11106500
S	-1.24078100	-1.36357700	1.79060000
O	-0.50403400	-2.65669500	1.45764900
O	-1.82295900	-1.33918400	3.20090100
C	-2.71814900	-1.41174000	0.70806000
C	-2.72176600	-2.21492900	-0.43432100
C	-3.80596900	-2.15191100	-1.31682400
C	-4.87468900	-1.28727000	-1.05459000
C	-4.86667700	-0.49173900	0.09820900
C	-3.78653200	-0.55183700	0.98250200
H	0.49472700	3.56464700	-0.95791200
H	-2.23616200	0.75008900	-1.15967900
H	-1.78345300	2.16931900	-2.14127200
H	-2.71460900	2.15671700	0.77382000
H	-3.41781000	3.11070400	-0.54450000
H	-1.46808100	4.18282200	1.12462200
H	-1.52283100	4.61458100	-0.58344300
H	-1.88579200	-2.88185200	-0.61877000
H	-3.81421400	-2.77288300	-2.20825400
H	-5.71169200	-1.23367000	-1.74469500
H	-5.69781000	0.17622500	0.30545300
H	-3.76830400	0.06353200	1.87671400
H	0.23398500	1.32294900	-1.24748600
C	2.14225300	-0.70447900	-1.19549700
C	0.93775700	-1.40394500	-1.30754600
C	0.11250200	-1.15175900	-2.40752200
C	0.46210000	-0.21390000	-3.39179300
C	1.67742200	0.47129600	-3.24702000
C	2.52316600	0.23344400	-2.15494100
H	0.62442400	-2.10249000	-0.53864100
H	-0.83311300	-1.67971600	-2.48688100
H	1.97276400	1.20908400	-3.98820900
H	3.45400100	0.78212900	-2.06296000
I	3.42038500	-1.06560000	0.49753600
C	-0.46657200	0.06210200	-4.55015400
H	-0.01442400	0.74633500	-5.27312600
H	-0.73131500	-0.86375800	-5.07250600
H	-1.40210700	0.51225300	-4.19759000

E(RB3LYP) -3103.88554404 a.u.

<u>(L-Prolinate)copper(I) phenylsulfinate-4-iodotoluene oxidative addition transition state‡</u>

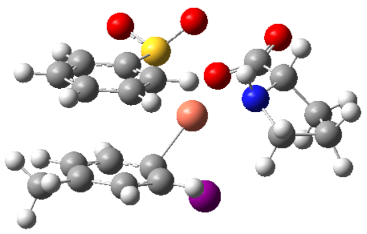

Geometry-optimised cartesian coordinates

Charge = -1 Multiplicity = 1

**ATOM**	**X**	**Y**	**Z**
C	-2.88037900	-1.88948600	0.54927200
N	-1.61613000	-1.33646500	1.10660000
Cu	-0.75344300	-0.08609700	-0.47616900
O	-2.16945800	-1.04246300	-1.61200000
C	-2.99255600	-1.79789900	-0.98604700
O	-3.91143500	-2.45827000	-1.52183700
C	-1.90572500	-0.71716500	2.43616700
C	-3.40454500	-0.95613100	2.69187500
C	-3.97538600	-1.08395700	1.27216800
S	0.91967400	-1.84206200	-0.96101100
C	2.51820700	-1.59255500	-0.09993200
C	2.55211000	-1.68524100	1.29442700
C	3.75757600	-1.47834700	1.96992400

C	4.92176700	-1.18390100	1.25016000
C	4.87892200	-1.09313700	-0.14492500
C	3.67170400	-1.28709200	-0.82380100
O	0.38888900	-3.12091500	-0.29551300
O	1.32153700	-2.03778000	-2.42192300
H	-2.98297300	-2.94988800	0.81226300
H	-1.26236600	-1.14611400	3.20971600
H	-1.69727300	0.35584700	2.37950200
H	-3.55662400	-1.89349700	3.23984600
H	-3.86124000	-0.14734700	3.27018500
H	-4.07154900	-0.09479700	0.80851200
H	-4.94564200	-1.58320000	1.22866100
H	1.64715600	-1.92172800	1.84562700
H	3.78886400	-1.54584200	3.05378100
H	5.85819200	-1.02139700	1.77626400
H	5.78136500	-0.86109100	-0.70330200
H	3.61697800	-1.21259900	-1.90495500
H	-0.93495900	-2.08992300	1.17781700
C	0.39007000	1.54819700	-0.12809900
C	0.99371600	1.53922800	1.13686800
C	1.13345800	1.84361600	-1.28205200
C	2.36854400	1.75980100	1.22863500
H	0.40668800	1.34510500	2.02668900
C	2.50772400	2.04842500	-1.16108200
H	0.64981800	1.89003200	-2.25101900
C	3.14431300	2.02995200	0.09128800
H	2.84311500	1.71711000	2.20491900
H	3.09199300	2.23785700	-2.05802100
I	-1.88192700	2.30646200	-0.18099400
C	4.61747100	2.33333700	0.20807100
H	5.03607800	1.92197100	1.13006600
H	5.17450400	1.91456000	-0.63509400
H	4.78976100	3.41707200	0.21273800

E(RB3LYP) -3103.86121253 a.u.
ν -99.82 cm^{-1}

(L-Prolinate) *p*-tolyl(*S*-sulfinylphenyl)copper(III) iodide (**16f**)

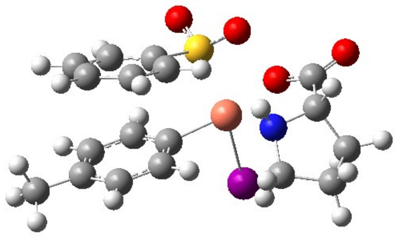

Geometry-optimised cartesian coordinates
Charge = -1 Multiplicity = 1

**ATOM**	**X**	**Y**	**Z**
C	2.63042200	1.85471600	0.84060700
N	1.37896700	1.19024400	1.29354000
Cu	0.70614000	-0.01229600	-0.52914400
O	2.13991700	0.98643800	-1.37331800
C	2.84120800	1.81872500	-0.67873600
O	3.71429800	2.55897400	-1.16669800
C	1.65547000	0.44891400	2.56164200
C	3.09755800	0.81137800	2.95007800
C	3.74729600	1.09864600	1.58972800
S	-0.72442700	1.71685700	-1.19510800
C	-2.34182800	1.71153500	-0.36204300
C	-2.46557300	2.34035700	0.87923000
C	-3.69581100	2.30000600	1.54041400
C	-4.78002600	1.62867500	0.96363700
C	-4.64001800	1.00459500	-0.28074700
C	-3.41510200	1.04191400	-0.95116900
O	-0.07191900	2.99336300	-0.72790000
O	-1.01915800	1.60661700	-2.66729600
H	2.63881000	2.90845400	1.14119000
H	0.92270600	0.71568800	3.32818400

H	1.56927600	-0.62427800	2.36292000
H	3.11321100	1.71430000	3.57197800
H	3.59397000	0.00797800	3.50246500
H	3.96837800	0.15902900	1.07039300
H	4.66477700	1.68771400	1.65061900
H	-1.62045800	2.86427700	1.31218500
H	-3.80569700	2.79018000	2.50319000
H	-5.73278800	1.59175500	1.48366800
H	-5.48050800	0.48189600	-0.72729700
H	-3.28922900	0.55633000	-1.91150500
H	0.66702700	1.89915900	1.43370400
C	-0.86308600	-1.03487300	-0.09668000
C	-1.37761200	-0.98000100	1.19435000
C	-1.51304300	-1.75332700	-1.09664800
C	-2.57937900	-1.64023400	1.47954000
H	-0.87369100	-0.41841300	1.97244500
C	-2.71619700	-2.40535900	-0.79790300
H	-1.10111600	-1.80543800	-2.09939100
C	-3.27170900	-2.35164900	0.48900500
H	-2.98694000	-1.58409200	2.48644100
H	-3.22866600	-2.95583600	-1.58382300
I	2.09323700	-2.23516300	-0.32637500
C	-4.60244300	-3.00133800	0.79056100
H	-4.64760500	-3.36319000	1.82270200
H	-5.42042000	-2.28089600	0.66074800
H	-4.79843100	-3.84505400	0.12171400

E(RB3LYP) -3103.87543824 a.u.

3.3.1.8. Ligand: (2*S*,4*R*)-*N*-(2,6-Dimethylphenyl)-4-hydroxypyrrolidine-2-carboxamide (DMPHPC)

((2*S*,4*R*)-*N*-(2,6-Dimethylphenyl)-4-hydroxypyrrolidine-2-carboxamide)copper(I) phenylsulfinate (**14g**)

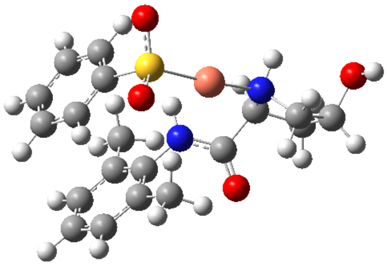

Geometry-optimised cartesian coordinates

Charge = 0 Multiplicity = 1

ATOM	X	Y	Z
Cu	1.30662300	1.44862000	0.09028700
N	0.10902200	-1.29503400	-0.39905200
C	1.34473400	-1.75937200	-0.05784300
C	2.51058100	-1.13532200	-0.85789000
N	2.79530900	0.25282000	-0.32774900
O	1.53260400	-2.57564100	0.84310200
C	3.79510800	0.01895300	0.76133400
C	4.76038100	-0.99298600	0.13535800
C	3.83346900	-1.91851200	-0.67493000
S	-0.39084500	2.82222100	0.34526600
O	-0.77972200	3.09549700	1.78472100
O	-0.26385800	4.06371400	-0.52036300
C	-1.80484400	1.90513400	-0.35719400
C	-1.80176700	1.62677300	-1.72845500
C	-2.84583600	0.87772200	-2.27535900
C	-3.87892300	0.41269800	-1.45227700
C	-3.86990400	0.69604200	-0.08439000
C	-2.82469300	1.43928100	0.47207200
C	-1.11657300	-1.76504100	0.17757500
C	-2.00998000	-2.46087300	-0.65828800
C	-3.21498500	-2.91422200	-0.10830600
C	-3.51567500	-2.68691800	1.23501100
C	-2.61770300	-1.98801400	2.04215300
C	-1.40687000	-1.50363600	1.52920400
C	-0.47129200	-0.71025200	2.40434500

C	-1.67946600	-2.70392700	-2.11054500
O	5.62915700	-0.33656100	-0.80329400
H	2.21521600	-1.03701600	-1.90465700
H	4.26187800	0.96297200	1.04705500
H	3.27654800	-0.40422600	1.62468100
H	5.34367600	-1.53289300	0.88819400
H	3.64781600	-2.84734800	-0.13477400
H	4.29281200	-2.15465900	-1.63625700
H	-0.99601500	1.99336700	-2.35820800
H	-2.85254900	0.65534800	-3.33817600
H	-4.68153900	-0.18257900	-1.87647300
H	-4.66010300	0.31703900	0.55533300
H	-2.78831300	1.65049900	1.53513400
H	-3.91721000	-3.45057300	-0.74034100
H	-4.45325200	-3.04504700	1.65029600
H	-2.86202600	-1.79583500	3.08317200
H	-0.93257500	-0.49514500	3.37113400
H	0.46550900	-1.24859000	2.57606300
H	-0.21624600	0.24887500	1.94102900
H	-2.41768400	-3.36402900	-2.57208300
H	-1.67549500	-1.76539900	-2.67893900
H	-0.68956000	-3.15804700	-2.22670600
H	6.26340500	0.20926800	-0.31662800
H	0.03361800	-0.75170100	-1.25136000
H	3.31319900	0.72446200	-1.07524000

E(RB3LYP) -3187.01556871 a.u.

((2*S*,4*R*)-*N*-(2,6-Dimethylphenyl)-4-hydroxypyrrolidine-2-carboxamide)copper(I) phenylsulfinate-4-iodotoluene cation-π complex (**15g**)

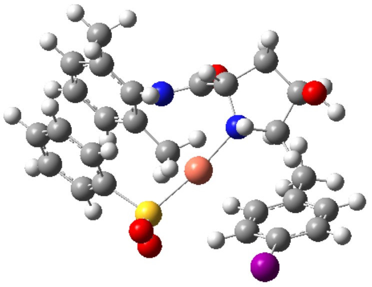

Geometry-optimised cartesian coordinates

Charge = 0 Multiplicity = 1

ATOM	X	Y	Z
Cu	-0.33493600	-0.39854900	-0.77836600
N	2.33858200	1.17883100	-0.71512400
C	1.48780600	2.24252800	-0.75480300
C	0.50555900	2.23870400	-1.94575900
N	-0.66420400	1.33632100	-1.62316700
O	1.46834200	3.12383800	0.10455900
C	-1.63908900	2.24496100	-0.94562900
C	-1.60093100	3.50751700	-1.80943500
C	-0.11538100	3.63568800	-2.19541100
S	0.21154400	-2.43953400	-0.14671500
O	-0.11277900	-2.76556100	1.29907100
O	-0.23038200	-3.49390800	-1.14479700
C	2.03330600	-2.45727300	-0.23123700
C	2.64047800	-2.32910900	-1.48593000
C	4.03395300	-2.29512600	-1.56938700
C	4.80728500	-2.38724600	-0.40481400
C	4.18906800	-2.51524800	0.84136800
C	2.79372900	-2.54353700	0.93425000
C	3.38392000	1.01706600	0.25196100
C	4.71386900	1.06937600	-0.20655700
C	5.74354300	0.90160800	0.72739700
C	5.45527400	0.69684500	2.07774300
C	4.12897400	0.64320200	2.50786900
C	3.06846700	0.79148800	1.60369900
C	1.64208200	0.69226700	2.07412500
C	5.01799800	1.29821100	-1.66690900

O	-2.33830000	3.29985000	-3.02664800
H	1.00653200	1.83276700	-2.82674400
H	-2.61767200	1.76930500	-0.89674800
H	-1.28786700	2.43882400	0.06933600
H	-1.97711200	4.38745400	-1.27712000
H	0.38063100	4.38421800	-1.57668200
H	-0.02733800	3.92884800	-3.24311600
H	2.03072800	-2.26217000	-2.38279300
H	4.51531400	-2.19550100	-2.53773700
H	5.89004700	-2.34478000	-0.47031200
H	4.78934300	-2.56756300	1.74392000
H	2.29445800	-2.62176500	1.89401800
H	6.77579300	0.93610400	0.39003700
H	6.26323700	0.56942600	2.79261700
H	3.90584200	0.46541200	3.55634300
H	1.59447800	0.33528800	3.10594900
H	1.13718700	1.66074000	2.01318700
H	1.06995400	-0.00717700	1.45520700
H	6.09250600	1.41606100	-1.82691100
H	4.68285000	0.45294500	-2.28094800
H	4.51180300	2.19294500	-2.04555700
H	-3.28201300	3.25179400	-2.81705800
H	2.36843800	0.57083000	-1.52540300
H	-1.08619200	1.10828800	-2.52803300
C	-2.28332200	-0.23388000	1.70330300
C	-3.48397700	-0.13968900	0.99618300
C	-4.29124400	0.99397500	1.09181200
C	-3.86861400	2.06212100	1.89119900
C	-2.65703600	2.01310900	2.59628700
C	-1.88522700	0.84635200	2.49857800
H	-1.66785200	-1.12779800	1.65923200
H	-5.22772600	1.06078600	0.54879600
H	-4.49436400	2.94847700	1.95638000
H	-0.95275200	0.77265300	3.04955900
I	-4.07757600	-1.76027800	-0.28968300
C	-2.17611000	3.19068100	3.40991500
H	-3.00805900	3.82528000	3.72855600
H	-1.62904400	2.86363900	4.29943800
H	-1.49310900	3.81252900	2.81746700

E(RB3LYP) -3469.41657294 a.u.

((2*S*,4*R*)-*N*-(2,6-Dimethylphenyl)-4-hydroxypyrrolidine-2-carboxamide)copper(I) phenylsulfinate-4-iodotoluene oxidative addition transition state‡

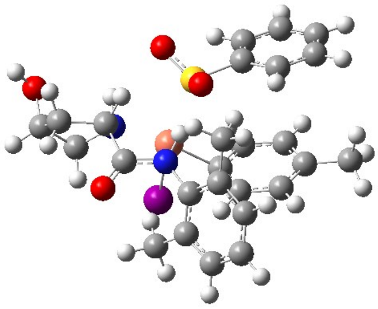

Geometry-optimised cartesian coordinates

Charge = 0 Multiplicity = 1

**ATOM**	**X**	**Y**	**Z**
C	-2.61930100	-0.61011800	-1.79756600
N	-1.98759600	-1.51140000	-0.76252400
Cu	-0.32360300	-0.91221500	0.31580600
N	-1.52393400	1.46490500	-1.12941800
C	-2.72717500	0.83175300	-1.25013500
C	-1.34717100	2.78213300	-0.61070100
O	-3.80371100	1.33785500	-0.93053200
C	-1.78637000	3.11149600	0.68926200
C	-1.53123900	4.40710900	1.16475400
C	-0.85538300	5.34522500	0.38594000
C	-0.42377000	4.99768600	-0.89640700

C	-0.66335400	3.72034400	-1.41622100
C	-2.49033300	2.12138300	1.58270800
C	-0.20868600	3.36419500	-2.81223900
C	-3.14531700	-2.07051100	-0.01277100
C	-4.15037400	-2.41150100	-1.10859100
C	-4.00333400	-1.23526200	-2.09902000
O	-3.70309200	-3.65460900	-1.67558600
S	0.88053800	-1.06113300	-1.76634000
C	2.69298700	-1.12477000	-1.71867000
C	3.42968200	0.04526300	-1.91180200
C	4.82269900	-0.00498500	-1.81097800
C	5.46133200	-1.21054000	-1.50020000
C	4.71010800	-2.37494000	-1.30064500
C	3.31757900	-2.33610500	-1.40904500
O	0.44845400	-2.35655500	-2.44219600
O	0.57346700	0.19452500	-2.59078600
H	-1.95824000	-0.59695600	-2.66632800
H	-1.85750600	4.66989700	2.16766500
H	-0.66214100	6.34056500	0.77576000
H	0.09936600	5.72721300	-1.50894400
H	-3.56642800	2.11018500	1.38458700
H	-2.13631900	1.10080600	1.41800400
H	-2.32933100	2.36770100	2.63578700
H	-1.02932700	2.93466800	-3.39822100
H	0.15934400	4.25053500	-3.33506400
H	0.59136800	2.61634900	-2.79965800
H	-2.83341500	-2.93517500	0.57370700
H	-3.53567700	-1.30304800	0.66045600
H	-5.17156200	-2.51414500	-0.72486400
H	-4.78991300	-0.49629700	-1.93503100
H	-4.06785400	-1.58391200	-3.13260600
H	-4.22990800	-3.85038500	-2.46240000
H	2.91613300	0.97283300	-2.14093400
H	5.40765100	0.89575500	-1.97090000
H	6.54336600	-1.24309100	-1.41292400
H	5.20680100	-3.31036600	-1.06069200
H	2.72336500	-3.23237800	-1.26171000
H	-0.74121300	1.07117500	-1.67297400
H	-1.58711700	-2.28880600	-1.29941300
C	1.11684400	-0.25731000	1.55439900
C	2.31650700	-0.97583900	1.61255100
C	1.09736900	1.14096400	1.54935400
C	3.51581600	-0.26744100	1.56763600
H	2.31440600	-2.05830400	1.65936400
C	2.31587600	1.82305800	1.48159600
H	0.16866600	1.69424000	1.57512400
C	3.53854000	1.13603400	1.49454500
H	4.45026600	-0.82098100	1.57126300
H	2.30294000	2.90899500	1.43646800
I	-0.74640400	-1.34064400	2.81679000
C	4.84508000	1.88334500	1.40390600
H	5.65955100	1.32497600	1.87430900
H	5.11843000	2.03858100	0.35308700
H	4.77765500	2.86757700	1.87673100

E(RB3LYP) -3469.40214604 a.u.
ν -100.11 cm^{-1}

((2*S*,4*R*)-*N*-(2,6-Dimethylphenyl)-4-hydroxypyrrolidine-2-carboxamide) *p*-tolyl(*S*-phenylsulfinyl)copper(III) iodide (**16g**)

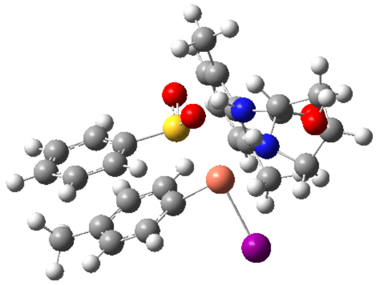

Geometry-optimised cartesian coordinates

Charge = 0 Multiplicity = 1

ATOM	X	Y	Z
C	2.59902500	-0.82537800	1.50788300
N	1.75267600	-1.74795000	0.65347900
Cu	0.10830300	-0.96725000	-0.28804100
N	1.76817400	1.38470300	0.89126600
C	2.82706600	0.52963800	0.79605100
C	1.77429700	2.75141200	0.46699400
O	3.87079400	0.79289300	0.19995800
C	1.94544900	3.09086200	-0.88936500
C	1.90492800	4.44985500	-1.23773600
C	1.68759200	5.43675500	-0.27699500
C	1.50665000	5.07693300	1.06098100
C	1.54973900	3.73485900	1.45575000
C	2.14114300	2.04376500	-1.95248900
C	1.36753100	3.35155000	2.90605600
C	2.74657800	-2.58635500	-0.07836300
C	3.76798500	-2.92580600	0.99957200
C	3.92194600	-1.59129600	1.75464700
O	3.15954200	-3.94230500	1.81247600
S	-0.86931700	-0.80467000	1.83021900
C	-2.67082800	-0.82271100	1.83898700
C	-3.36304500	0.38876600	1.86819900
C	-4.75829600	0.36124500	1.82209400
C	-5.43484800	-0.86056500	1.73843700
C	-4.72179500	-2.06498500	1.70663800
C	-3.32609200	-2.05330400	1.75130400
O	-0.42736500	-2.04986500	2.55529900
O	-0.46119600	0.50699100	2.46011200
H	2.04587900	-0.64416400	2.43091500
H	2.03152300	4.72714700	-2.28086900
H	1.65328300	6.48260200	-0.56871100
H	1.33806600	5.84406300	1.81186300
H	3.17778300	1.69316600	-1.97275300
H	1.52179500	1.16448500	-1.76568500
H	1.88211900	2.43645500	-2.93923800
H	2.18378800	2.70726500	3.25212700
H	1.34054600	4.24120800	3.53998500
H	0.43580300	2.79637400	3.06327500
H	2.25496300	-3.45848400	-0.50839600
H	3.19424800	-1.99342000	-0.87743500
H	4.71418300	-3.28483200	0.58013700
H	4.75956500	-1.02357800	1.34435600
H	4.10105200	-1.75081900	2.82044100
H	3.71875900	-4.10988200	2.58323500
H	-2.82070200	1.32546300	1.91829000
H	-5.31378300	1.29361700	1.84194800
H	-6.51971900	-0.87476400	1.69470800
H	-5.24974500	-3.01142900	1.64377500
H	-2.75694400	-2.97624400	1.72303100
H	1.02943400	1.13183900	1.55440800
H	1.29950100	-2.37738400	1.32670700
C	-1.45839800	-0.04087100	-0.96253900
C	-2.61168700	-0.71303200	-1.35479200
C	-1.39650900	1.34505400	-0.91110700
C	-3.74808100	0.03712700	-1.66979900
H	-2.63837700	-1.79557400	-1.40647900
C	-2.54948300	2.07928000	-1.23208600
H	-0.49658700	1.86913200	-0.61126900
C	-3.73800400	1.44032700	-1.60611300
H	-4.65640300	-0.48245000	-1.96469600
H	-2.50984200	3.16440100	-1.17568700
I	0.63057600	-1.55774900	-2.73110900
C	-4.99260000	2.22969300	-1.89808800
H	-5.53914500	1.80992600	-2.74876900
H	-5.66882400	2.20857700	-1.03420400
H	-4.76618600	3.27705600	-2.11726500

E(RB3LYP) -3469.40923851 a.u.

3.4. Oxidative addition to (bpy)Cu(SO_2Ph): Variation of $\Delta E_{OA}^{\ddagger}$ aryl iodide 4-position substituent

Geometry optimisations, followed by a frequency calculation were carried out for each species. The geometry-optimised molecular coordinates (Å) and energies (B3LYP/6-31G+(d,p) for C, H, N, O, S, Cu & SDD/ ECP46MWB for I) are shown below (solvent = *N,N*-dimethylformamide).

3.4.1. Geometry optimisations of common species to all pathways

(2,2'-Bipyridyl)copper(I) phenylsulfinate (**14b**)

See section 3.3.1.3 for the previously reported geometry-optimised structure of (2,2'-Bipyridyl)copper(I) phenylsulfinate.

3.4.1.1. Aryl iodide: Iodobenzene (R = H)

Iodobenzene (**17a**)

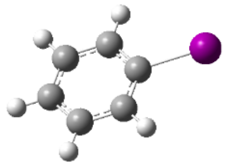

Geometry-optimised cartesian coordinates
Charge = 0 Multiplicity = 1

ATOM	X	Y	Z
C	3.36851600	0.00000100	0.00000000
C	2.66709300	-1.20966000	0.00000000
C	1.26689500	-1.21875100	0.00000000
C	0.58545000	-0.00000300	0.00000000
C	1.26689300	1.21875000	0.00000000
C	2.66708800	1.20966300	0.00000000
I	-1.57071400	0.00000000	0.00000000
H	4.45420300	0.00000500	0.00000000
H	3.20322600	-2.15426900	0.00000000
H	0.72780800	-2.15965800	0.00000000
H	0.72779800	2.15965300	0.00000000
H	3.20322400	2.15427000	0.00000000

E(RB3LYP) -243.08233067 a.u.

(2,2'-Bipyridyl)copper(I) phenylsulfinate-iodobenzene cation-π complex (**15a**)

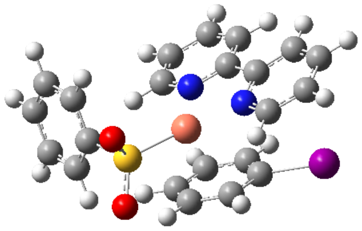

Geometry-optimised cartesian coordinates
Charge = 0 Multiplicity = 1

ATOM	X	Y	Z
C	1.51832100	-0.66719900	1.81660300
C	1.82492000	-2.65659500	0.64388100
C	3.00682200	-2.94034500	1.32548300
C	3.43854500	-2.04207900	2.30123700
C	2.68833800	-0.89335100	2.54904900
C	0.65932400	0.53675100	2.00257000
C	-1.34996000	1.56634700	1.43908700
C	-1.06912000	2.67453400	2.23788100
C	0.14067400	2.69884100	2.93100500
C	1.01782600	1.61959300	2.81292900
N	-0.51072200	0.52937800	1.32540900
N	1.09698100	-1.55566400	0.88460400
Cu	-0.70053400	-1.10774700	0.05223100

S	-2.56654500	-1.87036700	-0.85435200
O	-2.64790600	-1.86165900	-2.37581400
O	-3.09139000	-3.15085800	-0.21516000
C	-3.76586300	-0.58642800	-0.33818700
C	-4.24207800	-0.59587200	0.97702000
C	-5.05040200	0.45336800	1.42315900
C	-5.37247600	1.50747800	0.55859300
C	-4.89383300	1.50708100	-0.75653000
C	-4.08546200	0.45820300	-1.20880800
H	1.43778000	-3.32214200	-0.11971700
H	3.56324400	-3.84185100	1.09542200
H	4.34885700	-2.22819700	2.86170900
H	3.01532400	-0.18983800	3.30425000
H	-2.26905000	1.49760300	0.86775800
H	-1.77799000	3.49245900	2.30139700
H	0.40481100	3.54679100	3.55461500
H	1.96416300	1.63494600	3.33881000
H	-3.97602000	-1.41324400	1.64017400
H	-5.42385900	0.45166200	2.44312700
H	-5.99365600	2.32627100	0.90968000
H	-5.14595500	2.32224600	-1.42877900
H	-3.70727800	0.44123200	-2.22546000
C	0.76961500	0.27792800	-2.03743600
C	1.69093700	1.13018800	-1.42257900
C	1.33801200	2.42275600	-1.03332400
C	0.03585000	2.87395800	-1.28225700
C	-0.89583100	2.04143000	-1.90627200
C	-0.53072700	0.74434600	-2.27936300
H	1.04773800	-0.72821000	-2.33089300
H	2.05510200	3.07185800	-0.54344200
H	-0.24505600	3.87656500	-0.97431800
H	-1.24828800	0.07500200	-2.74245600
I	3.69447600	0.43666100	-1.07505800
H	-1.90693600	2.39287400	-2.08362500

E(RB3LYP) -3159.24436218 a.u.

<u>(2,2'-Bipyridyl)copper(I) phenylsulfinate-iodobenzene oxidative addition transition state‡</u>

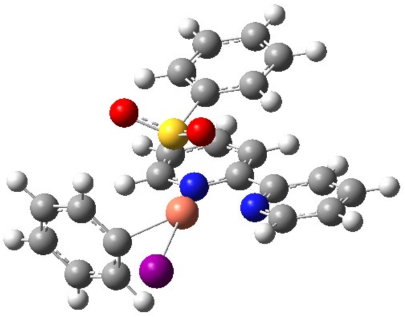

Geometry-optimised cartesian coordinates
Charge = 0 Multiplicity = 1

ATOM	X	Y	Z
C	1.81372000	-0.90309300	-1.57066200
C	0.66743900	-2.83916200	-0.96321700
C	1.69438200	-3.65776400	-1.42790400
C	2.81780500	-3.05156500	-1.99339900
C	2.87999800	-1.66058600	-2.06666600
C	1.79276500	0.58306700	-1.56177200
C	0.56328300	2.47682500	-0.99016400
C	1.57884800	3.33089400	-1.41550700
C	2.74455700	2.76455000	-1.93175600
C	2.85615500	1.37638700	-2.00428400
N	0.66685000	1.14488100	-1.06627700
N	0.72500900	-1.50336600	-1.03757300
Cu	-0.69383900	-0.21403500	-0.27410900
S	0.29504300	-0.39238100	2.07322100
C	2.04179300	0.10889500	1.84064300
C	2.37282600	1.46753000	1.85827400
C	3.00081200	-0.85471500	1.51944100

C	3.67972200	1.86230700	1.56101100
C	4.30832900	-0.45517300	1.22378600
C	4.64751900	0.90194800	1.24108700
O	0.39793100	-1.83357200	2.57549000
O	-0.22562200	0.58444000	3.13297800
H	-0.23231900	-3.24785000	-0.51423600
H	1.61025200	-4.73550700	-1.34683900
H	3.63941900	-3.65220600	-2.36978100
H	3.75024900	-1.18126000	-2.49672300
H	-0.36106300	2.85623000	-0.57004900
H	1.45566800	4.40491100	-1.33479500
H	3.56373100	3.39256000	-2.26632800
H	3.76313700	0.92761600	-2.38863200
H	1.61252800	2.20277300	2.10216300
H	2.71940900	-1.90270600	1.50007200
H	3.94052800	2.91675700	1.56930100
H	5.05643800	-1.20151200	0.97169200
H	5.66047600	1.21191500	1.00081500
I	-2.92544100	-1.49345800	0.01484900
C	-2.39056600	0.89424400	-0.17470400
C	-2.44404800	1.65236700	0.99978300
C	-2.73217800	1.42728800	-1.42466800
C	-2.78803100	3.00550300	0.89725200
H	-2.17245200	1.21833100	1.95538500
C	-3.07240200	2.78170200	-1.50103400
H	-2.71415800	0.80783000	-2.31361800
C	-3.10890500	3.56839200	-0.34368600
H	-2.80490900	3.61459700	1.79639700
H	-3.31621900	3.21335800	-2.46729800
H	-3.38609800	4.61591600	-0.40880300

E(RB3LYP) -3159.22500982 a.u.
ν -88.96 cm^{-1}

(2,2′-Bipyridyl) phenyl(*S*-sulfinylphenyl)copper(III) iodide (**16a**)

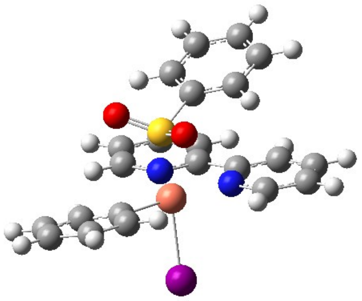

Geometry-optimised cartesian coordinates
Charge = 0 Multiplicity = 1

**ATOM**	**X**	**Y**	**Z**
C	-1.89194400	-1.38884800	-0.13030300
C	-0.99456300	-1.20630200	-2.28011400
C	-2.07389400	-1.87282200	-2.84992500
C	-3.09578400	-2.31272800	-2.00728300
C	-3.00385200	-2.07057500	-0.63839600
C	-1.71950600	-1.06329700	1.30987800
C	-0.41030100	0.01030000	2.89192700
C	-1.29624200	-0.30214700	3.92315900
C	-2.43746800	-1.04062700	3.60911700
C	-2.65468500	-1.42625900	2.28701400
N	-0.60903900	-0.36324200	1.62295300
N	-0.91179900	-0.96979200	-0.96175400
Cu	0.72020000	-0.04183400	-0.22159300
S	-0.10919300	2.08274500	-0.66078100
C	-1.91526600	1.94890100	-0.48875600
C	-2.48643100	2.11811600	0.77426600
C	-2.68789500	1.63541600	-1.60878000
C	-3.86751600	1.96351800	0.91633500
C	-4.06792800	1.48336600	-1.45479500
C	-4.65570100	1.64085400	-0.19423000
O	0.14560100	2.37422800	-2.11225300

O	0.29672100	3.13055200	0.32985400
H	-0.16841100	-0.84283300	-2.88068100
H	-2.10603000	-2.03694200	-3.92069600
H	-3.95749400	-2.83542700	-2.40895500
H	-3.79538100	-2.40449100	0.01955300
H	0.48586900	0.58915800	3.08688400
H	-1.09230400	0.02717500	4.93583500
H	-3.15376500	-1.30908100	4.37872100
H	-3.54259000	-1.99035600	2.03158200
H	-1.86265900	2.36319000	1.62624200
H	-2.21573200	1.51408200	-2.57693700
H	-4.32372100	2.08876800	1.89345300
H	-4.68009200	1.23679100	-2.31684700
H	-5.72756800	1.51209900	-0.07754900
I	2.09190000	-2.26745600	-0.16170800
C	2.39818800	0.89453500	0.01187400
C	3.07127900	1.34303000	-1.12074300
C	2.88917600	1.12052000	1.29413900
C	4.26260700	2.06516300	-0.95969100
H	2.66911000	1.16191800	-2.11196700
C	4.07966800	1.84499100	1.44533200
H	2.37632800	0.73568800	2.16730000
C	4.76555300	2.31753900	0.32103000
H	4.78918500	2.42828700	-1.83823700
H	4.46883800	2.02787200	2.44336000
H	5.68933900	2.87572300	0.44260700

E(RB3LYP) -3159.23128040 a.u.

3.4.1.2. Aryl iodide: 4-Iodotoluene (R = Me)

See section 3.3.1.1 for the previously reported geometry-optimised structure of 4-iodotoluene (**17b**). Additionally, see section 3.3.1.3 for the previously reported geometry-optimised structures of (2,2'-bipyridyl)copper(I) phenylsulfinate (**14b**), (2,2'-bipyridyl)copper(I) phenylsulfinate-4-iodotoluene cation-π complex (**15b**), (2,2'-bipyridyl)copper(I) phenylsulfinate-4-iodotoluene oxidative addition transition state‡ and (2,2'-bipyridyl) *p*-tolyl(*S*-sulfinylphenyl)copper(III) iodide (**16b**).

3.4.1.3. Aryl iodide: 4-Iodoanisole (R = OMe)

4-Iodoanisole (**17c**)

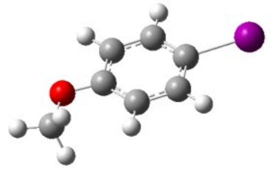

Geometry-optimised cartesian coordinates
Charge = 0 Multiplicity = 1

ATOM	X	Y	Z
C	0.58897600	-1.08107500	-0.00000100
C	1.98719100	-0.98523800	-0.00000200
C	2.59925200	0.27598400	-0.00000100
C	1.80558300	1.43490200	0.00000000
C	0.41576600	1.33940300	0.00000000
C	-0.18548300	0.07619500	-0.00000100
O	3.94772700	0.48099400	-0.00000100
I	-2.33382900	-0.07828000	0.00000000
C	4.81412800	-0.65834100	0.00000300
H	0.12670200	-2.06210300	-0.00000300
H	2.57215200	-1.89668000	-0.00000200
H	2.29117900	2.40558200	0.00000000
H	-0.18283400	2.24365500	0.00000100
H	5.82822900	-0.25864800	0.00000400
H	4.66161500	-1.27096400	0.89625700
H	4.66161800	-1.27096700	-0.89625000

E(RB3LYP) -357.61578516 a.u.

(2,2′-Bipyridyl)copper(I) phenylsulfinate-4-iodoanisole cation-π complex (**15c**)

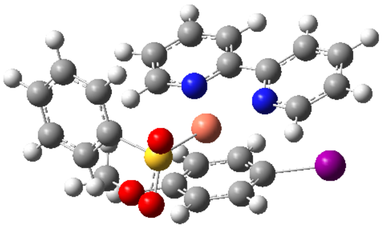

Geometry-optimised cartesian coordinates

Charge = 0 Multiplicity = 1

ATOM	X	Y	Z
C	1.57983300	-1.16991000	1.62606000
C	2.05472100	-2.70222000	-0.06221700
C	3.22752000	-3.11587900	0.56725500
C	3.57071100	-2.52111900	1.78070700
C	2.74079400	-1.53578600	2.31458500
C	0.62244400	-0.14447100	2.13057600
C	-1.46718300	0.83771900	1.83988500
C	-1.29791200	1.65779500	2.95419600
C	-0.09973300	1.56851800	3.66368200
C	0.87273700	0.65778100	3.25016700
N	-0.53272200	-0.03434200	1.43855600
N	1.24989800	-1.76083400	0.45245800
Cu	-0.54031700	-1.16643100	-0.30888900
S	-2.32104700	-1.74812200	-1.48351500
O	-2.46155400	-1.12905100	-2.86906200
O	-2.65080100	-3.23619800	-1.43686100
C	-3.65434000	-0.96248200	-0.50473000
C	-4.12036500	-1.60329400	0.64788700
C	-5.03819200	-0.94881300	1.47458200
C	-5.48024500	0.34010400	1.14965300
C	-5.00709700	0.97272100	-0.00545500
C	-4.08750400	0.32349500	-0.83664800
H	1.73867900	-3.12914600	-1.00765400
H	3.84657800	-3.88122400	0.11311800
H	4.47302900	-2.81498600	2.30685800
H	2.99618800	-1.06978100	3.25779600
H	-2.37170300	0.87143300	1.24163200
H	-2.08143900	2.34806500	3.24637300
H	0.07984200	2.19845100	4.52891200
H	1.80841300	0.58663100	3.79056800
H	-3.76458700	-2.59984100	0.89061500
H	-5.40545300	-1.44115100	2.37052100
H	-6.18806100	0.84996400	1.79679900
H	-5.34726600	1.97324300	-0.25639000
H	-3.69319000	0.81332000	-1.71908200
C	1.02678800	0.75480300	-1.81413600
C	1.93416100	1.36557500	-0.94028300
C	1.53757400	2.43368000	-0.14091500
C	0.22628700	2.92088800	-0.22234300
C	-0.68311700	2.32687300	-1.10531300
C	-0.28185500	1.23411300	-1.89336500
H	1.32324100	-0.08635800	-2.43073200
H	2.23145400	2.89970600	0.54985400
H	-0.06176200	3.74946100	0.41238000
H	-1.00015200	0.76045000	-2.55472800
I	3.95327200	0.64780200	-0.83422900
O	-1.97732300	2.72186500	-1.26199500
C	-2.46961700	3.79965900	-0.46071800
H	-1.92048800	4.72640800	-0.66329300
H	-3.51452100	3.92464900	-0.74415000
H	-2.40755300	3.55846200	0.60633300

E(RB3LYP) -3273.78040421 a.u.

(2,2'-Bipyridyl)copper(I) phenylsulfinate-4-iodoanisole oxidative addition transition state‡

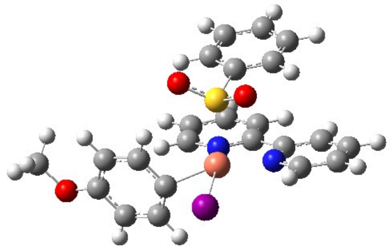

Geometry-optimised cartesian coordinates

Charge = 0   Multiplicity = 1

ATOM	X	Y	Z
C	2.37167400	-0.19535500	-1.52718600
C	2.20686500	-2.42619600	-0.87264800
C	3.51421100	-2.69546900	-1.27117100
C	4.26389200	-1.65589400	-1.82471700
C	3.68871800	-0.39242300	-1.95456800
C	1.67434000	1.11557100	-1.58480700
C	-0.29808900	2.25467900	-1.09873500
C	0.22222800	3.46230600	-1.55974100
C	1.52799700	3.47381500	-2.05087900
C	2.26497300	2.28994200	-2.06220700
N	0.40588400	1.11714600	-1.11700100
N	1.65254000	-1.21406300	-1.00217100
Cu	-0.21597800	-0.69865300	-0.30805300
S	0.75044500	-0.38351700	2.06447000
C	2.03461600	0.90435900	1.84199300
C	1.65373100	2.25001100	1.81367000
C	3.35222400	0.53053800	1.56811900
C	2.60466600	3.22940900	1.51670900
C	4.30190100	1.51472600	1.27363400
C	3.92861500	2.86251600	1.24399600
O	1.53853100	-1.59587300	2.56460700
O	-0.18309900	0.21285100	3.12498200
H	1.57251700	-3.18952000	-0.43346000
H	3.92659800	-3.69038600	-1.14771900
H	5.28637900	-1.82250700	-2.14756800
H	4.26503800	0.42327900	-2.37221400
H	-1.30180000	2.18314000	-0.69590600
H	-0.38093900	4.36244500	-1.52618400
H	1.97429100	4.39379600	-2.41399000
H	3.28325300	2.29160200	-2.42963200
H	0.62327700	2.52202300	2.02016000
H	3.62286400	-0.52039800	1.58449500
H	2.31236800	4.27521300	1.48814200
H	5.32742000	1.22773700	1.05850600
H	4.66402200	3.62492700	1.00355800
I	-1.61460300	-2.84198400	0.02192000
C	-2.20054900	-0.42982600	-0.20529700
C	-2.59959300	0.23645800	0.95446200
C	-2.78880200	-0.15028600	-1.44917700
C	-3.54093500	1.26826900	0.85863200
H	-2.14667400	0.00468500	1.91245500
C	-3.72453300	0.87424300	-1.53700500
H	-2.50139400	-0.70535500	-2.33439800
C	-4.10863900	1.58716000	-0.38574100
H	-3.81434800	1.80651300	1.75791800
H	-4.17238700	1.13297200	-2.49114900
O	-5.03445900	2.56417000	-0.58133000
C	-5.48909100	3.31955900	0.54870500
H	-4.66225100	3.86846900	1.01347100
H	-6.22071000	4.02524600	0.15562000
H	-5.96662000	2.66904900	1.29001400

E(RB3LYP)   -3273.75962766   a.u.

ν   -72.09 cm^{-1}

(2,2′-Bipyridyl) 4-methoxyphenyl(*S*-sulfinylphenyl)copper(III) iodide (**16c**)

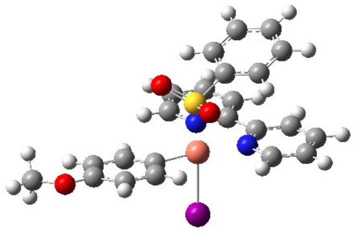

Geometry-optimised cartesian coordinates

Charge = 0   Multiplicity = 1

ATOM	X	Y	Z
C	2.54735900	0.99558500	0.07498500
C	1.78615300	1.21822500	-2.12303000
C	3.02929100	1.65240900	-2.57049300
C	4.06376200	1.76020500	-1.63986300
C	3.82129800	1.43085100	-0.30807500
C	2.20118800	0.60081800	1.46577400
C	0.56642500	-0.25453700	2.86791600
C	1.42316600	-0.24685700	3.96875600
C	2.72498100	0.22035900	3.78597800
C	3.12169100	0.64896300	2.51996200
N	0.93875600	0.16221500	1.65264300
N	1.55738800	0.89692700	-0.84030400
Cu	-0.29474600	0.32761200	-0.27337200
S	0.04791300	-1.88587900	-0.89112700
C	1.81806100	-2.20622000	-0.61790500
C	2.24124000	-2.61522700	0.64852400
C	2.72170200	-1.99188200	-1.66055900
C	3.60622200	-2.80839000	0.87430400
C	4.08425300	-2.18849500	-1.42345500
C	4.52626300	-2.58971600	-0.15740700
O	-0.16200300	-1.97541900	-2.37621200
O	-0.66189700	-2.89234700	-0.03815700
H	0.94278000	1.11572600	-2.79682900
H	3.17473100	1.89507300	-3.61667800
H	5.05014700	2.09327400	-1.94529500
H	4.62038200	1.50771900	0.41753500
H	-0.45197700	-0.61589700	2.95948500
H	1.07477700	-0.59826300	4.93349700
H	3.42623500	0.24684800	4.61365300
H	4.13276500	1.00382100	2.36628300
H	1.51760100	-2.77531000	1.43956000
H	2.36216700	-1.67832700	-2.63391400
H	3.94866400	-3.12239100	1.85551800
H	4.79786300	-2.02241300	-2.22463300
H	5.58723000	-2.73167300	0.02509300
I	-1.11237600	2.79977600	-0.05290000
C	-2.15457100	-0.20056500	-0.22301100
C	-2.83946500	-0.36663600	-1.42654300
C	-2.78296100	-0.42787800	0.99286800
C	-4.16651800	-0.79759800	-1.40662200
H	-2.34394500	-0.19023600	-2.37531600
C	-4.11718900	-0.86381200	1.02084800
H	-2.26690000	-0.26349500	1.93119400
C	-4.80961100	-1.05005200	-0.18372200
H	-4.71541300	-0.94563100	-2.33185200
H	-4.59061800	-1.04048000	1.97923700
O	-6.10975700	-1.46889700	-0.27249000
C	-6.82367800	-1.73472900	0.93750100
H	-6.90015200	-0.83432100	1.55876200
H	-7.82120400	-2.05055800	0.63080500
H	-6.34627600	-2.53790400	1.51154400

E(RB3LYP)   -3273.76375302   a.u.

3.4.1.4. Aryl iodide: 1-Iodo-4-nitrobenzene (R = NO_2)

1-Iodo-4-nitrobenzene (**17d**)

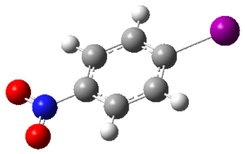

Geometry-optimised cartesian coordinates

Charge = 0 Multiplicity = 1

ATOM	X	Y	Z
C	0.26770700	1.22014200	0.00003100
C	1.66149900	1.22191900	0.00004300
C	2.33760000	0.00006400	0.00004800
C	1.66166000	-1.22178500	0.00005800
C	0.26787900	-1.22019000	0.00004600
C	-0.41637800	-0.00001900	0.00002800
N	3.80324700	-0.00000100	0.00003100
I	-2.55508900	-0.00001700	-0.00001500
O	4.38936400	-1.08791300	-0.00008900
O	4.38920500	1.08792500	-0.00004800
H	-0.26913900	2.16135200	0.00001400
H	2.21310400	2.15346000	0.00003600
H	2.21339900	-2.15327900	0.00006600
H	-0.26872400	-2.16149700	0.00004500

E(RB3LYP) -447.59523531 a.u.

(2,2'-Bipyridyl)copper(I) phenylsulfinate-1-iodo-4-nitrobenzene cation-π complex (**15d**)

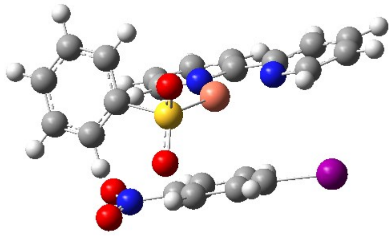

Geometry-optimised cartesian coordinates

Charge = 0 Multiplicity = 1

ATOM	X	Y	Z
C	1.80420900	-1.17192600	1.66842900
C	2.39607900	-2.66646700	-0.01953300
C	3.62845100	-2.93314600	0.57411100
C	3.94213600	-2.28191500	1.76640500
C	3.02205400	-1.39128600	2.31935800
C	0.75229700	-0.25450200	2.19279300
C	-1.43566000	0.49767400	1.94280300
C	-1.32981300	1.34311000	3.04649700
C	-0.11533500	1.38463200	3.73119900
C	0.93889100	0.57710200	3.30257700
N	-0.42290000	-0.27337900	1.52500600
N	1.50726300	-1.81298100	0.51153500
Cu	-0.33087000	-1.42228500	-0.22456000
S	-2.00682600	-1.85158100	-1.59024000
O	-2.06572800	-1.00958100	-2.85740200
O	-2.28860800	-3.33138000	-1.81672800
C	-3.42568700	-1.26475700	-0.59300200
C	-3.80165900	-2.00120500	0.53594400
C	-4.78565300	-1.49539300	1.38914600
C	-5.38174100	-0.25669300	1.11654200
C	-4.99972000	0.47128800	-0.01535700
C	-4.01703500	-0.03259800	-0.87572100
H	2.10068600	-3.14002700	-0.94896000
H	4.31568700	-3.63014900	0.10839300
H	4.89010400	-2.46112100	2.26292900
H	3.25399400	-0.88611300	3.24837500
H	-2.35381500	0.42847100	1.36918600

H	-2.17414300	1.95352600	3.34553800
H	0.01546700	2.03834800	4.58734500
H	1.88759800	0.60977000	3.82322600
H	-3.32261700	-2.95281800	0.74675900
H	-5.08183400	-2.06140000	2.26758800
H	-6.13764300	0.14011900	1.78786900
H	-5.45578300	1.43385600	-0.22703800
H	-3.70203000	0.52697000	-1.74864200
C	0.73897100	1.04861600	-1.71777200
C	1.73759200	1.51833000	-0.85729500
C	1.45826400	2.47184000	0.12857000
C	0.16782700	2.98404300	0.23774700
C	-0.81109600	2.54241100	-0.65529000
C	-0.54986400	1.57656500	-1.62731000
H	0.94859600	0.28512700	-2.45755200
H	2.23068300	2.81814500	0.80472200
H	-0.07410200	3.71878500	0.99505200
H	-1.33032900	1.20311100	-2.27809600
I	3.72884300	0.77958200	-1.05732100
N	-2.15925500	3.10075400	-0.55501500
O	-2.94199800	2.91091600	-1.49153300
O	-2.45510800	3.73429800	0.46462200

E(RB3LYP) -3363.76005937 a.u.

(2,2′-Bipyridyl)copper(I) phenylsulfinate-1-iodo-4-nitrobenzene oxidative addition transition state‡

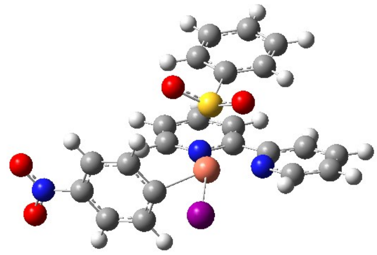

Geometry-optimised cartesian coordinates

Charge = 0 Multiplicity = 1

**ATOM**	**X**	**Y**	**Z**
C	2.41632500	0.05601000	-1.59920800
C	2.51814300	-2.19858900	-1.01030100
C	3.81573700	-2.32213800	-1.50112500
C	4.41838400	-1.19763100	-2.06789200
C	3.71373100	0.00459600	-2.11803500
C	1.58994300	1.28932000	-1.56284000
C	-0.45761700	2.20676200	-0.93545700
C	-0.07589800	3.48066000	-1.34927500
C	1.19856100	3.64353800	-1.89214700
C	2.04251200	2.53858400	-1.99784400
N	0.34708600	1.14349200	-1.04591900
N	1.83986400	-1.04505800	-1.06383900
Cu	-0.02024500	-0.73581800	-0.27950900
S	0.94921800	-0.40710400	2.05212800
C	2.11721600	0.98419700	1.83117900
C	1.63472700	2.29597800	1.87886600
C	3.44574400	0.72275600	1.48796900
C	2.49634800	3.35659300	1.58805400
C	4.30497800	1.78821500	1.19971900
C	3.83064700	3.10350100	1.24590500
O	1.84216400	-1.56810800	2.48577100
O	-0.00130200	0.07959600	3.14976200
H	1.99508400	-3.03400200	-0.55666200
H	4.33233700	-3.27296500	-1.43762300
H	5.42746600	-1.25159000	-2.46301700
H	4.17608000	0.88427100	-2.54731800
H	-1.43025900	2.02349700	-0.49459600
H	-0.75949700	4.31463300	-1.23922500
H	1.53946300	4.61972300	-2.22079300
H	3.03932100	2.65761700	-2.40249000
H	0.59731700	2.47866100	2.14075100

H	3.79475000	-0.30407800	1.44757700
H	2.12601700	4.37720500	1.61924400
H	5.33876600	1.58994200	0.93124800
H	4.49600700	3.92927700	1.01105900
I	-1.21496400	-3.01147200	-0.00953800
C	-2.02940900	-0.72589400	-0.09070000
C	-2.45864800	-0.16032700	1.12089100
C	-2.65803800	-0.41830500	-1.31155200
C	-3.49056100	0.77334700	1.09866300
H	-1.95991100	-0.40012200	2.05312700
C	-3.68893700	0.51466800	-1.32842000
H	-2.33382900	-0.88762700	-2.23227000
C	-4.09620800	1.09569500	-0.12181000
H	-3.82091200	1.24872100	2.01391600
H	-4.17399100	0.78546300	-2.25783500
N	-5.18120600	2.07108600	-0.13831300
O	-5.54210000	2.56715600	0.93636000
O	-5.69348500	2.36209200	-1.22679700

E(RB3LYP) -3363.73831340 a.u.
ν -66.53 cm^{-1}

(2,2'-Bipyridyl) 4-nitrophenyl(*S*-sulfinylphenyl)copper(III) iodide (**16d**)

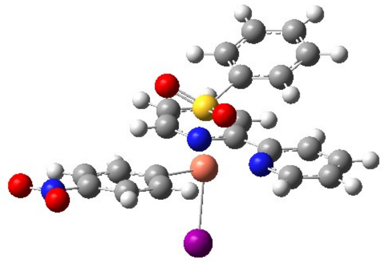

Geometry-optimised cartesian coordinates
Charge = 0 Multiplicity = 1

**ATOM**	**X**	**Y**	**Z**
C	2.71675500	0.92816300	-0.08436000
C	1.84569200	1.03011400	-2.25200600
C	3.07734400	1.38525200	-2.79108900
C	4.16373000	1.51452500	-1.92450600
C	3.98243200	1.28581200	-0.56223800
C	2.43047800	0.64601500	1.34642200
C	0.84108800	-0.04557500	2.88487600
C	1.75277600	0.00898900	3.93906300
C	3.05846100	0.41334600	3.65977000
C	3.40443600	0.73590100	2.34819500
N	1.16518300	0.26876100	1.62558900
N	1.67667000	0.80498100	-0.93953500
Cu	-0.14903400	0.34529700	-0.24127100
S	0.06911100	-1.92020700	-0.69527000
C	1.83693800	-2.28553700	-0.49915600
C	2.31892000	-2.61705000	0.76877200
C	2.68117600	-2.18257200	-1.60674300
C	3.68719300	-2.84832300	0.92829700
C	4.04725900	-2.41597200	-1.43385400
C	4.54976500	-2.74197400	-0.16863400
O	-0.24307300	-2.11906500	-2.14813400
O	-0.63232000	-2.79766900	0.29239100
H	0.96396800	0.91546300	-2.87241100
H	3.17435500	1.55176100	-3.85750500
H	5.14326500	1.78720600	-2.30292400
H	4.82158100	1.37952800	0.11437700
H	-0.18268600	-0.36096500	3.05304700
H	1.44245000	-0.25919900	4.94268500
H	3.80132600	0.47297900	4.44842700
H	4.41733300	1.04254000	2.12035700
H	1.63886800	-2.68990900	1.60965600
H	2.27506200	-1.92720700	-2.57870800
H	4.07705300	-3.10420900	1.90854400
H	4.71654400	-2.33763700	-2.28494000

H	5.61399400	-2.91290500	-0.03778200
I	-0.88871800	2.84643300	-0.13218800
C	-2.01894700	-0.09280200	-0.09731000
C	-2.74974000	-0.29405200	-1.27111200
C	-2.61202100	-0.24233400	1.15804800
C	-4.09038900	-0.66798100	-1.19424600
H	-2.27621100	-0.18596000	-2.24028400
C	-3.95155100	-0.61495100	1.24845200
H	-2.05047800	-0.06181400	2.06563700
C	-4.67284000	-0.82408200	0.06796700
H	-4.67373800	-0.83869600	-2.09063900
H	-4.43142800	-0.73741000	2.21155700
N	-6.07644100	-1.21632800	0.15654300
O	-6.70667600	-1.39527500	-0.89353900
O	-6.58100800	-1.35501800	1.27831000

E(RB3LYP) -3363.74734701 a.u.

3.4.1.5. Aryl iodide: 1-Fluoro-4-iodobenzene (R = F)

1-Fluoro-4-iodobenzene (**17e**)

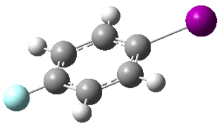

Geometry-optimised cartesian coordinates

Charge = 0 Multiplicity = 1

**ATOM**	**X**	**Y**	**Z**
C	-0.89711600	1.21774400	-0.00000200
C	-2.29621500	1.21947100	0.00000100
C	-2.96148300	0.00000100	-0.00000200
C	-2.29621500	-1.21947000	-0.00000200
C	-0.89711600	-1.21774400	0.00000100
C	-0.21269000	0.00000000	-0.00000100
F	-4.32490900	-0.00000100	0.00000200
I	1.93823500	0.00000000	0.00000000
H	-0.36212200	2.16051700	-0.00000100
H	-2.85651300	2.14798000	0.00000100
H	-2.85651600	-2.14797700	-0.00000100
H	-0.36212200	-2.16051700	0.00000100

E(RB3LYP) -342.32201426 a.u.

(2,2'-Bipyridyl)copper(I) phenylsulfinate-1-fluoro-4-iodobenzene cation-π complex (**15e**)

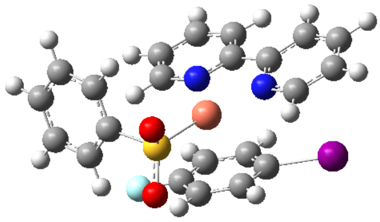

Geometry-optimised cartesian coordinates

Charge = 0 Multiplicity = 1

**ATOM**	**X**	**Y**	**Z**
C	1.54719200	-1.02707400	1.70956200
C	1.95696800	-2.71926800	0.16121700
C	3.14052400	-3.08955500	0.79718800
C	3.52172100	-2.39079900	1.94206400
C	2.71881500	-1.34767100	2.40230200
C	0.62343800	0.06424000	2.13166700
C	-1.44611600	1.06940900	1.78240300
C	-1.23106200	1.99351400	2.80422300
C	-0.02197100	1.94130900	3.49761000
C	0.91824500	0.96727600	3.15920600
N	-0.54425700	0.13468700	1.45464300
N	1.17861300	-1.72084400	0.60536700
Cu	-0.61841600	-1.18959100	-0.15910300

S	-2.40087200	-1.68762100	-1.36109100
O	-2.43950600	-1.13705100	-2.78123800
O	-2.85532100	-3.13751500	-1.24736900
C	-3.69438000	-0.73808000	-0.47947500
C	-4.18074900	-1.22865200	0.73723700
C	-5.06623100	-0.45019800	1.48726700
C	-5.45585300	0.81320300	1.02347700
C	-4.96433400	1.29508800	-0.19461600
C	-4.07747000	0.51969400	-0.95014800
H	1.61063800	-3.22801000	-0.73142300
H	3.73797300	-3.90290800	0.40143000
H	4.43270400	-2.64929100	2.47184700
H	3.00424900	-0.80126600	3.29222700
H	-2.36217600	1.07052400	1.20141100
H	-1.98894300	2.73331700	3.03601000
H	0.19249800	2.64977800	4.29115200
H	1.86362000	0.92597000	3.68541700
H	-3.86342700	-2.20476700	1.09122500
H	-5.44807900	-0.82538100	2.43238100
H	-6.13751600	1.42021600	1.61206300
H	-5.26361500	2.27516900	-0.55460800
H	-3.68073300	0.88444900	-1.89086700
C	0.82402300	0.79408000	-1.87619000
C	1.78852900	1.38942800	-1.05839800
C	1.49121900	2.52235900	-0.29764000
C	0.21240500	3.08381400	-0.37037500
C	-0.72712700	2.49019000	-1.20264300
C	-0.45783300	1.35426000	-1.95388000
H	1.04998100	-0.09483000	-2.45409700
H	2.23589200	2.97235800	0.34860400
H	-0.05023300	3.95867500	0.21362000
H	-1.22485900	0.88859000	-2.56266000
I	3.76238300	0.55751500	-0.97691100
F	-1.97420800	3.03771000	-1.26884200

E(RB3LYP) -3258.48453869 a.u.

(2,2'-Bipyridyl)copper(I) phenylsulfinate-1-fluoro-4-iodobenzene oxidative addition transition state‡

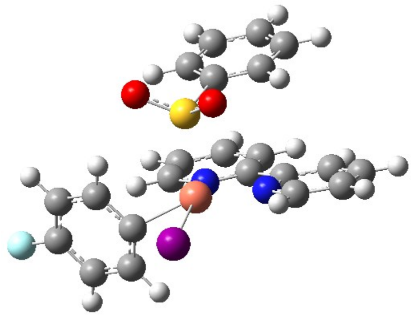

Geometry-optimised cartesian coordinates

Charge = 0 Multiplicity = 1

ATOM	X	Y	Z
C	2.07228400	-0.58805300	-1.59307200
C	1.35253400	-2.73050700	-1.02415700
C	2.52329200	-3.31037400	-1.50744400
C	3.49492100	-2.47466600	-2.06120700
C	3.26891800	-1.09953800	-2.10560200
C	1.74575900	0.86105200	-1.55498700
C	0.15859200	2.44986800	-0.93906000
C	0.97264500	3.50279900	-1.35116100
C	2.22502800	3.19830600	-1.88501400
C	2.61906900	1.86450300	-1.98661700
N	0.53239900	1.16944700	-1.04339800
N	1.13346300	-1.41017900	-1.07051700
Cu	-0.51104700	-0.45884100	-0.27709700
S	0.50883900	-0.47172500	2.06338400
C	2.11343000	0.38213600	1.83914700
C	2.15590500	1.77952800	1.87447400
C	3.25027000	-0.35851200	1.50704700

C	3.35221500	2.43969900	1.58281100
C	4.44631100	0.30659200	1.21753400
C	4.49715100	1.70425300	1.25170100
O	0.90609600	-1.86936600	2.54062300
O	-0.19933500	0.36061800	3.13801000
H	0.55894400	-3.32499800	-0.58270200
H	2.66289200	-4.38380100	-1.44900700
H	4.42046700	-2.88560000	-2.45104100
H	4.01915300	-0.44228400	-2.52643600
H	-0.81916900	2.62352900	-0.50493400
H	0.63192900	4.52663900	-1.24710900
H	2.89422100	3.98786300	-2.21069500
H	3.59516200	1.61930300	-2.38524800
H	1.26010000	2.33804700	2.12692200
H	3.19157300	-1.44180400	1.47627300
H	3.38926000	3.52508700	1.60428700
H	5.33242200	-0.26556800	0.95744200
H	5.42361500	2.22001800	1.01608000
I	-2.42142000	-2.18011800	0.00336500
C	-2.39054000	0.27108700	-0.12868100
C	-2.59748600	0.97234500	1.06600900
C	-2.86269700	0.75048700	-1.35936000
C	-3.23246500	2.21663500	1.01508400
H	-2.22434200	0.58808600	2.00865100
C	-3.49553800	1.99492400	-1.40621400
H	-2.72392700	0.17576200	-2.26708000
C	-3.67015700	2.69428800	-0.21627000
H	-3.38441000	2.80462000	1.91379200
H	-3.85184800	2.40992900	-2.34265900
F	-4.29706700	3.90215800	-0.25940800

E(RB3LYP) -3258.46444549 a.u.
ν -80.50 cm^{-1}

(2,2′-Bipyridyl) 4-fluorophenyl(*S*-sulfinylphenyl)copper(III) iodide (**16e**)

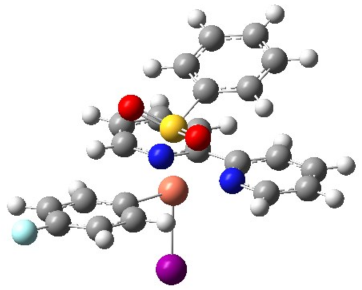

Geometry-optimised cartesian coordinates
Charge = 0 Multiplicity = 1

ATOM	X	Y	Z
C	2.24498400	1.16679800	-0.13627500
C	1.34315200	1.08463200	-2.29106000
C	2.50795900	1.58836900	-2.85971300
C	3.57596500	1.89242300	-2.01418900
C	3.44380200	1.68120600	-0.64343400
C	2.01805600	0.88834400	1.30616600
C	0.55373600	0.04375200	2.89102600
C	1.46532900	0.24652600	3.92720200
C	2.70444000	0.80559400	3.61352200
C	2.98689400	1.13114300	2.28759300
N	0.81684900	0.35802400	1.61774300
N	1.22094000	0.87882100	-0.97052000
Cu	-0.52636400	0.19896500	-0.23257000
S	-0.00638900	-2.02280300	-0.65161400
C	1.79776200	-2.14507100	-0.46172200
C	2.32744900	-2.37555000	0.80964100
C	2.61638200	-1.95908300	-1.57753800
C	3.71521100	-2.41608900	0.96422500
C	4.00251900	-2.00174800	-1.41054600
C	4.55076700	-2.22352000	-0.14186400
O	-0.28991800	-2.28504100	-2.10211100

O	-0.57051600	-2.98671000	0.34515300
H	0.47846000	0.83047800	-2.89380800
H	2.56895600	1.73387600	-3.93190300
H	4.50391900	2.28637600	-2.41520900
H	4.26977500	1.91016100	0.01710700
H	-0.41896300	-0.39420500	3.08617900
H	1.20597000	-0.02850300	4.94339800
H	3.44493200	0.98258300	4.38666900
H	3.94884100	1.55719900	2.03269800
H	1.66794300	-2.51684300	1.65822300
H	2.17470100	-1.78557300	-2.55205700
H	4.14051700	-2.59074900	1.94763400
H	4.65096600	-1.85619100	-2.26899500
H	5.62896300	-2.24595400	-0.01534600
I	-1.56658400	2.59724600	-0.16314000
C	-2.31983500	-0.49363400	-0.04230200
C	-3.04363200	-0.79102400	-1.19490100
C	-2.85248900	-0.71173100	1.22548200
C	-4.32837900	-1.33912900	-1.08205100
H	-2.61556200	-0.62274600	-2.17710000
C	-4.13575900	-1.25907200	1.35082600
H	-2.29595800	-0.45753100	2.11893500
C	-4.83968900	-1.55935600	0.19089700
H	-4.91564300	-1.59029400	-1.95909100
H	-4.57960200	-1.44284300	2.32361000
F	-6.09458900	-2.09278000	0.30833300

E(RB3LYP) -3258.47145376 a.u.

3.5. Reductive elimination from copper(III)

3.5.1. Reductive elimination pathways *via* an *O*-bound or *S*-bound copper(III) sulfinate

Geometry optimisations, followed by a frequency calculation were carried out for each species. The geometry-optimised molecular coordinates (Å) and energies (B3LYP/6-31G+(d,p) for C, H, N, O, S, Cu & SDD/ ECP46MWB for I) are shown below (solvent = *N,N*-dimethylformamide).

3.5.1.1. Geometry optimisations of common species to both pathways

(2,2'-Bipyridyl)copper(I) iodide

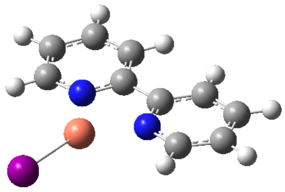

Geometry-optimised cartesian coordinates

Charge = 0 Multiplicity = 1

ATOM	X	Y	Z
C	2.24889300	-0.74598400	-0.00055300
C	2.24873300	0.74626900	0.00055200
C	3.42000400	-1.51236600	-0.03237300
N	1.03052400	-1.33444400	0.02793900
C	3.41968000	1.51290400	0.03234000
N	1.03023900	1.33446200	-0.02790700
C	3.32477100	-2.90416700	-0.02909700
C	0.94313000	-2.67277400	0.03063600
C	2.06435300	-3.50157100	0.00461300
C	3.32414200	2.90468400	0.02906500
C	0.94255000	2.67276900	-0.03060100
C	2.06359200	3.50181300	-0.00461000
Cu	-0.55683500	-0.00023100	0.00001800
I	-3.08073900	-0.00008200	-0.00001000
H	4.39465000	-1.04198800	-0.06402300
H	4.39442900	1.04273800	0.06396300
H	4.22469400	-3.51006600	-0.05379900
H	-0.06217400	-3.08060200	0.05469100
H	1.94563600	-4.57927100	0.00901500
H	4.22393400	3.51078000	0.05374100
H	-0.06284600	3.08037600	-0.05462600
H	1.94464100	4.57948700	-0.00900800

E(RB3LYP) -2147.32202298 a.u.

3.5.1.2. *O*-bound sulfinate pathway: Ground state and transition state geometry optimisations

(2,2'-Bipyridyl) *p*-tolyl(*O*-sulfinylphenyl)copper(III) iodide (**18**)

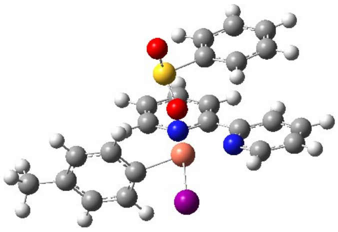

Geometry-optimised cartesian coordinates

Charge = 0 Multiplicity = 1

ATOM	X	Y	Z
C	-1.96532200	0.42139900	-1.53506500
N	-1.19442900	1.29218000	-0.84093800
Cu	0.59452300	0.53634200	-0.22501300
N	-0.19075900	-1.13347700	-1.11489700
C	-1.40216800	-0.93867000	-1.68785900

O	-0.11120500	0.20941700	1.85439500
S	-0.72623600	-1.16871800	2.26755500
C	-3.21571800	0.80127100	-2.02913500
C	-3.67885600	2.09344300	-1.78677700
C	-2.88663500	2.97303700	-1.05067500
C	-1.64606800	2.52836200	-0.59774500
C	0.36681500	-2.35171000	-1.12847000
C	-0.24646200	-3.44349400	-1.73773000
C	-1.47920000	-3.24991100	-2.36172300
C	-2.06477100	-1.98566000	-2.33530600
O	-0.91820800	-1.19392100	3.79287900
C	-2.43938600	-0.98924000	1.62999200
C	-3.10289100	0.23818200	1.71983700
C	-4.41099100	0.34877800	1.24709300
C	-5.04860000	-0.76221300	0.67671500
C	-4.37656400	-1.98448200	0.58324000
C	-3.06704000	-2.10049600	1.06615400
H	-3.82894800	0.10155800	-2.58169500
H	-4.65057600	2.40086000	-2.15873100
H	-3.21291300	3.98227200	-0.82715000
H	-0.98418700	3.17120400	-0.02859500
H	1.32746800	-2.44214100	-0.63651600
H	0.23733000	-4.41322800	-1.72034500
H	-1.98597000	-4.07311400	-2.85432400
H	-3.03006500	-1.82649200	-2.79821200
H	-2.58118300	1.09703600	2.13100700
H	-4.92796300	1.30254500	1.30212200
H	-6.06171500	-0.66918900	0.29617200
H	-4.86441400	-2.84267800	0.12981800
H	-2.53426200	-3.04515400	0.98473000
I	1.98312500	2.63761500	0.13884200
C	2.38984000	-0.19279000	-0.07935700
C	3.08836500	-0.41541600	-1.26301900
C	2.73876700	-0.80419100	1.11862000
C	4.11966600	-1.36243800	-1.25545900
H	2.82859300	0.10951600	-2.17548400
C	3.77823400	-1.74464000	1.10051600
H	2.19932900	-0.57473100	2.02868200
C	4.47649500	-2.04306500	-0.07991800
H	4.65508800	-1.56307300	-2.18023600
H	4.04530800	-2.24711800	2.02711600
C	5.57393700	-3.08158600	-0.08997400
H	5.16508800	-4.07397400	-0.31790300
H	6.32972200	-2.85663900	-0.84849900
H	6.07020400	-3.14719800	0.88285700

E(RB3LYP) -3198.55271298 a.u.

<u>(2,2'-Bipyridyl) *p*-tolyl(*O*-sulfinylphenyl)copper(III) iodide reductive elimination transition state</u>‡ (<u>yielding sulfinic acid ester product</u>)

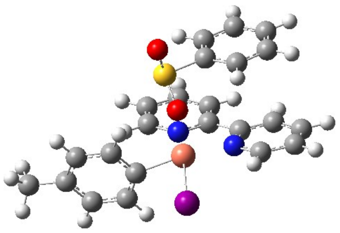

Geometry-optimised cartesian coordinates

Charge = 0 Multiplicity = 1

ATOM	X	Y	Z
C	1.82785800	-1.85657400	-0.79269700
N	1.63249700	-0.86878500	0.10614100
Cu	-0.29310300	-0.11679300	0.14439900
N	-0.50573400	-1.54913400	-1.28573700
C	0.62255100	-2.24509800	-1.56834500
O	-0.26383600	1.71940000	0.75144300
S	0.23082500	3.09982600	-0.04525100
C	3.07911800	-2.46728200	-0.92921600

C	4.12806500	-2.04809500	-0.11035800
C	3.90584900	-1.03647000	0.82497200
C	2.63397400	-0.47318300	0.89780800
C	-1.65514200	-1.86306800	-1.89804600
C	-1.74463300	-2.88421700	-2.84112600
C	-0.58727200	-3.59749400	-3.15593700
C	0.60783300	-3.27680800	-2.51272300
O	0.32055600	4.14030500	1.04426300
C	1.94564700	2.61789000	-0.40488100
C	2.97965300	3.00884500	0.44730500
C	4.28948500	2.63472700	0.13656000
C	4.55170700	1.88736100	-1.01811800
C	3.50720200	1.51418600	-1.87068800
C	2.19436200	1.88391200	-1.56855000
H	3.24002700	-3.25666100	-1.65278500
H	5.10592700	-2.50909100	-0.20356100
H	4.69535200	-0.68308600	1.47792600
H	2.39790000	0.32299700	1.59503000
H	-2.51766900	-1.26870400	-1.61880300
H	-2.69592000	-3.10628600	-3.31096400
H	-0.61196400	-4.39723300	-3.88886200
H	1.51039700	-3.82882600	-2.74283600
H	2.75309300	3.59510000	1.33227400
H	5.10346700	2.92386100	0.79448100
H	5.57063400	1.59447500	-1.25243200
H	3.71145100	0.93292800	-2.76446400
H	1.38112800	1.59154200	-2.22707800
I	-0.85367600	-1.62868100	2.58379100
C	-1.90156500	1.01475100	-0.11648100
C	-2.11235100	1.37744600	-1.44272600
C	-2.91556400	0.99689100	0.83606200
C	-3.43451000	1.59933100	-1.85372900
H	-1.29897300	1.44146800	-2.15518900
C	-4.22113800	1.21931200	0.39341600
H	-2.69512800	0.78662700	1.87429300
C	-4.50426300	1.52034600	-0.95199900
H	-3.61930500	1.83950700	-2.89740400
H	-5.03128500	1.17329100	1.11683700
C	-5.92740600	1.74371900	-1.40508500
H	-6.45408600	0.78740800	-1.51325900
H	-6.48655400	2.34091200	-0.67751400
H	-5.96476400	2.25483700	-2.37116600

E(RB3LYP) -3198.53479916 a.u.
ν -275.38 cm^{-1}

Phenyl 4-methylbenzenesulfinate (**19**)

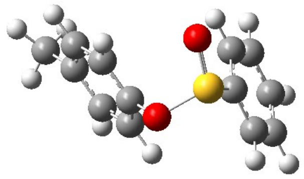

Geometry-optimised cartesian coordinates
Charge = 0 Multiplicity = 1

ATOM	X	Y	Z
C	-2.70143400	1.97851000	0.80268700
C	-2.49446900	0.60153800	0.90926500
C	-1.83859500	-0.06417600	-0.13052900
C	-1.39598900	0.60647400	-1.27155000
C	-1.60666500	1.98402400	-1.36644400
C	-2.25561400	2.66731900	-0.33169500
S	-1.54926400	-1.84205000	0.04514600
O	-0.17706300	-1.76064500	1.11449800
O	-1.07680500	-2.31036400	-1.30259700
C	0.90523100	-0.97405400	0.72193500
C	1.08950200	0.27158600	1.32719000
C	2.18979100	1.04989800	0.96688800
C	3.10532200	0.61166100	-0.00465100

C	2.89365200	-0.64261500	-0.59713800
C	1.80225100	-1.43997700	-0.24110800
C	4.27008400	1.48216500	-0.41535900
H	-3.20811400	2.51117800	1.60120200
H	-2.82993200	0.06080300	1.78987100
H	-0.89546100	0.05425300	-2.05987300
H	-1.26360200	2.52249900	-2.24437200
H	-2.41789000	3.73796200	-0.41045200
H	0.37487300	0.61569000	2.06692200
H	2.33663400	2.01505700	1.44464600
H	3.59345700	-1.00647000	-1.34481400
H	1.64000400	-2.40975300	-0.69690400
H	5.07845000	0.88909600	-0.85285000
H	3.95862500	2.21975900	-1.16548700
H	4.67369700	2.03668100	0.43762400

E(RB3LYP) -1051.27648103 a.u.

3.5.1.3. *S*-bound sulfinate pathway: Ground state and transition state geometry optimisations

(2,2′-Bipyridyl) *p*-tolyl(*S*-sulfinylphenyl)copper(III) iodide (**16b**)

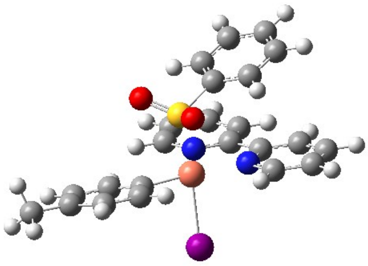

Geometry-optimised cartesian coordinates

Charge = 0 Multiplicity = 1

ATOM	X	Y	Z
C	2.27495800	1.14242700	-0.10055500
C	1.39411300	1.11205300	-2.26448000
C	2.57217700	1.60741800	-2.81309400
C	3.63656100	1.87899000	-1.95218100
C	3.48703300	1.64604000	-0.58678800
C	2.02990100	0.84389700	1.33505800
C	0.54088200	-0.00984700	2.89176900
C	1.44508000	0.16376100	3.93967800
C	2.69329400	0.71402700	3.64719700
C	2.99215600	1.05848100	2.32967800
N	0.81910700	0.32416100	1.62672200
N	1.25498000	0.88432000	-0.94933400
Cu	-0.51320900	0.21502000	-0.23796400
S	-0.01867000	-2.00937600	-0.68852500
C	1.78401600	-2.15654100	-0.48995800
C	2.30412500	-2.41316100	0.78038300
C	2.61223800	-1.96331400	-1.59741700
C	3.69040700	-2.47158000	0.94292800
C	3.99689100	-2.02420400	-1.42314400
C	4.53505200	-2.27157400	-0.15490300
O	-0.29224900	-2.25051100	-2.14587500
O	-0.59622000	-2.98871300	0.28701800
H	0.53159700	0.88314300	-2.88040600
H	2.64602400	1.77094200	-3.88189900
H	4.57479700	2.26465600	-2.33707600
H	4.31005900	1.84964600	0.08556600
H	-0.43864300	-0.44001100	3.06964400
H	1.17317900	-0.12619500	4.94844000
H	3.42839000	0.86941800	4.43010600
H	3.96138300	1.47775800	2.09147600
H	1.63791900	-2.56037200	1.62274500
H	2.17843700	-1.76970400	-2.57173100
H	4.10765500	-2.66619900	1.92609600
H	4.65220400	-1.87277500	-2.27539700
H	5.61215900	-2.30812800	-0.02229600

I	-1.52448700	2.62608900	-0.15740600
C	-2.31842900	-0.45643900	-0.05514800
C	-3.04232100	-0.74675900	-1.20647700
C	-2.86089400	-0.65627700	1.20932600
C	-4.33385700	-1.27670400	-1.08140600
H	-2.61108800	-0.58692100	-2.18927700
C	-4.15245500	-1.18744400	1.31799600
H	-2.30974800	-0.39626600	2.10513100
C	-4.90619200	-1.50859900	0.17838800
H	-4.89786300	-1.51067300	-1.98155100
H	-4.57708900	-1.34318200	2.30720700
C	-6.28693700	-2.11139100	0.30649600
H	-6.90891900	-1.87328000	-0.56184200
H	-6.22998100	-3.20477400	0.37967500
H	-6.79849400	-1.75051900	1.20434400

E(RB3LYP) -3198.55386175 a.u.

(2,2′-Bipyridyl) *p*-tolyl(*S*-sulfinylphenyl)copper(III) iodide reductive elimination transition state‡ (yielding sulfone product)

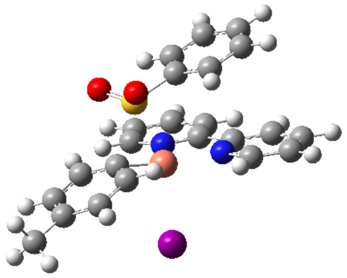

Geometry-optimised cartesian coordinates

Charge = 0 Multiplicity = 1

**ATOM**	**X**	**Y**	**Z**
C	-2.29176900	-1.21619400	-0.32066400
C	-1.34381100	-0.90890400	-2.42696200
C	-2.45887200	-1.43806900	-3.07021400
C	-3.53090100	-1.86564500	-2.28544900
C	-3.44721700	-1.75618700	-0.89867200
C	-2.10368900	-1.07469700	1.14979000
C	-0.69296300	-0.35705700	2.85087500
C	-1.60477000	-0.72058100	3.84059000
C	-2.81557100	-1.28903400	3.44248300
C	-3.06856500	-1.47132200	2.08346700
N	-0.93431600	-0.52663500	1.54525400
N	-1.26705100	-0.79445700	-1.09330000
Cu	0.39894900	-0.08453400	-0.12093000
S	0.11660900	2.37375300	0.03050400
C	-1.67537100	2.20261700	-0.23970300
C	-2.52344000	2.10459600	0.86355900
C	-2.15602800	2.18351200	-1.55113600
C	-3.89753700	1.98190300	0.64131700
C	-3.52980300	2.05309800	-1.75763200
C	-4.39825800	1.94765500	-0.66406700
O	0.56469200	3.25663900	-1.08394100
O	0.25353700	2.83765200	1.44009200
H	-0.48017000	-0.55930000	-2.98231900
H	-2.48137300	-1.50758700	-4.15176600
H	-4.42260300	-2.27987000	-2.74445800
H	-4.27431800	-2.08685100	-0.28380000
H	0.26155200	0.09088900	3.10474000
H	-1.36788700	-0.55980500	4.88628200
H	-3.55615000	-1.58666000	4.17765800
H	-4.00624700	-1.90971500	1.76657000
H	-2.11650200	2.12254000	1.86733000
H	-1.46989300	2.26444600	-2.38646700
H	-4.57083500	1.90301200	1.48904900
H	-3.91968100	2.02784700	-2.77024100
H	-5.46531700	1.83730900	-0.83114500
I	1.71452700	-2.51542000	-0.24081300
C	1.97624000	1.06556200	-0.01927200
C	2.64353900	1.15305500	-1.24186800

C	2.65194200	1.10119600	1.19748400
C	4.03856100	1.16905400	-1.23609100
H	2.09001600	1.19496900	-2.17357000
C	4.04851400	1.12508800	1.18226700
H	2.10551300	1.11054100	2.13298000
C	4.75976600	1.15288400	-0.02895000
H	4.57282700	1.20245900	-2.18208200
H	4.58882500	1.12709400	2.12530800
C	6.26790100	1.21754200	-0.03671600
H	6.60909700	2.25861400	-0.09636500
H	6.69068900	0.78383500	0.87402800
H	6.68359500	0.68783800	-0.89924600

E(RB3LYP) -3198.54252954 a.u.
ν -151.83 cm^{-1}

(*p*-Tolyl)sulfonylbenzene (**3**)

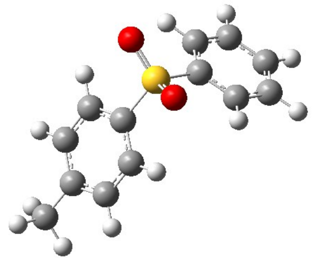

Geometry-optimised cartesian coordinates
Charge = 0 Multiplicity = 1

ATOM	X	Y	Z
C	-3.18111400	-1.42988100	1.21520600
C	-2.24904700	-0.38977200	1.22216400
C	-1.79547300	0.11287000	-0.00026600
C	-2.24638000	-0.39414500	-1.22190100
C	-3.17842100	-1.43424200	-1.21327800
C	-3.64241200	-1.95143000	0.00139300
S	-0.56505800	1.42488300	-0.00137100
C	1.01629200	0.58033300	-0.00022400
C	1.61149800	0.24513700	1.21846800
C	2.82681700	-0.43901600	1.20860100
C	3.45287500	-0.79146400	0.00154900
C	2.82703300	-0.44143500	-1.20657400
C	1.61193700	0.24259400	-1.21823400
O	-0.68859000	2.15761100	-1.28320300
O	-0.68913300	2.16035200	1.27883000
C	4.78311800	-1.50338000	0.00127600
H	-3.54775100	-1.82846300	2.15576900
H	-1.88826200	0.03081900	2.15430600
H	-1.88363400	0.02313500	-2.15477500
H	-3.54296800	-1.83621700	-2.15320900
H	-4.36684100	-2.76000400	0.00204000
H	1.13694500	0.52040900	2.15398700
H	3.29609000	-0.70072500	2.15265300
H	3.29667700	-0.70526400	-2.14987700
H	1.13758300	0.51599200	-2.15440100
H	5.60450100	-0.77680800	-0.02625600
H	4.91133500	-2.11088000	0.90158600
H	4.88868800	-2.15084400	-0.87397100

E(RB3LYP) -1051.27237794 a.u.

3.5.2. Reductive elimination of (*p*-tolyl)sulfonylbenzene from (L)Cu(Tol)(SO_2Ph)(I): Variation of $\Delta E_{RE}^{‡}$ with complex ligand

Geometry optimisations, followed by a frequency calculation were carried out for each species. The geometry-optimised molecular coordinates (Å) and energies (B3LYP/6-31G+(d,p) for C, H, N, O, S, Cu & SDD/ ECP46MWB for I) are shown below (solvent = *N,N*-dimethylformamide).

3.5.2.1. Geometry optimisations of common species to all pathways

(*p*-Tolyl)sulfonylbenzene (**3**)

See section 3.5.1.3 for the previously reported geometry-optimised structure of (*p*-tolyl)sulfonylbenzene (**3**).

3.5.2.2. Ligand: None

p-Tolyl(*S*-sulfinylphenyl)copper(III) iodide (**16a**)

See section 3.3.1.2 for the previously reported geometry-optimised structure of *p*-tolyl(*S*-sulfinylphenyl)copper(III) iodide (**16a**).

p-Tolyl(*S*-sulfinylphenyl)copper(III) iodide reductive elimination transition state‡

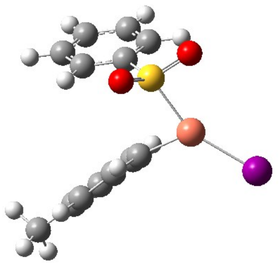

Geometry-optimised cartesian coordinates

Charge = 0 Multiplicity = 1

ATOM	X	Y	Z
Cu	-1.07430100	-0.18394800	0.25227400
S	0.70742600	-1.20631500	1.24230300
C	0.43731000	1.02312400	0.27129400
I	-3.46625200	-0.16343900	-0.48671200
C	2.11850500	-1.56957800	0.19954600
C	1.03542800	1.21432500	-0.97287500
C	1.63136000	2.45332300	-1.22425500
C	1.66304000	3.45883400	-0.24273400
C	1.08846100	3.19809400	1.01288000
C	0.48441800	1.96772900	1.29140400
C	3.33876400	-0.95370200	0.48692200
C	4.42027000	-1.21266300	-0.35614600
C	4.26252700	-2.05418900	-1.46456800
C	3.02594600	-2.65018100	-1.73682100
C	1.93275900	-2.40824000	-0.90161900
O	1.17159000	-0.74577100	2.56744500
O	-0.22001400	-2.38276100	1.17817700
C	2.30020600	4.79497300	-0.53592000
H	1.04274300	0.43457700	-1.72677800
H	2.08708200	2.62985300	-2.19473300
H	1.12186800	3.95635700	1.79042900
H	0.07821000	1.75351600	2.27399600
H	3.43650100	-0.29678200	1.34394200
H	5.38242700	-0.75536100	-0.14923600
H	5.10712300	-2.24473500	-2.11942700
H	2.90946300	-3.30210000	-2.59646900
H	0.96604900	-2.85983200	-1.09482500
H	1.56731700	5.47328000	-0.99025900
H	2.66887000	5.27149300	0.37667100
H	3.13305700	4.69469300	-1.23795900

E(RB3LYP) -2703.06467181 a.u.
ν -109.76 cm^{-1}

Copper(I) iodide

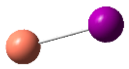

Geometry-optimised cartesian coordinates

Charge = 0 Multiplicity = 1

**ATOM**	**X**	**Y**	**Z**
Cu	0.00000000	0.00000000	-1.59942300
I	0.00000000	0.00000000	0.87515600

E(RB3LYP) -1651.82615553 a.u.

3.5.2.3. Ligand: 2,2'-Bipyridine (bpy)

See section 3.5.1.3 for the previously reported geometry-optimised structures of (2,2'-bipyridyl) *p*-tolyl(*S*-sulfinylphenyl)copper(III) iodide (**16b**) and (2,2'-bipyridyl) *p*-tolyl(*S*-sulfinylphenyl)copper(III) iodide reductive elimination transition state‡. Additionally see section 3.5.1.1 for the previously reported geometry-optimised structure of (2,2'-bipyridyl)copper(I) iodide.

3.5.2.4. Ligand: 4,4'-Dimethoxy-2,2'-bipyridine (4,4'-diMeObpy)

(4,4'-Dimethoxy-2,2'-bipyridyl) *p*-tolyl(*S*-sulfinylphenyl)copper(III) iodide (**16c**)

See section 3.3.1.4 for the previously reported geometry-optimised structure of (4,4'-dimethoxy-2,2'-bipyridyl) *p*-tolyl(*S*-sulfinylphenyl)copper(III) iodide (**16c**).

(4,4'-Dimethoxy-2,2'-bipyridyl) *p*-tolyl(*S*-sulfinylphenyl)copper(III) iodide reductive elimination transition state‡

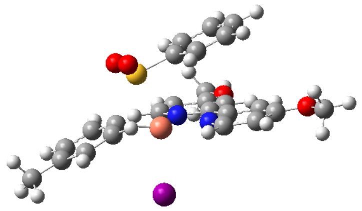

Geometry-optimised cartesian coordinates

Charge = 0 Multiplicity = 1

**ATOM**	**X**	**Y**	**Z**
C	1.99492200	-0.43359000	-0.68951300
C	0.99183900	-2.52184900	-0.54922900
C	2.18060000	-3.19053900	-0.81608400
C	3.33281000	-2.41612100	-1.01688500
C	3.22640600	-1.01759400	-0.96027500
C	1.79760600	1.04238800	-0.61146000
C	0.31696800	2.76284600	-0.16273300
C	1.27660700	3.74549600	-0.38796900
C	2.56464600	3.32549700	-0.75160500
C	2.81944400	1.94941400	-0.86651700
N	0.55314400	1.45096900	-0.26497200
N	0.89091000	-1.18895100	-0.47206100
Cu	-0.84680200	-0.19439300	-0.03824100
S	-0.92635200	-0.04231700	2.44333000
C	0.87721900	-0.27956800	2.54608600
C	1.70945600	0.83906700	2.58974300
C	1.38060100	-1.58150400	2.59499900
C	3.08974600	0.64279800	2.68573700
C	2.76139900	-1.76238800	2.68305500
C	3.61464300	-0.65275100	2.72403500
O	-1.48221400	-1.17874400	3.23333600
O	-1.15293200	1.35661600	2.90609800
H	0.07527700	-3.07706200	-0.38052200
H	2.18797600	-4.27149400	-0.85401300
H	4.11995900	-0.42830700	-1.11868700
H	-0.69168300	3.04522200	0.11940900
H	1.01019100	4.78841700	-0.28028400
H	3.81564100	1.63351100	-1.14794800
H	1.28630600	1.83533800	2.54732700
H	0.70672500	-2.42978800	2.56062400
H	3.75053700	1.50333500	2.71850500
H	3.16941900	-2.76775100	2.71278700

H	4.68873500	-0.79988700	2.78268200
I	-1.81065800	-0.30744000	-2.63758700
C	-2.56830600	-0.07488400	0.87726100
C	-3.24977500	-1.29259700	0.86350000
C	-3.23365700	1.14644300	0.80677900
C	-4.63093900	-1.27805300	0.66496000
H	-2.71527400	-2.22727000	0.99320000
C	-4.61698700	1.14023200	0.61549200
H	-2.68850800	2.07810500	0.89971800
C	-5.33238700	-0.06656100	0.53493100
H	-5.17032400	-2.22056100	0.61809600
H	-5.14491500	2.08666300	0.53258700
C	-6.83258000	-0.06309000	0.36658300
H	-7.17109800	-0.93574800	-0.20004500
H	-7.33047100	-0.09350400	1.34384600
H	-7.17411400	0.83933400	-0.14866800
O	3.60341100	4.14410600	-1.00514000
O	4.56214100	-2.90670400	-1.26294400
C	3.40602000	5.56602100	-0.91147200
H	3.11421400	5.85113200	0.10450600
H	4.37008800	6.00968400	-1.15655600
H	2.65043000	5.90043500	-1.62975900
C	4.74193300	-4.33276000	-1.32647800
H	5.80201100	-4.48277800	-1.52649300
H	4.47462500	-4.79989600	-0.37311900
H	4.14698800	-4.76281700	-2.13856700

E(RB3LYP) -3427.61964124 a.u.
ν -150.88 cm^{-1}

<u>(4,4’-Dimethoxy-2,2’-bipyridyl)copper(I) iodide</u>

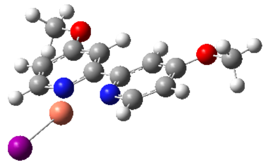

Geometry-optimised cartesian coordinates
Charge = 0 Multiplicity = 1

**ATOM**	**X**	**Y**	**Z**
C	1.51154700	0.74699200	-0.00457000
C	0.21858900	2.67067200	-0.04721000
C	1.33249400	3.50573000	-0.02854100
C	2.59821300	2.90308300	0.00727800
C	2.67941900	1.50053700	0.02053600
C	1.51117600	-0.74749100	0.00444800
C	0.21719200	-2.67048100	0.04666200
C	1.33066300	-3.50611800	0.02815000
C	2.59672000	-2.90414600	-0.00727100
C	2.67866200	-1.50164000	-0.02044000
N	0.28663500	-1.33266200	0.03558900
N	0.28730800	1.33280900	-0.03597500
Cu	-1.29674500	0.00048700	-0.00011600
O	3.77102800	3.56455700	0.03209500
C	3.75741000	5.00287500	0.01889500
O	3.76915800	-3.56625200	-0.03179800
C	3.75471800	-5.00458700	-0.01846900
H	-0.78111900	3.09232900	-0.07274800
H	1.19630500	4.57884400	-0.04008100
H	3.66063000	1.04476100	0.05429200
H	-0.78275100	-3.09161400	0.07195500
H	1.19392800	-4.57916600	0.03945500
H	3.66009600	-1.04633500	-0.05395500
H	4.80396700	5.30354300	0.04433400
H	3.28606800	5.37774400	-0.89543600
H	3.23769400	5.39396500	0.89955200
H	4.80112400	-5.30582600	-0.04330800
H	3.28265100	-5.37907500	0.89564000
H	3.23526800	-5.39542700	-0.89939000
I	-3.82355000	0.00060500	0.00012400

E(RB3LYP) -2376.39817933 a.u.

3.5.2.5. Ligand: 4,4'-Dinitro-2,2'-bipyridine (4,4'-diNO_2bpy)

(4,4'-Dinitro-2,2'-bipyridyl) *p*-tolyl(*S*-sulfinylphenyl)copper(III) iodide (**16d**)

See section 3.3.1.5 for the previously reported geometry-optimised structure of (4,4'-dinitro-2,2'-bipyridyl) *p*-tolyl(*S*-sulfinylphenyl)copper(III) iodide (**16d**).

(4,4'-Dinitro-2,2'-bipyridyl) *p*-tolyl(*S*-sulfinylphenyl)copper(III) iodide reductive elimination transition state‡

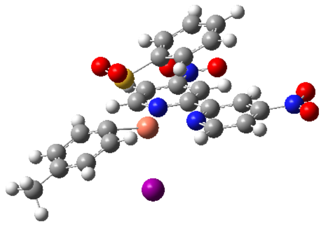

Geometry-optimised cartesian coordinates

Charge = 0 Multiplicity = 1

ATOM	X	Y	Z
C	1.81994000	-0.51522200	-0.54135200
N	0.67255100	-1.21503000	-0.42564400
C	0.67835100	-2.54626700	-0.57242300
C	1.84177900	-3.27121500	-0.81669100
C	3.02050700	-2.54152000	-0.90456600
C	3.03856800	-1.15997600	-0.77641000
Cu	-1.07126200	-0.13444100	-0.05497900
N	0.47249700	1.43313600	-0.12295900
C	1.70407700	0.96117800	-0.40282600
C	2.79929300	1.81894100	-0.54514000
C	2.57140900	3.17849200	-0.38107300
C	1.30887100	3.68160600	-0.09493800
C	0.27954500	2.74796200	0.02310400
N	4.29862500	-3.25306800	-1.13318600
N	3.71298700	4.11392700	-0.51129200
O	3.48549400	5.31323600	-0.36245800
O	4.25390800	-4.47518300	-1.26359100
S	-1.30611000	-0.04127500	2.40899100
C	0.45783200	-0.44498700	2.61897100
O	-1.43864900	1.38725200	2.80745300
O	-2.00735300	-1.09709300	3.19029700
C	1.38305900	0.59022900	2.75731100
C	2.73324800	0.26450300	2.91420300
C	3.13751800	-1.07443700	2.92073000
C	2.19083800	-2.09918100	2.79534200
C	0.83821100	-1.78922100	2.64732300
O	4.81956100	3.63634800	-0.75794600
O	5.32863900	-2.58142900	-1.17462200
H	-0.28018900	-3.04366400	-0.48267400
H	1.82607400	-4.34763100	-0.92152200
H	3.97511600	-0.62668500	-0.85251400
H	3.79564200	1.46488300	-0.76792700
H	1.13118600	4.74076900	0.03461500
H	-0.73046000	3.06573900	0.25260300
H	1.05303600	1.62200400	2.74414600
H	3.46553200	1.05854500	3.02059600
H	4.18942000	-1.32140500	3.02523400
H	2.50438700	-3.13820000	2.80770700
H	0.09419700	-2.57187500	2.55025600
C	-2.86131200	-0.05785000	0.72753100
C	-3.53861700	-1.27577600	0.63596600
C	-3.52050800	1.16533600	0.67697700
C	-4.90843000	-1.25048300	0.37885300
H	-3.01221700	-2.21664400	0.75299400
C	-4.89658400	1.16778100	0.42750800
H	-2.98057500	2.09218400	0.83105200
C	-5.60536000	-0.03263400	0.26569700

H	-5.44594300	-2.18963200	0.27561500
H	-5.42087300	2.11755200	0.36598200
I	-1.76805000	-0.03716600	-2.69653100
C	-7.08870700	-0.03071800	-0.01328600
H	-7.28836900	-0.38011300	-1.03342400
H	-7.61958900	-0.70413200	0.66806400
H	-7.51591100	0.97022300	0.08948600

E(RB3LYP) -3607.55012412 a.u.
ν -154.01 cm^{-1}

(4,4'-Dinitro-2,2'-bipyridyl)copper(I) iodide

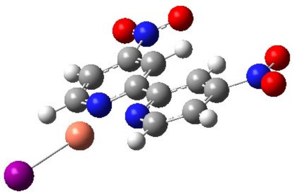

Geometry-optimised cartesian coordinates
Charge = 0 Multiplicity = 1

**ATOM**	**X**	**Y**	**Z**
C	-1.22268700	0.74481600	0.01126400
N	-0.00601800	1.33089200	0.06619200
C	0.09031200	2.66651200	0.09012400
C	-1.02384200	3.50533200	0.06131100
C	-2.26576200	2.88664200	0.00033800
C	-2.39593400	1.50369100	-0.02731000
Cu	1.59306900	0.00065700	0.00097000
N	-0.00538700	-1.33055000	-0.06385600
C	-1.22234500	-0.74502800	-0.01027200
C	-2.39529300	-1.50444100	0.02709200
C	-2.26443400	-2.88732400	-0.00017000
C	-1.02215800	-3.50546700	-0.05961400
C	0.09162800	-2.66613200	-0.08744500
N	-3.48931500	3.71962900	-0.04021800
N	-3.48769200	-3.72089700	0.03910400
O	-4.57249200	-3.14402900	0.10708800
O	-3.34848600	4.94086200	0.00002900
O	-3.34621000	-4.94211700	0.00118400
O	-4.57365400	3.14228900	-0.11154100
H	1.09397900	3.07349100	0.13475000
H	-0.92258700	4.58211800	0.08361100
H	-3.37878000	1.05752200	-0.08302800
H	-3.37840300	-1.05873000	0.08163900
H	-0.92040000	-4.58221100	-0.08155300
H	1.09554900	-3.07260900	-0.13098800
I	4.10642200	0.00038100	-0.00088900

E(RB3LYP) -2556.32942272 a.u.

3.5.2.6. Ligand: *N,N'*-Dimethylethylenediamine (DMEDA)

(*N,N'*-Dimethylethylenediamine) *p*-tolyl(*S*-sulfinylphenyl)copper(III) iodide (**16e**)

See section 3.3.1.6 for the previously reported geometry-optimised structure of (*N,N'*-dimethylethylenediamine) *p*-tolyl(*S*-sulfinylphenyl)copper(III) iodide (**16e**).

(*N,N'*-Dimethylethylenediamine) *p*-tolyl(*S*-sulfinylphenyl)copper(III) iodide reductive elimination transition state‡

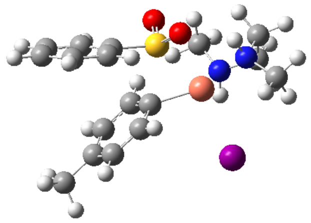

Geometry-optimised cartesian coordinates

Charge = 0 Multiplicity = 1

ATOM	X	Y	Z
C	3.23798800	-1.60273400	1.35108300
N	2.10498700	-0.80801700	1.87409700
Cu	0.97629800	-0.40777200	-0.07070800
N	2.10192900	-2.01154900	-0.79896300
C	2.72441200	-2.66519300	0.38001500
C	1.48680800	-1.40976600	3.06984400
C	3.06943000	-1.71077600	-1.87645900
S	-1.09145600	-1.68800400	-0.06474500
C	-2.84001200	-1.28161400	-0.33503200
C	-3.33986800	-1.28134300	-1.63826000
C	-4.68534500	-0.96058600	-1.83806500
C	-5.50272400	-0.64536300	-0.74700600
C	-4.98154300	-0.65503800	0.55229800
C	-3.63802400	-0.97047500	0.76710000
O	-0.72909400	-2.51988500	-1.25885400
O	-1.00010400	-2.30473500	1.28877000
H	3.90855200	-0.91900100	0.82318600
H	3.80832400	-2.07785800	2.16116100
H	3.54065200	-3.33167900	0.07396400
H	1.95693300	-3.27349400	0.86716200
H	0.98114000	-2.33610300	2.79548700
H	2.22869000	-1.61551900	3.85441900
H	0.73602300	-0.72798300	3.47345600
H	3.56186600	-2.62469600	-2.23164700
H	2.54010600	-1.23312900	-2.70288600
H	3.82096200	-1.01047300	-1.51151500
H	-2.69351100	-1.52999200	-2.47219300
H	-5.09164600	-0.95772900	-2.84475500
H	-6.54640200	-0.39311200	-0.90836300
H	-5.61775800	-0.41567700	1.39871400
H	-3.21884600	-0.97615600	1.76669800
H	1.39115900	-2.63377400	-1.18347900
H	2.44353300	0.12204900	2.11005200
C	-0.69636900	0.58374500	0.21132400
C	-1.17910100	1.32533000	-0.87173800
C	-0.99122300	0.93225300	1.52616100
C	-1.88193300	2.49933800	-0.61418900
H	-0.99428400	1.00370400	-1.89110300
C	-1.69547300	2.11729100	1.76511800
H	-0.68310200	0.29654200	2.34642300
C	-2.15006500	2.91498300	0.70483600
H	-2.23803000	3.09818500	-1.44863000
H	-1.90680500	2.40907600	2.79038200
C	-2.91883300	4.18859900	0.95774200
H	-2.30755600	5.06383800	0.70624200
H	-3.81985600	4.23545300	0.33684800
H	-3.21721300	4.27696800	2.00576300
I	2.58196500	1.78200100	-0.66382700

E(RB3LYP) -2972.29432593 a.u.

ν -179.40 cm^{-1}

(*N,N'*-Dimethylethylenediamine)copper(I) iodide

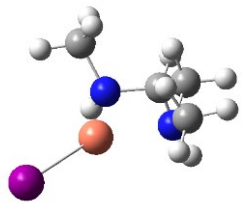

Geometry-optimised cartesian coordinates

Charge = 0 Multiplicity = 1

ATOM	X	Y	Z
C	3.04081800	-0.67566100	0.06086300
N	1.80209400	-1.42136400	-0.25401200
Cu	0.27192700	0.05677800	-0.19788500
N	1.84815400	1.46914900	-0.29556300
C	3.04114200	0.66997900	-0.66728200
C	1.59928400	-2.59928600	0.61251200
C	1.99674800	2.19612500	0.98387000
H	3.94089000	-1.24495200	-0.20855900
H	3.06338000	-0.51983900	1.14402500
H	3.97276700	1.20986700	-0.44778800
H	2.99831400	0.49768300	-1.74760100
H	1.44292300	-2.26089200	1.64002700
H	0.70335100	-3.13068400	0.28468700
H	2.45669300	-3.28640700	0.59107800
H	2.07534900	1.48118200	1.80572000
H	1.10451500	2.80337500	1.14888700
H	2.88543400	2.84236600	0.99034100
H	1.86269000	-1.74464500	-1.21964000
H	1.67582800	2.15121700	-1.03019200
I	-2.25825900	0.03158000	0.01311200

E(RB3LYP) -1921.07623127 a.u.

3.5.2.7. Ligand: L-Prolinate

(L-Prolinate) *p*-tolyl(*S*-sulfinylphenyl)copper(III) iodide (**16f**)

See section 3.3.1.7 for the previously reported geometry-optimised structure of (L-Prolinate) *p*-tolyl(*S*-sulfinylphenyl)copper(III) iodide (**16f**).

(L-Prolinate) *p*-tolyl(*S*-sulfinylphenyl)copper(III) iodide reductive elimination transition state‡

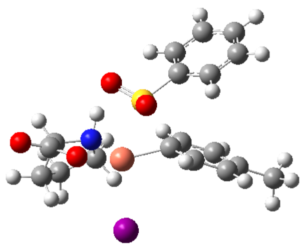

Geometry-optimised cartesian coordinates

Charge = -1 Multiplicity = 1

ATOM	X	Y	Z
C	-2.53963400	-2.18568800	0.79843000
N	-1.37632100	-1.38449300	1.26785300
Cu	-0.80512000	-0.18322000	-0.48556300
O	-2.02897800	-1.41960500	-1.44742100
C	-2.65527100	-2.28597200	-0.73440500
O	-3.39514500	-3.18133400	-1.19308900
C	-1.76911400	-0.62484600	2.49562600
C	-3.21897700	-1.04256400	2.79589600
C	-3.74942100	-1.45830600	1.41654400
S	1.26338900	-1.48339400	-0.91919700
C	2.96398500	-1.39774100	-0.28569700
C	3.26707800	-2.03371500	0.91955500

C	4.57035400	-1.94414900	1.41658900
C	5.54289700	-1.22467100	0.71335200
C	5.21933300	-0.59545400	-0.49471000
C	3.91991500	-0.67487600	-1.00141400
O	0.71093000	-2.73418400	-0.30912400
O	1.35923600	-1.38374600	-2.40591600
H	-2.48833400	-3.20843800	1.19042900
H	-1.08322400	-0.84319900	3.31915800
H	-1.71120800	0.44532800	2.27299400
H	-3.23856800	-1.90003300	3.47886300
H	-3.79674500	-0.23255000	3.25098500
H	-3.98415600	-0.57010300	0.81872300
H	-4.63264500	-2.09950800	1.45654600
H	2.50257600	-2.58969100	1.45061500
H	4.82314200	-2.43626200	2.35077900
H	6.55306000	-1.15485000	1.10548800
H	5.97570700	-0.04144000	-1.04214600
H	3.65279300	-0.18774500	-1.93223100
H	-0.59523600	-2.00825500	1.44621200
C	0.90986700	0.65860500	-0.12257900
C	1.25234600	0.85266800	1.21961300
C	1.37150400	1.51650200	-1.12244700
C	1.98161300	1.98876900	1.56671600
H	0.94118500	0.14600100	1.98018700
C	2.09327300	2.65456600	-0.75357100
H	1.16374400	1.30278100	-2.16557300
C	2.40942800	2.90822100	0.59040500
H	2.22904100	2.16107100	2.61128000
H	2.42951000	3.34198700	-1.52543500
I	-2.41198800	2.06559700	-0.43474200
C	3.18808200	4.13837700	0.98859800
H	3.73001300	4.56296000	0.13888100
H	2.51268100	4.91307800	1.37282500
H	3.90861700	3.91395900	1.78161900

E(RB3LYP) -3103.86054550 a.u.
ν -179.15 cm^{-1}

(L-Prolinate)copper(I) iodide

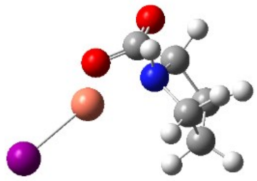

Geometry-optimised cartesian coordinates
Charge = -1 Multiplicity = 1

**ATOM**	**X**	**Y**	**Z**
Cu	0.10915100	-0.27668200	0.23153900
O	-1.27788700	-1.78242600	-0.18519500
C	-2.49571000	-1.42685400	-0.01699800
C	-2.76868800	0.04191600	0.37294200
N	-1.58410000	0.71208100	0.98242200
O	-3.49841200	-2.15885500	-0.18412700
C	-1.67120900	2.14755600	0.61041900
C	-2.07422600	2.06415700	-0.86106900
C	-3.10888400	0.91652400	-0.87819900
H	-3.61246100	0.04626900	1.07128100
H	-0.70925800	2.63662400	0.78066300
H	-2.44454300	2.66357800	1.19824500
H	-1.19484200	1.79399400	-1.45747200
H	-2.47501700	3.00590600	-1.24484600
H	-3.06117000	0.33577000	-1.80317400
H	-4.12637600	1.30616800	-0.78804100
H	-1.59614000	0.59716900	1.99416700
I	2.60501500	-0.00520400	-0.10849800

E(RB3LYP) -2052.64060244 a.u.

3.5.2.8. Ligand: (2*S*,4*R*)-*N*-(2,6-Dimethylphenyl)-4-hydroxypyrrolidine-2-carboxamide (DMPHPC)

((2*S*,4*R*)-*N*-(2,6-Dimethylphenyl)-4-hydroxypyrrolidine-2-carboxamide) *p*-tolyl(*S*-sulfinylphenyl)copper(III) iodide (**16g**)

See section 3.3.1.8 for the previously reported geometry-optimised structure of ((2*S*,4*R*)-*N*-(2,6-dimethylphenyl)-4-hydroxypyrrolidine-2-carboxamide) *p*-tolyl(*S*-sulfinylphenyl)copper(III) iodide (**16g**).

((2*S*,4*R*)-*N*-(2,6-Dimethylphenyl)-4-hydroxypyrrolidine-2-carboxamide) *p*-tolyl(*S*-sulfinylphenyl)copper(III) iodide reductive elimination transition state‡

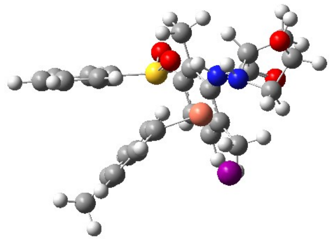

Geometry-optimised cartesian coordinates

Charge = 0 Multiplicity = 1

ATOM	X	Y	Z
C	-2.65488400	-0.24010100	-1.81505800
N	-2.02199500	-1.33279600	-0.98317800
Cu	-0.39202000	-0.97325600	0.22019000
N	-1.51534900	1.71062400	-0.92813300
C	-2.71711200	1.07436700	-1.00455500
C	-1.27696400	2.98437300	-0.32621400
O	-3.74973900	1.48513600	-0.47530100
C	-1.47135900	3.17553200	1.05617700
C	-1.18229100	4.44051500	1.58951100
C	-0.69377500	5.47031400	0.78476700
C	-0.48237600	5.24963100	-0.57840400
C	-0.77091800	4.00745100	-1.15617700
C	-1.93177600	2.05650100	1.95155500
C	-0.54230600	3.76469700	-2.62986600
C	-3.18227500	-2.05618000	-0.38553200
C	-4.17337500	-2.15053100	-1.53859600
C	-4.05853900	-0.76482800	-2.20363900
O	-3.68773800	-3.20144800	-2.39095000
S	1.10967200	-0.80515600	-1.64718600
C	2.90397700	-0.86452200	-1.83160700
C	3.61808600	0.33556800	-1.84075900
C	5.00714000	0.27969900	-1.96400000
C	5.65536600	-0.95758900	-2.06525300
C	4.91847500	-2.14672700	-2.05289700
C	3.52672000	-2.10948100	-1.93172300
O	0.60883800	-2.10961400	-2.18163200
O	0.66663800	0.46186300	-2.31303100
H	-2.01145700	-0.07856000	-2.68216000
H	-1.32644700	4.60686300	2.65365200
H	-0.46923800	6.44005600	1.21978900
H	-0.09633600	6.04882700	-1.20525700
H	-2.98593200	1.81830500	1.78067500
H	-1.36861300	1.13795300	1.76048500
H	-1.79552300	2.31817400	3.00389400
H	-1.43923300	3.35787500	-3.11016800
H	-0.27047600	4.69290200	-3.13831200
H	0.26535200	3.04174700	-2.79733500
H	-2.86091400	-3.02417400	-0.00224100
H	-3.58114500	-1.46214100	0.43871800
H	-5.19128100	-2.37286500	-1.19962700
H	-4.82612600	-0.09449300	-1.81100100
H	-4.17910200	-0.82959200	-3.28770700
H	-4.21985600	-3.22553800	-3.19788200
H	3.10169700	1.28468900	-1.75372600
H	5.58108600	1.20080500	-1.97830500
H	6.73678900	-0.99370700	-2.15405900

H	5.42285100	-3.10417700	-2.13640300
H	2.93932700	-3.02041600	-1.91921700
H	-0.77964800	1.35455600	-1.53946400
H	-1.61024200	-1.98132000	-1.66038600
C	1.42574800	-0.32569600	0.62001800
C	2.34019400	-1.18904000	1.23380800
C	1.51528000	1.05689300	0.75332600
C	3.29981400	-0.64261900	2.07823900
H	2.28603400	-2.26008000	1.07516400
C	2.47816800	1.58450100	1.62338000
H	0.85887700	1.72418000	0.21050000
C	3.38311500	0.74998400	2.29039900
H	3.99868400	-1.30452400	2.58303100
H	2.52985200	2.66144100	1.75692500
I	-1.08363900	-1.91479700	2.59977300
C	4.44013400	1.31256200	3.20738700
H	4.27169500	0.98082300	4.23871600
H	5.43716300	0.96416700	2.91601700
H	4.44070100	2.40544100	3.19792300

**E(RB3LYP)**	-3469.39725318	a.u.
**ν**	-178.67	cm^{-1}

((2*S*,4*R*)-*N*-(2,6-Dimethylphenyl)-4-hydroxypyrrolidine-2-carboxamide)copper(I) iodide

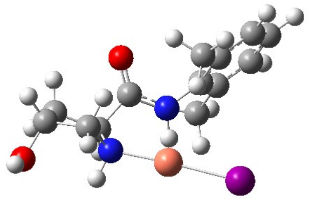

Geometry-optimised cartesian coordinates

Charge = 0   Multiplicity = 1

**ATOM**	**X**	**Y**	**Z**
Cu	0.95651600	1.22900700	-0.19052100
N	-0.42808100	-1.40901700	-0.54354600
C	0.67592500	-2.00423800	-0.01194400
C	2.00357600	-1.58379600	-0.68494500
N	2.34413500	-0.16105900	-0.30064600
O	0.63276400	-2.78619200	0.93724200
C	3.17930000	-0.31627300	0.93055600
C	4.11510700	-1.47666700	0.57596000
C	3.20410000	-2.43771700	-0.20852300
C	-1.77274600	-1.59969000	-0.09514200
C	-2.69912500	-2.12418000	-1.01938400
C	-4.02757300	-2.28853600	-0.60849500
C	-4.42077300	-1.94677300	0.68744200
C	-3.48802200	-1.42261100	1.58337700
C	-2.14930400	-1.22823300	1.21096700
C	-1.17054900	-0.62553100	2.18626100
C	-2.26589000	-2.49925000	-2.41699000
O	5.14025900	-1.02985500	-0.32743800
H	1.86880900	-1.61108700	-1.76881400
H	3.69462000	0.62012700	1.15202000
H	2.52366100	-0.57231500	1.76675100
H	4.55876100	-1.94271300	1.46201500
H	2.85391500	-3.24661100	0.43409000
H	3.75022100	-2.86779800	-1.05000600
H	-4.75290300	-2.69490500	-1.30812100
H	-5.45338800	-2.08278800	0.99607300
H	-3.79941700	-1.14351200	2.58637600
H	-1.69519600	-0.17704200	3.03368200
H	-0.47436900	-1.38075100	2.56409000
H	-0.57107800	0.15755300	1.70941400
H	-3.07517100	-3.00077000	-2.95321100
H	-1.98070800	-1.61587100	-3.00296700
H	-1.39753200	-3.16704400	-2.39957100
H	5.75723600	-0.46008300	0.15356400
H	-0.30704100	-0.90897600	-1.41654500
H	2.98796500	0.16553700	-1.02643400

I	-0.73449200	3.04014600	-0.13492900

E(RB3LYP) -2418.17234411 a.u.

3.5.3. Reductive elimination of biaryl sulfones from (bpy)Cu(Ar)(SO_2Ph)(I): variation of $\Delta E_{RE}^{‡}$ with aryl R group

3.5.3.1. Geometry optimisations of common species to all pathways

See section 3.5.1.1 for the previously reported geometry-optimised structure of (2,2'-bipyridyl)copper(I) iodide.

3.5.3.2. Aryl group: Phenyl (R = H)

(2,2'-Bipyridyl) phenyl(*S*-sulfinylphenyl)copper(III) iodide (**16h**)

See section 3.4.1.1 for the previously reported geometry-optimised structure of (2,2'-Bipyridyl) phenyl(*S*-sulfinylphenyl)copper(III) iodide (**16h**).

(2,2'-Bipyridyl) phenyl(*S*-sulfinylphenyl)copper(III) iodide reductive elimination transition state‡

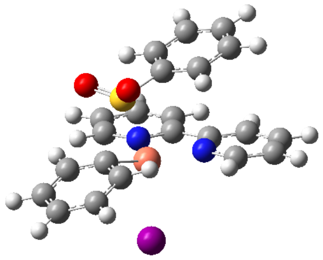

Geometry-optimised cartesian coordinates

Charge = 0 Multiplicity = 1

**ATOM**	**X**	**Y**	**Z**
C	-1.97814800	-1.40549600	-0.27006800
C	-1.06481800	-1.08124400	-2.38900900
C	-2.11172300	-1.75814900	-3.00768300
C	-3.12940600	-2.27377700	-2.20358700
C	-3.06228300	-2.09860200	-0.82270100
C	-1.81420100	-1.17858100	1.19253500
C	-0.49447200	-0.24584600	2.86166600
C	-1.37557300	-0.64711300	3.86449200
C	-2.52295300	-1.34769200	3.49016200
C	-2.74570400	-1.61836000	2.14066000
N	-0.70555300	-0.50270500	1.56508100
N	-1.00474200	-0.90418900	-1.06122200
Cu	0.58565700	-0.00466400	-0.11998300
S	0.06792300	2.43501600	0.01269800
C	-1.68748400	2.08070800	-0.30488000
C	-2.55533600	1.91566800	0.77471700
C	-2.12233100	1.99410400	-1.62953700
C	-3.90248400	1.65179100	0.51390400
C	-3.46883800	1.72237900	-1.87414300
C	-4.35598700	1.54649000	-0.80485500
O	0.44732300	3.37967300	-1.07566600
O	0.13418800	2.88150800	1.43207100
H	-0.24392900	-0.66146200	-2.96031500
H	-2.12363200	-1.87369700	-4.08545800
H	-3.96592500	-2.80710100	-2.64312600
H	-3.84584400	-2.49931500	-0.19259200
H	0.40753400	0.30790200	3.09712100
H	-1.16470100	-0.41257400	4.90178100
H	-3.23875200	-1.67776900	4.23595100
H	-3.63673900	-2.15509700	1.84112500
H	-2.18370700	1.99085000	1.78941300
H	-1.42254800	2.13049800	-2.44624300

H	-4.59076400	1.51883700	1.34263200
H	-3.82238400	1.64245400	-2.89719600
H	-5.40082100	1.32630400	-1.00119900
I	2.13629800	-2.28997600	-0.18726700
C	2.03779900	1.30753100	-0.05176100
C	2.67083600	1.43419400	-1.28986100
C	2.71994100	1.42847100	1.15679200
C	4.05975400	1.58438300	-1.31045400
H	2.09715300	1.40888100	-2.20964100
C	4.10879800	1.58903600	1.12092900
H	2.18453800	1.40277500	2.09807400
C	4.77512400	1.65811700	-0.10831500
H	4.57727500	1.65508900	-2.26253800
H	4.66307200	1.66581300	2.05158500
H	5.85246600	1.79208900	-0.13015000

E(RB3LYP) -3159.21857922 a.u.
ν -162.42 cm^{-1}

Sulfonyldibenzene

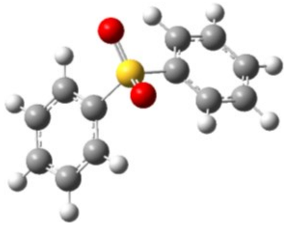

Geometry-optimised cartesian coordinates
Charge = 0 Multiplicity = 1

ATOM	X	Y	Z
C	3.04702800	-1.05452100	1.21474200
C	1.95761300	-0.18065600	1.22138200
C	1.42465200	0.23530300	-0.00156500
C	1.94728200	-0.19740400	-1.22324400
C	3.03665900	-1.07120000	-1.21387300
C	3.58315800	-1.49946400	0.00115200
S	0.00000300	1.33159300	-0.00317900
C	-1.42464000	0.23529800	0.00058300
O	0.00107100	2.07809500	1.27600400
O	-0.00109300	2.07154400	-1.28618200
C	-1.94886000	-0.18965900	1.22427400
C	-3.03827300	-1.06346800	1.21899400
C	-3.58316900	-1.49943500	0.00600400
C	-3.04541400	-1.06222300	-1.20968900
C	-1.95601700	-0.18840200	-1.22041900
H	3.47703000	-1.38316900	2.15552300
H	1.53592000	0.18027100	2.15295200
H	1.51780900	0.15058200	-2.15620200
H	3.45863700	-1.41282900	-2.15366300
H	4.43003100	-2.17868500	0.00220000
H	-1.52055500	0.16418000	2.15556100
H	-3.46151900	-1.39909600	2.16037500
H	-4.43005500	-2.17863700	0.00815400
H	-3.47415200	-1.39685900	-2.14893500
H	-1.53313000	0.16663100	-2.15371800

E(RB3LYP) -1011.94750713 a.u.

3.5.3.3. Aryl group: *p*-Tolyl (R = Me)

See section 3.5.1.3 for the previously reported geometry-optimised structures of (2,2′-bipyridyl) *p*-tolyl(*S*-sulfinylphenyl)copper(III) iodide (**16b**), (*p*-tolyl)sulfonylbenzene (**3**) and (2,2′-bipyridyl) *p*-tolyl(*S*-sulfinylphenyl)copper(III) iodide reductive elimination transition state[‡]. Additionally see section 3.5.1.1 for the previously reported geometry-optimised structure of (2,2′-bipyridyl)copper(I) iodide.

3.5.3.4. Aryl group: 4-Methoxyphenyl (R = OMe)

<u>(2,2’-Bipyridyl) 4-methoxyphenyl(*S*-sulfinylphenyl)copper(III) iodide (**16i**)</u>

See section 3.4.1.3 for the previously reported geometry-optimised structure of (2,2’-bipyridyl) 4-methoxyphenyl(*S*-sulfinylphenyl)copper(III) iodide (**16i**).

<u>(2,2’-Bipyridyl) 4-methoxyphenyl(*S*-sulfinylphenyl)copper(III) iodide reductive elimination transition state‡</u>

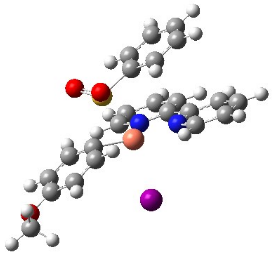

Geometry-optimised cartesian coordinates

Charge = 0 Multiplicity = 1

ATOM	X	Y	Z
C	-2.51950600	-1.10980200	-0.39697000
C	-1.50168500	-0.81827800	-2.47320400
C	-2.63268500	-1.25820600	-3.15462700
C	-3.74926300	-1.63190300	-2.40547000
C	-3.69243800	-1.55992000	-1.01496100
C	-2.36025500	-1.01471700	1.08031900
C	-0.95570800	-0.42400600	2.83280000
C	-1.90847600	-0.76731000	3.79074000
C	-3.13737100	-1.25906700	3.34886100
C	-3.36617400	-1.38839100	1.97968400
N	-1.17394200	-0.53929700	1.51722200
N	-1.45095000	-0.73792700	-1.13559300
Cu	0.21820700	-0.12249800	-0.11938200
S	0.05354000	2.33491100	-0.05889000
C	-1.75961300	2.25307700	-0.22838500
C	-2.54745400	2.22338100	0.92275600
C	-2.31629400	2.23306200	-1.50914600
C	-3.93643700	2.16746000	0.78206400
C	-3.70500800	2.17332000	-1.63448000
C	-4.51323800	2.13447400	-0.49177700
O	0.50452400	3.04669200	-1.28896900
O	0.26724300	2.95862200	1.27817600
H	-0.60400700	-0.51429900	-3.00057900
H	-2.63257300	-1.30177000	-4.23774700
H	-4.65478600	-1.97555200	-2.89463400
H	-4.55426500	-1.84911800	-0.42769800
H	0.01604200	-0.04052500	3.12414700
H	-1.68761100	-0.65109000	4.84579500
H	-3.90946600	-1.53955000	4.05785900
H	-4.31627400	-1.77067200	1.62877700
H	-2.08468600	2.24305600	1.90201700
H	-1.67608600	2.26108000	-2.38308400
H	-4.56266700	2.14049700	1.66821400
H	-4.15294400	2.15011300	-2.62285600
H	-5.59247900	2.07734500	-0.59536100
I	1.40780300	-2.62569200	-0.23302700
C	1.84304000	0.93410600	0.08122600
C	2.60842200	0.99655700	-1.08063400
C	2.43124600	0.95219300	1.34952400
C	4.00201700	0.96413500	-0.98663900
H	2.13003700	1.06529700	-2.05120700
C	3.81583500	0.92327200	1.44762600
H	1.81928900	0.99282900	2.24202700
C	4.60679000	0.91710200	0.28128300
H	4.59278600	0.98557800	-1.89383700
H	4.30651500	0.91352500	2.41553800
O	5.94888500	0.89068500	0.48785100

C	6.82535600	0.89931000	-0.64672700
H	6.69724700	1.81484500	-1.23484000
H	7.83503600	0.86527200	-0.23799300
H	6.65511200	0.02214100	-1.28081300

E(RB3LYP) -3273.75377021 a.u.
ν -147.12 cm^{-1}

(*p*-tolyl)sulfonylbenzene (**3**)

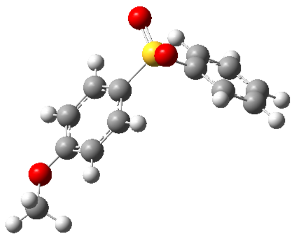

Geometry-optimised cartesian coordinates
Charge = 0 Multiplicity = 1

**ATOM**	**X**	**Y**	**Z**
C	2.61267700	-0.21098600	-0.96991600
C	1.34037400	0.34244700	-1.11810900
C	0.62912800	0.75778800	0.00827100
C	1.17059900	0.62422900	1.29466300
C	2.43459300	0.07308600	1.44503300
C	3.16214600	-0.34943900	0.31586700
S	-1.01406800	1.43043600	-0.18678700
C	-2.12500200	0.02103700	-0.05591400
C	-2.46810300	-0.68259600	-1.21340700
C	-3.30806300	-1.79269900	-1.09979700
C	-3.78849500	-2.18457600	0.15470300
C	-3.43554400	-1.46719900	1.30317000
C	-2.59674300	-0.35482900	1.20467100
O	-1.28193600	2.31854600	0.96909400
O	-1.12826100	1.96519200	-1.56408000
H	3.15851300	-0.52256600	-1.85115400
H	0.91152000	0.45837700	-2.10741100
H	0.61131200	0.95853500	2.16142900
H	2.88188300	-0.03660300	2.42710300
H	-2.09413600	-0.36153200	-2.17933300
H	-3.58863300	-2.34686500	-1.98983400
H	-3.81472300	-1.76934700	2.27421500
H	-2.32163400	0.21661600	2.08428900
H	-4.44115400	-3.04818000	0.23711200
O	4.38569700	-0.87504100	0.57229500
C	5.18919300	-1.33344600	-0.52464600
H	5.42302100	-0.51121800	-1.20943000
H	6.10823100	-1.70977000	-0.07667400
H	4.68632800	-2.14089700	-1.06772700

E(RB3LYP) -1126.48478419 a.u.

3.5.3.5. Aryl group: 4-Nitrophenyl (R = NO_2)

(2,2'-Bipyridyl) 4-nitrophenyl(*S*-sulfinylphenyl)copper(III) iodide (**16j**)

See section 3.4.1.4 for the previously reported geometry-optimised structure of (2,2'-bipyridyl) 4-nitrophenyl(*S*-sulfinylphenyl)copper(III) iodide (**16j**).

(2,2′-Bipyridyl) 4-nitrophenyl(*S*-sulfinylphenyl)copper(III) iodide reductive elimination transition state‡

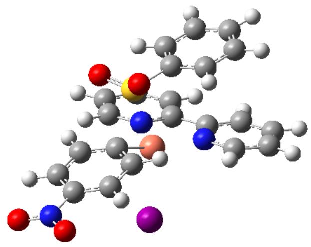

Geometry-optimised cartesian coordinates

Charge = 0 Multiplicity = 1

ATOM	X	Y	Z
C	2.72400900	1.10453600	-0.27593600
C	1.74569800	0.94787400	-2.38558000
C	2.89007500	1.42749600	-3.01569300
C	3.99045400	1.75588700	-2.22222400
C	3.90787200	1.59521000	-0.84025400
C	2.53592700	0.90750700	1.18807600
C	1.08388000	0.23101700	2.87182800
C	2.03378500	0.46363700	3.86496700
C	3.28687300	0.94033300	3.47822700
C	3.54188000	1.16670300	2.12618800
N	1.32560400	0.44458300	1.57251900
N	1.66861200	0.78585200	-1.05667100
Cu	-0.04963100	0.21145100	-0.09368500
S	-0.00654700	-2.35208600	0.10358100
C	1.75330800	-2.29854400	-0.31838200
C	2.69592200	-2.25485400	0.70978500
C	2.11640500	-2.31429000	-1.66736000
C	4.04985900	-2.22334500	0.36797400
C	3.47273600	-2.27355200	-1.99098600
C	4.43645900	-2.22289000	-0.97618200
O	-0.61472900	-3.25820600	-0.90594200
O	-0.08643400	-2.69008200	1.54811800
H	0.85661700	0.68083400	-2.94643800
H	2.91193600	1.53856000	-4.09374900
H	4.90325800	2.13370500	-2.67101100
H	4.75611200	1.85165200	-0.21869800
H	0.09858300	-0.14788900	3.11773800
H	1.79343100	0.27211000	4.90461600
H	4.05871800	1.13216400	4.21639400
H	4.51328500	1.53060800	1.81681100
H	2.37650000	-2.24501000	1.74469000
H	1.35880100	-2.35221500	-2.44184100
H	4.79779900	-2.18800600	1.15361100
H	3.77500300	-2.27561200	-3.03325000
H	5.48996200	-2.18211000	-1.23491400
I	-1.24159700	2.64949700	-0.19389200
C	-1.68817300	-0.85667500	0.00079500
C	-2.34464300	-0.90173100	-1.23724600
C	-2.39012200	-0.86278500	1.20934000
C	-3.73213600	-0.82905900	-1.26841500
H	-1.78015200	-0.97826600	-2.15929500
C	-3.78036800	-0.80027600	1.18501200
H	-1.86093100	-0.91492300	2.15243900
C	-4.42764400	-0.77141300	-0.05449200
H	-4.26982200	-0.82485300	-2.20807600
H	-4.35338400	-0.77556800	2.10325700
N	-5.88979400	-0.70564000	-0.08352900
O	-6.45381100	-0.68257200	-1.18361600
O	-6.49756500	-0.67357400	0.99277300

E(RB3LYP) -3363.73072146 a.u.

ν -161.05 cm^{-1}

1-Nitro-4-(phenylsulfonyl)benzene

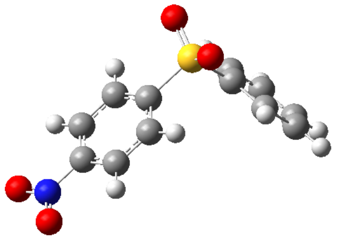

Geometry-optimised cartesian coordinates

Charge = 0 Multiplicity = 1

ATOM	X	Y	Z
C	-2.31202800	0.03151800	1.22525400
C	-1.01900800	0.54970900	1.22288000
C	-0.39126600	0.79807300	0.00018900
C	-1.01872700	0.54915400	-1.22259500
C	-2.31172300	0.03103700	-1.22504700
C	-2.93183900	-0.21910100	0.00006600
S	1.29377400	1.45377900	0.00023200
C	2.34224700	-0.00225900	0.00003900
C	2.72731300	-0.55802300	1.22344500
C	3.52355400	-1.70494700	1.21463100
C	3.91846600	-2.27625300	-0.00025600
C	3.52476600	-1.70379200	-1.21498400
C	2.72851000	-0.55686800	-1.22350400
O	1.48628900	2.16492400	-1.28178500
O	1.48634600	2.16460200	1.28241300
H	-2.83090300	-0.17003100	2.15336700
H	-0.51273300	0.76689900	2.15608000
H	-0.51221900	0.76586600	-2.15577200
H	-2.83048200	-0.17092600	-2.15316000
H	2.42085700	-0.09755600	2.15609600
H	3.83706000	-2.14772200	2.15444000
H	3.83921300	-2.14567500	-2.15489900
H	2.42289000	-0.09551600	-2.15599100
H	4.53734800	-3.16807700	-0.00037000
N	-4.30036600	-0.76586300	-0.00021500
O	-4.83948600	-0.98029500	-1.08849700
O	-4.83945500	-0.98250200	1.08753100

E(RB3LYP) -1216.45997536 a.u.

3.5.3.6. Aryl group: Phenyl (R = F)

(2,2’-Bipyridyl) 4-fluorophenyl(*S*-sulfinylphenyl)copper(III) iodide (**16k**)

See section 3.4.1.5 for the previously reported geometry-optimised structure of (2,2’-bipyridyl) 4-fluorophenyl(*S*-sulfinylphenyl)copper(III) iodide (**16k**).

(2,2’-Bipyridyl) 4-fluorophenyl(*S*-sulfinylphenyl)copper(III) iodide reductive elimination transition state‡

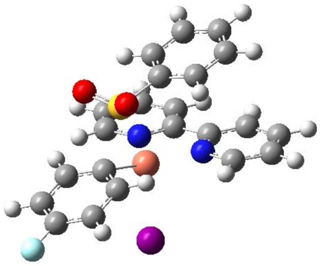

Geometry-optimised cartesian coordinates

Charge = 0 Multiplicity = 1

ATOM	X	Y	Z
C	-2.27778300	-1.22722100	-0.33905000
C	-1.32191800	-0.89663800	-2.43849200

C	-2.43379800	-1.42066600	-3.09140400
C	-3.50822600	-1.85781900	-2.31527300
C	-3.43016900	-1.76274100	-0.92707800
C	-2.09565000	-1.09977200	1.13348200
C	-0.69583700	-0.39026100	2.84752300
C	-1.60879100	-0.77029200	3.82982000
C	-2.81396800	-1.34323400	3.42128300
C	-3.06083600	-1.51293500	2.05951500
N	-0.93123600	-0.54795700	1.53912400
N	-1.25071200	-0.79593700	-1.10333300
Cu	0.40646800	-0.09208800	-0.11246700
S	0.11792200	2.37977200	0.06795200
C	-1.66786800	2.19598500	-0.22050100
C	-2.52458100	2.07877200	0.87427800
C	-2.13528900	2.19046900	-1.53694200
C	-3.89555200	1.95049800	0.63731700
C	-3.50614100	2.05363900	-1.75735600
C	-4.38370500	1.92918800	-0.67310100
O	0.57033200	3.28465500	-1.02497500
O	0.24749500	2.80887500	1.48767200
H	-0.45685700	-0.53973400	-2.98704500
H	-2.45212700	-1.47887800	-4.17369500
H	-4.39763600	-2.26826000	-2.78203600
H	-4.25942900	-2.10038900	-0.31901500
H	0.25440200	0.06186400	3.10995600
H	-1.37686600	-0.61871400	4.87796700
H	-3.55494400	-1.65408000	4.15054400
H	-3.99402000	-1.95512900	1.73469900
H	-2.12708100	2.08671500	1.88189800
H	-1.44223900	2.28659000	-2.36489600
H	-4.57617500	1.85724000	1.47764800
H	-3.88656700	2.03850100	-2.77369700
H	-5.44840900	1.81411500	-0.85138500
I	1.75433100	-2.49265300	-0.24755900
C	1.96877900	1.07958100	-0.01029900
C	2.62675600	1.18319900	-1.23932400
C	2.65348200	1.10534500	1.20457500
C	4.02130600	1.21044300	-1.25752700
H	2.06438300	1.23437800	-2.16464600
C	4.04920500	1.14083100	1.19370300
H	2.11168000	1.10257400	2.14232700
C	4.69466100	1.18086400	-0.03876800
H	4.57718800	1.26321100	-2.18712000
H	4.62494800	1.14307700	2.11261300
F	6.05471200	1.22081400	-0.05328200

E(RB3LYP) -3258.45869740 a.u.
ν -154.44 cm^{-1}

1-Fluoro-4-(phenylsulfonyl)benzene

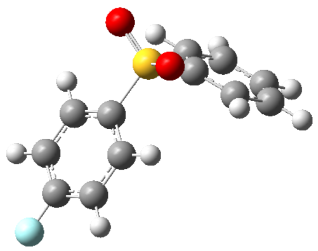

Geometry-optimised cartesian coordinates
Charge = 0 Multiplicity = 1

ATOM	X	Y	Z
C	3.18446500	-1.38093900	-1.21462200
C	2.23106200	-0.36053600	-1.22250400
C	1.76822400	0.13345200	0.00004600
C	2.23043300	-0.36158900	1.22241800
C	3.18382600	-1.38198900	1.21415500
C	3.65730600	-1.89093500	-0.00033300
S	0.51447400	1.42134500	0.00031300
C	-1.05294700	0.54433600	0.00008100

C	-1.63974000	0.20216100	-1.22176900
C	-2.84398700	-0.50130200	-1.22466800
C	-3.41271800	-0.83714700	-0.00024900
C	-2.84397400	-0.50189700	1.22433700
C	-1.63974000	0.20156700	1.22177900
O	0.61784600	2.15505400	1.28206000
O	0.61791700	2.15567600	-1.28106800
H	3.55880900	-1.77290800	-2.15487200
H	1.86159300	0.05151900	-2.15503400
H	1.86051400	0.04967400	2.15512000
H	3.55767300	-1.77477800	2.15426100
H	4.39847200	-2.68415700	-0.00048100
H	-1.17023600	0.48965700	-2.15561600
H	-3.33618300	-0.78120100	-2.14890100
H	-3.33618600	-0.78225800	2.14842200
H	-1.17022800	0.48863200	2.15575400
F	-4.58613000	-1.52251600	-0.00040300

E(RB3LYP) -1111.18804952 a.u.

4. References

1. *Maestro*, Schrödinger LLC, New York, NY, 2019-02.
2. M. J. Frisch, G. W. Trucks, H. B. Schlegel, G. E. Scuseria, M. A. Robb, J. R. Cheeseman, G. Scalmani, V. Barone, G. A. Petersson, H. Nakatsuji, X. Li, M. Caricato, A. V. Marenich, J. Bloino, B. G. Janesko, R. Gomperts, B. Mennucci, H. P. Hratchian, J. V. Ortiz, A. F. Izmaylov, J. L. Sonnenberg, Williams, F. Ding, F. Lipparini, F. Egidi, J. Goings, B. Peng, A. Petrone, T. Henderson, D. Ranasinghe, V. G. Zakrzewski, J. Gao, N. Rega, G. Zheng, W. Liang, M. Hada, M. Ehara, K. Toyota, R. Fukuda, J. Hasegawa, M. Ishida, T. Nakajima, Y. Honda, O. Kitao, H. Nakai, T. Vreven, K. Throssell, J. A. Montgomery Jr., J. E. Peralta, F. Ogliaro, M. J. Bearpark, J. J. Heyd, E. N. Brothers, K. N. Kudin, V. N. Staroverov, T. A. Keith, R. Kobayashi, J. Normand, K. Raghavachari, A. P. Rendell, J. C. Burant, S. S. Iyengar, J. Tomasi, M. Cossi, J. M. Millam, M. Klene, C. Adamo, R. Cammi, J. W. Ochterski, R. L. Martin, K. Morokuma, O. Farkas, J. B. Foresman and D. J. Fox, *Gaussian 16 Rev. C.01*, Wallingford, CT, 2016.
3. *MacroModel*, Schrödinger LLC, New York, NY, 2019-02.
4. A. D. Becke, *J. Chem. Phys.*, **1993**, *98*, 5648-5652.
5. C. Lee, W. Yang and R. G. Parr, *Phys. Rev. B: Condens. Matter Mater. Phys.*, **1988**, *37*, 785-789.
6. M. J. Frisch, J. A. Pople and J. S. Binkley, *J. Chem. Phys.*, **1984**, *80*, 3265-3269.
7. R. Ditchfield, W. J. Hehre and J. A. Pople, *J. Chem. Phys.*, **1971**, *54*, 724-728.
8. A. Bergner, M. Dolg, W. Kuechle, H. Stoll and H. Preuss, *Mol. Phys.*, **1993**, *80*, 1431-1441.
9. M. E. Foster and K. Sohlberg, *Phys. Chem. Chem. Phys.*, **2010**, *12*, 307-322.
10. S. Miertuš, E. Scrocco and J. Tomasi, *Chem. Phys.*, **1981**, *55*, 117-129.
11. J. P. Foster and F. Weinhold, *J. Am. Chem. Soc.*, **1980**, *102*, 7211-7218.
12. G. Vitzthum and E. Lindner, *Angew. Chem. Int. Ed. Engl.*, **1971**, *10*, 315-326.
13. C. Le, T. Q. Chen, T. Liang, P. Zhang and D. W. C. MacMillan, *Science*, **2018**, *360*, 1010-1014.
