# Supplementary material for: Experimental and computational insights into the mechanism of the copper(i)-catalysed sulfonylative Suzuki–Miyaura reaction
Source: Chem Sci. 2023 May 31;14(24):6738–55. doi: 10.1039/d3sc01337e (PMC10284122; doi:10.1039/d3sc01337e)
Supplement: SC-014-D3SC01337E-s002 [file SC-014-D3SC01337E-s002.pdf]

Electronic Supplementary Material (ESI) for Chemical Science.
This journal is © The Royal Society of Chemistry 2023

Experimental and computational insights into the mechanism of the copper(I)-catalysed sulfonylative Suzuki-Miyaura reaction

Callum G. J. Hall,[a,b] Helen F. Sneddon,*[a,†] Peter Pogány,[a] David M. Lindsay,[b] and William J. Kerr*[b]

[a] Medicines Design, GlaxoSmithKline, Gunnels Wood Road, Stevenage, SG1 2NY, England, UK

[b] Department of Pure and Applied Chemistry, University of Strathclyde, 295 Cathedral Street, Glasgow, G1 1XL, Scotland, UK

[†] Present address: Green Chemistry Centre of Excellence, Department of Chemistry, University of York, York, YO10 5DD, England, UK

Experimental Supporting Information

Contents 1

1. **General experimental methods** 3
2. **Preparation of reference compounds** 5
3. **Calculation of solution-based yields of major reaction components** 9
4. **Mechanistic investigations** 12

4.1. Identification of a sulfinate intermediate 12
4.1.1. Isolation of a sodium benzenesulfinate intermediate 12
4.1.2. Reaction of 4-iodotoluene with sodium benzenesulfinate 13
4.1.3. LCMS & HRMS identification of a benzenesulfinate intermediate 13
4.2. Identification and analysis of a resting-state copper(I) species 15
4.2.1. LCMS & HRMS identification of the resting-state copper(I) species 15
4.3. Transmetalation of arylboronic acid onto copper(I) 17
4.3.1. Reaction of 2,4,6-triphenylboroxine and 4-iodotoluene with 1,4-diazabicyclo[2.2.2]octane bis(sulfur dioxide) 17
4.3.2. Behaviour of phenylboronic acid in the presence of DABCO under reaction conditions 18
4.3.3. ^{11}B NMR reaction timecourse: identification of transmetalation intermediates and by-products 20
4.3.4. ^{19}F{^{1}H} NMR reaction timecourse: identification of transmetalation intermediates and by-products 21
4.3.5. Effect of counterion on the reactivity of the copper catalyst 22
4.3.6. Reaction spiking with inorganic fluorides 22
4.3.7. ^{19}F{^{1}H} NMR time course: transmetalation of 4-fluorophenylboronic acid onto copper(I), and identification of a fluorinated copper(I) aryl species 23
4.4. Insertion of SO_2(g) into the copper(I)–aryl bond 24
4.4.1. Effect of boronic acid R group on the migratory insertion process 24
4.4.2. ^{19}F{^{1}H} NMR time course: formation of 4-fluorobenzenesulfinate from insertion of SO_2(g) 27
4.4.3. ^{19}F{^{1}H} NMR study: interaction of 4-fluorobenzenesulfinate with copper(I) 28

4.5. Oxidative addition of copper(I) into the carbon-iodine bond 29
4.5.1. Effect of aryl halide on the yield of desired sulfone product: proof of a copper(I)/copper(III) two-electron catalytic cycle 29
4.5.2. $^{19}F\{^1H\}$ NMR timecourse: reaction of sodium 4-fluorobenzenesulfinate with iodobenzene 30
4.5.3. $^{19}F\{^1H\}$ NMR timecourse: reaction of sodium benzenesulfinate with 1-fluoro-4-iodobenzene 31
4.5.4. Effect of complex ligand on the oxidative addition of copper(I) into the carbon-iodine bond 32
4.5.5. Effect of aryl iodide on the oxidative addition of copper(I) into the carbon-iodine bond 33
4.6. Investigation of side-reactions in the sulfonylative Suzuki-Miyaura reaction 36
4.6.1. Investigation of benzenesulfinate disproportionation within the reaction mixture 36
4.6.2. Identification of starting material fates in the synthesis of an unsymmetrical sulfone39
4.6.3. Mechanistic control experiments: undesired symmetrical sulfone formation 41
5. References 52

1. General experimental methods

All procedures below were conducted under an inert nitrogen, N_2(g), atmosphere unless stated otherwise. Prior to commencing all reactions, reaction vessels were sealed, evacuated, and backfilled with N_2(g) (× 3) to ensure the presence of an inert atmosphere.

For reactions carried out in a CAT96 instrument, plates were prepared as described in the relevant section. Upon placing the plate into the instrument, it was sealed and a leak test was carried out, pressurising the system with N_2(g) (5 bar), and holding the pressure for 5 min (× 3). Upon completion, the system was heated to 110°C and pressurised with N_2(g) (5 bar). The reactions were left to stir under these conditions for 36 h. Upon completion and cooling, the system was automatically vented and purged with N_2(g) (5 bar) for 30 cycles, then opened. Plates were quenched and analysed as described in the relevant section.

Reactions were monitored by liquid chromatography-mass spectroscopy (LCMS). TLC was carried out using polyester-backed pre-coated silica plates (0.2 mm particle size). Spots were visualised under ultraviolet light of λ_{max} = 254 nm. In cases where spots were difficult to visualise, the plate was stained with $KMnO_4$ (potassium permanganate) before gentle heating.

During investigations into the copper(I)-catalysed sulfonylative Suzuki-Miyaura reaction, the visual deposition of metallic copper onto Teflon®-coated magnetic stirrer bars was observed throughout.

Column chromatography was carried out using the Teledyne ISCO Combi*Flash*® R*f+* apparatus with Redi*Sep*® silica cartridges (normal-phase), Biotage® SNAP KP-C18 cartridges (reverse-phase) or an EZ Prep® column (preparatory HPLC). Eluent conditions are stated in a form describing a gradient of the minor solvent (e.g. ethyl acetate) in the major solvent (e.g. cyclohexane).

Proton (^{1}H), carbon (^{13}C), boron (^{11}B), and fluorine (^{19}F) NMR spectra were measured on a Bruker AV400 (^{1}H = 400 MHz, ^{13}C = 101 MHz, ^{11}B = 128 MHz, ^{19}F = 376 MHz) spectrometer. For ^{1}H and ^{13}C spectra, chemical shifts (δ) are reported in ppm, relative to the chemical shift of tetramethylsilane (TMS = 0.00 ppm) or the following solvent peaks: chloroform-d$_1$; $CDCl_3$ (^{1}H = 7.26 ppm, ^{13}C = 77.2 ppm), methanol-d$_4$; CD_3OD (^{1}H = 3.31 ppm, ^{13}C = 49.0 ppm), or *N,N*-dimethylformamide-d$_7$; $(CD_3)_2NCDO$ (^{1}H = 8.02, 2.92, 2.75 ppm, ^{13}C = 162.7, 35.2, 30.1 ppm). Hetero-nuclear NMR spectra (^{11}B and ^{19}F) were referenced to the resonances of $BF_3{\cdot}OEt_2$, using the IUPAC universal reference scale with ^{1}H signal of TMS referenced to 0 ppm. Peak assignments are stated as chemical shifts, integrations, and coupling constants (where relevant). Coupling constants are quoted to the nearest 0.1 Hz and multiplicities described as either singlet (s), doublet (d), triplet (t), quartet (q), quintet (quin), sextet (sxt), septet (sept), broad (br), or multiplet (m). Crude ^{11}B and ^{19}F NMR spectra were processed, where appropriate, to remove broad borosilicate peaks or background noise.

HPLC analysis was completed on an Agilent 1260 Infinity instrument equipped with a Waters X-Select CSH C18 column (30 mm × 2.0 mm with 2.5 µm packing diameter). Analytes were detected at the wavelength specified by the user. Quantitative calculation of product yields was carried out using HPLC data and solution yield (K_x) methods (Section 3). All HPLC samples used for determination of quantitative yields were prepared using a Thermo Scientific™ E1-Cliptip™ electronic multichannel pipette to allow maximum accuracy. A single HPLC method was used:

- **General CSH Method:** 40 °C, 1 mL/min flow rate, using a mobile phase gradient of water containing 0.05% trifluoroacetic acid (v/v) and acetonitrile containing 0.05% trifluoroacetic acid

(v/v). Gradient conditions were initially 3% of the acetonitrile mixture, increasing linearly to 95% over 3.7 min, before remaining at 95% for 0.3 min.

LCMS analysis was completed on a Waters® Acquity UPLC instrument equipped with a BEH (ethylene-bridged hybrid) column (50 mm × 2.1 mm with 1.7 µm packing diameter) and a Waters® Micromass ZQ MS using alternate-scan positive and negative electrospray ionisation. Analytes were detected as a summed UV wavelength spectrum between 210-350 nm. High-resolution mass spectra were recorded on a micromass Q-Tof Ultima® time-of-flight mass spectrometer, and analytes were separated on an Agilent® 1100 instrument equipped with a Phenomenex® Luna C18 reverse-phase column (100 mm × 2.1 mm, 3 µm packing diameter). Mass to charge (m/z) ratios are shown in Daltons. Two LCMS methods were used:

- **Formic:** 40 °C, 1 mL/min flow rate, using a mobile phase gradient of water containing 0.1% formic acid (v/v) and acetonitrile containing 0.1% formic acid (v/v). Gradient conditions were initially 1% of the acetonitrile mixture, increasing linearly to 97% over 1.5 min, before remaining at 97% for 0.4 min, then rising to 100% over 0.1 min.
- **High pH:** 40 °C, 1 mL/min flow rate, using a mobile phase gradient of water containing aq. ammonium bicarbonate (10 mM, adjusted to pH 10 with 0.88 M aqueous ammonia) and acetonitrile. Gradient conditions were initially 1% of the acetonitrile mixture, increasing linearly to 97% over 1.5 min, before remaining at 97% for 0.4 min, then rising to 100% over 0.1 min.

Karl-Fisher titrations to determine H_2O content of solvent samples were carried out using a Mettler Toledo V10S volumetric KF instrument, using solutions of Hydranal Medium K solvent and Hydranal Composite 5K reagent.

2. Preparation of reference compounds

1,4-Diazabicyclo[2.2.2]octane bis(sulfur dioxide) adduct (DABSO)

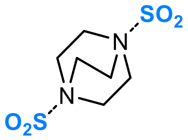

To a stirring solution of 1,4-diazabicyclo[2.2.2]octane (4.49 g, 40 mmol, 1.0 equiv.) in anhydrous tetrahydrofuran (72 mL) at 0 °C was added Karl Fisher Reagent solution A (pyridine-SO_2 solution in MeOH, 48 mL, ~112-150 mmol SO_2, ~2.80-3.75 equiv. SO_2) dropwise over a period of 5 min. Precipitation of the amine-SO_2 adduct as a white solid was observed shortly after addition. After stirring for 30 min at 0 °C, the flask was removed from the ice bath and allowed to warm to room temperature and stir for 3 h. Upon completion, the suspension was filtered under reduced pressure through a filter tube, and subsequently washed with diethyl ether. The solid was dried under high vacuum for a period of 72 h to yield the titled compound as a white solid (8.88 g, 92%).

^{1}H NMR (400 MHz, CD_3OD) δ_H ppm 3.21 (s, 12H); **^{13}C NMR** (151 MHz, CD_3OD) δ_C ppm 45.5; The analytical data were in accordance with the literature.[1]

Sulfonyldibenzene (20)

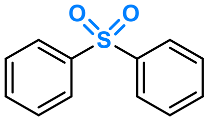

To a stirring solution of phenylboronic acid (366.0 mg, 3.0 mmol, 3.0 equiv.), DABSO (360.0 mg, 1.5 mmol, 1.5 equiv.), $Cu(MeCN)_4BF_4$ (31.5 mg, 0.1 mmol, 10 mol%), and 4,4'-dimethoxy-2,2'-bipyridine (21.6 mg, 0.1 mmol, 10 mol%) in DMPU (5 mL, 0.2 M) was added iodobenzene (112 µL, 1.0 mmol, 1.0 equiv.). The reaction was heated to 110 °C and left to stir for 36 h under N_2(g). Upon completion, the mixture was allowed to cool to room temperature, quenched with H_2O (5 mL), and was subsequently extracted with ethyl acetate (2×10 mL). The combined organic phases were washed with water (2×10 mL) and brine (2×10 mL), passed through a hydrophobic frit, and concentrated *in vacuo*. The crude product was purified by normal-phase flash column chromatography on a Redi*Sep*® pre-packed 12 g silica cartridge (cyclohexane, 0-10% ethyl acetate). UV active fractions were analysed by LCMS with product-containing fractions combined and concentrated *in vacuo* to give the titled compound as a white solid (143.7 mg, 66%).

^{1}H NMR (400 MHz, $CDCl_3$) δ_H ppm 7.94-7.99 (m, 4H), 7.58 (tt, J = 7.3, 1.5 Hz, 2H), 7.49-7.54 (m, 4H); **^{13}C NMR** (101 MHz, $CDCl_3$) δ_C ppm 141.1, 133.7, 129.7, 127.3; **LCMS** (Formic, low pH): $[M+H]^+$ not observed, t_{ret} = 0.99 min, 100% purity; The analytical data were in accordance with the literature.[2]

1-Methyl-4-(phenylsulfonyl)benzene (3)

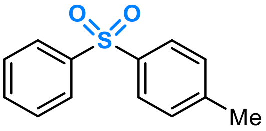

To a suspension of sodium 4-methylbenzenesulfinate (428.0 mg, 2.4 mmol, 1.2 equiv.), $(CuOTf)_2 \cdot C_6H_6$ (50.0 mg, 0.1 mmol, 5 mol%) and *N*,*N'*-dimethylethylenediamine (22 µL, 0.2 mmol, 10 mol%) in DMSO (5 mL) was added iodobenzene (224 µL, 2.0 mmol, 1.0 equiv.). The reaction was heated to 110 °C and left to stir for 24 h under N_2(g). Upon completion, the mixture was allowed to cool to room temperature, quenched with H_2O (10 mL), and was subsequently extracted with ethyl acetate (2×20 mL). The combined organic phases were washed with water (20 mL) and brine (20 mL), passed through a hydrophobic frit, and concentrated *in vacuo*. The crude product was purified by normal-phase flash column chromatography on a Redi*Sep*® pre-packed 24 g silica cartridge (cyclohexane, 0-15% ethyl acetate). UV active fractions were analysed by LCMS with product-containing fractions combined and concentrated *in vacuo* to give the titled compound as a white solid (229.7 mg, 49%).

^{1}H NMR (400 MHz, $CDCl_3$) δ_H ppm 7.92-7.96 (m, 2H), 7.82-7.86 (m, 2H), 7.56 (tt, *J* = 7.3, 1.0 Hz, 1H), 7.47-7.52 (m, 2H), 7.28-7.32 (m, 2H), 2.41 (s, 3H); **^{13}C NMR** (101 MHz, $CDCl_3$) δ_C ppm 144.1, 142.1, 138.7, 132.9, 129.9, 129.2, 127.7, 127.5, 21.5; **LCMS** (Formic, low pH): $[M+NH_4]^+$ observed at 250.12, t_{ret} = 1.07 min, 100% purity; The analytical data were in accordance with the literature.[2]

***S*-(*p*-Tolyl) 4-methylbenzenesulfonothioate**

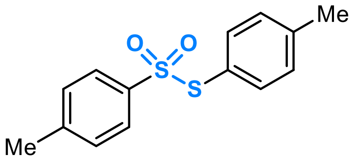

To a stirring solution of sodium *p*-toluenesulfinate (89.0 mg, 0.5 mmol, 1.0 equiv.) and CuI (95.0 mg, 0.5 mmol, 1.0 equiv.) in CH_2Cl_2 (3 mL) was added a solution of H_2SO_4 (200 µL, 0.5 mmol) in CH_2Cl_2 (3 mL) dropwise over 1 min. The reaction was stirred at room temperature for 30 min. Upon completion, the mixture was diluted with H_2O (10 mL), and was subsequently extracted with CH_2Cl_2 (2×10 mL). The combined organic phases were washed with water (10 mL) and brine (10 mL), passed through a hydrophobic frit, and concentrated *in vacuo*. The crude product was purified by normal-phase flash column chromatography on a Redi*Sep*® pre-packed 12 g silica cartridge (cyclohexane, 0-7.5% ethyl acetate). UV active fractions were analysed by LCMS with product-containing fractions combined and concentrated *in vacuo* to give the titled compound as a white solid (43.5 mg, 33%).

^{1}H NMR (400 MHz, $CDCl_3$) δ_H ppm 7.47-7.51 (m, 2H), 7.23-7.30 (m, 4H), 7.15-7.20 (m, 2H), 2.46 (s, 3H), 2.42 (s, 3H); **^{13}C NMR** (101 MHz, $CDCl_3$) δ_C ppm 144.5, 142.0, 140.5, 136.5, 130.2, 129.3, 127.6, 124.6, 21.6, 21.4; **LCMS** (Formic, low pH): $[M+NH_4]^+$ observed at 296.02, t_{ret} = 1.32 min, 85% purity; The analytical data were in accordance with the literature.[3]

S-(4-Fluorophenyl) 4-fluorobenzenethiosulfonate

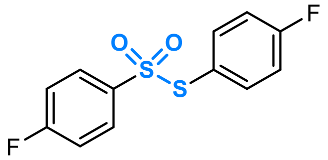

To a stirring solution of 4-fluorobenzenesulfonyl chloride (97.0 mg, 0.5 mmol, 1.0 equiv.) in a 5:1 mixture of acetonitrile:acetone (1.25:0.25 mL) was added dropwise a solution of tetrabutylammonium iodide (554 mg, 1.5 mmol, 3.0 equiv.) in a 5:1 mixture of acetonitrile:acetone (1.25:0.25 mL). The reaction was stirred at room temperature for 20 h. Upon completion, the solvent was removed *in vacuo* to yield a crude residue. The crude product was purified by normal-phase flash column chromatography on a Redi*Sep*® pre-packed 24 g silica cartridge (cyclohexane, 0-5% ethyl acetate). UV active fractions were analysed by LCMS with product-containing fractions combined and concentrated *in vacuo* to give the titled compound as a white solid (27.4 mg, 38%).

^{1}H NMR (400 MHz, $CDCl_3$) δ_H ppm 7.58-7.65 (m, 2H), 7.34-7.43 (m, 2H), 7.05-7.18 (m, 4H); **^{13}C NMR** (101 MHz, $CDCl_3$) δ_C ppm 166.0 (d, *J*=73.2 Hz), 163.8 (d, *J*=71.7 Hz), 138.9, 138.8, 130.4 (d, *J*=9.2 Hz), 123.2 (d, *J*=3.1 Hz), 116.8 (d, *J*=21.4 Hz), 116.2 (d, *J*=22.9 Hz); **^{19}F{^{1}H} NMR** (376 MHz, DMF-d_7) δ_F ppm -104.4, -108.8; **LCMS** (Formic, low pH): $[M+H]^+$ not observed, t_{ret} = 1.22 min, 80% purity – decomposition of thiosulfonate observed to an additional peak at t_{ret} = 1.47 min, although pure by NMR; The analytical data were in accordance with the literature.[4]

4-Methyl-1,1'-biphenyl

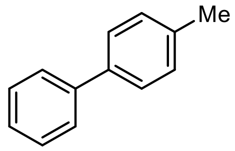

To a stirring solution of *p*-tolylboronic acid (204.0 mg, 1.5 mmol, 1.5 equiv.), palladium(II) acetate (1.1 mg, 5 µmol, 0.5 mol%), potassium carbonate (276 mg, 2 mmol, 2.0 equiv.) in ethanol (3 mL) and H_2O (3 mL) was added iodobenzene (112 µL, 1.0 mmol, 1.0 equiv.). The reaction was heated to 80 °C and left to stir for 12 h under N_2(g). Upon completion, the mixture was allowed to cool to room temperature, and was subsequently extracted with ethyl acetate (2×10 mL). The combined organic phases were washed with water (10 mL) and brine (10 mL), passed through a hydrophobic frit, and concentrated *in vacuo*. The crude product was purified by normal-phase flash column chromatography on a Redi*Sep*® pre-packed 24 g silica cartridge (100% cyclohexane). UV active fractions were analysed by LCMS with product-containing fractions combined and concentrated *in vacuo* to give the titled compound as a white solid (122.2 mg, 73%).

^{1}H NMR (400 MHz, $CDCl_3$) δ_H ppm 7.57-7.62 (m, 2H), 7.49-7.53 (m, 2H), 7.41-7.47 (m, 2H), 7.34 (tt, *J* = 7.8, 2.9 Hz, 1H), 7.24-7.29 (m, 2H), 2.41 (s, 3H); **^{13}C NMR** (101 MHz, $CDCl_3$) δ_C ppm 141.2, 138.4, 137.0, 129.5, 128.7, 127.0, 127.0, 126.8, 21.1; **LCMS** (Formic, low pH): $[M+H]^+$ not observed, t_{ret} = 1.38 min, 97% purity; The analytical data were in accordance with the literature.[5]

4-Fluoro-1,1'-biphenyl

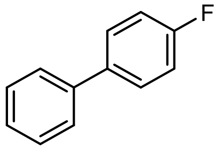

To a mixture of 4-fluorophenylboronic acid (168.0 mg, 1.2 mmol, 1.5 equiv.), palladium(II) acetate (0.9 mg, 4 µmol, 0.5 mol%), potassium carbonate (221.0 mg, 1.6 mmol, 2.0 equiv.) and iodobenzene (90 µL, 0.8 mmol, 1.0 equiv.) was added ethanol (2.5 mL) and H_2O (2.5 mL). The reaction was heated to 80 °C and left to stir for 12 h under N_2(g). Upon completion, the mixture was allowed to cool to room temperature, diluted with H_2O (5 mL), and was subsequently extracted with ethyl acetate (2×10 mL). The combined organic phases were washed with water (10 mL) and brine (10 mL), passed through a hydrophobic frit, and concentrated *in vacuo*. The crude product was purified by normal-phase flash column chromatography on a Redi*Sep*® pre-packed 12 g silica cartridge (100% cyclohexane). UV active fractions were analysed by LCMS with product-containing fractions combined and concentrated *in vacuo* to give the titled compound as a white solid (63.5 mg, 46%).

^{1}H NMR (400 MHz, $CDCl_3$) δ_H ppm 7.53-7.59 (m, 4H), 7.43-7.48 (m, 2H), 7.36 (tt, *J*=8.1, 3.0 Hz, 1H), 7.11-7.17 (m, 2H); **^{13}C NMR** (101 MHz, $CDCl_3$) δ_C ppm 163.7, 161.3, 140.3, 137.3 (d, *J*=3.1 Hz), 128.8, 128.7 (d, *J*=9.2 Hz), 127.1 (d, *J*=24.4 Hz), 115.4 (d, *J*=21.4 Hz); **^{19}F{^{1}H} NMR** (376 MHz, DMF-d_7) δ_F ppm -117.1; **LCMS** (Formic, low pH): $[M+H]^+$ not observed, t_{ret} = 1.38 min, 97% purity; The analytical data were in accordance with the literature.[5]

Tetrabutylammonium phenylfluoroboronate (*in situ* generation of reference compound)

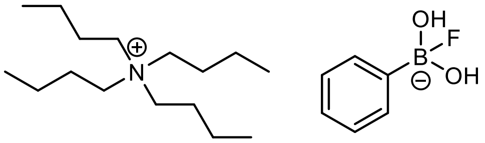

To a microwave vial containing phenylboronic acid (73.2 mg, 0.6 mmol, 1.0 equiv.) under N_2(g) was added a solution of tetrabutylammonium fluoride in THF (1M soln., 0.6 mL, 0.6 mmol, 1.0 equiv.). The reaction mixture was stirred at 20 °C for 2 h under N_2(g). Upon completion, a reaction aliquot was taken and diluted in a vial with $CDCl_3$ (~0.3 mL), after which the contents were transferred to an NMR tube. The sample was analysed using ^{11}B and ^{19}F{^{1}H} NMR to identify the resonances to be used as a reference in later NMR investigations.

^{11}B NMR (128 MHz, $CDCl_3$) δ_B ppm 4.3; **^{19}F{^{1}H} NMR** (376 MHz, $CDCl_3$) δ_F ppm -138.6.

3. Calculation of solution-based yields of major reaction components

As a robust method for calculation of HPLC yields for each reaction mixture, an internal standard was introduced into the reaction mixture. K_x values were calculated for sulfonyldibenzene **20** in 7 different samples, and in 4 duplicates for 1-methyl-4-(phenylsulfonyl)benzene **3**, iodobenzene **22,** 4-iodotoluene **2**, and *S*-phenyl benzenethiosulfonate **21**, with a mean value calculated, to ensure an accurate value used for yield calculation. Anomalous values were discarded in some cases, as highlighted in red in the tables to follow.

General procedure for calculation of K_x values for major reaction components

To a required number of volumetric flasks containing the internal standard dimethyl terephthalate (~25 mg, weighed accurately on a ±0.01 mg balance) and the desired reaction component (~25 mg, weighed accurately on a ±0.01 mg balance) was carefully added MeCN (25 mL) to the volume where the meniscus of solution lay on the graduation marking. The flask was sealed with an appropriate stopper, and vigorously shaken until all solids were solubilised. A 1 mL aliquot was taken from each flask using an electronic pipette, placed into individual HPLC vials, and analysed by HPLC (λ = 235 nm). The % areas of the internal standard and desired reaction component were then used to calculate individual K_x values for each vial by the following method, with symbols having the below definitions:

$$\boldsymbol{K_x} = \frac{\%_{IS}/\%_c}{mass_{IS}/mass_c}$$

- *$\%_c$ is the % area of the reaction component in the HPLC trace*
- *$\%_{IS}$ is the % area of the internal standard in the HPLC trace*
- *$mass_c$ is the mass of reaction component in the volumetric flask*
- *$mass_{IS}$ is the mass of reaction component in the volumetric flask*

This calculated K_x value is then used to calculate the mass of reaction component in the reaction HPLC sample:

$$\boldsymbol{Sample\ mass_c} = K_x * Sample\ mass_{IS} * {}^{\%_c}/_{\%_{IS}}$$

- *$Sample\ mass_c$ is the mass of the reaction component in the HPLC sample*
- *$Sample\ mass_{IS}$ is the mass of the reaction component in the HPLC sample*

Sulfonyldibenzene (20)

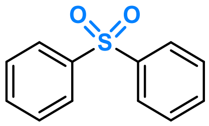

20

Entry	$mass_{IS}$ (mg)	$mass_{C}$ (mg)	Volume of MeCN (mL)	$\%_{IS}$	$\%_{C}$	K_x
1	25.49	24.05	25	55.511	44.489	1.1772
2	25.69	24.28	25	55.153	44.847	1.1623
3	24.9	24.95	25	53.313	46.987	1.1369
4	27.63	25.78	25	55.491	44.509	1.1632
5	24.11	24.78	25	53.367	46.633	1.1762
6	25.44	24.05	25	55.178	44.822	1.1637
7	26.93	25.63	25	55.528	44.472	1.1883
						**1.167**

1-Methyl-4-(phenylsulfonyl)benzene (3)

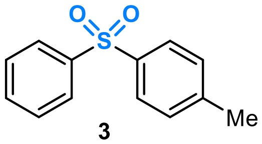

3

Entry	$mass_{IS}$ (mg)	$mass_{C}$ (mg)	Volume of MeCN (mL)	$\%_{IS}$	$\%_{C}$	K_x
1	24.51	26.01	25	51.500	48.250	1.1327
2	24.64	24.89	25	52.065	47.836	1.0995
3	26.64	24.26	25	53.057	46.860	1.0313
4	25.12	26.38	25	51.194	47.717	1.1266
						**1.112**

**red values indicate anomalous results, and were discarded when calculating a mean K_x value*

Iodobenzene (22)

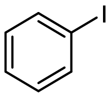

22

Entry	$mass_{IS}$ (mg)	$mass_{C}$ (mg)	Volume of MeCN (mL)	$\%_{IS}$	$\%_{C}$	K_x
1	25.93	25	25	77.478	22.522	3.3167
2	25.02	25	25	77.269	22.731	3.3966
3	29.03	25	25	79.242	20.758	3.2875
4	25.53	25	25	77.476	22.524	3.3683
						**3.342**

4-Iodotoluene (2)

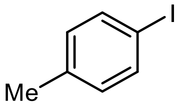

2

Entry	*mass*$_{IS}$ (mg)	*mass*$_{C}$ (mg)	Volume of MeCN (mL)	%$_{IS}$	%$_{C}$	K_x
1	25.69	25.91	25	60.537	39.095	1.5620
2	23.85	25.44	25	60.375	39.625	1.6251
3	26.33	26.21	25	60.741	38.858	1.5565
4	25.56	24.84	25	61.142	38.858	1.5289
						**1.162**

**red values indicate anomalous results, and were discarded when calculating a mean K_x value*

S-Phenyl benzenethiosulfonate (21)

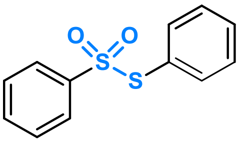

21

Entry	*mass*$_{IS}$ (mg)	*mass*$_{C}$ (mg)	Volume of MeCN (mL)	%$_{IS}$	%$_{C}$	K_x
1	24.70	25.65	25	52.818	46.653	1.1757
2	24.56	24.41	25	53.352	45.772	1.1585
3	25.16	25.11	25	54.055	45.294	1.1911
4	24.81	26.22	25	52.683	46.584	1.1953
						**1.180**

4. Mechanistic investigations

4.1. Identification of a sulfinate intermediate

4.1.1. Isolation of a sodium benzenesulfinate intermediate

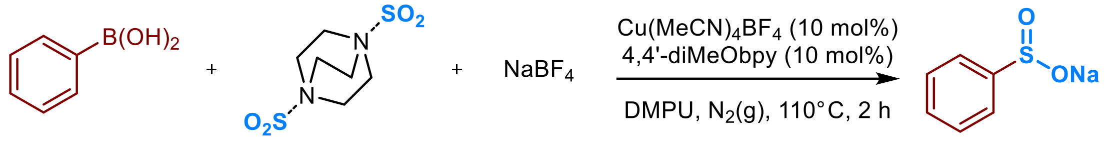

4 (3.0 equiv.) (1.5 equiv.) (15 equiv.) **1**

To a microwave vial containing phenylboronic acid (73.2 mg, 0.6 mmol, 3.0 equiv.), DABSO (72.1 mg, 0.3 mmol, 1.5 equiv.), $Cu(MeCN)_4BF_4$ (18.9 mg, 0.06 mmol, 10 mol%), 4,4'-dimethoxy-2,2'-bipyridine (13.0 mg, 0.06 mmol, 10 mol%) and sodium tetrafluoroborate (329 mg, 3.0 mmol, 5.0 equiv.) was added DMPU (3.0 mL). The reaction was heated to 110 °C and left to stir for 16 h under N_2(g). Upon completion, the mixture was left to cool to room temperature, after which it was diluted with water (10 mL) and CH_2Cl_2 (10 mL). The mixture was cooled to 0 °C and hydrochloric acid (37% aq. soln., 99 µL, 1.2 mmol) was added. The reaction was stirred at 0 °C for 5 min, after which the mixture was quickly transferred to a separating funnel, extracted, and the organic layer retained. This was quickly washed with sodium carbonate (3×10 mL, 10% w/w solution), and the aqueous layer was dried *in vacuo* to yield a blue-white solid. This solid can be assumed to be a mixture of inorganics (remaining sodium carbonate), copper species and the sulfinate species. A ^{1}H NMR spectrum of the residue, and a commercially available reference of sodium benzenesulfinate were recorded and compared.

Crude aqueous residue: **^{1}H NMR** (400 MHz, *DMF-d$_7$*) δ_H ppm 7.60-7.65 (m, 2H), 7.32-7.39 (m, 2H), 7.22-7.27 (m, 1H);

Reference commercial sodium benzenesulfinate: **^{1}H NMR** (400 MHz, *DMF-d$_7$*) δ_H ppm 7.61-7.66 (m, 2H), 7.31-7.36 (m, 2H), 7.28 (tt, *J*=7.1, 2.2 Hz, 1H).

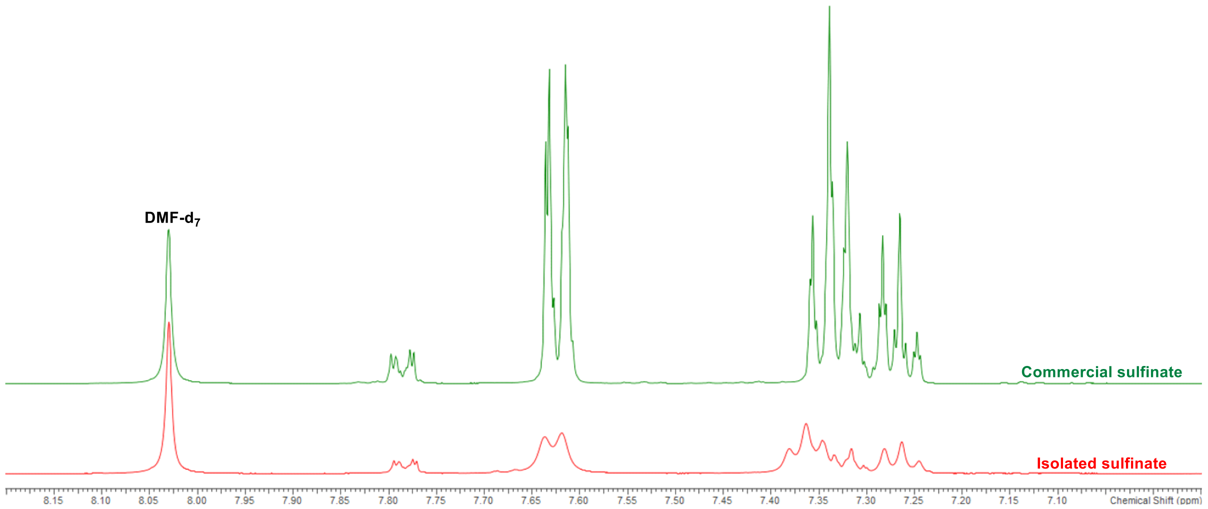

* discrepancies between samples in peak identification and intensity likely arise from the presence of quadrupolar Cu within the crude reaction mixture, leading to rapid relaxation of nuclei, and as a consequence, less defined multiplets.

4.1.2. Reaction of 4-iodotoluene with sodium benzenesulfinate

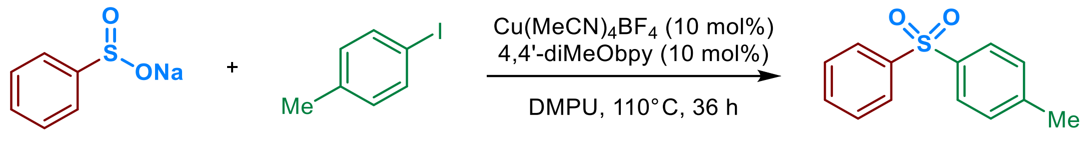

To a microwave vial containing sodium benzenesulfinate (49.2 mg, 0.3 mmol, 3.0 equiv.), $Cu(MeCN)_4BF_4$ (3.2 mg, 0.01 mmol, 10 mol%), and 4,4'-dimethoxy-2,2'-bipyridine (2.2 mg, 0.01 mmol, 10 mol%) under an N_2(g) atmosphere was added a solution of 4-iodotoluene (22 mg, 0.1 mmol, 1.0 equiv.) in DMPU (0.5 mL). The reaction was heated to 110 °C and left to stir for 36 h under N_2(g). Upon completion, the mixture was allowed to cool to room temperature, and then quenched with a stock solution of dimethyl terephthalate (1.5 mL, 0.0133 M solution in acetonitrile, 0.02 mmol). The resulting solution was sonicated, and a 50 µL aliquot was taken and diluted in a HPLC vial with acetonitrile (950 µL). Yields were determined by HPLC (λ = 235 nm) using % area and pre-determined K_x values for the major reaction components.

Entry	HPLC area$_{IS}$ (%)	HPLC area sulfone 3 (%)	HPLC yield sulfone 3 (%)
1	14.312	12.041	16

* Solution yields determined by HPLC (λ = 235 nm) using % area of internal standard, % area of reaction component, and pre-determined K_x values for the major reaction components (Section 3).

4.1.3. LCMS & HRMS identification of a benzenesulfinate intermediate

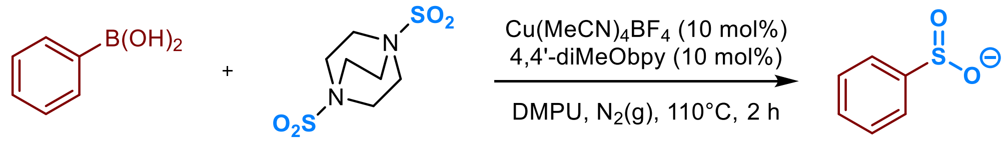

To a microwave vial containing phenylboronic acid (73.0 mg, 0.6 mmol, 3.0 equiv.), DABSO (72.0 mg, 0.3 mmol, 1.5 equiv.), $Cu(MeCN)_4BF_4$ (6.3 mg, 0.02 mmol, 10 mol%), and 4,4'-dimethoxy-2,2'-bipyridine (4.3 mg, 0.02 mmol, 10 mol%) was added DMPU (1.0 mL). The reaction was heated to 110 °C and left to stir for 2 h under N_2(g). After this time, an aliquot of the reaction mixture (~50 µL) was taken and diluted in an LCMS vial with acetonitrile (~950 µL). The reaction mixture was analysed by LCMS, with the peak in the UV trace eluting at t_{ret} = 0.61 min (formic, low pH), and corresponding mass ions of $[M]^-$ = 141.03 (ESI^-, expected 141.00) and $[M+2H]^+$ = 143.01 (ESI^+, expected 143.02).

UV Trace

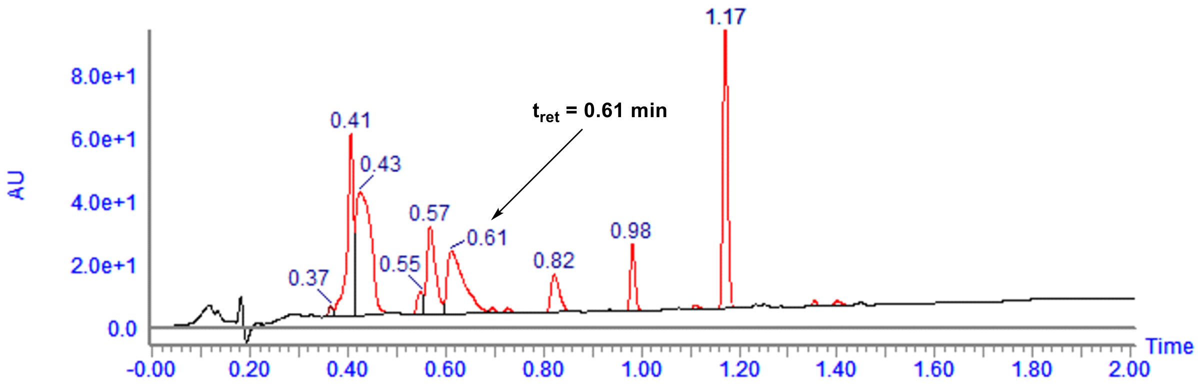

Positive ionisation (ESI$^+$)

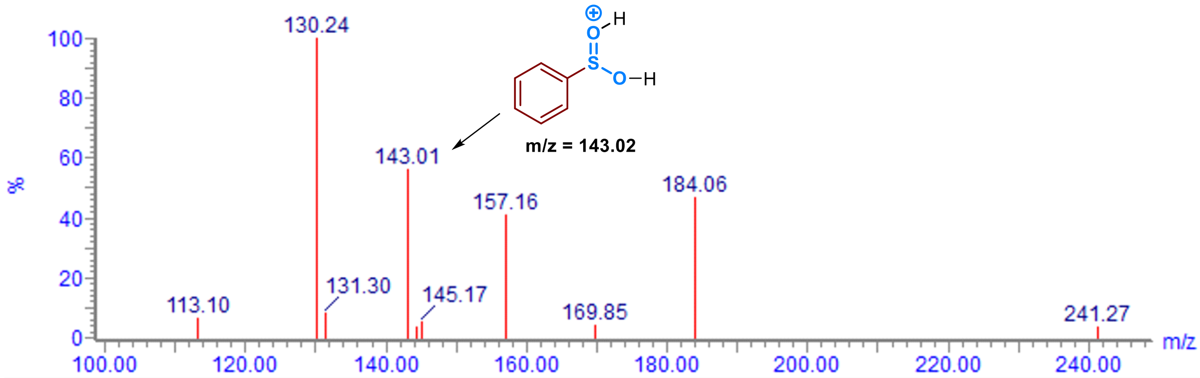

Negative ionisation (ESI$^-$)

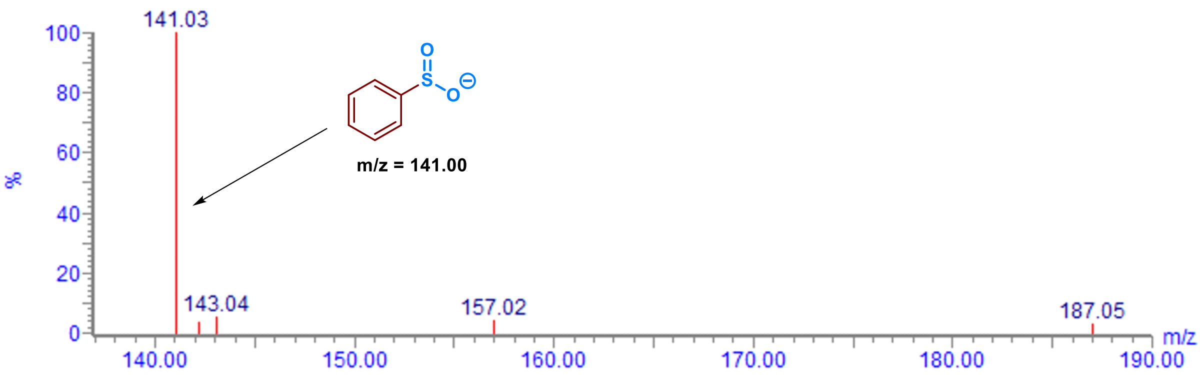

Commercial sodium benzenesulfinate reference

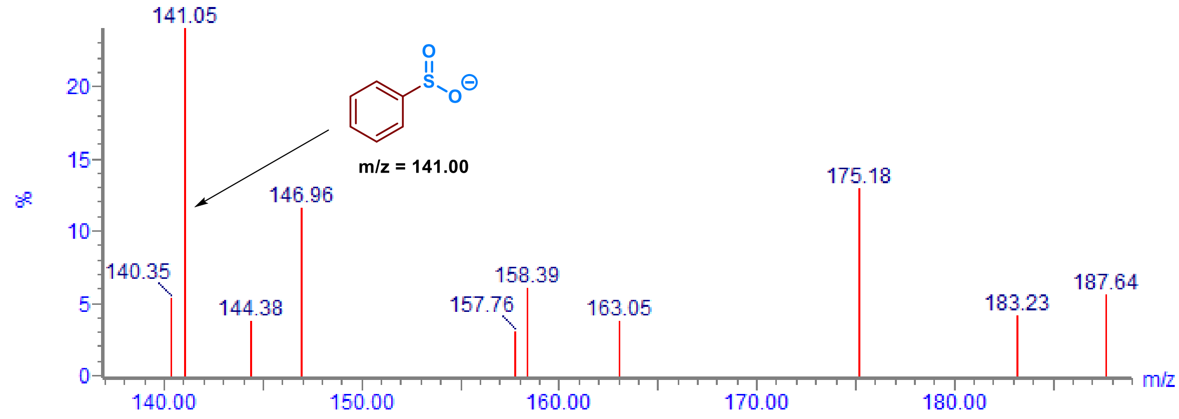

The same LCMS vial was then analysed using high-resolution mass spectrometry HRMS (formic, low pH), with the peak in the UV trace eluting at t_{ret} = 5.20 min, with a corresponding mass ion of $[M+2H]^+$ = 143.0176 (ESI$^+$, expected 143.0161).

UV trace

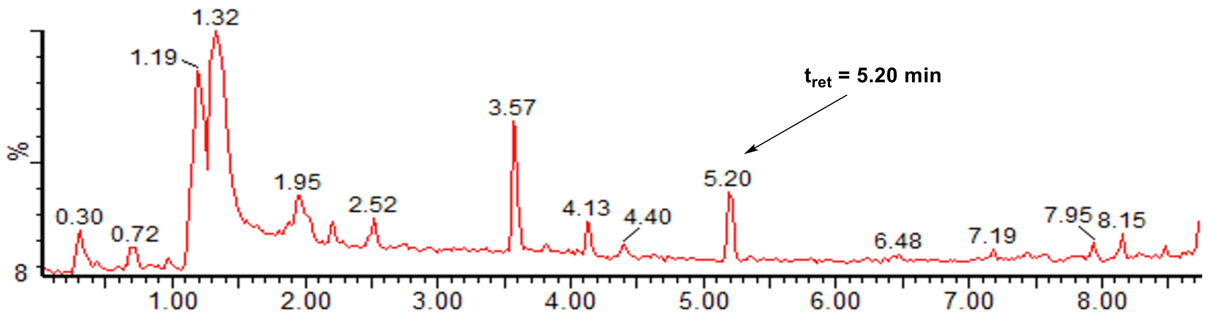

HRMS Positive ionisation (ESI$^+$)

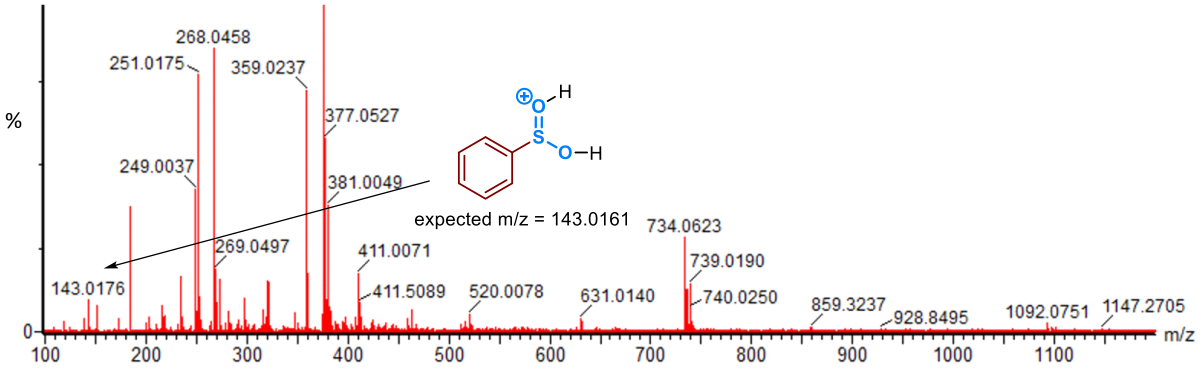

4.2. Identification and analysis of a resting-state copper(I) species

4.2.1. LCMS & HRMS identification of the resting-state copper(I) species

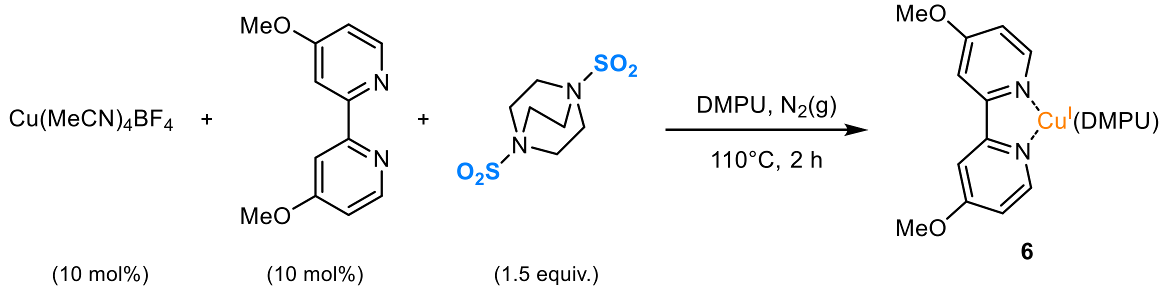

6

To a microwave vial containing DABSO (72.0 mg, 0.3 mmol, 1.5 equiv.), $Cu(MeCN)_4BF_4$ (6.3 mg, 0.02 mmol, 10 mol%), and 4,4'-dimethoxy-2,2'-bipyridine (4.3 mg, 0.02 mmol, 10 mol%) was added DMPU (1.0 mL). The reaction was heated to 110 °C and left to stir for 2 h under N_2(g). After this time, an aliquot of the reaction mixture (~50 µL) was taken and diluted in an LCMS vial with acetonitrile (~950 µL). The reaction mixture was analysed by LCMS, with the peak in the UV trace eluting at t_{ret} = 0.98 min (formic, low pH), and corresponding mass ions of $[LCu]^+$ = 279.10 (ESI+, expected 279.02), $[LCu(DABCO)]^+$ = 391.26 (ESI$^+$, expected 391.12), and $[LCu(DMPU)]^+$ = 407.15 (ESI$^+$, expected 407.11).

UV trace

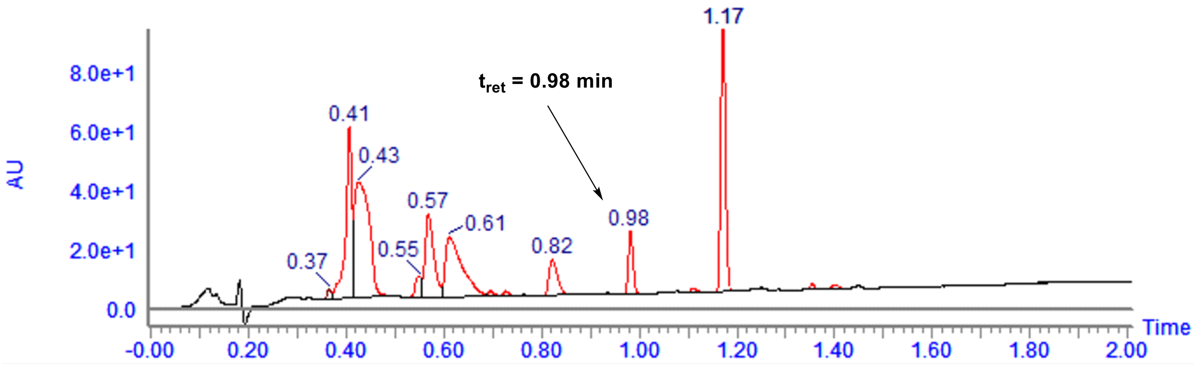

Positive ionisation (ESI$^+$)

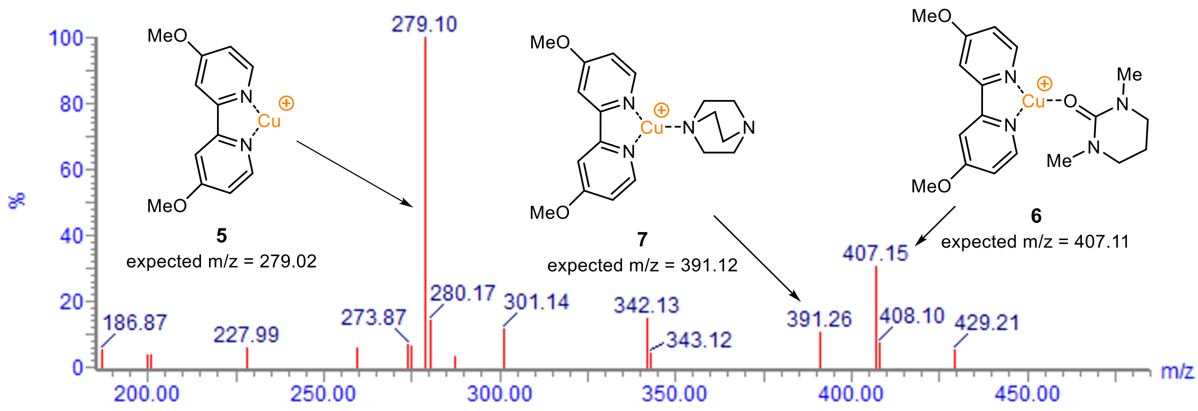

The same LCMS vial was then analysed using high-resolution mass spectrometry HRMS (formic, low pH), with the peak in the UV trace eluting at t_{ret} = 1.19 min, with a corresponding mass ion of $[M]^+$ = 279.0201 (ESI$^+$, expected 279.0189).

UV trace

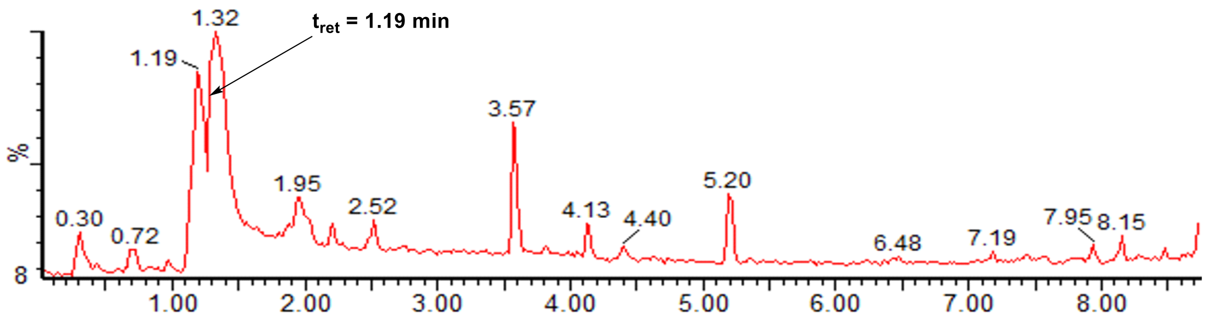

HRMS Positive ionisation (ESI$^+$)

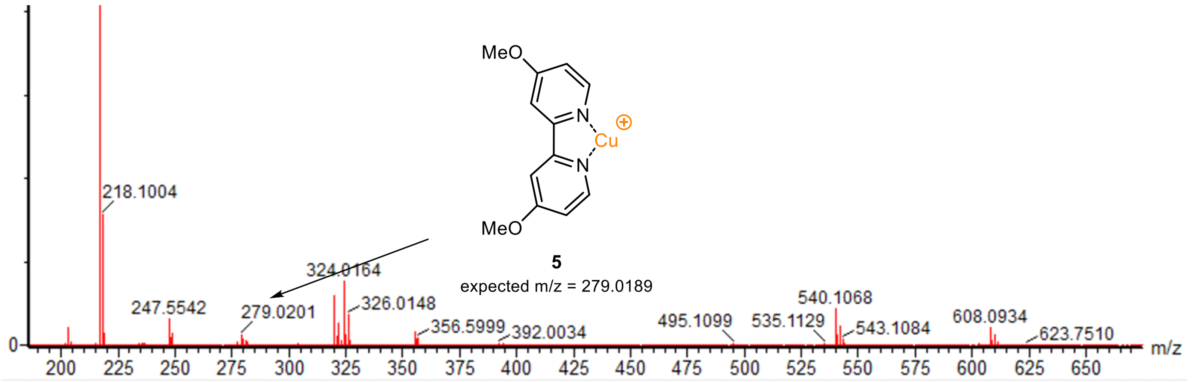

4.3. Transmetalation of arylboronic acid onto copper(I)

4.3.1. Reaction of 2,4,6-triphenylboroxine and 4-iodotoluene with 1,4-diazabicyclo[2.2.2]octane bis(sulfur dioxide)

In the presence of organic bases, such as 1,4-diazabicyclo[2.2.2]octane (DABCO), boronic acid species are known to dehydrate to form the corresponding boroxine species. In order to determine if this was occurring within the reaction mixture, and transmetalation occurring from the boroxine species instead of the boronic acid, a reaction was performed using 2,4,6-triphenylboroxine in place of phenylboronic acid.

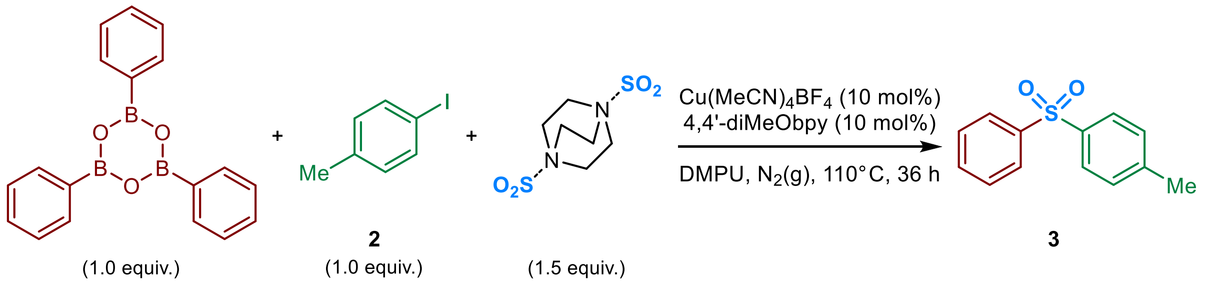

To a microwave vial containing 2,4,6-triphenylboroxine (31.2 mg, 0.1 mmol, 1.0 equiv.), $Cu(MeCN)_4BF_4$ (3.2 mg, 0.01 mmol, 10 mol%), 4,4’-dimethoxy-2,2’-bipyridine (2.2 mg, 0.01 mmol, 10 mol%) and DABSO (36.0 mg, 0.15 mmol, 1.5 equiv.) under N_2(g) was added a solution of 4-iodotoluene (21.8 mg, 0.1 mmol, 1.0 equiv.) in DMPU (0.5 mL). The reaction was heated to 110 °C and left to stir for 36 h under N_2(g). Upon completion, the mixture was allowed to cool to room temperature, and then quenched with a stock solution of dimethyl terephthalate (1.5 mL, 0.0133 M solution in acetonitrile, 0.02 mmol). The resulting solution was sonicated, and a 50 µL aliquot was taken and diluted in a HPLC vial with acetonitrile (950 µL). Yields were determined by HPLC

(λ = 235 nm) using % area and pre-determined K_x values for the major reaction components. A comparison reaction with phenylboronic acid is shown as Entry 1 in the table.

Entry	Boron species	HPLC area$_{IS}$ (%)	HPLC area sulfone 3 (%)	HPLC yield sulfone 3 (%)
1	Phenylboronic acid	10.444	22.517	**41**
2	2,4,6-Triphenylboroxine	13.385	20.624	**29**

* Solution yields determined by HPLC (λ = 235 nm) using % area of internal standard, % area of reaction component, and pre-determined K_x values for the major reaction components (Section 3).

Conclusions: Based on the diminished yields of the sulfone product when using the boroxine instead of the boronic acid, we can propose that the active transmetalation species is likely the boronic acid. Product is still observed using the boroxine, presumably due to the equilibrium nature of the two species in solution.

4.3.2. Behaviour of phenylboronic acid in the presence of DABCO under reaction conditions

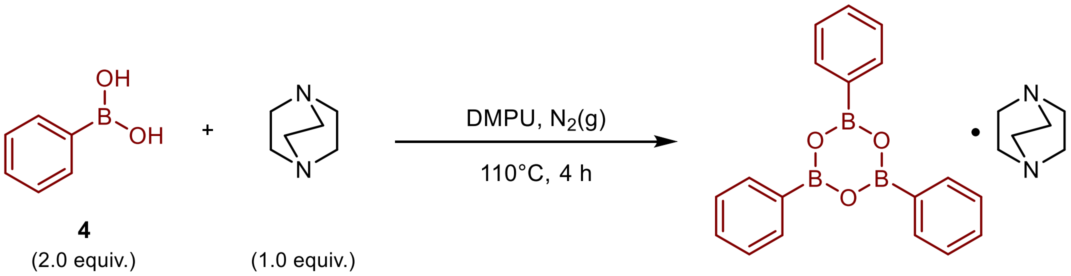

4
(2.0 equiv.)

(1.0 equiv.)

To a microwave vial containing phenylboronic acid (37.0 mg, 0.3 mmol, 2.0 equiv.) and 1,4-diazabicyclo[2.2.2]octane (16.8 mg, 0.15 mmol, 1.0 equiv.) was added DMPU (0.5 mL). The reaction was heated to 110 °C and left to stir for 4 h under N_2(g). After this time, a reaction aliquot was taken and diluted with $CDCl_3$ (~0.3 mL) and subsequently transferred to an NMR tube. The sample was analysed using ^{1}H and ^{13}C NMR, comparing to independent reference spectra for the phenylboronic acid and 1,4-diazabicyclo[2.2.2]octane starting materials. Comparison spectra are shown below.

^{1}H aliphatic

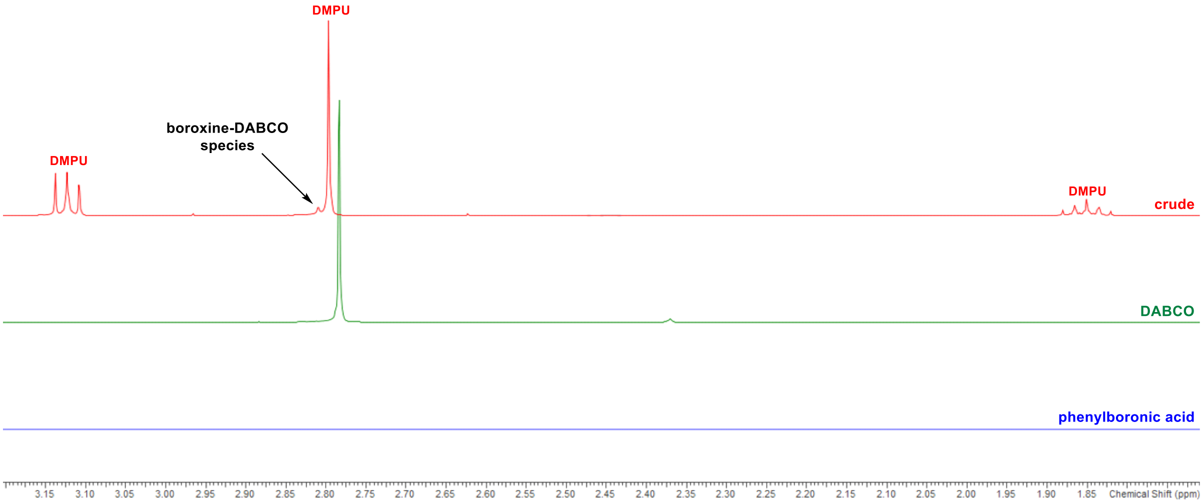

^{13}C aliphatic

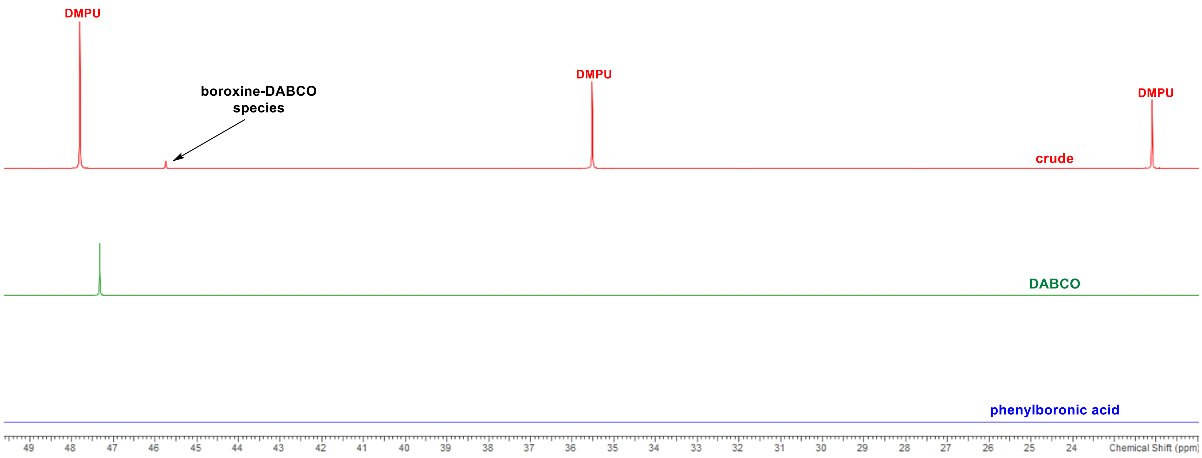

^{1}H aromatic

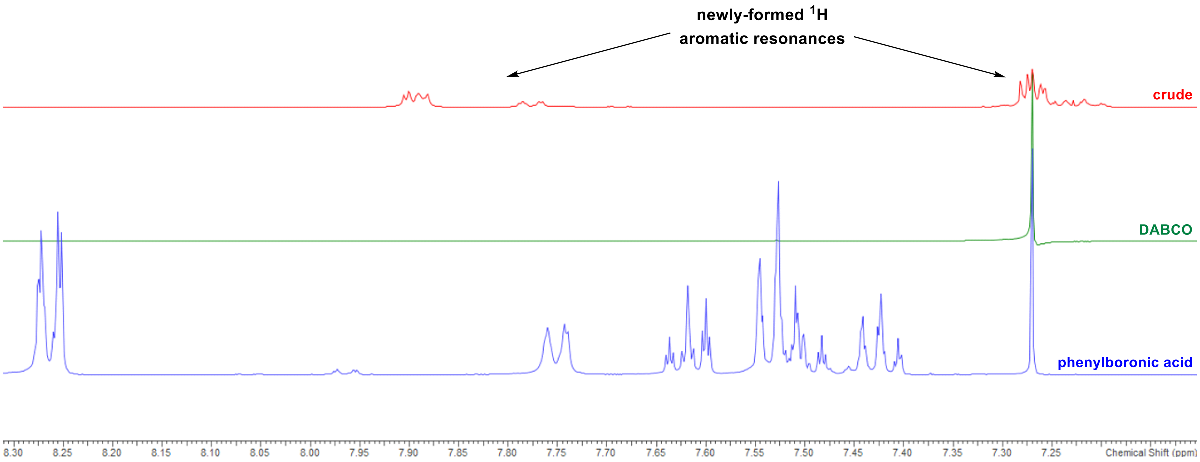

^{13}C aromatic

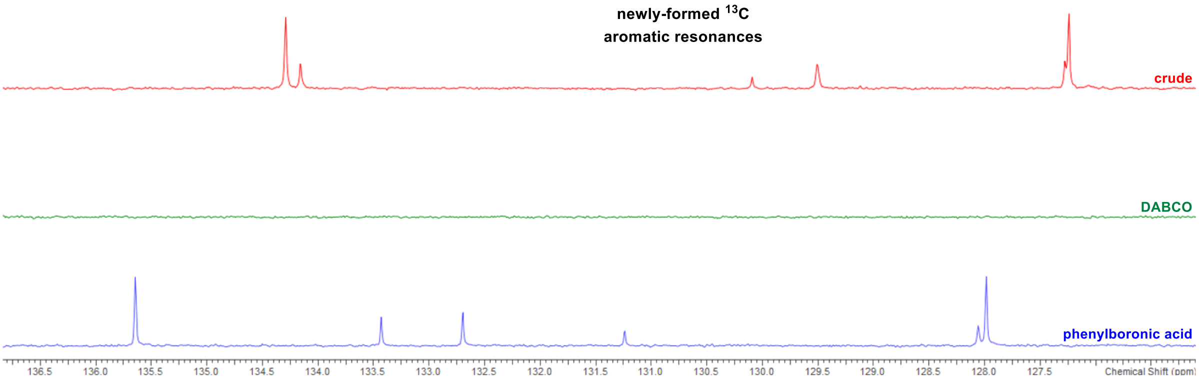

Conclusions: It appears that under reaction conditions, in the presence of DABCO, phenylboronic acid is converted into a boroxine-DABCO adduct. The presence of only a single ^{1}H and ^{13}C resonance for the boroxine-DABCO adduct suggests the system is fluctional in solution, in agreement with the

literature.[6] Slight shifts in these resonances compared with free amine suggest complexation to the Lewis-acidic boron atoms.

4.3.3. ^{11}B NMR reaction timecourse: identification of transmetalation intermediates and by-products

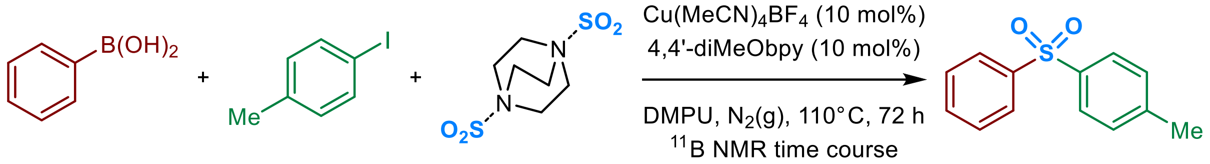

4 (3.0 equiv.) **2** (1.0 equiv.) (1.5 equiv.) **3**

To a microwave vial containing phenylboronic acid (73.2 mg, 0.6 mmol, 3.0 equiv.), DABSO (72.1 mg, 0.3 mmol, 1.5 equiv.), $Cu(MeCN)_4BF_4$ (6.3 mg, 0.02 mmol, 10 mol%), and 4,4'-dimethoxy-2,2'-bipyridine (4.3 mg, 0.02 mmol, 10 mol%) under N_2(g) was added a solution of 4-iodotoluene (43.6 mg, 0.2 mmol, 1.0 equiv.) in DMPU (1.0 mL). The reaction was heated to 110 °C and left to stir for 72 h under N_2(g). Reaction aliquots were taken at timepoints of t = 0, 4, and 72 h. The individual aliquots were diluted with $CDCl_3$ in an LCMS vial and transferred to an NMR tube, after which the sample was analysed by ^{11}B NMR (128 MHz, $CDCl_3$). The timepoints showed the following peaks:

Timepoint 1, t = 0 h: δ_B ppm +29.6 **[$PhB(OH)_2$]**, -1.1 **[BF_4^-]**;
Timepoint 2, t = 4 h: δ_B ppm +29.6 **[$PhB(OH)_2$]**, +2.1 **[$B(OH)_xF_y$ species]**, +0.7 **[BF_3-DABCO adduct]**;
Timepoint 3, t = 72 h: δ_B ppm +20.4 **[$B(OH)_3$]**, -1.1 **[BF_4^-]**;

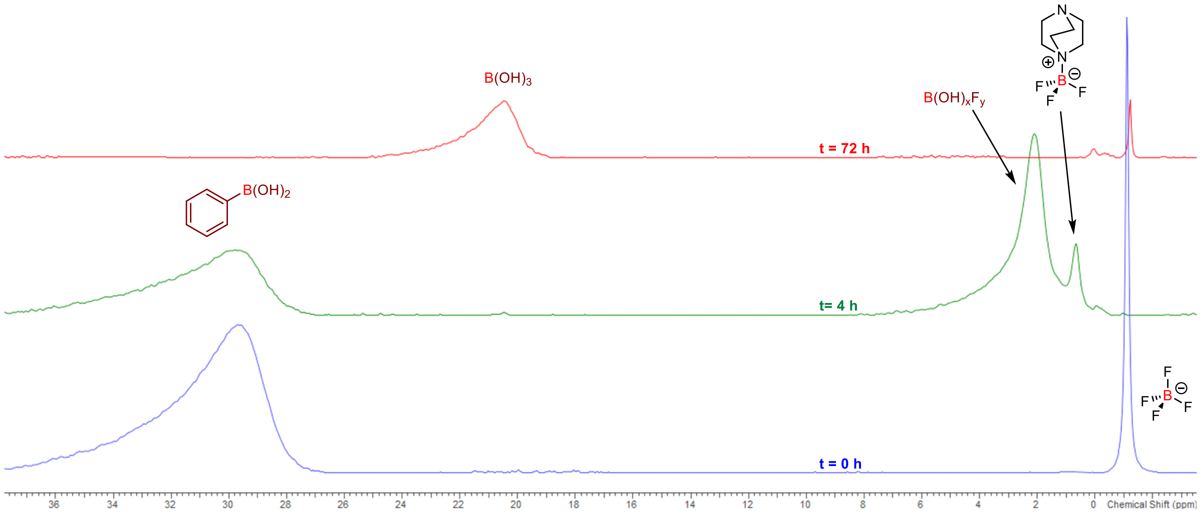

Peaks in the ^{11}B NMR spectrum were assigned using a combination of previous literature precedent and analysis of commercial references of the starting materials. Reference ^{11}B NMR spectra of the species phenylboronic acid and tetrakis(acetonitrile) copper(I) tetrafluoroborate showed chemical shifts of δ_B = 29.6 and δ_B = -0.9 ppm respectively. In relation to the slight shift in ^{11}B δ_B ppm of the tetrafluoroborate anion, this is known to occur upon changing of the coordination sphere of the anion.[7] The identification of the boric acid, $B(OH)_3$ (~20 ppm), and boron hydroxyfluoride species, $B(OH)_xF_y$ (~1-2 ppm), correlates well with the literature ^{11}B NMR shifts.[8] ^{11}B NMR chemical shifts of tertiary amine-BF_3 complexes such as F_3B·N-methylpiperidine are known to reside at approximate chemical shifts of 0-1 ppm, so the peak at δ_B = 0.66 ppm is in agreement for a DABCO-BF_3 adduct.[9] Attempts at independent preparation of this species using DABCO and $BF_3·OEt_2$ were unsuccessful.

4.3.4. $^{19}F\{^1H\}$ NMR reaction timecourse: identification of transmetalation intermediates and by-products

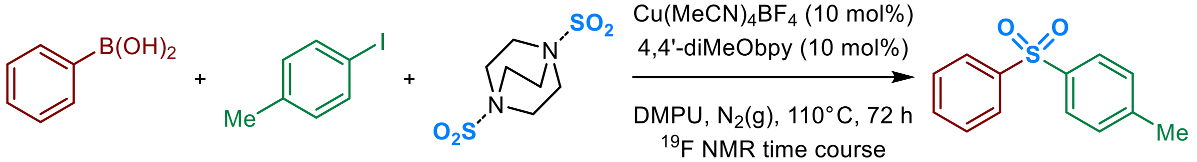

To a microwave vial containing phenylboronic acid (73.2 mg, 0.6 mmol, 3.0 equiv.), DABSO (72.1 mg, 0.3 mmol, 1.5 equiv.), $Cu(MeCN)_4BF_4$ (6.3 mg, 0.02 mmol, 10 mol%), and 4,4'-dimethoxy-2,2'-bipyridine (4.3 mg, 0.02 mmol, 10 mol%) under N_2(g) was added a solution of 4-iodotoluene (43.6 mg, 0.2 mmol, 1.0 equiv.) in DMPU (1.0 mL). The reaction was heated to 110 °C and left to stir for 72 h under N_2(g). Reaction aliquots were taken at timepoints of t=0, 4, and 72 h. The individual aliquots were diluted with $CDCl_3$ in an LCMS vial and transferred to an NMR tube, after which the sample was analysed by $^{19}F\{^1H\}$ NMR (376 MHz, $CDCl_3$). The timepoints showed the following peaks:

$^{19}F\{^1H\}$ NMR (376 MHz, *N,N*-dimethylformamide-d$_7$):

Timepoint 1, t = 0 h: δ_F ppm -152.8 **[BF_4^-]**;
Timepoint 2, t = 4 h: δ_F ppm -138.9 **[$PhB(OH)_2F^-$]**, -147.5 **[$B(OH)_xF_y$ species]**, -151.5 and -152.4 **varying [BF_4^-] coordination environments**, -161.6 **[BF_3-DABCO adduct];**
Timepoint 4, t = 72 h: δ_F ppm -153.8 **[BF_4^-]**, -161.6 **[BF_3-DABCO adduct]**;

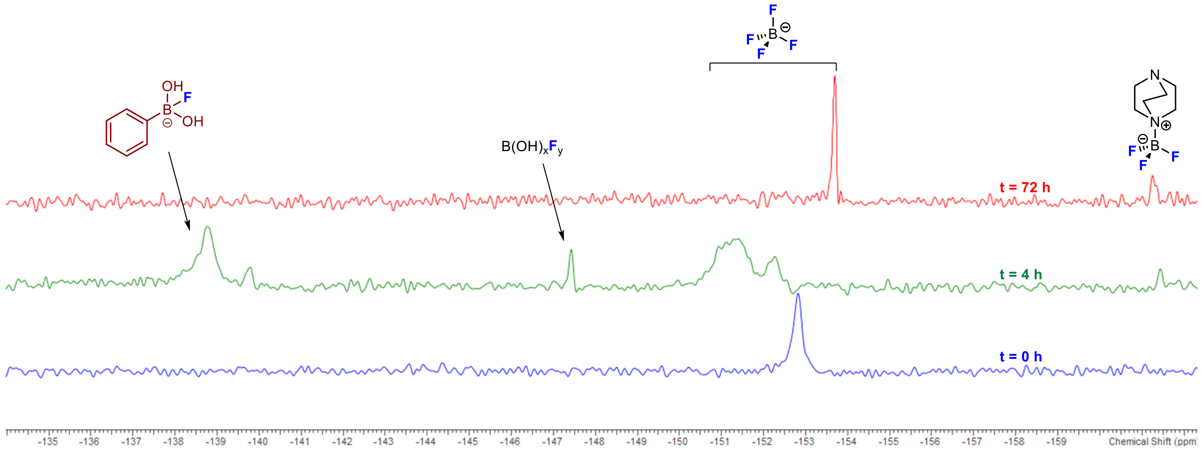

Peaks in the $^{19}F\{^1H\}$ NMR spectrum were assigned using a combination of previous literature precedent and analysis of commercial references of the starting materials. The reference $^{19}F\{^1H\}$ NMR spectrum of tetrakis(acetonitrile) copper(I) tetrafluoroborate showed chemical shifts of δ_F = -153.4 ppm. In relation to the slight shift in ^{19}F δ_F ppm of the tetrafluoroborate anion, this is known to occur upon changing of the coordination sphere of the anion.[7] The identification of a boron hydroxyfluoride species, $B(OH)_xF_y$ (~-147 ppm), correlates well with the literature ^{11}B NMR shifts.[8] ^{19}F NMR chemical shifts of tertiary amine-BF_3 complexes such as F_3B·N-methylpiperidine are known to reside at chemical shifts of ~-161 ppm, so the peak at δ_F = -161.4 ppm is in agreement for a DABCO-BF_3 adduct.[9] An independently prepared sample of tetrabutylammonium fluorodihydroxy(phenyl)borate from tetrabutylammonium fluoride and phenylboronic acid showed a crude reaction ^{19}F NMR shift of -

138.6 ppm, which is in agreement with both literature and the proposed intermediate in the reaction mechanism.[10]

4.3.5. Effect of counterion on the reactivity of the copper catalyst

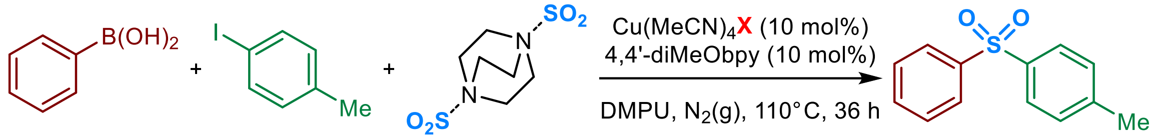

To a microwave vial containing phenylboronic acid (36.6 mg, 0.3 mmol, 3.0 equiv.), DABSO (36.0 mg, 0.15 mmol, 1.5 equiv.), tetrakis(acetonitrile) copper(I) salt (0.01 mmol, 10 mol%), and 4,4′-dimethoxy-2,2′-bipyridine (2.2 mg, 0.01 mmol, 10 mol%) under N_2(g) was added a solution of 4-iodotoluene (21.8 mg, 0.1 mmol, 1.0 equiv.) was added DMPU (0.5 mL). The reaction was heated to 110 °C and left to stir for 36 h under N_2(g). Upon completion, the mixture was allowed to cool to room temperature, and then quenched with a stock solution of dimethyl terephthalate (1.5 mL, 0.0133 M solution in acetonitrile, 0.02 mmol). The resulting solution was sonicated, and a 50 µL aliquot was taken and diluted in a HPLC vial with acetonitrile (950 µL). Yields were determined by HPLC (λ = 235 nm) using % area and pre-determined K_x values for the major reaction components.

Entry	Cu^I salt	Mass of Cu^I salt (mg)	HPLC area$_{IS}$ (%)	HPLC area sulfone 3 (%)	HPLC yield sulfone 3 (%)
1	$Cu(MeCN)_4$**BF_4**	3.1	10.444	22.517	**41**
2	$Cu(MeCN)_4$**OTf**	3.8	13.076	17.516	**25**
3	$Cu(MeCN)_4$**PF_6**	3.7	13.414	1.311	**2**

* Solution yields determined by HPLC (λ = 235 nm) using % area of internal standard, % area of reaction component, and pre-determined K_x values for the major reaction components (Section 3).

4.3.6. Reaction spiking with inorganic fluorides

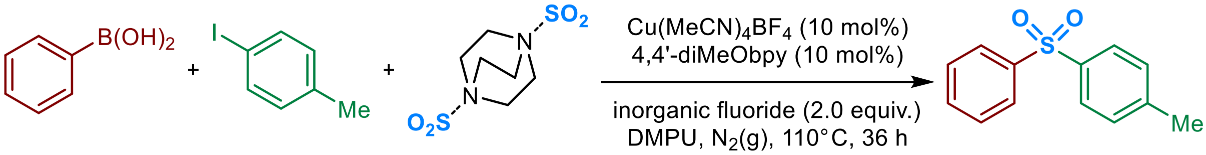

To a microwave vial containing phenylboronic acid (37.0 mg, 0.3 mmol, 3.0 equiv.), DABSO (36.0 mg, 0.15 mmol, 1.5 equiv.), $Cu(MeCN)_4BF_4$ (3.2 mg, 0.01 mmol, 10 mol%), 4,4′-dimethoxy-2,2′-bipyridine (2.2 mg, 0.01 mmol, 10 mol%) and inorganic fluoride (0.2 mmol, 2.0 equiv.) was added a solution of 4-iodotoluene (21.8 mg, 0.1 mmol, 1.0 equiv.) in DMPU (0.5 mL). The reaction was heated to 110 °C and left to stir for 36 h under N_2(g). Upon completion, the mixture was allowed to cool to room temperature, and then quenched with a stock solution of dimethyl terephthalate (1.5 mL, 0.0133 M solution in acetonitrile, 0.02 mmol). The resulting solution was sonicated, and a 50 µL aliquot was taken and diluted in a HPLC vial with acetonitrile (950 µL). Yields were determined by HPLC (λ = 235 nm) using % area and pre-determined K_x values for the major reaction components. A comparison reaction with no added inorganic fluoride salt is shown as Entry 1 in the table.

Entry	Inorganic fluoride (mass)	Mass of inorganic fluoride (mg)	HPLC area$_{IS}$ (%)	HPLC area sulfone 3 (%)	HPLC yield sulfone 3 (%)
1	–	–	10.444	22.517	**41**
2	KF	11.6	15.476	11.387	**14**
3	AgF	25.4	13.967	0.709	**1**

* Solution yields determined by HPLC (λ = 235 nm) using % area of internal standard, % area of reaction component, and pre-determined K_x values for the major reaction components.

4.3.7. ^{19}F{^{1}H} NMR time course: transmetalation of 4-fluorophenylboronic acid onto copper(I), and identification of a fluorinated copper(I) aryl species

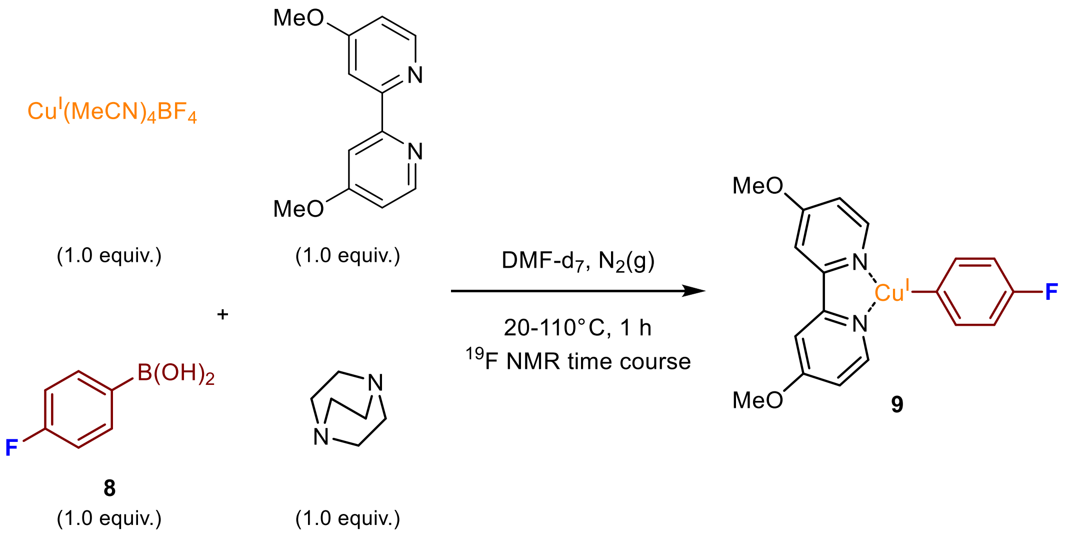

To a 4 mL vial was added 4-fluorophenylboronic acid (11.0 mg, 0.08 mmol, 1.0 equiv.), 1,4-diazabicyclo[2.2.2]octane (9.0 mg, 0.08 mmol, 1.0 equiv.), Cu(MeCN)$_4$BF$_4$ (12.6 mg, 0.04 mmol, 1.0 equiv.), and 4,4′-dimethoxy-2,2′-bipyridine (8.7 mg, 0.04 mmol, 1.0 equiv.). The vial was sealed with a septum cap and placed under an N$_2$(g) atmosphere. The contents of the vial were evacuated and refilled with N$_2$(g) (x 3) to ensure the presence of an inert atmosphere. To the vial was then added DMF-d$_7$ (0.5 mL), and the contents were again evacuated and refilled with N$_2$(g). The contents were shaken and transferred to an NMR tube which was also under an inert N$_2$(g)atmosphere. A ^{19}F{^{1}H} NMR spectra was recorded immediately, and the tube was subsequently placed in a sand bath at 50 °C for 30 min. Another ^{19}F{^{1}H} NMR spectrum was recorded, followed by placement of the tube into a sand bath at 110 °C for 30 min. One final ^{19}F{^{1}H} NMR spectrum was recorded. The observed (significant) resonances at each time point are as follows, with a figure of the time course below (-100 to -160 ppm range).

^{19}F{^{1}H} NMR (376 MHz, *N,N*-dimethylformamide-d$_7$):

Timepoint 1, t = 0 h at 20°C: δ_F ppm -114.2 **[ArB(OH)$_2$]**, -116.9 **[Ar–Ar]**, -150.8 **[BF$_4^-$]**;
Timepoint 2, +30 min at 50°C: δ_F ppm -114.2 **[ArB(OH)$_2$]**, -116.9 **[Ar–Ar]**, -121.3 **[LCuAr]**, -150.8 **[BF$_4^-$]**;
Timepoint 3, +30 min at 110°C: δ_F ppm -114.2 **[ArB(OH)$_2$]**, -116.9 **[Ar–Ar]**, -121.3 **[LCuAr]**, -150.8 **[BF$_4^-$]**, -160.1 **[BF$_3$-DABCO adduct]**;

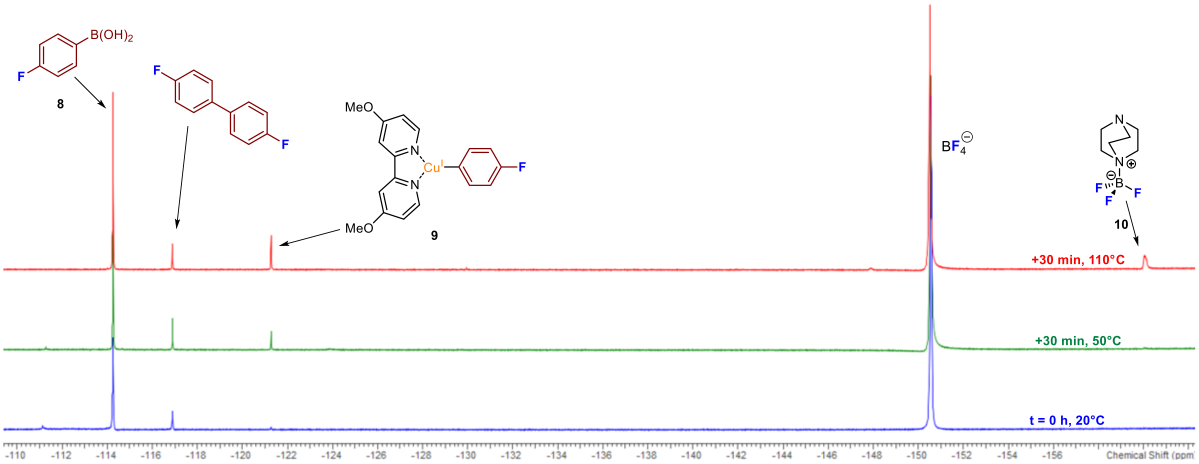

Conclusions: Upon gradual heating, the appearance of a new resonance at δ_F = -121.3 ppm occurs, within the region expected for a *p*-fluorophenyl copper(I) species.[11] The intensity of this peak grows as the reaction is heated to 110°C, providing greater thermal energy for activation. Additionally observed is a species corresponding to homocoupled 4-fluorophenylboronic acid **8**, 4,4'-difluorobiphenyl, at δ_F = -116.9 ppm. The formation of a peak at δ_F = -160.1 ppm is consistent with the fluoride-activation mechanism of the boronic acid, identified as the DABCO-BF_3 adduct species **10**.[9]

4.4. Insertion of SO_2(g) into the copper(I)–aryl bond

4.4.1. Effect of boronic acid R group on the migratory insertion process

In order to observe experimentally the effect of variation of the boronic acid electronics on the reactivity towards migratory insertion, a small number of reactions with varying boronic acid coupling partners were performed.

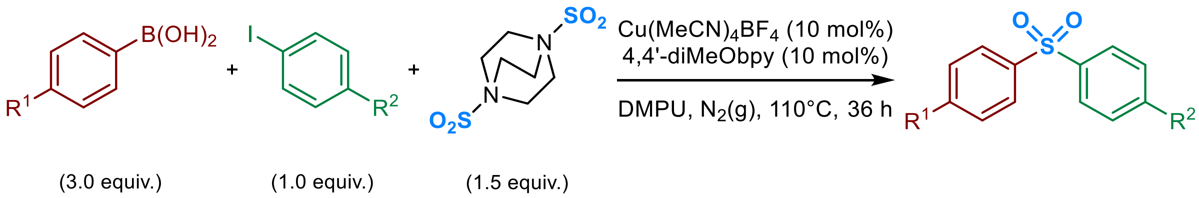

General procedure:

To a microwave vial containing arylboronic acid (1.2 mmol, 3.0 equiv.), $Cu(MeCN)_4BF_4$ (12.6 mg, 0.04 mmol, 10 mol%), 4,4'-dimethoxy-2,2'-bipyridine (8.7 mg, 0.04 mmol, 10 mol%) and DABSO (144.2 mg, 0.6 mmol, 1.5 equiv.) was added a solution of aryl iodide (0.4 mmol, 1.0 equiv.) in DMPU (2 mL). The reaction was heated to 110 °C and left to stir for 36 h under N_2(g). Upon completion, the mixture was allowed to cool to room temperature, quenched with H_2O (10 mL), and was subsequently extracted with ethyl acetate (2×10 mL). The combined organic phases were washed with water (10 mL) and brine (10 mL), passed through a hydrophobic frit, and concentrated *in vacuo*. The crude product was purified by normal-phase flash column chromatography on a Redi*Sep*® pre-packed 12 g silica cartridge (cyclohexane, ethyl acetate). UV active fractions were analysed by LCMS with product-containing fractions combined and concentrated *in vacuo* to give the titled compound.

Entry	Mass of arylboronic acid (mg)	Mass/volume of aryl iodide	Product	Isolated yield (%)
1	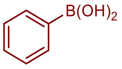 146.0 mg	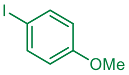 93.6 mg	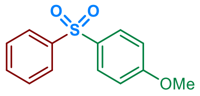	42
2	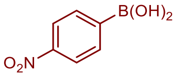 200.0 mg	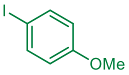 93.6 mg	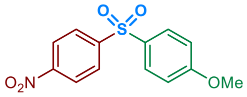	3
3	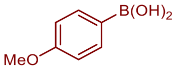 182.0 mg	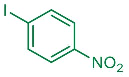 99.6 mg	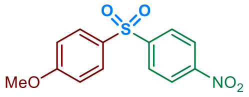	39
4	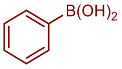 146.0 mg	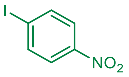 99.6 mg	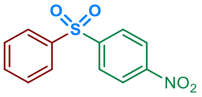	15

1-Methoxy-4-(phenylsulfonyl)benzene

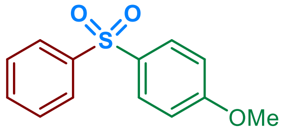

Prepared according to the general procedure using phenylboronic acid (146.0 mg, 1.2 mmol, 3.0 equiv.), 4-iodoanisole (93.6 mg, 0.4 mmol, 1.0 equiv.), tetrakis(acetonitrile) copper(I) tetrafluoroborate (12.6 mg, 0.04 mmol, 10 mol%), 4,4'-dimethoxy-2,2'-bipyridine (8.7 mg, 0.04 mmol, 10 mol%), 1,4-diazabicyclo[2.2.2]octane bis(sulfur dioxide) adduct (144.0 mg, 0.6 mmol, 1.5 equiv.) and DMPU (2 mL). The crude product was purified by normal-phase flash column chromatography on a Redi*Sep*® pre-packed 12 g silica cartridge (cyclohexane, 0-10% ethyl acetate) to yield the desired product as an off-white solid (42.0 mg, 42%).

^{1}H NMR (400 MHz, $CDCl_3$) δ_H ppm 7.86-7.94 (m, 4H), 7.53 (tt, *J*=6.8, 2.0 Hz, 1H), 7.48 (m, 2H), 6.97 (dt, *J*=8.8, 2.9 Hz, 2H), 3.84 (s, 3H); **^{13}C NMR** (101 MHz, $CDCl_3$) δ_C ppm 163.4, 142.4, 133.1, 132.8, 129.8, 129.1, 127.3, 114.5, 55.6; **LCMS** (Formic, low pH): $[M+H]^+$ observed at 249.26, t_{ret} = 1.02 min, 100% purity; The analytical data were in accordance with the literature.[2]

1-Methoxy-4-((4-nitrophenyl)sulfonyl)benzene

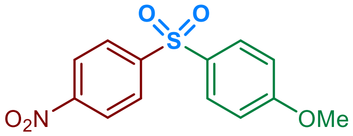

Prepared according to the general procedure using 4-nitrophenylboronic acid (200.0 mg, 1.2 mmol, 3.0 equiv.), 4-iodoanisole (93.6 mg, 0.4 mmol, 1.0 equiv.), tetrakis(acetonitrile) copper(I) tetrafluoroborate (12.6 mg, 0.04 mmol, 10 mol%), 4,4'-dimethoxy-2,2'-bipyridine (8.7 mg, 0.04 mmol, 10 mol%), 1,4-diazabicyclo[2.2.2]octane bis(sulfur dioxide) adduct (144.0 mg, 0.6 mmol, 1.5 equiv.) and DMPU (2 mL). The crude product was purified by normal-phase flash column chromatography on a Redi*Sep*® pre-packed 12 g silica cartridge (cyclohexane, 0-10% ethyl acetate) to yield the desired product as an off-white solid (3.5 mg, 3%).

^{1}H NMR (400 MHz, $CDCl_3$) δ_H ppm 8.31-8.35 (m, 2H), 8.08-8.12 (m, 2H), 7.88-7.93 (m, 2H), 6.99-7.04 (m, 2H), 3.87 (s, 3H); **^{13}C NMR** (101 MHz, $CDCl_3$) δ_C ppm 164.1, 150.1, 148.2, 130.3, 129.4, 128.6, 124.4,

114.9, 55.8; **LCMS** (Formic, low pH): $[M+H]^+$ observed at 294.04, t_{ret} = 1.05 min, 89% purity; The analytical data were in accordance with the literature.[12]

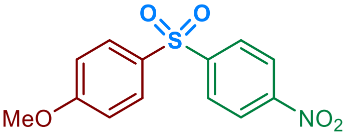

Prepared according to the general procedure using 4-methoxyphenylboronic acid (182.0 mg, 1.2 mmol, 3.0 equiv.), 1-iodo-4-nitrobenzene (99.6 mg, 0.4 mmol, 1.0 equiv.), tetrakis(acetonitrile) copper(I) tetrafluoroborate (12.6 mg, 0.04 mmol, 10 mol%), 4,4'-dimethoxy-2,2'-bipyridine (8.7 mg, 0.04 mmol, 10 mol%), 1,4-diazabicyclo[2.2.2]octane bis(sulfur dioxide) adduct (144.0 mg, 0.6 mmol, 1.5 equiv.) and DMPU (2 mL). The crude product was purified by normal-phase flash column chromatography on a Redi*Sep*® pre-packed 12 g silica cartridge (cyclohexane, 0-15% ethyl acetate) to yield the desired product as an off-white solid (45.7 mg, 39%).

^{1}H NMR (400 MHz, $CDCl_3$) δ_H ppm 8.33 (dt, *J*=8.8, 2.9 Hz, 2H), 8.10 (m, 2H), 7.91 (m, 2H), 7.02 (m, 2H), 3.87 (s, 3H); **^{13}C NMR** (101 MHz, $CDCl_3$) δ_C ppm 164.1, 150.1, 148.2, 131.4, 130.3, 128.6, 124.4, 114.9, 55.8; **LCMS** (Formic, low pH): $[M+H]^+$ not observed, t_{ret} = 1.06 min, 100% purity; The analytical data were in accordance with the literature.[12]

1-Nitro-4-(phenylsulfonyl)benzene

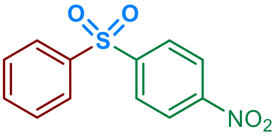

Prepared according to the general procedure using phenylboronic acid (146.0 mg, 1.2 mmol, 3.0 equiv.), 1-iodo-4-nitrobenzene (99.6 mg, 0.4 mmol, 1.0 equiv.), tetrakis(acetonitrile) copper(I) tetrafluoroborate (12.6 mg, 0.04 mmol, 10 mol%), 4,4'-dimethoxy-2,2'-bipyridine (8.7 mg, 0.04 mmol, 10 mol%), 1,4-diazabicyclo[2.2.2]octane bis(sulfur dioxide) adduct (144.0 mg, 0.6 mmol, 1.5 equiv.) and DMPU (2 mL). The crude product was purified by normal-phase flash column chromatography on a Redi*Sep*® pre-packed 12 g silica cartridge (cyclohexane, 0-10% ethyl acetate) to yield the desired product as an off-white solid (15.5 mg, 15%).

^{1}H NMR (400 MHz, $CDCl_3$) δ_H ppm 8.32-8.37 (m, 2H), 8.11-8.17 (m, 2H), 7.95-8.02 (m, 2H), 7.62-7.68 (m, 1H), 7.53-7.61 (m, 2H); **^{13}C NMR** (101 MHz, $CDCl_3$) δ_C ppm 150.4, 147.4, 140.1, 134.1, 129.7, 129.0, 128.0, 124.5; **LCMS** (Formic, low pH): $[M+H]^+$ not observed, t_{ret} = 1.04 min, 100% purity; The analytical data were in accordance with the literature.[13]

4.4.2. $^{19}F\{^1H\}$ NMR time course: formation of 4-fluorobenzenesulfinate from insertion of SO_2(g)

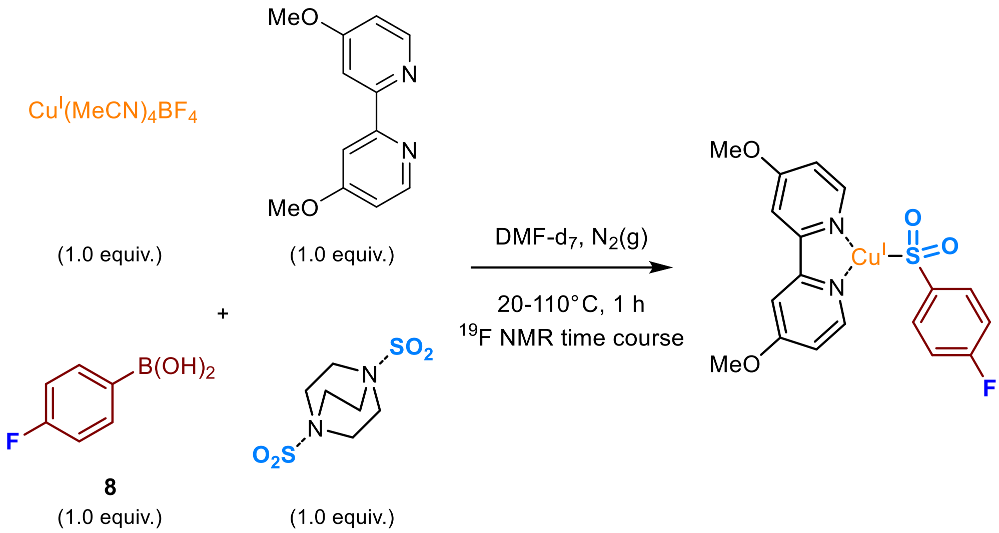

To a 4 mL vial was added 4-fluorophenylboronic acid (7.0 mg, 0.05 mmol, 1.0 equiv.), 1,4-diazabicyclo[2.2.2]octane bis(sulfur dioxide) adduct (12.0 mg, 0.05 mmol, 1.0 equiv.), tetrakis(acetonitrile) copper(I) tetrafluoroborate (15.7 mg, 0.05 mmol, 1.0 equiv.), and 4,4'-dimethoxy-2,2'-bipyridine (10.8 mg, 0.05 mmol, 1.0 equiv.). The vial was sealed with a septum cap and placed under a N_2(g) atmosphere. The contents of the vial were evacuated and refilled with N_2(g) (x 3) to ensure the presence of an inert atmosphere. To the vial was then added DMF-d_7 (0.5 mL), and the contents were again evacuated and refilled with N_2(g). The contents were shaken and transferred to an NMR tube which was also under an inert N_2(g) atmosphere. A $^{19}F\{^1H\}$ NMR spectra was recorded immediately, and the tube was subsequently placed in a sand bath at 50°C for 30 min. Another $^{19}F\{^1H\}$ NMR spectrum was recorded, followed by placement of the tube into a sand bath at 110°C for 30 min. One final $^{19}F\{^1H\}$ NMR spectrum was recorded. The observed (significant) resonances at each time point are as follows, with a figure of the time course below (-100 to -160 ppm range).

$^{19}F\{^1H\}$ NMR (376 MHz, *N,N*-dimethylformamide-d_7):

Timepoint 1, t = 0 h at 20°C: δ_F ppm -114.2 **[ArB(OH)$_2$]**, -116.9 **[Ar-Ar]**, -150.8 **[BF_4^-]**;
Timepoint 2, +30 min at 50°C: δ_F ppm -113.9 **[LCuSO$_2$Ar]**, -114.2 **[ArB(OH)$_2$]**, -115.5 **[LCuOS(O)Ar]**, -116.9 **[Ar-Ar]**, -150.8 **[BF_4-]**;
Timepoint 3, +30 min at 110°C: δ_F ppm -114.2 **[ArB(OH)$_2$]**, -115.5 **[LCuOS(O)Ar]**, -116.9 **[Ar-Ar]**, -150.8 **[BF_4^-], **-160.1 **[BF_3-DABCO adduct]**;

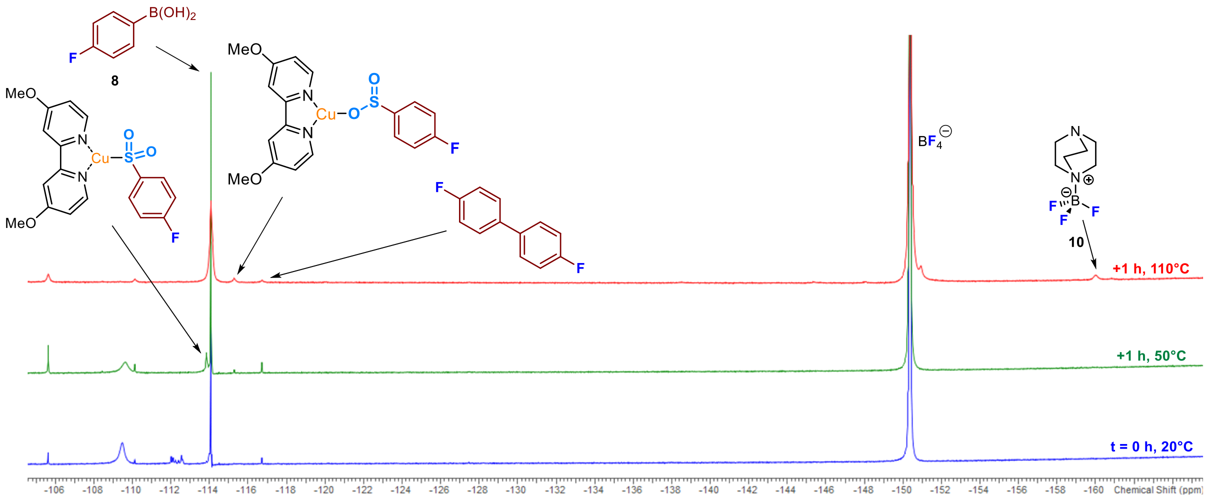

Conclusions: Upon heating of the reaction mixture in an NMR tube, additional weak resonances were observed at -113.9 and -115.5 ppm, resembling the metal-bound *S*- and *O*-sulfinate species, respectively. Additional products observed are the homocoupled boronic acid species, 4,4'-difluorobiphenyl, and a DABCO-BF_3 adduct as a result of the transmetalation process. Interestingly, absence of a resonance at -121.3 ppm (LCuAr species) suggests that upon binding of SO_2(g) to the copper species, migratory insertion is rapid.

4.4.3. $^{19}F\{^1H\}$ NMR study: interaction of 4-fluorobenzenesulfinate with copper(I)

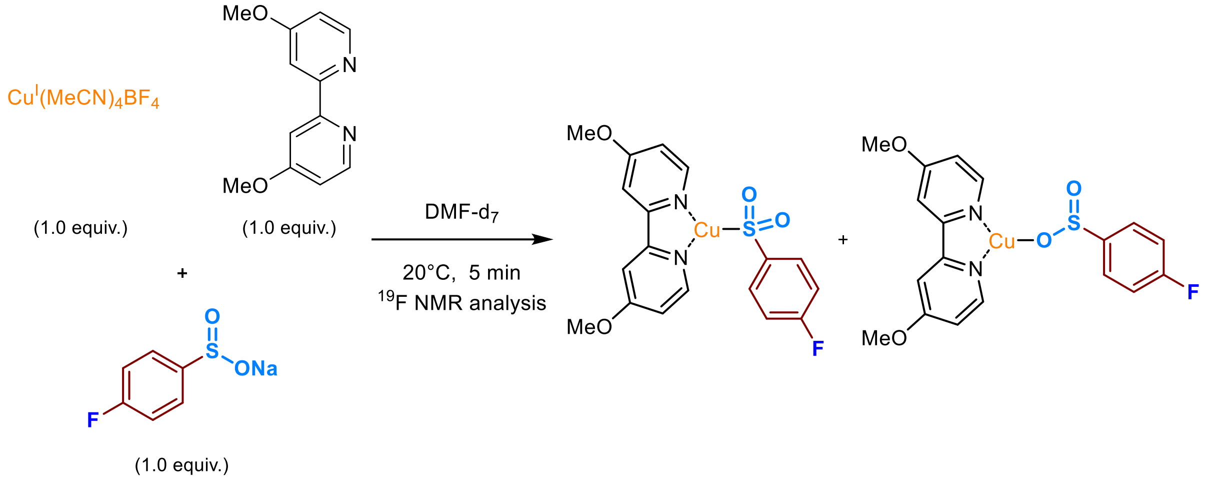

To a 4 mL vial was added sodium 4-fluorobenzenesulfinate (9.1 mg, 0.05 mmol, 1.0 equiv.), $Cu(MeCN)_4BF_4$ (15.7 mg, 0.05 mmol, 1.0 equiv.), 4-iodotoluene (10.9 mg, 0.05 mmol, 1.0 equiv.), and 4,4'-dimethoxy-2,2'-bipyridine (10.8 mg, 0.05 mmol, 1.0 equiv.). The vial was sealed with a septum cap and placed under a N_2(g) atmosphere. The contents of the vial were evacuated and refilled with N_2(g) (x 3) to ensure the presence of an inert atmosphere. To the vial was then added DMF-d_7, and the contents were again evacuated and refilled with N_2(g). The contents were shaken and transferred to an NMR tube which was also under an inert N_2(g) atmosphere. A $^{19}F\{^1H\}$ NMR spectrum was recorded of the mixture, in addition to a sample of the sodium 4-fluorobenzenesulfinate starting material, shown below.

$^{19}F\{^1H\}$ NMR (376 MHz, *N,N*-dimethylformamide-d_7):

Commercial sodium 4-fluorobenzenesulfinate: δ_F ppm -116.7 **[reference Na *O*-sulfinate]**;
Reaction mixture: δ_F ppm -113.9 **[*S*-sulfinate]**, -115.5 **[*O*-sulfinate]**;

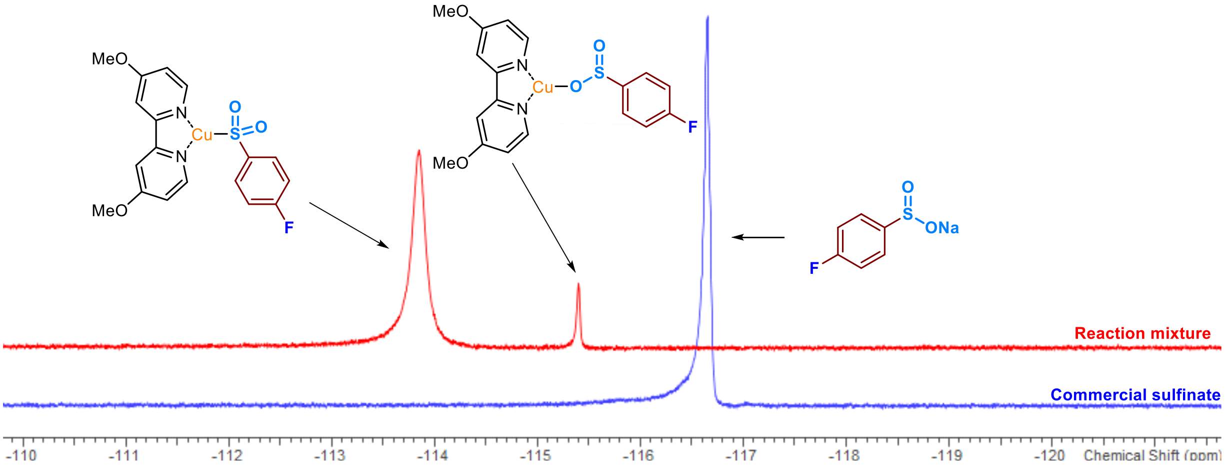

Conclusions: Upon mixing sodium 4-fluorobenzenesulfinate, tetrakis(acetonitrile) copper(I) tetrafluoroborate, and 4,4'-dimethoxy-2,2'-bipyridine in DMF-d_7 the single resonance at -116.7 ppm is observed to split into two individual peaks at -113.9 and -115.5 ppm. Given the known binding modes of sulfinates to metal species, it can be proposed that these two peaks represent the *O*- and *S*-bound sulfinate species. These results also suggest that the sodium cation is exchanged for copper(I), given the shifts in δ_F ppm. Computational calculations of ^{19}F NMR shielding constants suggest the identities of each peak, with the ratio of species arising from the relative stabilities of the two species (see Computational Supporting Information Section 2.4).

4.5. Oxidative addition of copper(I) into the carbon-iodine bond

4.5.1. Effect of aryl halide on the yield of desired sulfone product: proof of a copper(I)/copper(III) two-electron catalytic cycle

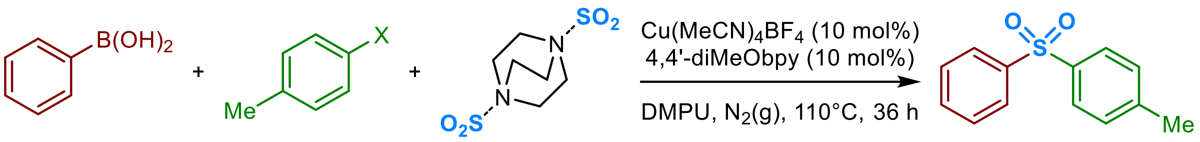

4 (3.0 equiv.) | X = I, Br, Cl, OTf (1.0 equiv.) | (1.5 equiv.) | **3**

To 12 HPLC vials (4 × 3), stock solutions of Cu(MeCN)$_4$BF$_4$ (47.2 mg in 6 mL MeOH, 200 µL aliquots), 4,4'-diMeObpy (32.4 mg in 6 mL CH_2Cl_2, 200 µL aliquots), and phenylboronic acid (548.7 mg in 12 mL CH_2Cl_2, 400 µL aliquots) were concentrated under a stream of N_2(g) into each vial prior to addition of 1,4-diazabicyclo[2.2.2]octane bis(sulfur dioxide) adduct (18.0 mg, 0.075 mmol). Stock solutions of 4-tolyl halide or psuedohalide (0.25 mmol) in DMPU sample (1250 µL) was added to the respective vials in 250 µL aliquots (consisting of 0.05 mmol of 4-tolyl halide or psuedohalide and 250 µL DMPU) as a final step. Vials 1-3 contained 4-iodotoluene, 4-6 4-bromotoluene, 7-9 4-chlorotoluene, and 9-12 *p*-tolyl trifluoromethanesulfonate. Each reaction therefore contained a fixed quantity of Cu(MeCN)$_4$BF$_4$ (1.6 mg, 5 µmol, 10 mol%), 4,4'-diMeObpy (1.1 mg, 5 µmol, 10 mol%), phenylboronic acid (18.3 mg, 0.15 mmol), 1,4-diazabicyclo[2.2.2]octane bis(sulfur dioxide) adduct (18.0 mg, 0.075 mmol), and 4-tolyl halide or psuedohalide (0.05 mmol). The vials were sealed with septa screwcaps and placed into a CAT96 instrument. The plate was heated to 110°C for 36 h under N_2(g) (5 bar), after which it was allowed to cool to room temperature. Each vial was quenched with a stock solution of dimethyl terephthalate (0.75 mL, 0.0133 M solution in MeCN, 0.01 mmol). The resulting solutions were sonicated, and a 50 µL aliquot was taken and diluted in a HPLC vial with MeCN (950 µL). Yields were

determined by HPLC (λ = 235 nm) using % area and pre-determined K_x values for the major reaction components.

Entry	Aryl halide or pseudohalide	HPLC area$_{IS}$ (%)	HPLC area sulfone 3 (%)	HPLC yield sulfone 3 (%)	Mean yield sulfone 3 (%)
1		11.005	22.465	38.2	
2	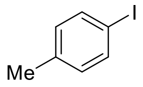	10.264	21.967	40.1	**40.5**
3		10.064	23.120	43.0	
4		15.134	1.388	1.7	
5	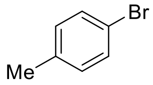	15.426	1.336	1.6	**1.8**
6		14.656	1.692	2.2	
7		16.056	0.055	0.1	
8	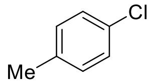	15.711	0.068	0.1	**0.1**
9		16.025	0.103	0.1	
10		16.192	0.082	0.1	
11	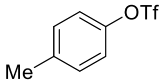	15.744	0.103	0.1	**0.1**
12		15.477	0.058	0.1	

* Solution yields determined by HPLC (λ = 235 nm) using % area of internal standard, % area of reaction component, and pre-determined K_x values for the major reaction components (Section 3).

Conclusions: Based on the obtained mean HPLC yields of unsymmetrical sulfone for each aryl halide or psuedohalide, the reactivity follows the trend of C–I > C–Br > C–Cl ~ C–OTf. These observations point towards a copper(I)/copper(III) two electron oxidative addition pathway for reactivity in the sulfonylative Suzuki-Miyaura reaction.

4.5.2. $^{19}F\{^1H\}$ NMR timecourse: reaction of sodium 4-fluorobenzenesulfinate with iodobenzene

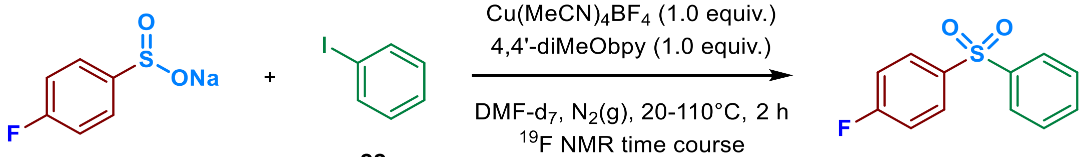

(1.0 equiv.) **22** (1.0 equiv.)

To a 4 mL vial was added sodium 4-fluorobenzenesulfinate (9.1 mg, 0.05 mmol, 1.0 equiv.), iodobenzene (5.6 µL, 0.05 mmol, 1.0 equiv.), $Cu(MeCN)_4BF_4$ (15.7 mg, 0.05 mmol, 1.0 equiv.), and 4,4'-dimethoxy-2,2'-bipyridine (10.8 mg, 0.05 mmol, 1.0 equiv.). The vial was sealed with a septa screw-cap. The contents were evacuated and refilled with N_2(g) (x 3), and to the vial was then added DMF-d_7 (0.4 mL). The contents were shaken and sonicated, and then transferred to an NMR tube which was also under a N_2(g) atmosphere. The tube was sealed and an initial $^{19}F\{^1H\}$ NMR spectrum was recorded. The tube was transferred to a sand bath at 50 °C, in which it was heated for 1 h. After this time, the contents were shaken, and another $^{19}F\{^1H\}$ NMR spectrum was recorded. This process was repeated, heating at 110 °C for an additional hour, prior to recording a final spectrum. The observed (significant) resonances at each time point are as follows, with a figure of the time course below (-100 to -130 ppm range).

$^{19}F\{^1H\}$ NMR (376 MHz, *N,N*-dimethylformamide-d_7):

Timepoint 1, t = 0 h at 20°C: δ_F ppm -106.1 **[sulfone]**, -113.9 **[*S*-sulfinate]**, -115.5 **[*O*-sulfinate]**, -150.8 **[BF_4^-]**;
Timepoint 2, +1 h at 50°C: δ_F ppm -105.9, -106.1 **[sulfone]**, -111.6, -115.0, -115.8, -150.8 **[BF_4^-]**;
Timepoint 3, +1 h at 110°C: δ_F ppm -105.9, -106.1 **[sulfone]**, -114.4, -115.6, -115.8, -127.6 **[suspected Cu^{III} species]**, -150.8 **[BF_4^-]**;

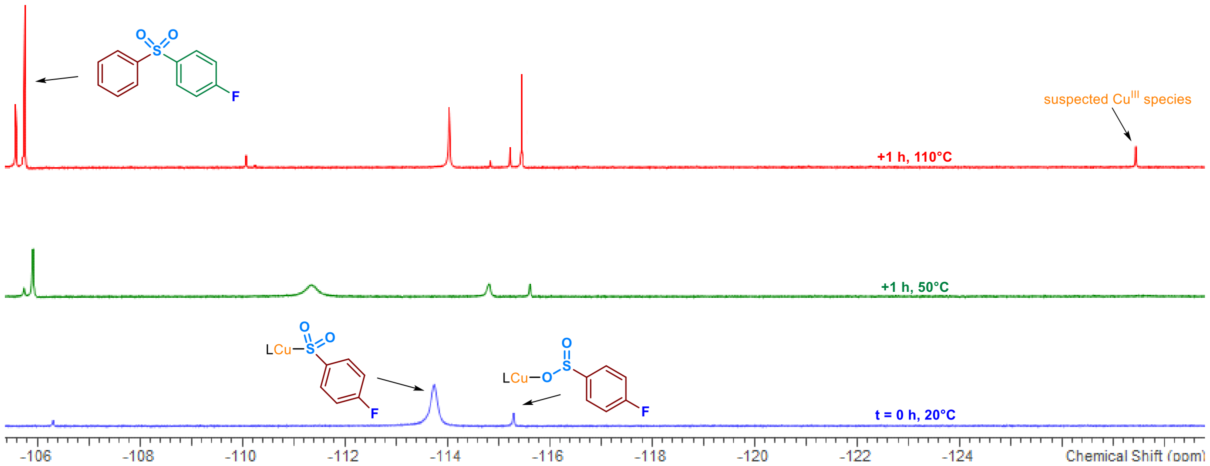

Conclusions: Upon heating of the NMR tube containing reaction mixture, the two sulfinate peaks, observed at t = 0 h disappear, and formation of the desired sulfone product is seen. Using the synthesised reference compounds of 4-fluoro-1,1'-biphenyl and *S*-(4-fluorophenyl) 4-fluorobenzenethiosulfonate, it can be confirmed that no desulfinylative coupling, or disproportionation, of the sodium sulfinate species is occurring. The resonance at -127.6 ppm is suspected to be a transient copper(III) species, although this has not been confirmed. It has not yet been possible to identify other species within the reaction mixture at the third time point.

4.5.3. ^{19}F{^{1}H} NMR timecourse: reaction of sodium benzenesulfinate with 1-fluoro-4-iodobenzene

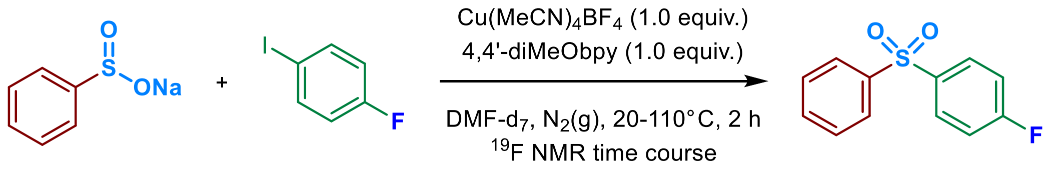

1

(1.0 equiv.) (1.0 equiv.)

To a 4 mL vial was added sodium benzenesulfinate (8.2 mg, 0.05 mmol, 1.0 equiv.), 1-fluoro-4-iodobenzene (5.77 µL, 0.05 mmol, 1.0 equiv.), $Cu(MeCN)_4BF_4$ (16 mg, 0.05 mmol, 1.0 equiv.), and 4,4'-dimethoxy-2,2'-bipyridine (10.8 mg, 0.05 mmol, 1.0 equiv.). The vial was sealed with a septa screw-cap. The contents were evacuated and refilled with N_2(g) (x 3), and to the vial was then added DMF-d_7 (0.3 mL). The contents were shaken and sonicated, and then transferred to an NMR tube which was also under a N_2(g) atmosphere. The tube was sealed and an initial ^{19}F{^{1}H} NMR spectrum was recorded. The tube was transferred to a sand bath at 50 °C, in which it was heated for 1 h. After this time, the contents were shaken, and another ^{19}F{^{1}H} NMR spectrum was recorded. This process was repeated, heating at 110 °C for an additional hour, prior to recording a final spectrum. The observed (significant) resonances at each time point are as follows, with a figure of the time course below (-100 to -130 ppm range).

^{19}F{^{1}H} NMR (376 MHz, *N,N*-dimethylformamide-d$_7$):

Timepoint 1, t = 0 h at 20°C: δ_F ppm -115.8 **[Ar–I]**, -150.8 **[BF_4^-]**;
Timepoint 2, +1 h at 50°C: δ_F ppm -106.1 **[sulfone]**, -115.8 **[Ar–I]**, -150.8 **[BF_4^-]**;
Timepoint 3, +1 h at 110°C: δ_F ppm -105.9, -106.1 **[sulfone]**, -114.4, -115.2, -115.8 **[Ar–I]**, -127.8 **[suspected Cu^{III} species]**, -150.8 **[BF_4^-]**;

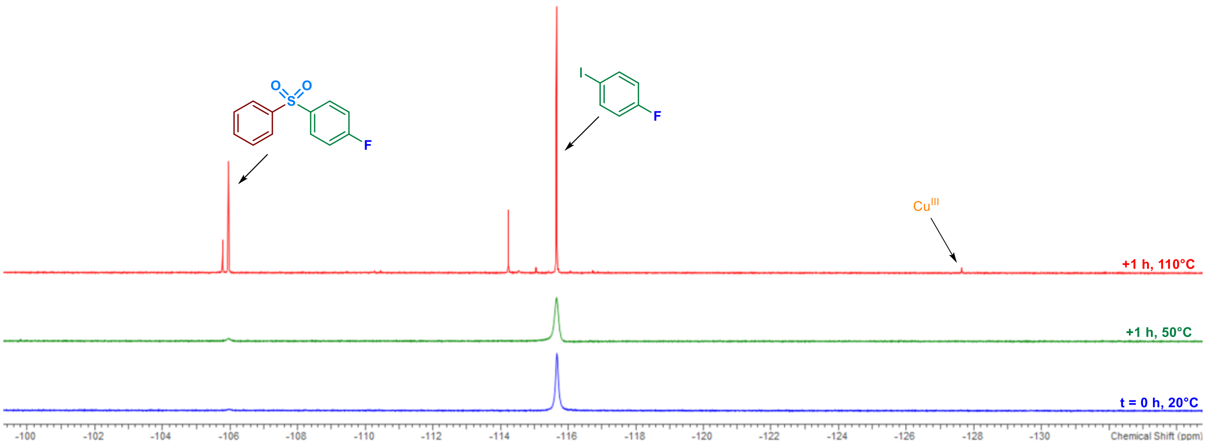

Conclusions: Again, as observed with the fluorinated sulfinate species, unidentifiable resonances at -105.9 and -114.4 ppm are observed after heating at 110°C for 1 h. These could arise from disproportionation side-reactions (experimental supporting information Section 4.6). The resonance at -127.8 ppm is again suspected to be a transient copper(III) species, although this has not been confirmed.

4.5.4. Effect of complex ligand on the oxidative addition of copper(I) into the carbon-iodine bond

In order to observe experimentally the effect of variation of complex ligand on the reactivity towards oxidative addition, 3 reactions with functionalised 2,2'-bipyridyl ligands were performed, starting from commercially sourced sodium benzenesulfinate. One reaction was additionally carried out in the absence of any ligand.

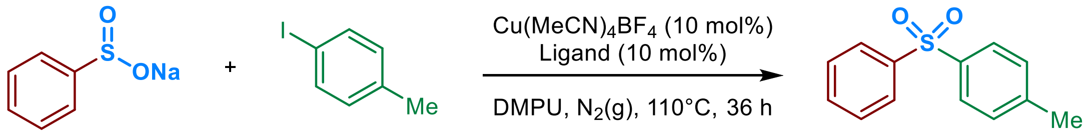

General procedure:

To a microwave vial containing sodium benzenesulfinate (49 mg, 0.3 mmol, 3.0 equiv.), $Cu(MeCN)_4BF_4$ (3.2 mg, 0.01 mmol, 10 mol%), and ligand (0.01 mmol, 10 mol%) was added a solution of 4-iodotoluene (21.8 mg, 0.1 mmol, 1.0 equiv.) in DMPU (0.5 mL). The reaction was heated to 110 °C and left to stir for 36 h under N_2(g). Upon completion, the mixture was allowed to cool to room temperature, and then quenched with a stock solution of dimethyl terephthalate (1.5 mL, 0.0133 M solution in acetonitrile, 0.02 mmol). The resulting solution was sonicated, and a 50 µL aliquot was taken and diluted in a HPLC vial with acetonitrile (950 µL). Yields were determined by HPLC (λ = 235 nm) using % area and pre-determined K_x values for the major reaction components.

Entry	Ligand (mass)	HPLC area$_{IS}$ (%)	HPLC area sulfone 3 (%)	HPLC yield sulfone 3 (%)
1	–	13.878	4.195	6
2	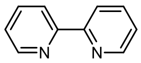 bpy (1.5 mg)	13.587	7.155	10
3	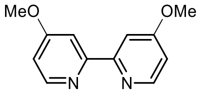 4,4’-diMeObpy (2.2 mg)	14.276	12.110	16
4	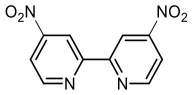 4,4’-diNO$_2$bpy (2.5 mg)	13.844	7.380	10

* Solution yields determined by HPLC (λ = 235 nm) using % area of internal standard, % area of reaction component, and pre-determined K_x values for the major reaction components (Section 3).

Conclusions: For the model reaction converting sodium benzenesulfinate and 4-iodotoluene to 1-methyl-4-(phenylsulfonyl)benzene, experimental results appear to agree with computational predictions. For electron-donating ligands such as 4,4’-diMeObpy, the increased electron density provided to the copper(I) centre results in higher yields of the cross-coupled product. For electron-deficient ligands, effectiveness in this transformation is decreased, in line with decreased electron density on copper(I).

4.5.5. Effect of aryl iodide on the oxidative addition of copper(I) into the carbon-iodine bond

In order to observe experimentally the effect of variation of the aryl iodide electronics on the reactivity towards oxidative addition, 5 reactions with varying aryl iodide coupling partners were performed, starting from commercially sourced sodium benzenesulfinate.

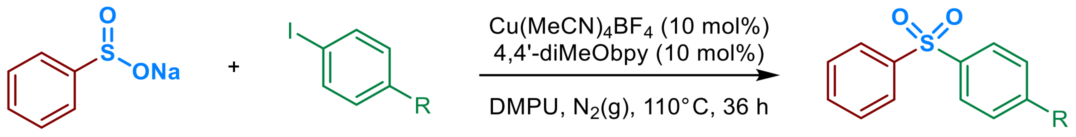

1
3.0 equiv.

1.0 equiv.

General procedure:

To a microwave vial containing sodium benzenesulfinate (197.0 mg, 1.2 mmol, 3.0 equiv.), $Cu(MeCN)_4BF_4$ (12.6 mg, 0.04 mmol, 10 mol%), and 4,4’-dimethoxy-2,2’-bipyridine (8.7 mg, 0.04 mmol, 10 mol%) was added a solution of aryl iodide (0.4 mmol, 1.0 equiv.) in DMPU (2 mL). The reaction was heated to 110 °C and left to stir for 36 h under N_2(g). Upon completion, the mixture was allowed to cool to room temperature, quenched with H_2O (5 mL), and was subsequently extracted with ethyl acetate (2×10 mL). The combined organic phases were washed with water (10 mL) and brine (10 mL), passed through a hydrophobic frit, and concentrated *in vacuo*. The crude product was purified by normal-phase flash column chromatography on a Redi*Sep*® pre-packed 12 g silica cartridge (cyclohexane, ethyl acetate). UV active fractions were analysed by LCMS with product-containing fractions combined and concentrated *in vacuo* to give the titled compound.

Entry	Mass/volume of aryl iodide	Product	Isolated yield (%)
1	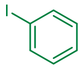 45.0 µL	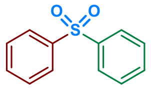	22
2	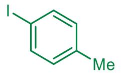 87.0 mg	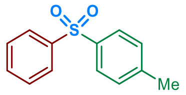	14
3	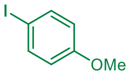 94.0 mg	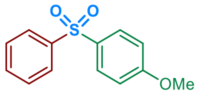	25
4	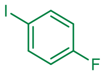 46.0 µL	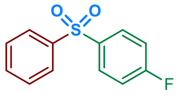	14
5	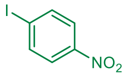 100.0 mg	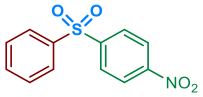	24

Sulfonyldibenzene (20)

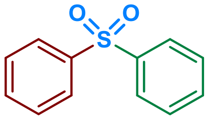

Prepared according to the general procedure using sodium benzenesulfinate (197.0 mg, 1.2 mmol, 3.0 equiv.), iodobenzene (45.0 µL, 0.4 mmol, 1.0 equiv.), tetrakis(acetonitrile) copper(I) tetrafluoroborate (12.6 mg, 0.04 mmol, 10 mol%), 4,4'-dimethoxy-2,2'-bipyridine (8.7 mg, 0.04 mmol, 10 mol%) and DMPU (2 mL). The crude product was purified by normal-phase flash column chromatography on a Redi*Sep*® pre-packed 12 g silica cartridge (cyclohexane, 0-10% ethyl acetate) to yield the desired product as a white solid (19.1 mg, 22%).

^{1}H NMR (400 MHz, $CDCl_3$) δ_H ppm 7.94-7.99 (m, 4H), 7.54-7.60 (m, 2H), 7.49-7.54 (m, 4H); **^{13}C NMR** (101 MHz, $CDCl_3$) δ_C ppm 141.7, 133.1, 129.3, 127.7; **LCMS** (Formic, low pH): $[M+H]^+$ observed at 219.29, t_{ret} = 1.00 min, 100% purity; The analytical data were in accordance with the literature.[2]

1-Methyl-4-(phenylsulfonyl)benzene (3)

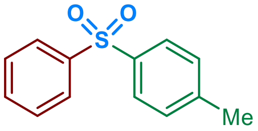

Prepared according to the general procedure using sodium benzenesulfinate (197.0 mg, 1.2 mmol, 3.0 equiv.), 4-iodotoluene (87.0 mg, 0.4 mmol, 1.0 equiv.), tetrakis(acetonitrile) copper(I) tetrafluoroborate (12.6 mg, 0.04 mmol, 10 mol%), 4,4'-dimethoxy-2,2'-bipyridine (8.7 mg, 0.04 mmol, 10 mol%) and DMPU (2 mL). The crude product was purified by normal-phase flash column chromatography on a Redi*Sep*® pre-packed 12 g silica cartridge (cyclohexane, 0-10% ethyl acetate) to yield the desired product as a white solid (13.4 mg, 14%).

^{1}**H NMR** (400 MHz, $CDCl_3$) δ_H ppm 7.92-7.96 (m, 2H), 7.82-7.86 (m, 2H), 7.55 (tt, *J* = 8.3, 2.9 Hz, 1H), 7.28-7.32 (m, 2H), 2.41 (s, 3H); ^{13}**C NMR** (101 MHz, $CDCl_3$) δ_C ppm 144.1, 142.0, 138.7, 132.9, 129.9, 129.2, 127.7, 127.5, 21.5; **LCMS** (Formic, low pH): $[M+H]^+$ observed at 233.34, t_{ret} = 1.08 min, 98% purity; The analytical data were in accordance with the literature.[2]

1-Methoxy-4-(phenylsulfonyl)benzene

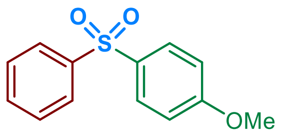

Prepared according to the general procedure using sodium benzenesulfinate (197.0 mg, 1.2 mmol, 3.0 equiv.), 4-iodoanisole (94.0 mg, 0.4 mmol, 1.0 equiv.), tetrakis(acetonitrile) copper(I) tetrafluoroborate (12.6 mg, 0.04 mmol, 10 mol%), 4,4'-dimethoxy-2,2'-bipyridine (8.7 mg, 0.04 mmol, 10 mol%) and DMPU (2 mL). The crude product was purified by normal-phase flash column chromatography on a Redi*Sep*® pre-packed 12 g silica cartridge (cyclohexane, 0-15% ethyl acetate) to yield the desired product as a white solid (24.5 mg, 25%).

^{1}**H NMR** (400 MHz, $CDCl_3$) δ_H ppm 7.87-7.95 (m, 4H), 7.52-7.57 (m, 1H), 7.47-7.52 (m, 2H), 6.95-6.99 (m, 2H), 3.85 (s, 3H); ^{13}**C NMR** (101 MHz, $CDCl_3$) δ_C ppm 163.4, 142.4, 133.2, 132.8, 129.9, 129.2, 127.3, 114.5, 55.6; **LCMS** (Formic, low pH): $[M+H]^+$ observed at 249.32, t_{ret} = 1.02 min, 100% purity; The analytical data were in accordance with the literature.[2]

1-Fluoro-4-(phenylsulfonyl)benzene

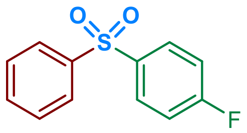

Prepared according to the general procedure using sodium benzenesulfinate (197.0 mg, 1.2 mmol, 3.0 equiv.), 1-fluoro-4-iodobenzene (46.0 µL, 0.4 mmol, 1.0 equiv.), tetrakis(acetonitrile) copper(I) tetrafluoroborate (12.6 mg, 0.04 mmol, 10 mol%), 4,4'-dimethoxy-2,2'-bipyridine (8.7 mg, 0.04 mmol, 10 mol%) and DMPU (2 mL). The crude product was purified by normal-phase flash column chromatography on a Redi*Sep*® pre-packed 12 g silica cartridge (cyclohexane, 0-20% ethyl acetate) to yield the desired product as a white solid (13.1 mg, 14%).

^{1}**H NMR** (400 MHz, $CDCl_3$) δ_H ppm 7.92-8.00 (m, 4H), 7.56-7.62 (m, 1H), 7.49-7.55 (m, 2H), 7.15-7.22 (m, 2H); ^{13}**C NMR** (101 MHz, $CDCl_3$) δ_C ppm 166.7 (d, *J*=256.0 Hz), 141.5, 137.7 (d, *J*=3.7 Hz), 133.3, 130.4 (d, *J*=9.5 Hz), 129.3, 127.6, 116.5 (d, *J*=22.7 Hz); **LCMS** (Formic, low pH): $[M+H]^+$ observed at 237.30, t_{ret} = 1.03 min, 96% purity; The analytical data were in accordance with the literature.[14]

1-Nitro-4-(phenylsulfonyl)benzene

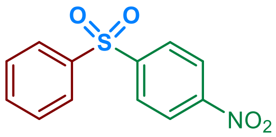

Prepared according to the general procedure using sodium benzenesulfinate (197.0 mg, 1.2 mmol, 3.0 equiv.), 1-iodo-4-nitrobenzene (100.0 mg, 0.4 mmol, 1.0 equiv.), tetrakis(acetonitrile) copper(I) tetrafluoroborate (12.6 mg, 0.04 mmol, 10 mol%), 4,4'-dimethoxy-2,2'-bipyridine (8.7 mg, 0.04 mmol, 10 mol%) and DMPU (2 mL). The crude product was purified by normal-phase flash column

chromatography on a Redi*Sep*® pre-packed 12 g silica cartridge (cyclohexane, 0-40% ethyl acetate) to yield the desired product as a white solid (24.1 mg, 23%).

^{1}H NMR (400 MHz, $CDCl_3$) δ_H ppm 8.32-8.37 (m, 2H), 8.11-8.17 (m, 2H), 7.95-8.02 (m, 2H), 7.62-7.68 (m, 1H), 7.53-7.61 (m, 2H); **^{13}C NMR** (101 MHz, $CDCl_3$) δ_C ppm 150.4, 147.4, 140.1, 134.1, 129.7, 129.0, 128.0, 124.5; **LCMS** (Formic, low pH): $[M+H]^+$ not observed, t_{ret} = 1.04 min, 81% purity; The analytical data were in accordance with the literature.[13]

Conclusions: Unfortunately, no correlations with experimental data could be determined for these transformations, where the 4-aryl group on the aryl iodide was varied. Although some substrates with electron-withdrawing groups (R = NO_2) performed more effectively than those with electron-donating groups (R = Me), this trend was not widespread, as seen with the higher yielding R = OMe substrate. These trends can be accounted to the small different in activation values calculated in the computational supporting information Section 4.7. Existing literature, however, does support the observation that electron-rich aryl iodides are expected to perform less effectively in the cross-coupling reactions of sulfinates with aryl iodides.[15]

4.6. Investigation of side-reactions in the sulfonylative Suzuki-Miyaura reaction

4.6.1. Investigation of benzenesulfinate disproportionation within the reaction mixture

4.6.1.1. Karl-Fisher titrations for determination of H_2O content of reaction solvent (DMPU)

A sample of *N,N'*-dimethylpropylene urea (DMPU) was drawn up into a syringe (~0.5 mL) and weighed using a balance. The contents of the syringe were then injected into the instrument, with the syringe again weighed after the injection. The mass of solvent added to the instrument could then be calculated based on the change in mass. The sample was titrated, and based on the mass of injection input into the instrument, a water content of the sample could be calculated. Each result was repeated in triplicate, and a mean water content (%) was calculated.

Entry	Mass of solvent injected (g)	Calculated water content (%)
1	0.5591	0.057
2	0.5134	0.067
3	0.5988	0.072
		**0.0653**

Based on the obtained results, a water content of ~0.06% for the reaction solvent was measured, approximately twice that of proposed by the supplier (≤0.03%). When calculated, this equates to 64 mg of H_2O per 1 mL of DMPU used, or close to 6 times the number of equivalents of sulfinate forming, assuming full conversion of boronic acid to sulfinate. Dissolution of SO_2(g) in this water would result in formation of sulfurous acid (H_2SO_3), which is likely promoting the disproportionation of the sulfinate to the corresponding thiosulfonate.

4.6.1.2. Test-reactions using dried reaction solvent (DMPU)

In order to study the effect of solvent water content on the disproportionation reaction of benzenesulfinate to thiosulfonate **21** a number of reactions were carried out with dried solvent. These reactions were carried out in triplicate, with three solvent grades: an opened bottle, a freshly opened bottle, and vacuum distilled DMPU. For the drying process, DMPU (25 mL) was dried onto activated molecular sieves (4 Å) by distillation under vacuum, refluxing in calcium hydride.

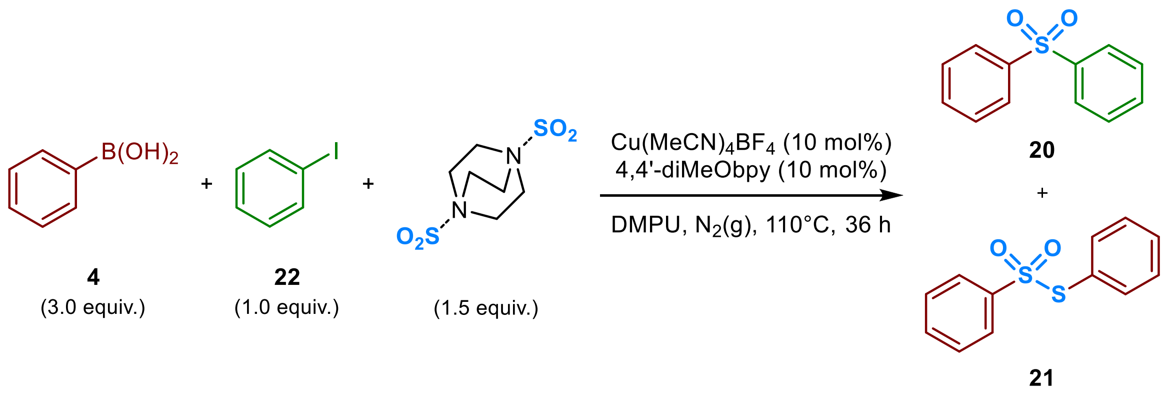

To 9 HPLC vials (3×3), stock solutions of $Cu(MeCN)_4BF_4$ (23.6 mg in 3 mL MeOH, 200 µL aliquots), 4,4'-dimethoxy-2,2'-bipyridine (16.2 mg in 3 mL CH_2Cl_2, 200 µL aliquots), and phenylboronic acid (274 mg in 6 mL CH_2Cl_2, 400 µL aliquots) were concentrated under a stream of N_2(g) into each vial prior to addition of DABSO (18.0 mg, 0.075 mmol). A stock solution of iodobenzene (28 µL) in each DMPU sample (1222 µL) was added to the respective vials in 250 µL aliquots (consisting of 5.6 µL iodobenzene and 244.4 µL DMPU) as a final step. Vials 1-3 contained a DMPU sample from the opened bottle, 4-6 a sample from the freshly opened bottle, and 7-9 a sample of vacuum-distilled DMPU Each reaction therefore contained a fixed quantity of $Cu(MeCN)_4BF_4$ (1.6 mg, 5 µmol, 10 mol%), 4,4'-dimethoxy-2,2'-bipyridine (1.1 mg, 5 µmol, 10 mol%), phenylboronic acid (18.3 mg, 0.15 mmol), DABSO (18.0 mg, 0.075 mmol), and iodobenzene (5.6 µL, 0.05 mmol). The vials were sealed with septa screwcaps and placed into a CAT96 instrument. The plate was heated to 110 °C for 36 h under N_2(g) (5 bar), after which it was allowed to cool to room temperature. Each vial was quenched with a stock solution of dimethyl terephthalate (0.75 mL, 0.0133 M solution in acetonitrile, 0.01 mmol). The resulting solutions were sonicated, and a 50 µL aliquot was taken and diluted in a HPLC vial with acetonitrile (950 µL). Yields were determined by HPLC (λ = 235 nm) using % area and pre-determined K_x values for the major reaction components.

Entry	Solvent sample	HPLC area$_{IS}$ (%)	HPLC area sulfone 20 (%)	HPLC yield sulfone 20 (%)	HPLC area thiosulfonate 21 (%)	HPLC yield thiosulfonate 21 (%)
1	Opened	14.051	32.840	**48.6**	7.108	**9.3**
2		15.914	25.108	**32.8**	4.494	**5.2**
3		13.760	31.354	**47.4**	6.605	**8.8**
4	Fresh	14.669	31.512	**44.6**	5.413	**6.8**
5		12.829	32.670	**52.9**	6.531	**9.3**
6		14.631	29.731	**42.2**	5.087	**6.4**
7	Vacuum-distilled	16.566	23.309	**29.2**	2.030	**2.2**
8		13.334	30.382	**47.4**	4.826	**6.6**
9		15.590	27.586	**36.8**	4.529	**5.3**

* Solution yields determined by HPLC (λ = 235 nm) using % area of internal standard, % area of reaction component, and pre-determined K_x values for the major reaction components (Section 3).

Entry	Solvent sample	Mean yield sulfone 20 (%)	Mean yield thiosulfonate 21 (%)	Mean ratio 20:21
1	Opened	42.9	7.7	**5.6**
2	Fresh	46.6	7.5	**6.2**
3	Vacuum distilled	37.8	4.7	**8.0**

Conclusions: Based on the obtained HPLC yields of the thiosulfonate by-product for each entry, the yield appears to somewhat decrease in line with water content. Given the hygroscopic nature of the

solvent DMPU, the use of a fresh bottle resulted in slightly lower yields of the thiosulfonate, whereas there is a more appreciable decrease in yield upon rigorous drying of the reaction solvent.

4.6.1.3. Test-reaction using a commercially available sodium benzenesulfinate intermediate

In order to prove the involvement of DABSO (SO_2) in promoting the disproportionation of the benzenesulfinate reaction intermediate to form *S*-phenyl benzenethiosulfonate, a test reaction using the commercially available sulfinate salt and iodobenzene was performed, to observe if the quantity of the side-product decreased in the absence of SO_2.

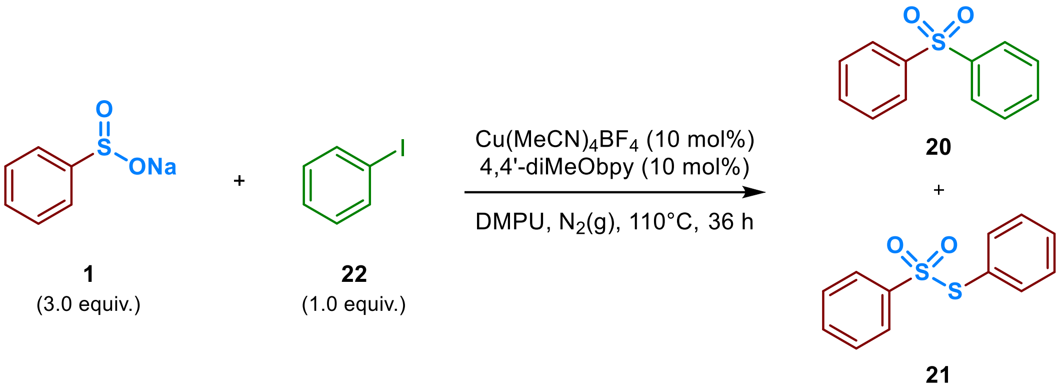

To a microwave vial containing sodium benzenesulfinate (82.0 mg, 0.5 mmol, 1.0 equiv.), iodobenzene (56 µL, 0.5 mmol, 1.0 equiv.), tetrakis(acetonitrile) copper(I) tetrafluoroborate (15.7 mg, 0.05 mmol, 10 mol%), and 4,4'-dimethoxy-2,2'-bipyridine (10.8 mg, 0.05 mmol, 10 mol%) was added DMPU (2.5 mL, fresh bottle). The reaction was heated to 110°C and left to stir for 36 h under $N_2(g)$. Upon completion, the mixture was allowed to cool to room temperature, and then quenched with a stock solution of dimethyl terephthalate (1.5 mL, 0.0133 M solution in MeCN, 0.02 mmol). The resulting solution was sonicated, and a 50 µL aliquot was taken and diluted in a HPLC vial with MeCN (950 µL). Yields were determined by HPLC (λ = 235 nm) using % area and pre-determined K_x values for the major reaction components.

Entry	Conditions	HPLC area$_{IS}$ (%)	HPLC area sulfone 20 (%)	HPLC yield sulfone 20 (%)	HPLC area thiosulfonate 21 (%)	HPLC yield thiosulfonate 21 (%)	Mean ratio of 20:21
1	Section 4.6.1.2. mean value for fresh DMPU bottle	14.043	31.304 (mean)	**46.6**	5.677 (mean)	**7.5**	**6.2**
2	Use of benzenesulfinate intermediate	23.904	28.311	**24.6**	0.268	**0.2**	**123**

* Solution yields determined by HPLC (λ = 235 nm) using % area of internal standard, % area of reaction component, and pre-determined K_x values for the major reaction components (Section 3).

Conclusions: Based on the obtained HPLC yields of the thiosulfonate by-product for each entry, the yield of the thiosulfonate by-product appears to be generated more readily when a SO_2 source is used within the reaction. Upon switching to the sulfinate intermediate, and elimination of the acidifying SO_2 source, only trace quantities of the thiosulfonate are observed, with a 123:1 ratio compared with the desired sulfone product.

4.6.2. Identification of starting material fates in the synthesis of an unsymmetrical sulfone

4.6.2.1. HPLC analysis of commercial and synthesised reference compounds

In order to identify the fate of each reaction component, a model reaction for the formation of 1-methyl-4-(phenylsulfonyl)benzene from phenylboronic acid and 4-iodotoluene was chosen to study. Using a combination of commercially available and synthesised reference compounds, the major products of the reaction were identified. Also accounted for were all possible combinations of a regular Suzuki-Miyaura coupling, protodeboronation, and protodehalogenation. The HPLC reference retention times are shown below.

Entry	Compound name	Compound structure	Retention Time (min)
1	Benzene	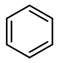	2.08
2	Sulfonyldibenzene **(20)**	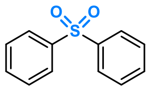	2.56
3	Toluene	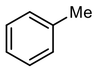	2.64
4	1-Methyl-4-(phenylsulfonyl)benzene **(3)**	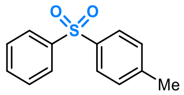	2.77
5	Iodobenzene **(22)**	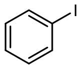	2.93
6	*S*-Phenyl benzenethiosulfonate **(21)**	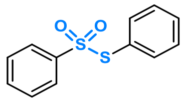	2.99
7	4-Iodotoluene **(2)**	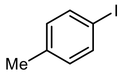	3.20
8	1,1'-Biphenyl	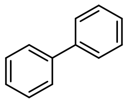	3.20
9	*S*-(*p*-tolyl) 4-methylbenzenethiosulfonate	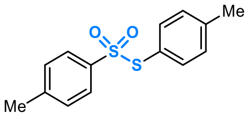	3.31
10	4-Methyl-1,1'-biphenyl	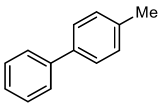	3.40
11	4,4'-Dimethyl-1,1'-biphenyl	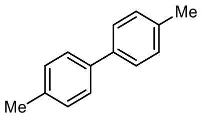	3.59

Once HPLC retention times were acquired, two model reactions were carried out, one for the synthesis of symmetrical sulfone sulfonyldibenzene **20**, and the other for the unsymmetrical sulfone, 1-methyl-4-(phenylsulfonyl)benzene **3**.

4.6.2.2. HPLC test reaction for the preparation of sulfonyldibenzene

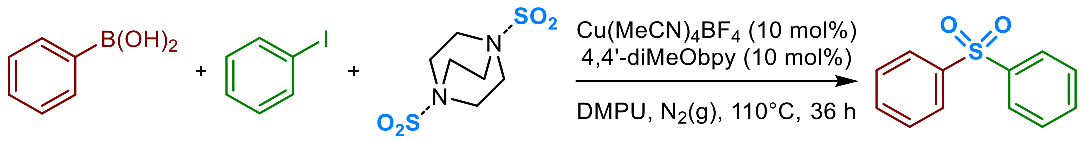

4 (3.0 equiv.) **22** (1.0 equiv.) (1.5 equiv.) **20** crude HPLC analysis

To a microwave vial containing phenylboronic acid (73.0 mg, 0.6 mmol, 3.0 equiv.), $Cu(MeCN)_4BF_4$ (6.3 mg, 0.02 mmol, 10 mol%), 4,4’-dimethoxy-2,2’-bipyridine (4.3 mg, 0.02 mmol, 10 mol%) and DABSO (72.0 mg, 0.3 mmol, 1.5 equiv.) and iodobenzene (22.0 µL, 0.2 mmol, 1.0 equiv.) was added DMPU (1 mL). The reaction was heated to 110 °C and left to stir for 36 h under N_2(g). Upon completion, the mixture was allowed to cool to room temperature, after which an aliquot was taken. This was diluted with acetonitrile to 1 mL and analysed by HPLC, noting the retention times of peaks in relation to the previously run references. The resulting HPLC spectrum is shown below, annotated with the identity of the major peaks.

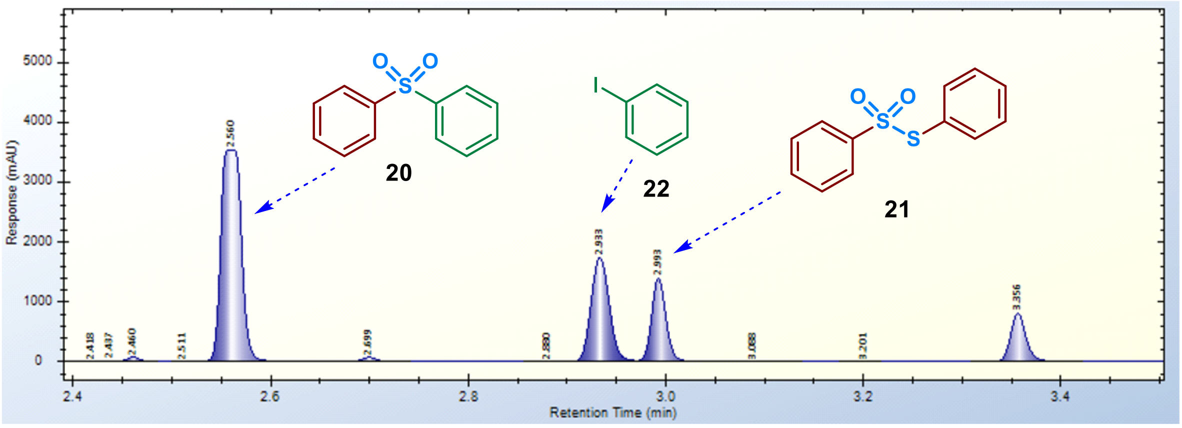

Major components within the reaction mixture were found to be the desired sulfone product sulfonyldibenzene (t_{ret} = 2.56 min), remaining iodobenzene starting material (t_{ret} = 2.93 min), and *S*-phenyl benzenethiosulfonate (t_{ret} = 2.99 min, compared with commercially available reference), arising from disproportionation of the benzenesulfinate intermediate.

4.6.2.3. HPLC test reaction for the preparation of 1-methyl-4-(phenylsulfonyl)benzene

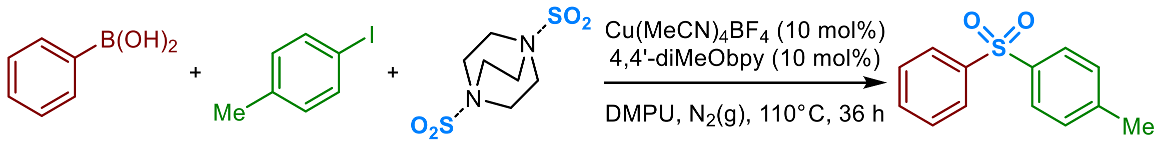

4 (3.0 equiv.) **2** (1.0 equiv.) (1.5 equiv.) **3** crude HPLC analysis

To a microwave vial containing phenylboronic acid (73.0 mg, 0.6 mmol, 3.0 equiv.), $Cu(MeCN)_4BF_4$ (6.3 mg, 0.02 mmol, 10 mol%), 4,4’-dimethoxy-2,2’-bipyridine (4.3 mg, 0.02 mmol, 10 mol%) and DABSO (72.0 mg, 0.3 mmol, 1.5 equiv.) was added a solution of 4-iodotoluene (44.0 mg, 0.2 mmol, 1.0 equiv.) in DMPU (1 mL). The reaction was heated to 110 °C and left to stir for 36 h under N_2(g). Upon completion, the mixture was allowed to cool to room temperature, after which an aliquot was taken. This was diluted with acetonitrile to 1 mL and analysed by HPLC, noting the retention times of

peaks in relation to the previously run references. The resulting HPLC spectrum is shown below, annotated with the identity of the major peaks.

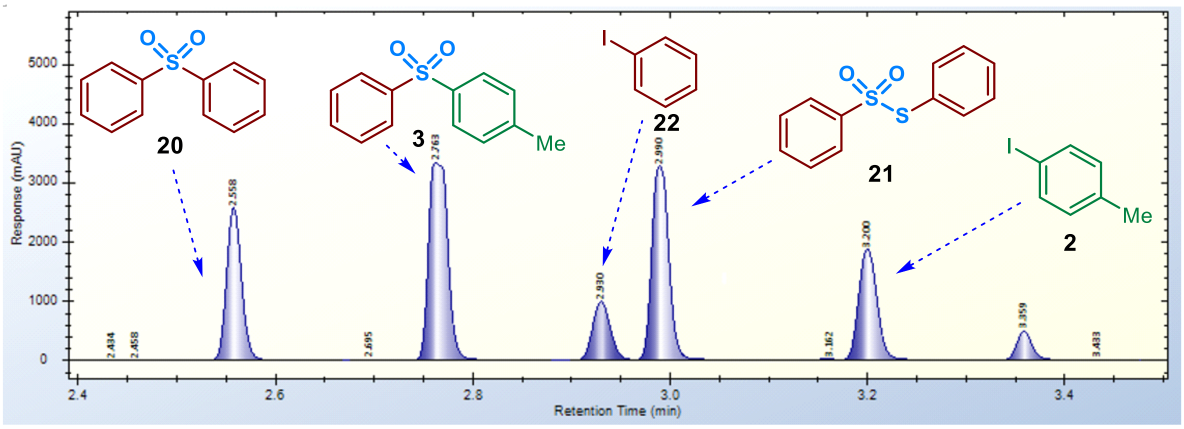

The expected major reaction components of desired unsymmetrical sulfone product 1-methyl-4-(phenylsulfonyl)benzene **3** (t_{ret} = 2.76 min), remaining 4-iodotoluene starting material **2** (t_{ret} = 3.20 min), and disproportionation product *S*-phenyl benzenethiosulfonate (t_{ret} = 2.99 min, compared with commercially available reference) were observed. However, additionally observed by-products were the undesired symmetrical sulfone sulfonyldibenzene **20** (t_{ret} = 2.56 min), with iodobenzene **22** also present (t_{ret} = 2.93 min). Due to the unsymmetrical nature of the product, these phenyl-containing by-products could only arise from reaction of the phenylboronic acid starting material, or the intermediate benzenesulfinate.

4.6.3. Mechanistic control experiments: undesired symmetrical sulfone formation

Following the discovery of the side-reaction leading for formation of an undesired symmetrical sulfone product , sulfonyldibenzene **20**, a number of mechanistic control experiments were carried out to aid in further understanding of the reaction, discussed below.

4.6.3.1. Confirming symmetrical product formation *does not* arise from iodide liberation from copper(III)

Based on the observation of iodobenzene within the reaction mixture, we initially proposed that iodide liberated from displacement by benzenesulfinate on the (4,4'-diMeObpy)Cu^{III}(Ph)(I) complex was reacting with boronic acid starting material, in a pathway similar to that described in the literature.[16] In order to test this hypothesis, it was proposed that addition of inorganic iodide into a mixture of phenylboronic acid and 1,4-diazabicyclo[2.2.2]octane bis(sulfur dioxide) could lead to formation of iodobenzene, and subsequent coupling with the intermediate formed benzenesulfinate would lead to formation of sulfonyldibenzene, if the mechanism was occurring by this pathway. In order to account for both an oxidative pathway, and inert atmosphere pathway, one reaction was carried out under an air atmosphere, and the other under N_2(g).

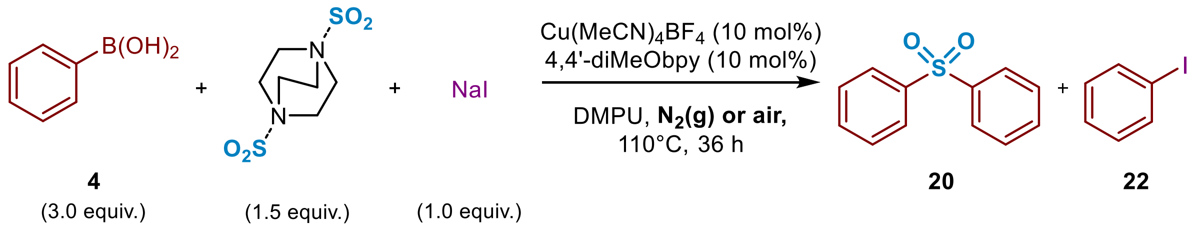

To two microwaves vial containing phenylboronic acid (73.2 mg, 0.6 mmol, 3.0 equiv.), tetrakis(acetonitrile) copper(I) tetrafluoroborate (6.3 mg, 0.02 mmol, 10 mol%), 4,4'-dimethoxy-2,2'-bipyridine (4.3 mg, 0.02 mmol, 10 mol%), 1,4-diazabicyclo[2.2.2]octane bis(sulfur dioxide) adduct (72.1 mg, 0.3 mmol, 1.5 equiv.) and sodium iodide (30.0 mg, 0.2 mmol, 1.0 equiv.) was added DMPU (1 mL). Both vials were heated to 110°C, and left to stir for 36 h, one vial open to **air** and one under **N_2(g)**. Upon completion, the mixture was allowed to cool to room temperature, after which an aliquot was taken. This was diluted with MeCN to 1 mL and analysed by HPLC, noting whether the symmetrical sulfone product sulfonyldibenzene **20** (t_{ret} = 2.56 min) or iodobenzene **22** (t_{ret} = 2.93 min) were present.

Entry	Atmosphere	HPLC yield sulfone 20 (%)	HPLC yield iodobenzene 22 (%)
1	**N_2**	0	trace
2	**air**	0	trace

Conclusions: Although trace amounts of iodobenzene were detected (<0.3% HPLC area), it can be concluded that symmetrical sulfone formation is not occurring from the iodide species liberated from (4,4'-diMeObpy)Cu^{III}(Ph)(I), either under oxidative or inert conditions.

4.6.3.2. Confirming symmetrical product formation *does not* arise from formation of molecular iodine

In addition to our investigation using sodium iodide, the inherent ability of iodide ions to undergo oxidation to molecular iodine is well known, and methods using copper catalysis in combination with molecular iodine are known to efficiently convert arylboronic acids to the corresponding aryl iodides.[17] To rule out this pathway, a similar reaction of phenylboronic acid and 1,4-diazabicyclo[2.2.2]octane bis(sulfur dioxide) with an iodine additive was performed.

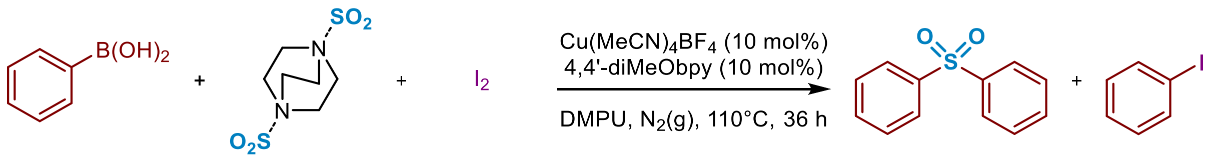

4 (3.0 equiv.) (1.5 equiv.) (1.0 equiv.) **20** **22**

To a microwave vial containing phenylboronic acid (73.2 mg, 0.6 mmol, 3.0 equiv.), tetrakis(acetonitrile) copper(I) tetrafluoroborate (6.3 mg, 0.02 mmol, 10 mol%), 4,4'-dimethoxy-2,2'-bipyridine (4.3 mg, 0.02 mmol, 10 mol%), 1,4-diazabicyclo[2.2.2]octane bis(sulfur dioxide) adduct (72.1 mg, 0.3 mmol, 1.5 equiv.) and iodine (50.8 mg, 0.2 mmol, 1.0 equiv.) was added DMPU (1 mL). The reaction was heated to 110°C and left to stir for 36 h under N_2(g). Upon completion, the mixture was allowed to cool to room temperature, after which an aliquot was taken. This was diluted with MeCN to 1 mL and analysed by HPLC, noting whether the symmetrical sulfone product sulfonyldibenzene (t_{ret} = 2.56 min) or iodobenzene (t_{ret} = 2.93 min) were present.

Entry	Atmosphere	HPLC yield sulfone 20 (%)	HPLC yield iodobenzene 22 (%)
1	**N_2**	trace	~6

Conclusions: Reaction with molecular iodine results in production of a greater amount of iodobenzene **Z** from the boronic acid starting material, however this is not in a sufficient amount to propose this is the mechanistic pathway. The trace amounts of symmetrical sulfone product observed lead us to conclude that iodobenzene formation is not contributing to this side-reaction.

4.6.3.3. Confirming symmetrical product formation *does not* arise from desulfinylation of a sulfinate

Recent reports by Willis and co-workers have discovered desulfinylative couplings of sulfinate salts, with the extrusion of SO_2(g) in the process. Although reports of these transformations have not been reported with copper(I), just palladium(0)/(II), we did not discard this potential pathway for formation of the symmetrical sulfone. It could be possible that a desulfinylative process involving one equivalent of benzenesulfinate intermediate, reacting with another equivalent could lead to the by-product. In order to investigate this, a previously used reaction (Section 4.1.) could be used to disprove this pathway.

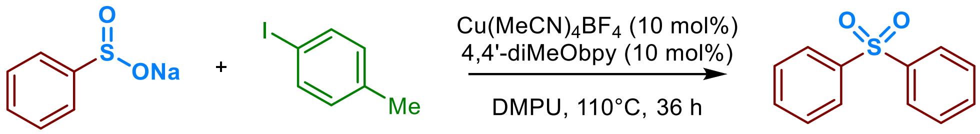

To a microwave vial containing sodium benzenesulfinate (49.2 mg, 0.3 mmol, 3.0 equiv.), $Cu(MeCN)_4BF_4$ (3.2 mg, 0.01 mmol, 10 mol%) and 4,4'-dimethoxy-2,2'-bipyridine (2.2 mg, 0.01 mmol, 10 mol%) was added a solution of 4-iodotoluene (21.8 mg, 0.1 mmol, 1.0 equiv.) in DMPU (0.5 mL). The reaction was heated to 110 °C and left to stir for 36 h under N_2(g). Upon completion, the mixture was allowed to cool to room temperature, and then quenched with a stock solution of dimethyl terephthalate (1.5 mL, 0.0133 M solution in acetonitrile, 0.02 mmol). The resulting solution was sonicated, and a 50 µL aliquot was taken and diluted in a HPLC vial with acetonitrile (950 µL). Yields were determined by HPLC (λ = 235 nm) using % area and pre-determined K_x values for the major reaction components.

Entry	HPLC area$_{IS}$ (%)	HPLC area sulfone 20 (%)	HPLC yield sulfone 20 (%)
1	14.312	0.084	<1

* Solution yields determined by HPLC (λ = 235 nm) using % area of internal standard, % area of reaction component, and pre-determined K_x values for the major reaction components (Section 3).

Conclusions: The presence of majority unsymmetrical sulfone **3** with only trace amounts of symmetrical sulfone **20**, when starting from the sulfinate intermediate, suggests that formation of the symmetrical sulfone from the sulfinate intermediate is not a major reaction pathway. We can therefore conclude the side-reaction is originating from the phenylboronic acid coupling partner.

4.6.3.4. Reaction of phenylboronic acid with 1,4-diazabicyclo[2.2.2]octane bis(sulfur dioxide) under copper(I)-catalysis conditions

Given the symmetrical sulfone by-product **20** had been confirmed to not arise from iodobenzene formation and desulfinylation of the intermediate sulfinate, we next turned to the potential of the intermediate sulfinate reacting directly with the boronic acid starting material. In a reaction in which the stoichiometries were carefully selected such that 1 equivalent of boronic acid would react to form the benzenesulfinate intermediate and 1 equivalent of the boronic acid remained, we tested this hypothesis.

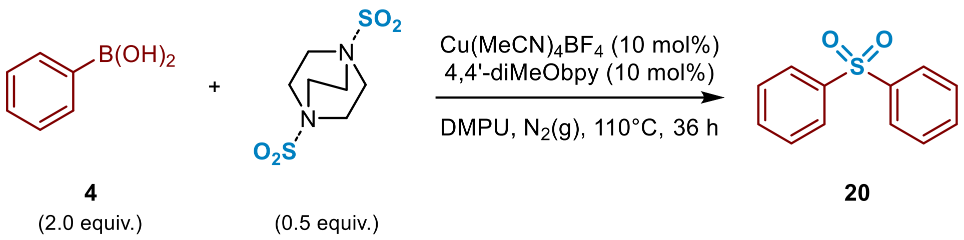

To a microwave vial containing phenylboronic acid (24.2 mg, 0.2 mmol, 2.0 equiv.), $Cu(MeCN)_4BF_4$ (3.2 mg, 0.01 mmol, 10 mol%), 4,4'-dimethoxy-2,2'-bipyridine (2.2 mg, 0.01 mmol, 10 mol%), and DABSO (12.0 mg, 0.05 mmol, 0.5 equiv.) was added DMPU (0.5 mL). The reaction was heated to 110 °C and left to stir for 36 h under N_2(g). Upon completion, the mixture was allowed to cool to room temperature, and then quenched with a stock solution of dimethyl terephthalate (1.5 mL, 0.0133 M solution in acetonitrile, 0.02 mmol). The resulting solution was sonicated, and a 50 µL aliquot was taken and diluted in a HPLC vial with acetonitrile (950 µL). Yields were determined by HPLC (λ = 235 nm) using % area and pre-determined K_x values for the major reaction components.

Entry	HPLC area$_{IS}$ (%)	HPLC area sulfone 20 (%)	HPLC yield sulfone 20 (%)
1	22.699	20.932	19

* Solution yields determined by HPLC (λ = 235 nm) using % area of internal standard, % area of reaction component, and pre-determined K_x values for the major reaction components (Section 3).

Conclusions: Observed formation of symmetrical sulfone **20** in 19% HPLC yield confirms the product is arising from direct reaction of the benzenesulfinate intermediate with phenylboronic acid. The exact nature of this process cannot be established based on this limited experimental study, but literature precedent suggests it could be occurring through a Cu^{II}-catalysed process.[18] Given the maximum HPLC yield observed for this side reaction after 36 h throughout this mechanistic investigation was 22%, a 19% yield for this control experiment is strong evidence that the intermediate sulfinate is reacting with boronic acid starting material to yield the symmetrical sulfone product.

4.6.3.5. Reaction of phenylboronic acid with sodium benzenesulfinate under oxidative copper(I)-catalysis conditions

Following the proposal of a Cu^{II}-catalysed cross-coupling of benzenesulfinate with phenylboronic acid to yield symmetrical sulfone **20**, we decided to see if simply exposing this reaction under air would yield the identical product, presumably through oxidation of Cu^{I} to Cu^{II}.

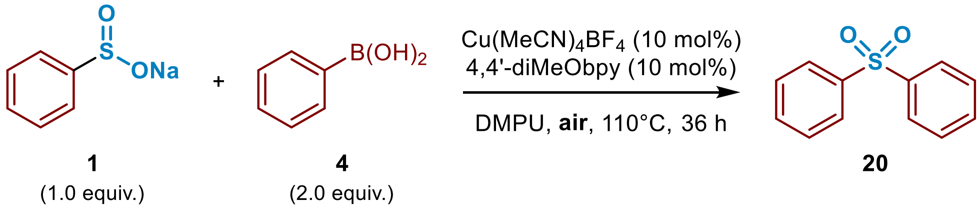

To a microwave vial containing phenylboronic acid (24.0 mg, 0.2 mmol, 2.0 equiv.), sodium benzenesulfinate (16.4 mg, 0.1 mmol, 1.0 equiv.), $Cu(MeCN)_4BF_4$ (3.2 mg, 0.01 mmol, 10 mol%), and 4,4'-dimethoxy-2,2'-bipyridine (2.2 mg, 0.01 mmol, 10 mol%) was added DMPU (0.5 mL). The reaction was heated to 110 °C and left to stir for 36 h under air. Upon completion, the mixture was allowed to cool to room temperature, and then quenched with a stock solution of dimethyl terephthalate (1.5 mL, 0.0133 M solution in acetonitrile, 0.02 mmol). The resulting solution was sonicated, and a

50 µL aliquot was taken and diluted in a HPLC vial with acetonitrile (950 µL). Yields were determined by HPLC (λ = 235 nm) using % area and pre-determined K_x values for the major reaction components.

Entry	HPLC area$_{IS}$ (%)	HPLC area sulfone 20 (%)	HPLC yield sulfone 20 (%)
1	24.164	26.034	22

* Solution yields determined by HPLC (λ = 235 nm) using % area of internal standard, % area of reaction component, and pre-determined K_x values for the major reaction components (Section 3).

Conclusions: Observation of symmetrical sulfone **20** in a 22% yield suggests the oxidation of Cu^{I} to Cu^{II} is occurring in the reaction mixture. This yield is identical to the maximum yield of symmetrical sulfone observed when the overall sulfonylative Suzuki-Miyaura reaction is carried out for the synthesis of 1-methyl-4-(phenylsulfonyl)benzene under a N_2(g) atmosphere, which suggests the oxidation of Cu^{I} to Cu^{II} is not caused by air – otherwise we would likely see an increased yield under air.

4.6.3.6. Reaction of phenylboronic acid with *S*-phenyl benzenethiosulfonate under copper(I)-catalysis conditions

Given the known reversion process of thiosulfonates to their corresponding sulfinate products in the presence of a base,[19] we decided to investigate whether the observed disproportionation product, *S*-phenyl benzenethiosulfonate, in the presence of the base 1,4-diazabicyclo[2.2.2]octane, could also contribute to this side reaction.

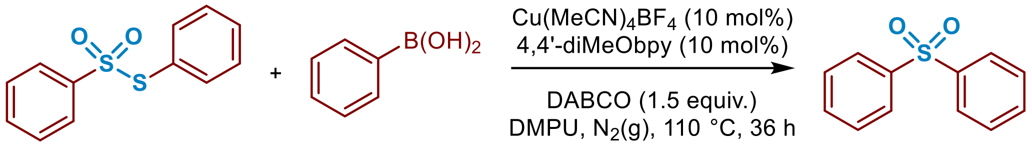

21 (1.0 equiv.)   **4** (1.0 equiv.)   **20**

To two microwave vials, one containing 1,4-diazabicyclo[2.2.2]octane (34.0 mg, 0.3 mmol, 1.5 equiv.) and the other empty, was added phenylboronic acid (24.0 mg, 0.2 mmol, 1.0 equiv.), *S*-phenyl benzenethiosulfonate (50.0 mg, 0.2 mmol, 1.0 equiv.), $Cu(MeCN)_4BF_4$ (6.3 mg, 0.02 mmol, 10 mol%), and 4,4'-dimethoxy-2,2'-bipyridine (4.3 mg, 0.02 mmol, 10 mol%). To both vials was then added DMPU (0.5 mL). The reactions were heated to 110 °C and left to stir for 36 h under N_2(g). Upon completion, the mixtures were allowed to cool to room temperature, and were then quenched with a stock solution of dimethyl terephthalate (1.5 mL, 0.0133 M solution in acetonitrile, 0.02 mmol). The resulting solutions were sonicated, and a 50 µL aliquot was taken from each and diluted in a HPLC vial with acetonitrile (950 µL). Yields were determined by HPLC (λ = 235 nm) using % area and pre-determined K_x values for the major reaction components.

Entry	Base	HPLC area$_{IS}$ (%)	HPLC area sulfone 20 (%)	HPLC yield sulfone 20 (%)
1	–	18.428	2.134	2
2	DABCO	13.979	9.589	14

* Solution yields determined by HPLC (λ = 235 nm) using % area of internal standard, % area of reaction component, and pre-determined K_x values for the major reaction components (Section 3).

Conclusions: Observation of symmetrical sulfone **20** formation again confirms that the sulfinate species is involved in a cross-coupling process with the phenylboronic acid starting material. The presence of base in the reaction mixture, known to promote the reversion of thiosulfonates to sulfinates, results in a much higher yield of the symmetrical sulfone product. As the base DABCO is

also present within the reaction mixture of the sulfonylative Suzuki-Miyaura reaction, it could be that both the intermediate sulfinate and thiosulfonate lead to the by-product.

4.6.3.7. Reaction of phenylboronic acid and 4-iodotoluene with 1,4-diazabicyclo[2.2.2]octane bis(sulfur dioxide) under oxidative copper(I)-catalysis conditions

Given our observations that symmetrical sulfone **20** is formed in the same yield when comparing reaction of boronic acid and sulfinate under air, and that of the overall reaction of phenylboronic acid and 4-iodotoluene for the synthesis of 1-methyl-4-(phenylsulfonyl)benzene under N_2(g), it appears that the Cu^{II} species is not being formed by aerobic oxidation of Cu^{I}. To further confirm this, we decided to perform the overall transformation under air.

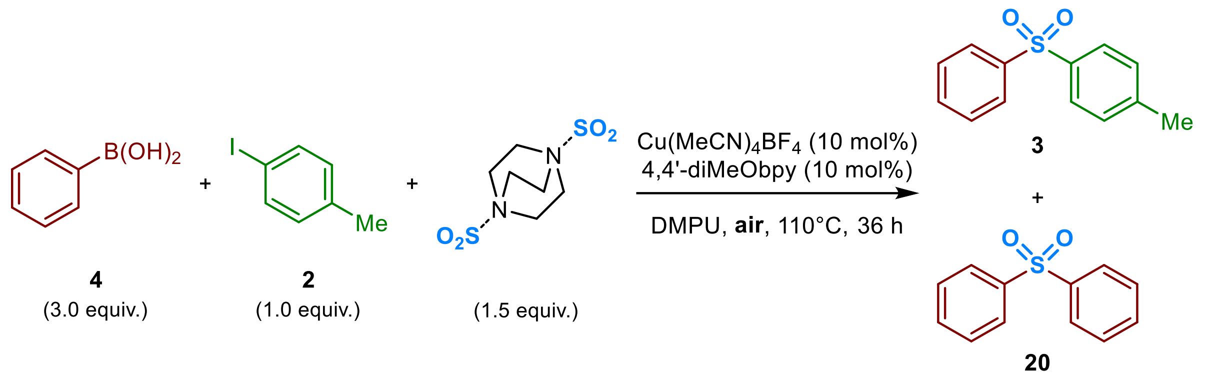

To a microwave vial containing phenylboronic acid (37.0 mg, 0.3 mmol, 3.0 equiv.), $Cu(MeCN)_4BF_4$ (3.2 mg, 0.01 mmol, 10 mol%), 4,4'-dimethoxy-2,2'-bipyridine (2.2 mg, 0.01 mmol, 10 mol%) and DABSO (36.0 mg, 0.15 mmol, 1.5 equiv.) under air was added a solution of 4-iodotoluene (21.8 mg, 0.1 mmol, 1.0 equiv.) in DMPU (0.5 mL). The reaction was heated to 110 °C and left to stir for 36 h under air. Upon completion, the mixture was allowed to cool to room temperature, and then quenched with a stock solution of dimethyl terephthalate (1.5 mL, 0.0133 M solution in acetonitrile, 0.02 mmol). The resulting solution was sonicated, and a 50 µL aliquot was taken and diluted in a HPLC vial with acetonitrile (950 µL). Yields were determined by HPLC (λ = 235 nm) using % area and pre-determined K_x values for the major reaction components.

Entry	HPLC area$_{IS}$ (%)	HPLC area sulfone 3 (%)	HPLC yield sulfone 3 (%)	HPLC area sulfone 20 (%)	HPLC yield sulfone 20 (%)
1	15.662	1.015	1	1.393	2

* Solution yields determined by HPLC (λ = 235 nm) using % area of internal standard, % area of reaction component, and pre-determined K_x values for the major reaction components (Section 3).

Conclusions: It appears that when an air atmosphere is used in place of an inert N_2(g) atmosphere, the reaction is significantly inhibited. This suggests that the oxidation of Cu^{I} to Cu^{II} does not occur via oxygen – it instead appears to be caused by a reaction component – feasibly *via* a disproportionation of Cu^{I} to Cu^{II} and Cu^{0}. This effect has also been observed when carrying out the chemistry in a glovebox, with no oxygen present.

4.6.3.8. Reaction of phenylboronic acid (4) and 4-iodotoluene (2) with 1,4-diazabicyclo[2.2.2]octane bis(sulfur dioxide) in degassed solvent under copper(I)-catalysis conditions

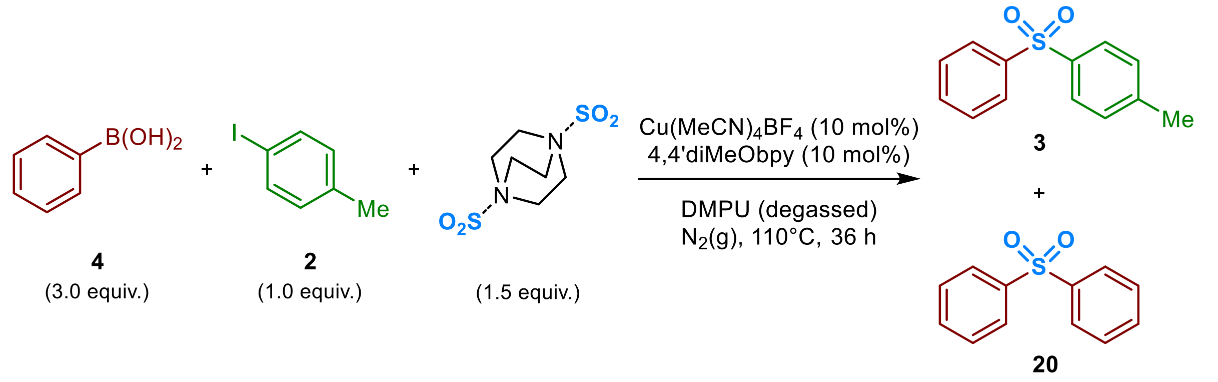

To a microwave vial containing phenylboronic acid (37.0 mg, 0.3 mmol, 3.0 equiv.), $Cu(MeCN)_4BF_4$ (3.2 mg, 0.01 mmol, 10 mol%), 4,4'-dimethoxy-2,2'-bipyridine (2.2 mg, 0.01 mmol, 10 mol%) and DABSO (36.0 mg, 0.15 mmol, 1.5 equiv.) was added a solution of 4-iodotoluene (21.8 mg, 0.1 mmol, 1.0 equiv.) in DMPU (0.5 mL). Prior to heating, the reaction mixture was degassed (x 3) using the freeze-pump-thaw method, freezing the reaction mixture in an acetone-dry ice base (–78 °C). The sequence followed involved freezing (5 min), evacuating the vial whilst still frozen (5 min), defrosting under vacuum and refilling with N_2(g) upon thawing. The reaction was then heated to 110 °C and left to stir for 36 h under N_2(g). Upon completion, the mixture was allowed to cool to room temperature, and then quenched with a stock solution of dimethyl terephthalate (1.5 mL, 0.0133 M solution in acetonitrile, 0.02 mmol). The resulting solution was sonicated, and a 50 µL aliquot was taken and diluted in a HPLC vial with acetonitrile (950 µL). Yields were determined by HPLC (λ = 235 nm) using % area and pre-determined K_x values for the major reaction components.

Entry	Conditions	HPLC area$_{IS}$ (%)	HPLC area sulfone 3 (%)	HPLC yield sulfone 3 (%)	HPLC area sulfone 20 (%)	HPLC yield sulfone 20 (%)
1	Standard	10.444	22.517	**41**	11.207	**22**
2	Degassed solvent	10.201	21.415	**39**	9.043	**18**

* Solution yields determined by HPLC (λ = 235 nm) using % area of internal standard, % area of reaction component, and pre-determined K_x values for the major reaction components (Section 3).

Conclusions: Given there was very little difference in yields of both sulfones when comparing a reaction using degassed solvent to a reference reaction with no modification to literature conditions, oxidation of Cu^{I} to Cu^{II} does not appear to occur from the presence of air or oxygen.

4.6.3.9. Reaction of phenylboronic acid (4) and 4-iodotoluene (2) with 1,4-diazabicyclo[2.2.2]octane bis(sulfur dioxide) under copper(II)-catalysis conditions

Following our observation that Cu^{I} was being converted into Cu^{II} and facilitating the unwanted side reaction of benzenesulfinate with phenylboronic acid, a comparative reaction using a Cu^{II} source was carried out to observe whether the Cu^{I}-derived unsymmetrical sulfone product would still form – and thus would confirm a combination of Cu^{I}, Cu^{II}, and Cu^{III} being present within the reaction mixture.

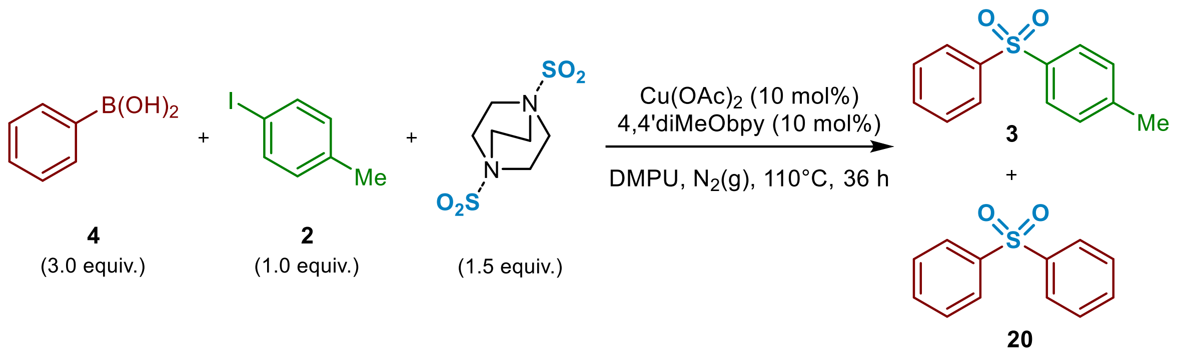

To a microwave vial containing phenylboronic acid (37.0 mg, 0.3 mmol, 3.0 equiv.), DABSO (36.0 mg, 0.15 mmol, 1.5 equiv.), $Cu(OAc)_2$ (1.8 mg, 0.01 mmol, 10 mol%), and 4,4'-dimethoxy-2,2'-bipyridine (2.2 mg, 0.01 mmol, 10 mol%) was added a solution of 4-iodotoluene (21.8 mg, 0.1 mmol, 1.0 equiv.) DMPU (0.5 mL). The reaction was heated to 110 °C and left to stir for 36 h under N_2(g). Upon completion, the mixture was allowed to cool to room temperature, and then quenched with a stock solution of dimethyl terephthalate (1.5 mL, 0.0133 M solution in acetonitrile, 0.02 mmol). The resulting solution was sonicated, and a 50 µL aliquot was taken and diluted in a HPLC vial with acetonitrile (950 µL). Yields were determined by HPLC (λ = 235 nm) using % area and pre-determined K_x values for the major reaction components. A comparison reaction under Cu^{I} catalysis is shown as Entry 1 in the table.

Entry	Cu catalyst	Cu oxidation state	HPLC area$_{IS}$ (%)	HPLC area sulfone 3 (%)	HPLC yield sulfone 3 (%)	HPLC area sulfone 20 (%)	HPLC yield sulfone 20 (%)
1	$Cu(MeCN)_4BF_4$	+1	10.444	22.517	**41**	11.207	**22**
2	$Cu(OAc)_2$	+2	13.139	18.208	**26**	13.912	**22**

* Solution yields determined by HPLC (λ = 235 nm) using % area of internal standard, % area of reaction component, and pre-determined K_x values for the major reaction components (Section 3).

Conclusions: Based on the observation of both sulfone products, one originating from the Cu^{I} catalysis and the other from Cu^{II}, it can be concluded that the Cu^{II} cycle generates Cu^{I} by-products (such as in a Chan-Lam reaction), which are able to catalyse the reaction to generate the unsymmetrical sulfone **3**.

4.6.3.10. Timecourse of reaction of phenylboronic acid (4) with 4-iodotoluene (2) and 1,4-diazabicyclo[2.2.2]octane bis(sulfur dioxide) adduct under copper(I) catalysis

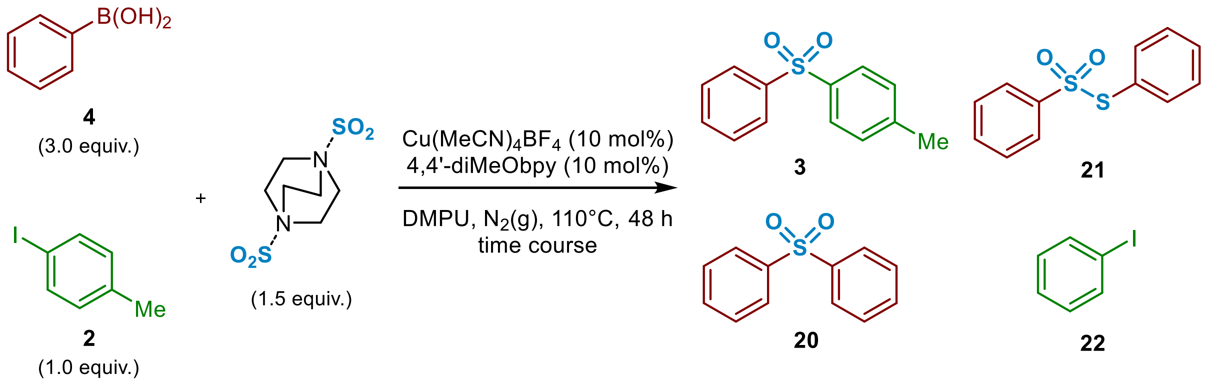

To a microwave vial containing phenylboronic acid (73.0 mg, 0.6 mmol, 3.0 equiv.), $Cu(MeCN)_4BF_4$ (6.3 mg, 0.02 mmol, 10 mol%), 4,4'-dimethoxy-2,2'-bipyridine (4.3 mg, 0.02 mmol, 10 mol%), DABSO

(72.0 mg, 0.3 mmol, 1.5 equiv.) and dimethyl terephthalate (7.8 mg, 0.04 mmol) was added a solution of 4-iodotoluene (44.0 mg, 0.2 mmol, 1.0 equiv.) in DMPU (1 mL). The reaction was heated to 110 °C under N_2(g). Over a period of 48 h, reaction aliquots were taken, diluted to ~1 mL with acetonitrile, and analysed by HPLC. Yields were determined by HPLC (λ = 235 nm) using % area and pre-determined K_x values for the major reaction components. Reaction time -points were as follows, and yields of major reaction components are tabulated below, accompanied by a plot of the timecourse.

t = 0, 2, 5, 10, 15, 20, 30, 45, 60, 75, 90, 105, 120, 150, 180, 210, 240, 300, 360, 420, 480, 1440, 1560, 1680, 1800 and 2880 min

Time (min)	HPLC yield sulfone 3 (%)	HPLC yield sulfone 20 (%)	HPLC yield thiosulfonate 21 (%)	HPLC yield 4-iodotoluene 2 (%)	HPLC yield iodobenzene 22 (%)
0	0.00	0.00	0.00	76.64	0.00
2	1.05	0.00	0.06	67.51	0.00
5	2.49	0.15	1.01	63.08	0.29
10	4.36	0.29	2.13	59.57	0.57
15	6.02	0.40	3.23	49.80	0.85
20	7.09	0.49	3.30	56.60	1.08
30	9.09	0.67	4.61	50.31	1.58
45	11.08	0.90	6.20	51.76	2.15
60	12.66	1.13	7.31	51.36	2.63
75	14.01	1.37	8.31	46.88	3.04
90	15.17	1.60	9.10	44.18	3.39
105	16.04	1.79	9.71	46.60	3.61
120	16.98	2.06	10.62	43.66	3.91
150	18.45	2.41	13.10	43.62	4.31
180	19.65	2.79	12.78	49.58	4.64
210	20.71	3.17	13.88	45.42	4.96
240	21.56	3.58	13.68	41.48	5.20
300	22.72	4.34	14.87	37.11	5.66
360	24.17	4.86	14.44	38.12	5.88
420	25.31	5.47	15.18	36.70	6.13
480	26.08	6.09	14.70	35.02	6.31
1440	36.07	14.14	12.85	23.96	8.76
1560	35.64	15.37	12.61	20.54	7.91
1680	34.80	16.37	13.43	20.20	8.67
1800	37.02	17.21	12.53	20.81	9.40
2880	40.03	23.49	9.05	13.69	10.57

* Solution yields determined by HPLC (λ = 235 nm) using % area of internal standard, % area of reaction component, and pre-determined K_x values for the major reaction components (Section 3).

$Cu(MeCN)_4BF_4$ (10 mol%), 4,4'-diMeObpy (10 mol%)

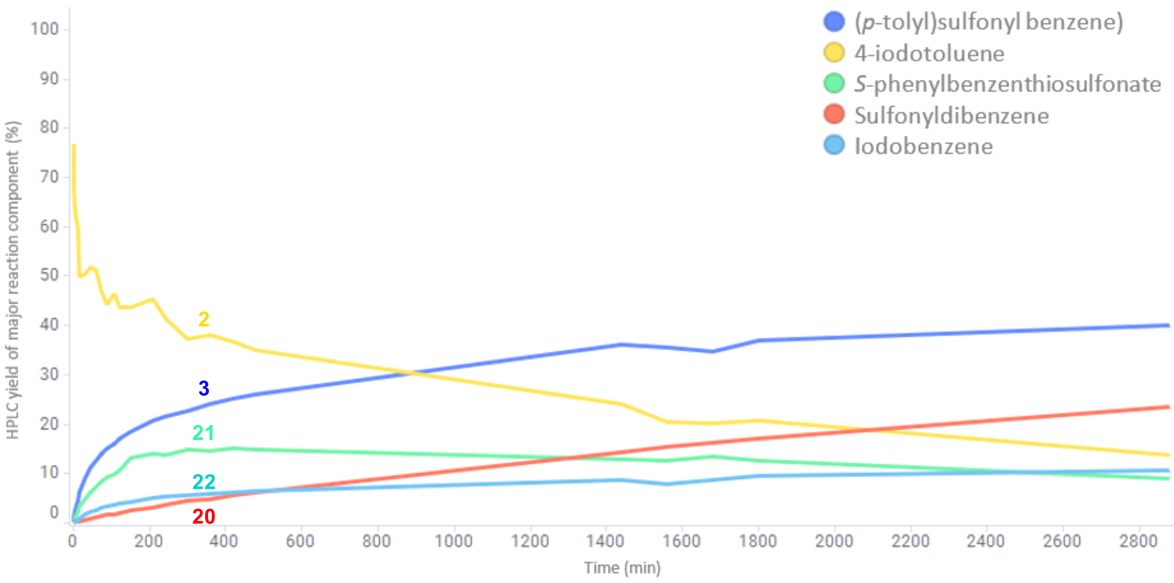

4.6.3.11. Timecourse of reaction of phenylboronic acid (4) with 4-iodotoluene (2) and 1,4-diazabicyclo[2.2.2]octane bis(sulfur dioxide) adduct under copper(II) catalysis

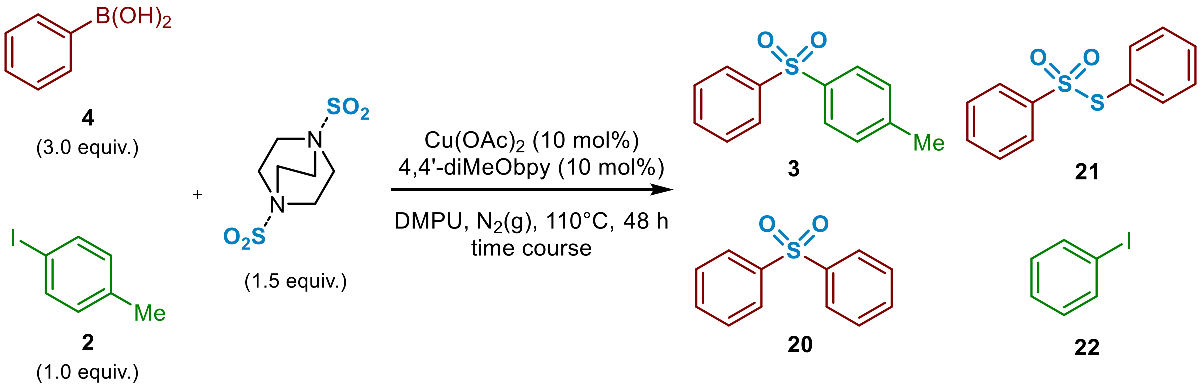

To a microwave vial containing phenylboronic acid (73.2 mg, 0.6 mmol, 3.0 equiv.), $Cu(OAc)_2$ (3.6 mg, 0.02 mmol, 10 mol%), 4,4'-dimethoxy-2,2'-bipyridine (4.3 mg, 0.02 mmol, 10 mol%), DABSO (72.1 mg, 0.3 mmol, 1.5 equiv.) and dimethyl terephthalate (7.8 mg, 0.04 mmol) was added a solution of 4-iodotoluene (43.6 mg, 0.2 mmol, 1.0 equiv.) in DMPU (1 mL). The reaction was heated to 110 °C under N_2(g). Over a period of 48 h, reaction aliquots were taken, diluted to ~1 mL with acetonitrile acetonitrile, and analysed by HPLC. Yields were determined by HPLC (λ = 235 nm) using % area and pre-determined K_x values for the major reaction components. Reaction time -points were as follows, and yields of major reaction components are tabulated below, accompanied by a plot of the timecourse.

t = 0, 2, 5, 10, 15, 20, 30, 45, 60, 75, 90, 105, 120, 150, 180, 210, 240, 300, 360, 420, 480, 1440, 1560, 1680, 1800 and 2880 min

Time (min)	HPLC yield sulfone 3 (%)	HPLC yield sulfone 20 (%)	HPLC yield thiosulfonate 21 (%)	HPLC yield 4-iodotoluene 2 (%)	HPLC yield iodobenzene 22 (%)
0	0.00	0.03	0.00	57.23	0.00
2	0.00	0.04	0.00	68.12	0.00
5	0.44	0.04	0.19	67.10	0.00
10	1.19	0.36	3.83	61.09	0.18
15	2.86	0.60	4.36	68.53	0.34
20	4.46	0.77	5.93	62.12	0.53
30	6.83	1.05	7.34	61.04	0.99
45	9.69	1.44	8.78	61.45	1.74
60	11.87	1.79	12.78	58.50	2.35
75	13.83	2.17	13.87	59.25	2.94
90	15.68	2.62	15.89	56.81	3.45
105	17.28	3.03	17.76	52.65	3.91
120	18.62	3.41	18.27	54.52	4.45
150	21.38	4.26	19.21	53.01	5.00
180	23.43	5.07	20.68	49.68	5.49
210	24.88	5.60	22.83	50.56	5.89
240	25.53	6.12	22.66	45.42	6.10
300	27.76	7.16	24.27	44.59	6.59
360	29.67	8.12	25.50	43.94	7.07
420	30.99	8.87	26.17	43.73	7.55
480	31.88	9.47	25.67	42.10	7.41
1440	36.18	15.53	20.12	28.35	11.49
1560	37.53	15.88	18.29	28.54	9.40
1680	38.04	16.15	18.96	27.67	11.32
1800	38.10	16.47	16.46	27.09	9.23
2880	38.82	19.31	10.64	19.04	8.90

* Solution yields determined by HPLC (λ = 235 nm) using % area of internal standard, % area of reaction component, and pre-determined K_x values for the major reaction components (Section 3).

$Cu(OAc)_2$ (10 mol%), 4,4'-diMeObpy (10 mol%)

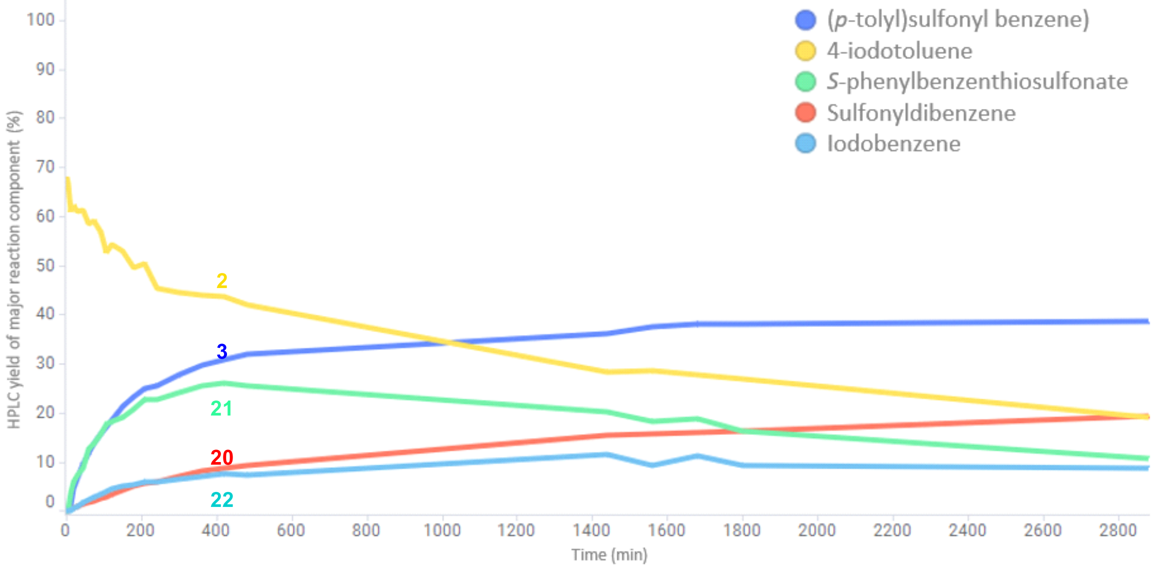

5. References

1. L. Martial and L. Bischoff, *Org. Synth.*, **2013**, *90*, 301-305.
2. Y. Chen and M. C. Willis, *Chem. Sci.*, **2017**, *8*, 3249-3253.
3. P. K. Shyam, Y. K. Kim, C. Lee and H.-Y. Jang, *Adv. Synth. Catal.*, **2016**, *358*, 56-61.
4. Y. Zheng, F.-L. Qing, Y. Huang and X.-H. Xu, *Adv. Synth. Catal.*, **2016**, *358*, 3477-3481.
5. H. Liu, B. Yin, Z. Gao, Y. Li and H. Jiang, *Chem. Commun.*, **2012**, *48*, 2033-2035.
6. J. F. Mariategui and K. Niedenzu, *J. Organomet. Chem.*, **1989**, *369*, 137-145.
7. R. M. P. Veenboer, A. Collado, S. Dupuy, T. Lebl, L. Falivene, L. Cavallo, D. B. Cordes, A. M. Z. Slawin, C. S. J. Cazin and S. P. Nolan, *Organometallics*, **2017**, *36*, 2861-2869.
8. A. Pituckchon, Further Investigation of Fluoboric Acid in Sandstone Acidizing Using 11B And 19F NMR MSc Thesis, Texas A&M University, 2014.
9. M. Güizado-Rodríguez, A. Flores-Parra, S. A. Sánchez-Ruiz, R. Tapia-Benavides, R. Contreras and V. I. Bakhmutov, *Inorg. Chem.*, **2001**, *40*, 3243-3246.
10. *Patent:* US9452425, 2016.
11. H. Saijo, M. Ohashi and S. Ogoshi, *J. Am. Chem. Soc.*, **2014**, *136*, 15158-15161.
12. R. Chandrasekaran, S. Perumal and D. A. Wilson, *Magn. Reson. Chem.*, **1989**, *27*, 360-367.
13. N. Umierski and G. Manolikakes, *Org. Lett.*, **2013**, *15*, 188-191.
14. X. Yang, L. Shi and H. Fu, *Synlett*, **2014**, *25*, 847-852.
15. J. M. Baskin and Z. Wang, *Org. Lett.*, **2002**, *4*, 4423-4425.
16. G. Zhang, G. Lv, L. Li, F. Chen and J. Cheng, *Tetrahedron Lett.*, **2011**, *52*, 1993-1995.
17. Y.-L. Ren, X.-Z. Tian, C. Dong, S. Zhao, J. Wang, M. Yan, X. Qi and G. Liu, *Catal. Commun*, **2013**, *32*, 15-17.
18. F. Huang and R. A. Batey, *Tetrahedron*, **2007**, *63*, 7667-7672.
19. P. K. Shyam and H.-Y. Jang, *J. Org. Chem.*, **2017**, *82*, 1761-1767.
